# Antibacterial Indole Diketopiperazine Alkaloids from the Deep-Sea Cold Seep-Derived Fungus *Aspergillus chevalieri*

**DOI:** 10.3390/md21030195

**Published:** 2023-03-22

**Authors:** Li-Hong Yan, Feng-Yu Du, Xiao-Ming Li, Sui-Qun Yang, Bin-Gui Wang, Xin Li

**Affiliations:** 1 CAS and Shandong Province Key Laboratory of Experimental Marine Biology, Institute of Oceanology, Chinese Academy of Sciences, Nanhai Road 7, Qingdao 266071, China; 2 University of Chinese Academy of Sciences, Yuquan Road 19A, Beijing 100049, China; 3 College of Chemistry and Pharmacy, Qingdao Agricultural University, Changcheng Road 700, Qingdao 266109, China; 4 Laboratory of Marine Biology and Biotechnology, Qingdao National Laboratory for Marine Science and Technology, Wenhai Road 1, Qingdao 266237, China; 5 Center for Ocean Mega-Science, Chinese Academy of Sciences, Nanhai Road 7, Qingdao 266071, China

**Keywords:** *Aspergillus chevalieri*, deep-sea cold seep, indole diketopiperazine, antibacterial activity

## Abstract

A large body of fungal secondary metabolites has been discovered to exhibit potent antibacterial activities with distinctive mechanisms and has the potential to be an untapped resource for drug discovery. Here, we describe the isolation and characterization of five new antibacterial indole diketopiperazine alkaloids, namely 24,25-dihydroxyvariecolorin G (**1**), 25-hydroxyrubrumazine B (**2**), 22-chloro-25-hydroxyrubrumazine B (**3**), 25-hydroxyvariecolorin F (**4**), and 27-epi-aspechinulin D (**5**), along with the known analogue neoechinulin B (**6**) from a fungal strain of deep-sea cold seep-derived *Aspergillus chevalieri*. Among these compounds, **3** and **4** represented a class of infrequently occurring fungal chlorinated natural products. Compounds **1**–**6** showed inhibitory activities against several pathogenic bacteria with MIC values ranging from 4 to 32 μg/mL. It was revealed that compound **6** could induce structural damage to the *Aeromonas hydrophila* cells based on the observation by scanning electron microscopy (SEM), which led to the bacteriolysis and death of *A. hydrophila*, suggesting that neoechinulin B (**6**) might be a potential alternative to novel antibiotics development.

## 1. Introduction

A wide range of fungi-derived natural products have been proved to possess significant pharmaceutical applications, such as penicillin derived from *Penicillium* sp. and cephalosporin C derived from *Acremonium chrysogenum*. Therefore, much attention has been paid to fungal secondary metabolites with potent antibacterial activities and novel mechanisms [1,2,3]. Deep-sea cold seeps are special extreme environments characterized by fluid emissions rich in methane and hydrogen sulfide, developing a unique chemosynthetically-driven ecosystem that creates a unique habitat for microorganisms in sediments [4]. Recently, a batch of intriguing findings on secondary metabolites from deep-sea cold seep-derived fungi has highlighted the potential of these microbes as a source of new antibiotics [5,6,7,8,9].

In our continuous research for bioactive metabolites from deep-sea cold seep-derived fungi [5,6,7,8,9], the fungal strain *Aspergillus chevalieri* CS-122, which was isolated from the sulfate-reducing sediments collected at a cold seep in the northeast of the South China Sea, drew our attention. As a result, a couple of indole diketopiperazine alkaloids containing an unprecedented spiro-bicyclo skeleton were isolated and identified [9]. Further work on this fungus has now resulted in the isolation of five other new indole diketopiperazine alkaloids. Among these compounds, compounds **3** and **4** represented a class of infrequently occurring fungal chlorinated natural products. Their structures were determined by detailed analyses of spectroscopic data, NMR calculation with DP4+ probability analysis, and 2,2-dimethoxypropane derivatization. This paper describes the isolation, structure elucidation, and stereochemical assignment of compounds **1**–**5** as well as the antibacterial activities of **1**–**6**.

## 2. Results and Discussion

### 2.1. Structure Elucidation

**Figure 1:**
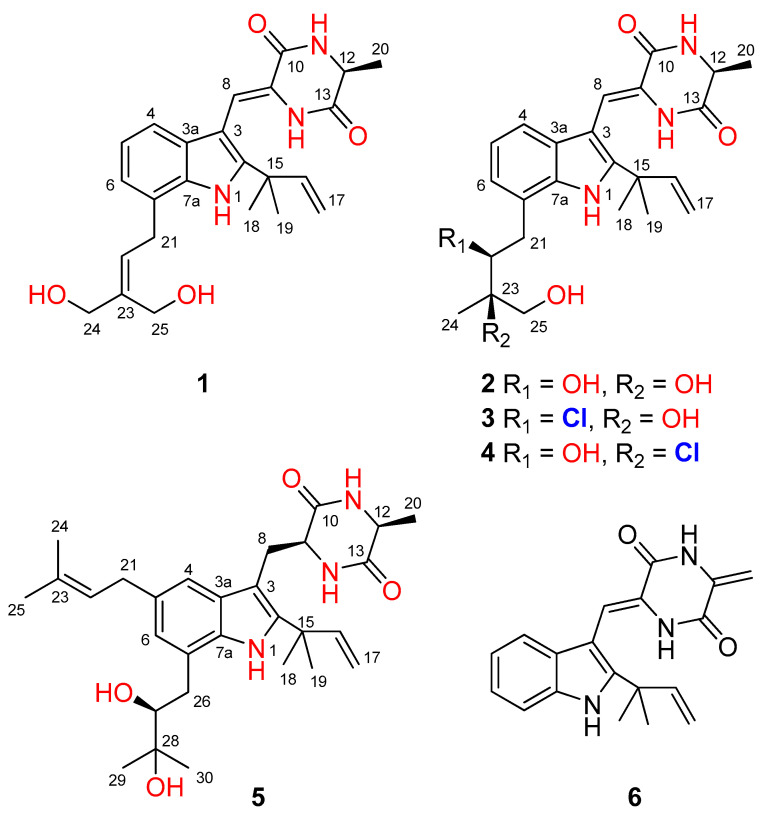
Structures of the isolated compounds **1**–**6**.

The culture broth of the fungus *Aspergillus chevalieri* CS-122 was extracted with EtOAc, and the crude extract was subjected to multiple chromatographic methods (a combination of column chromatography on Si gel, Sephadex LH-20, and Lobar LiChroprep RP-18) to yield subfractions, which were further purified by preparative TLC and semipreparative HPLC to provide the compounds **1**–**6** (Figure 1).

**Figure 2:**
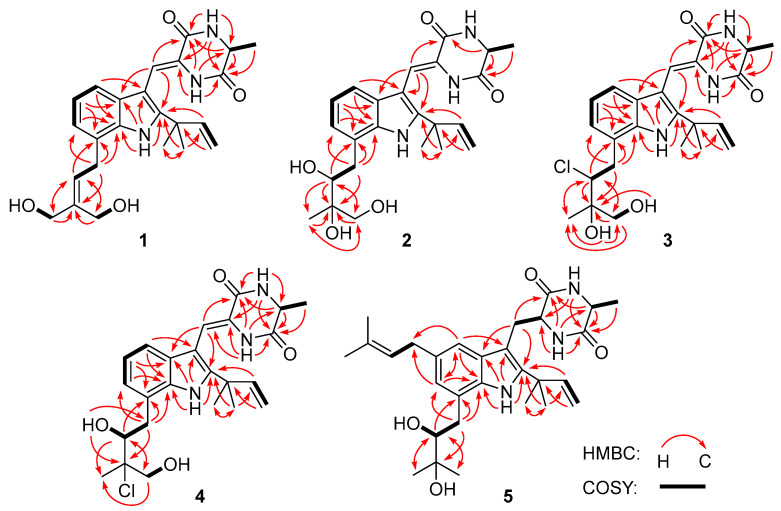
Key ^1^H-^1^H COSY (bold lines) and HMBC (red arrows) correlations of compounds **1**–**5**.

Compound **1** was obtained as a colorless amorphous powder. Its molecular formula was deduced as C_24_H_29_N_3_O_4_ on the basis of positive HRESIMS data, indicating 12 degrees of unsaturation. In the ^1^H NMR spectrum (Table 1), the signals for the *ortho*-coupled aromatic protons (*δ*_H_ 7.04, dd, *J* = 7.4, 1.7 Hz, H-4; *δ*_H_ 6.96, t, *J* = 7.4 Hz, H-5; and *δ*_H_ 6.93, dd, *J* = 7.4, 1.7 Hz, H-6) were indicative of a 1,2,3-trisubstituted benzene ring system. Meanwhile, two trisubstituted olefins (*δ*_H_ 6.88, s, H-8 and *δ*_H_ 5.59, t, *J* = 7.8 Hz, H-22) and a vinyl group (*δ*_H_ 6.10, dd, *J* = 17.6, 10.3 Hz, H-16; *δ*_H_ 5.03, dd, *J* = 17.6, 1.3 Hz, H-17a; *δ*_H_ 5.03, dd, *J* = 10.3, 1.3 Hz, H-17b) were also observed. The ^13^C NMR and DEPT data (Table 2) displayed 24 carbon signals, accounting for three methyls, four methylenes (two oxygenated and one olefinic), seven methines (six aromatic/olefinic), and ten quaternary carbons (two carbonyls and seven aromatic/olefinic) (Table 1). Detailed analysis of the 1D and 2D NMR data revealed that the structure of compound **1** was similar to variecolorin G, a previously reported indole diketopiperazine isolated from the fungal strain *Aspergillus variecolor* B-17 [10]. However, the signals for two methyls (*δ*_H_ 1.75/*δ*_C_ 25.6, CH_3_-24 and *δ*_H_ 1.75/*δ*_C_ 17.8, CH_3_-25) in the NMR spectra of variecolorin G were absent in those of **1**, while resonances for two oxygenated methylenes (*δ*_H_ 3.96/*δ*_C_ 63.5, CH_2_-24 and *δ*_H_ 4.19/*δ*_C_ 57.1, CH_2_-25) were observed in the NMR spectra of **1** (Table 1 and Table 2). The key HMBC correlations from H-24 and H-25 to C-22 and C-23 (Figure 2) further determined the planar structure of **1**.

The geometry of the double bond at C-8 was determined to be *Z*, as the chemical shift of H-8 (*δ*_H_ 6.88) was observed downfield under the influence of the deshielding effect of the C=O group [10,11,12,13]. The absolute configuration of C-12 was assigned by chiral HPLC analysis of the acidic hydrolysate. The retention time of the acidic hydrolysate of **1** matched well with that of _L_-Ala (Figure S1), indicating the *S* configuration of C-12. Thus, the structure of **1** was determined, and it was named 24,25-dihydroxyvariecolorin G.

Compound **2** possessed the molecular formula C_24_H_31_N_3_O_5_ on the basis of HRESIMS data, indicating 11 degrees of unsaturation. Detailed analysis of the ^1^H and ^13^C NMR data of **2** (Table 1 and Table 2) suggested that **2** was an indole diketopiperazine derivative, showing similarity to rubrumazine B, which was characterized from *Eurotium rubrum* MA-150, a fungus isolated from marine mangrove rhizospheric soil [12]. However, the signals for a methyl group (CH_3_-25) resonating at *δ*_H_ 1.18 and *δ*_C_ 26.8 in the NMR spectra of rubrumazine B disappeared in those of **2**. Instead, signals for an oxygenated methylene (CH_2_-25) resonating at *δ*_H_ 3.46/3.40 and *δ*_C_ 67.6 were observed in the NMR spectra of **2** (Table 1 and Table 2). Meanwhile, compared to rubrumazine B, a smaller chemical shift for CH_3_-24 (*δ*_H_ 1.12/*δ*_C_ 19.1) was also observed. The key HMBC correlations from H-25 to C-22, C-23, and C-24 (Figure 2) further constructed the planar structure of **2**, which was designed as the 25-hydroxylated derivative of rubrumazine B.

Compound **3,** isolated as a colorless amorphous powder, was found to have the molecular formula C_24_H_30_ClN_3_O_4_ on the basis of HRESIMS data, suggesting 11 degrees of unsaturation. Specifically, the existence of a chlorine group was deduced by the isotopic peaks at *m/z* 460 and 462 with a ratio of 3:1. Its NMR spectroscopic data were very similar to those of **2**, except for the signal of the oxygenated methine group (CH-22) resonating at *δ*_H_ 3.70/*δ*_C_ 74.8 in **2**, which shifted to *δ*_H_ 4.37/*δ*_C_ 66.9 in those of **3**. The above observation revealed that the 22-OH in compound **2** was replaced by a substituent with less electronegativity in compound **3**. Considering the molecular formula, all the atoms have been assigned except for a remaining chlorine atom, suggesting the substitution of a chlorine group at C-22. Compared to **2**, an obvious larger chemical shift of H-22 was observed, which might be attributed to the steric effect of the chlorine atom. Therefore, the planar structure of **3** was determined as 22-chloro-25-hydroxyrubrumazine B.

The HRESIMS data of compound **4** demonstrated its molecular formula to be C_24_H_30_ClN_3_O_4_, the same as that of compound **3**. Similarly, the isotopic peaks at *m/z* 460 and 462 with a ratio of 3:1 suggested the presence of a chlorine group. The NMR spectra of **4** showed close similarity to those of the chlorine-containing indole diketopiperazine variecolorin F [10]. However, the signals for a methyl (CH_3_-25) at *δ*_H_ 1.63 and *δ*_C_ 29.5 in the NMR spectrum of variecolorin F were replaced by an oxygenated methylene (CH_2_-25) resonating at *δ*_H_ 3.75 and *δ*_C_ 68.1 in those of **4**. This deduction was further supported by the COSY correlations from H-25 to 25-OH and the HMBC correlations from H-25 to C-22 and C-24. The structure of **4** was finally defined as 25-hydroxyvariecolorin F.

**Figure 3:**
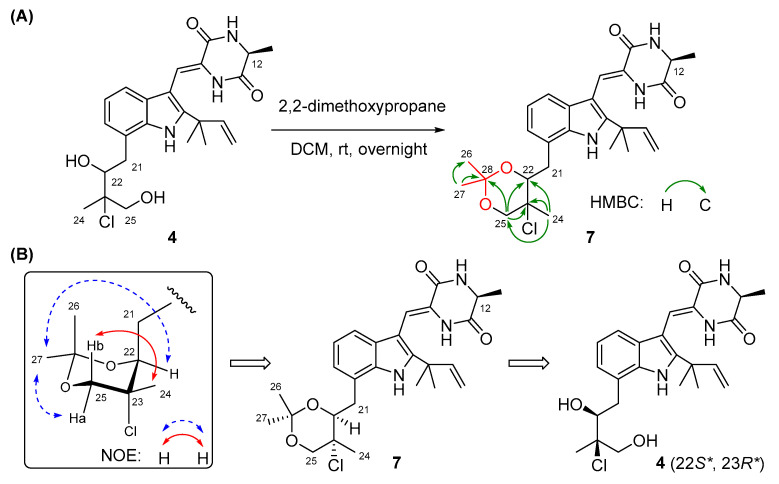
(**A**) The preparation of acetonide **7** and its HMBC correlations. (**B**) NOESY correlations of acetonide **7**.

The configurations of Δ^8^ and C-12 in compounds **2**–**4** were identical to those in **1** based on the same approaches used for **1** (Figures S9, S23, and S37). However, it was extremely difficult to assign the absolute configurations of C-22 and C-23 on the isopentyl moiety of **2**–**4** because of the flexible nature of the saturated side chain and the separation of C-22 and C-23 away from the chiral center C-12, which continued to puzzle the natural product chemists for a long time [14,15]. To determine the absolute configurations of C-22 and C-23 in compounds **2**–**4**, the relative configurations of C-22 and C-23 should be assigned first and then correlated with the absolute configuration of C-12. Quantum chemical calculations of NMR with DP4+ probability analysis is a recently developed and widely used strategy for structural elucidation and configuration assignment [16]. According to the previously reported examples, approximately 20% of compounds with stereoclusters separated through flexible spacers were assigned the configurations with the aid of DP4+ [16,17,18]. Hence, GIAO (gauge-including atomic orbitals) NMR calculations at mPW1PW91/6-31+G(d,p) level with DP4+ probability analyses were performed on compounds **2**–**4**. As a result, the experimental ^1^H and ^13^C NMR data of **2**–**4** matched well with those calculated for the isomers **2c**, **3c**, and **4c** (12S*,22S*,23R*), as indicated by DP4+ probabilities of 99.6% (Table S1), 95.9% (Table S2), and 99.1% (Table S3), respectively. Among them, the relative configuration of compound **4** was further supported by the 2,2-dimethoxypropane derivatization, which produced acetonide **7** (Figure 3A), fixing the rotation of the single bond (C22-C23) through the formation of a six-membered ketal ring. The chemical shifts of two acetal methyl groups at *δ*_C_ 29.0 (C-26) and *δ*_C_ 18.7 (C-27) indicated the chair conformation of the six-membered ketal ring [19]. Subsequently, NOE correlations from H_3_-27 to H-25a and H-22 in **7** suggested the co-face of these groups (Figure 3B), whereas correlations from H_3_-24 to H-25b placed them on the opposite face, and this assignment is consistent with the results obtained by DP4+ probability analysis. Thus, the relative configurations of compounds **2**–**4** were speculated as 12S*, 22S*, and 23R*, and the absolute configurations of compounds **2**–**4** were tentatively speculated as 12*S*, 22*S*, and 23*R* by correlating the stereo configurations of C-22 and C-23 with the absolute configuration of C-12.

To verify the above deduction, many efforts, including a modification of Mosher’s method and the cultivation of single crystals, were made to further assign the configurations of C-22 and C-23 in **2**–**4**. Unfortunately, the presence of multiple -OH groups (three in **2** and two in **3** and **4**) and three -NH groups caused many side reactions during Mosher’s esterification, which resulted in failure to obtain the aim products. Meanwhile, the cultivation of single crystals under various conditions has also not succeeded. Hence, continuous investigations on the absolute configurations of **2**–**4** are still required in our future work, which might involve the continuous cultivation of high-quality crystals and applying alternative protocols for NMR calculations (including different methods for conformational sampling and other functionals and bases for calculations) or performing RSCA and RDC experiments. Once the configurations of **2**–**4** are unambiguously assigned, we will report the findings in due course.

Compound **5** had a molecular formula of C_29_H_41_N_3_O_4_ as determined by HRESIMS analysis. The ^1^H and ^13^C NMR data revealed that **5** is an indole diketopiperazine alkaloid, similar to previously reported aspechinulin D from the deep-sea-derived fungus *Aspergillus* sp. FS445 [20]. Compared to the NMR data of aspechinulin D (*δ*_C_ 75.8, C-28; *δ*_H_/*δ*_C_ 1.62/27.6, CH_3_-29; *δ*_H_/*δ*_C_ 1.62/30.0, CH_3_-30, measured in DMSO-*d*_6_), obvious upfielded shifts for the oxygenated quaternary carbon (C-28) and two methyl groups (CH_3_-29 and CH_3_-30) in the NMR spectra of **5** were observed (*δ*_C_ 72.2, C-28; *δ*_H_ 1.16/*δ*_C_ 23.6, CH_3_-29; *δ*_H_ 1.17/*δ*_C_ 27.0, CH_3_-30), suggesting that **5** is a diastereomer of aspechinulin D, epimeric at C-27. Thus, compound **5** was named 27-epi-aspechinulin D.

### 2.2. Antimicrobial Activity

All the isolated compounds were examined to assess their antimicrobial activity against several human pathogenic and aqua-pathogenic bacteria (Table 3). Compounds **1**–**5** showed moderate activity against the human pathogen *Escherichia coli* and the aquatic bacterium *Vibrio harveyi* (MIC ≤ 32 μg/mL). Among them, compound **1** displayed significant inhibitory effects against *E. coli*, with a MIC value of 4 μg/mL, while compound **3** displayed noticeable inhibitory effects against *V. harveyi*, with a MIC value of 8 μg/mL. Moreover, compounds **2** (with hydroxyl groups at C-22 and C-23) and **5** (with hydroxyl groups at C-27 and C-28) exhibited broad-spectrum antibacterial activity against five tested bacterial strains (MIC ≤ 32 μg/mL), implying that the bishyroxylation at these positions made them broad-spectrum.

**Figure 4:**
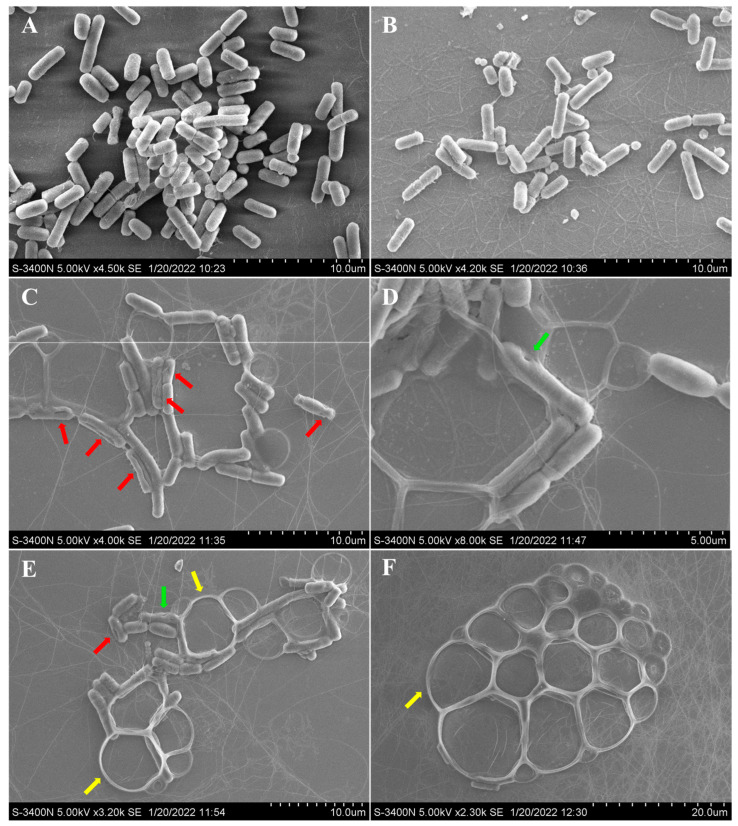
SEM observations of morphological changes of *Aeromonas hydrophila* following treatment with neoechunulin B (**6**). *A. hydrophila* cell morphology without any treatment (**A**), with DMSO treatment (**B**), and with compound **6** treatment (**C**–**F**). The red, green, and yellow arrows indicate the grooves, pores, and bubbles, respectively.

Additionally, compound **6** showed significant activity against aquatic *Aeromonas hydrophila*, with a MIC value of 4 μg/mL. The possible antibacterial mechanism of **6** was studied based on the observation by scanning electron microscopy (SEM) for the cells before and after treatment with compound **6**. For the *A. hydrophila* cells untreated with **6** (Figure 4A) or treated only with DMSO (Figure 4B), it was observed that the cell surfaces were smooth and the cell structures were plump and intact. However, for the *A. hydrophila* cells treated with compound **6**, conglutination of the cells with a viscous substance attached was observed, while deep grooves (red arrow) and obvious pores (green arrow) on the surfaces of the cells were also observed (Figure 4C,D). Meanwhile, serious cell deformation and severe damage to cell membranes also appeared on a number of cells, which were accompanied by bacteriolysis and even complete disappearance, which resulted in large bubbles (Figure 4E,F, yellow arrow). The above observations revealed that compound **6** could induce structural damage to the *A. hydrophila* cells, which led to the bacteriolysis and death of *A. hydrophila*.

## 3. Experimental Section

### 3.1. General Experimental Procedures

Detailed information for the apparatus, reagents, solvents, and materials used in the present work is the same as that described in our previous publication [9].

### 3.2. Fungal Material

The fungus *Aspergillus chevalieri* CS-122 was isolated from the deep-sea cold seep sediment, which was collected in the northeast of the South China Sea (119°17′ E, 22°06′ N) in August 2018. The fungal strain was identified as *A. chevalieri* according to the *β*-tubulin gene sequence [21], which is the same (100%) as that of *A. chevalieri* (accession No. KU872171.1). The sequence data of CS-122 were deposited in GenBank with accession No. OM304365.1 (https://www.ncbi.nlm.nih.gov/nuccore/OM304356.1, accessed on 22 March 2023). This strain is stored at the Key Laboratory of Experimental Marine Biology, Institute of Oceanology, Chinese Academy of Sciences (IOCAS).

### 3.3. Fermentation, Extraction, and Isolation

The fungal strain *Aspergillus chevalieri* CS-122 was cultivated on potato dextrose agar medium at 28 °C for 7 days. Next, it was transferred into 1 L Erlenmeyer flasks containing rice solid medium (each flask contained 70 g rice, 0.3 g peptone from animal tissue, 0.5 g yeast extract, 0.2 g corn steep liquor, 0.1 g monosodium glutamate, and naturally sourced seawater) and incubated at room temperature for 30 days. Then, the solid fermented substrate was extracted three times with EtOAc. The combined extracts were concentrated under reduced pressure to provide a dark brown crude extract (256 g).

The total extract (256 g) was subjected to VLC (vacuum liquid chromatography) eluting with a gradient of petroleum ether (PE)/EtOAc (from 20:1 to 1:1) and CH_2_Cl_2_/MeOH (from 50:1 to 1:1) to yield ten fractions (Frs. 1–10). Fr. 8 (10.3 g), eluted with CH_2_Cl_2_/MeOH (10:1), was further purified by reversed-phase column chromatography over RP-18 with a MeOH/H_2_O gradient (from 10:90 to 90:10) to afford nine subfractions (Frs. 8.1–8.9). Fr. 8.4 (2.1 g) was further purified by CC on silica gel eluting with a CH_2_Cl_2_/MeOH gradient (from 150:1 to 50:1) and then by preparative TLC as well as Sephadex LH-20 (MeOH) to yield **1** (8.6 mg) and **2** (4.9 mg). Fr. 8.5 (1.6 g) was split by CC on silica gel eluting with a CH_2_Cl_2_/MeOH gradient (from 150:1 to 30:1) to afford five subfractions (Frs. 8.5.1–8.5.5). Fr. 8.5.2 (57.7 mg) was further purified by semipreparative HPLC (42% MeCN−H_2_O, 12 mL/min, 254 nm) to provide **4** (5.8 mg), while Fr. 8.5.5 (62.8 mg) was also purified by semipreparative HPLC (77% MeOH−H_2_O, 10 mL/min, 254 nm) to provide **5** (6.2 mg). Fr. 8.6 (2.4 g) was fractionated by CC on silica gel eluting with a CH_2_Cl_2_/MeOH gradient (from 150:1 to 50:1) and then purified by semipreparative HPLC (80% MeOH−H_2_O, 10 mL/min, 254 nm) to obtain **3** (5.6 mg). The isolation of compound **6** was described in our previous publication [9].

*24,25-Dihydroxyvariecolorin G* (**1**): colorless amorphous powder; [α]25 D –17.4 (*c* =0.23, MeOH); UV (MeOH) *λ_max_* (log *ε*) 226 (3.26) nm, 252 (3.11) nm, 279 (2.90) nm, 335 (2.96) nm; ECD (0.35 mM, MeOH) *λ_max_* (Δ*ε*) 214 (–5.52), 240 (+2.22), 342 (–1.46) nm; ^1^H and ^13^C NMR data, Table 1 and Table 2; HRESIMS *m/z* 424.2225 [M+H]^+^ (calcd for C_24_H_30_N_3_O_4_, 424.2231).
*25-Hydroxyrubrumazine B* (**2**): colorless amorphous powder; [α]25 D –23.8 (*c* =0.21, MeOH); UV (MeOH) *λ_max_* (log *ε*) 225 (3.66) nm, 255 (3.28) nm, 282 (3.13) nm, 339 (3.22) nm; ECD (0.57 mM, MeOH) *λ_max_* (Δ*ε*) 221 (–11.88), 248 (+0.52), 333 (–1.97) nm; ^1^H and ^13^C NMR data, Table 1 and Table 2; HRESIMS *m/z* 442.2330 [M+H]^+^ (calcd for C_24_H_32_N_3_O_5_, 442.2336).
*22-Chloro-25-hydroxyrubrumazine B* (**3**): colorless amorphous powder; [α]25 D –25.0 (*c* =0.16, MeOH); UV (MeOH) *λ_max_* (log *ε*) 224 (3.56) nm, 257 (3.15) nm, 278 (2.98) nm, 335 (3.06) nm; ECD (0.35 mM, MeOH) *λ_max_* (Δ*ε*) 206 (–9.54), 234 (+7.56), 328 (–3.46) nm; ^1^H and ^13^C NMR data, Table 1 and Table 2; HRESIMS *m/z* 460.1987 [M+H]^+^ (calcd for C_24_H_30_ClN_3_O_4_, 460.1998).
*25-Hydroxyvariecolorin F* (**4**): colorless amorphous powder; [α]25 D –53.8 (*c* =0.26, MeOH); UV (MeOH) *λ_max_* (log *ε*) 226 (3.70) nm, 255 (3.30) nm, 279 (3.13) nm, 334 (3.22) nm; ECD (0.28 mM, MeOH) *λ_max_* (Δ*ε*) 204 (–12.69), 240 (+2.38), 334 (–2.71) nm; ^1^H and ^13^C NMR data, Table 1 and Table 2; HRESIMS *m/z* 460.1992 [M+H]^+^ (calcd for C_24_H_30_ClN_3_O_4_, 460.1998).
*27-epi-Aspechinulin D* (**5**): colorless amorphous powder; [α]25 D –23.1 (*c* =0.13, MeOH); UV (MeOH) *λ_max_* (log *ε*) 231 (3.61) nm, 280 (2.99) nm; ECD (0.26 mM, MeOH) *λ_max_* (Δ*ε*) 226 (–9.63), 271 (+1.03) nm; ^1^H and ^13^C NMR data, Table 1 and Table 2; HRESIMS m/z 496.3157 [M+H]^+^ (calcd for C_29_H_42_N_3_O_4_, 496.3170).

### 3.4. Computational NMR Chemical Shift Calculation and DP4+ Analysis

All the theoretical calculations were conducted in the Gaussian09 program package. Conformational searches for possible isomers based on molecular mechanics with the MMFF method were performed using HyperChem 8.0 software. NMR shielding tensors were calculated using the GIAO method. The corresponding stable conformers whose Boltzmann distributions were higher than 2%, were further optimized at B3LYP/6-31G(d) in vacuo. Then, all the optimized conformers were subjected to the DFT method at mPW1PW91\6-31+G(d) with PCM level in DMSO to acquire the calculated shielding tensors. The calculated shielding tensors were later obtained according to the Boltzmann weighting of each conformer. Finally, the DP4+ analysis of the calculated shielding tensors and experimental chemical shifts were applied with the Excel formulas provided by the original authors [22].

### 3.5. Acidic Hydrolysis of Compounds ***1***–***5***

Compounds **1–5** (1 mg each) were dissolved in 10 mL of 6 N HCl and heated in a sealed tube at 110 °C for 24 h [12]. The solutions were then evaporated to dryness under reduced pressure. Each sample, including the standard amino acids _L_-Ala and _D_-Ala, was dissolved in 1 mL of eluting solvent (2 mM CuSO_4_·5H_2_O in 100 mL of H_2_O). Chiral HPLC analysis, both alone and by co-injection with standards, was carried out using a Phenomenex-Chirex-3126 column (250 mm × 4.60 mm, 5 μm; flow rate 1.0 mL/min at 40 °C; detection at 254 nm).

### 3.6. 2,2-Dimethoxypropane Derivatization of Compound ***4***

Compound **4** (2 mg) was dissolved in 2 mL CH_2_Cl_2_. Next, 50 μL 2,2-dimethoxypropane and 10 mg Amberlyst-15H were added to the solution. The reaction mixture was stirred at room temperature overnight and then filtered, and the organic layer was evaporated at reduced pressure [23]. Further purification by preparative TLC afforded acetonide **7** (0.5 mg).

Acetonide **7**: colorless amorphous powder; Diagnostic ^1^H NMR data (DMSO-*d*_6_, 500 MHz) *δ*_H_ 4.41 (1H, br d, *J* = 9.6 Hz, H-22), 4.06 (1H, d, *J* = 11.3 Hz, H-25a), 3.76 (1H, d, *J* = 11.3 Hz, H-25b), 3.08 (1H, dd, *J* = 16.1, 9.6 Hz, H-21b), 1.78 (3H, s, H-24), 1.52 (6H, s, H-18/H-19), 1.43 (3H, s, H-27), 1.37 (3H, d, *J* = 6.9 Hz, H-20), 1.31 (3H, s, H-26); Diagnostic ^13^C NMR data (DMSO-*d*_6_, 125 MHz) *δ*_C_ 99.4 (C, C-28), 76.3 (CH, C-22), 70.5 (CH_2_, C-25), 65.7 (C, C-23), 29.0 (CH_3_, C-26), 27.5 (CH_3_, C-18/C-19), 20.6 (CH_3_, C-24), 19.6 (CH_3_, C-20), 18.7 (CH_3_, C-27); Key HMBC correlations from H_2_-25b to C-28, from H_3_-26 to C-27 and C-28, from H_3_-27 to C-26 and C-28.

### 3.7. Antibacterial Assay

The antibacterial activities against human pathogenic bacteria (*Escherichia coli* and *Micrococcus luteus*) and aquatic pathogens (*Vibrio harveyi*, *Edwardsiella tarda*, *V. anguillarum*, and *Aeromonas hydrophilia*) were determined by a serial dilution technique using 96-well microtiter plates with minor modifications, as described in our previous report [5,6,7,8,9]. The bacteria were cultivated overnight at 37 °C in liquid LB medium and diluted with the LB broth to a concentration of 1.5 × 10 ^8^ CFU/mL. The tested compounds and positive control (chloramphenicol) were dissolved in DMSO to provide a stock solution. Then, 5 μL of the sample solutions with different concentrations, together with 95 μL of the bacterial suspension, were added to the 96-well plates and incubated at 37 °C for 12 h. The growth situation of the bacteria was measured by a multi-detection microplate reader (Infinite M1000 Pro, Tecan) at 600 nm. The human or aquatic pathogenic strains were offered by the Institute of Oceanology, Chinese Academy of Sciences.

### 3.8. Scanning Electron Microscopy (SEM)

The effect of neoechinulin B (**6**) on the morphological changes of *Aeromonas hydrophilia* was examined by scanning electron microscopy (SEM) [24]. Briefly, *Aeromonas hydrophilia* was incubated at 28 °C in LB broth at 140 rpm, with shaking for 12 h, to an OD_600_ of 0.2. Then, the bacteria were cultivated for an additional 12 h with 64 µg/mL neoechinulin B or DMSO. After cultivation, the culture broth was centrifuged and then washed with PBS (pH 7.2–7.4) three times. The collected bacteria were further fixed with 5% glutaraldehyde solution, washed twice with PBS, and then dehydrated for 15 min through a graded ethanol series (30%, 50%, 70%, 80%, 90% and 100%). The dried bacterial cells were transferred to isopentyl acetate for 20 min. The samples were gold coated and visualized under the scanning electron microscope (Hitachi, S-3400N).

## 4. Conclusions

In this study, we isolated and characterized five new compounds (**1**–**5**) from the cold seep sediment-derived fungus *Aspergillus chevalieri* CS-122, which are new members of the indole diketopiperazine alkaloids. Among them, compounds **3** and **4** were a kind of infrequently occurring fungal chlorinated natural products. Compounds **1**–**6** showed potent antibacterial activities against several aquatic pathogens. Among them, compound **6** significantly destroyed the cell morphology of *A. hydrophila* to exert a growth-inhibitory effect, the specific mechanism and target of which must be further investigated. These compounds possess the potential to be developed as antibiotic lead compounds for aquaculture.

## Figures and Tables

**Table 1:** ^1^H NMR Spectroscopic Data for Compounds **1**–**5** (*δ* in ppm, *J* in Hz) ^a^.

No.	1	2	3	4	5
1-NH	10.58, s	10.46, s	10.13, s	10.10, s	9.89, s
4	7.04, dd, (7.4, 1.7)	7.05, m	7.09, m	7.08, dd, (7.3, 1.7)	7.07, s
5	6.96, t, (7.4)	6.94, overlap	6.99, overlap	6.98, overlap	
6	6.93, dd, (7.4, 1.7)	6.94, overlap	7.00, overlap	7.00, overlap	6.71, s
8	6.88, s	6.90, s	6.88, s	6.89, s	Ha, 3.01, dd, (14.5, 9.6) Hb, 3.34, m
9					3.96, m
11-NH	8.31, s	8.31, s	8.33, s	8.34, s	8.18, s
12	4.15, qd, (6.9, 1.6)	4.16, q, (6.9)	4.14, qd, (6.9, 1.4)	4.17, qd, (6.9, 1.9)	3.82, qd, (6.9, 2.3)
14-NH	8.62, s	8.56, s	8.74, s	8.57, s	7.41, s
16	6.10, dd, (17.6, 10.3)	6.09, dd, (17.3, 10.6)	6.11, dd, (17.2, 10.6)	6.10, dd, (17.3, 10.6)	6.16, dd, (17.4, 10.6)
17	Ha, 5.03, dd, (17.6, 1.3) Hb, 5.03, dd, (10.3, 1.3)	Ha, 5.05, dd, (17.3, 1.2) Hb, 5.06, dd, (10.6, 1.2)	Ha, 5.04, d, (17.2) Hb, 5.05, d, (10.6)	Ha, 5.06, dd, (17.3, 1.2) Hb, 5.07, dd, (10.6, 1.2)	Ha, 5.08, d, (17.4) Hb, 5.04, d, (10.6)
18	1.48, s	1.49, s	1.50, s	1.50, s	1.48, s
19	1.48, s	1.49, s	1.50, s	1.50, s	1.49, s
20	1.37, d, (6.9)	1.37, d, (6.9)	1.39, d, (6.9)	1.38, d, (6.9)	1.33, d, (6.9)
21	3.76, d, (7.8)	Ha, 2.76, dd, (14.6, 8.8) Hb, 3.25, d, (14.6)	Ha, 3.15, dd, (15.5, 10.6) Hb, 3.67, d, (15.5)	Ha, 2.93, dd, (14.6, 8.8) Hb, 3.31, d, (14.6)	3.31, d, (7.2)
22	5.59, t, (7.8)	3.70, d, (8.8)	4.37, d, (10.6)	3.97, t, (8.8)	5.32, t, (7.2)
22-OH				5.31, d, (7.2)	
23-OH			5.14, s		
24	3.96, d, (4.2)	1.12, s	1.28, s	1.56, s	1.71, s
24-OH	4.70, t, (4.2)				
25	4.19, d, (3.8)	Ha, 3.46, d, (10.9) Hb, 3.40, d, (10.9)	3.57, m	3.75, m	1.70, s
25-OH	5.32, t, (3.8)		5.26, t, (5.5)	5.54, t, (6.1)	
26					Ha, 2.65, dd, (14.5, 8.7) Hb, 3,13, d, (14.5)
27					3.42, m
27-OH					4.99, d, (5.7)
28-OH					4.69, s
29					1.16, s
30					1.17, s

^a^ Measured at 500 MHz in DMSO-*d*_6_.

**Table 2:** ^13^C NMR Spectroscopic Data for Compounds **1**–**5** (*δ* in ppm, type) ^a^.

No.	1	2	3	4	5
2	143.8, C	143.5, C	143.6, C	143.5, C	141.0, C
3	103.9, C	103.8, C	104.2, C	104.0, C	104.9, C
3a	126.0, C	125.9, C	126.1, C	126.0, C	129.1, C
4	117.0, CH	116.8, CH	117.3, CH	117.0, CH	114.8, CH
5	121.1, CH	119.6, CH	119.5, CH	119.7, CH	131.4, C
6	119.7, CH	121.7, CH	121.3, CH	121.9, CH	122.2, CH
7	123.7, C	125.2, C	122.6, C	123.8, C	124.1, C
7a	133.9, C	134.3, C	133.9, C	134.2, C	132.7, C
8	110.3, CH	110.2, CH	110.1, CH	110.0, CH	31.3, CH_2_
9	125.1, C	124.8, C	125.2, C	125.1, C	55.6, CH
10	159.9, C	159.9, C	159.8, C	159.8, C	167.4, C
12	50.5, CH	50.5, CH	50.6, CH	50.5, CH	50.3, CH
13	166.4, C	166.3, C	166.4, C	166.4, C	167.9, C
15	39.1, C	38.9, C	39.0, C	38.9, C	38.7, C
16	145.4, CH	145.1, CH	145.2, CH	145.1, CH	146.5, CH
17	111.4, CH_2_	111.7, CH_2_	111.7, CH_2_	111.8, CH_2_	111.1, CH2
18	27.6, CH_3_	27.4, CH_3_	27.6, CH_3_	27.5, CH_3_	27.9, CH_3_
19	27.6, CH_3_	27.4, CH_3_	27.5, CH_3_	27.4, CH_3_	27.9, CH_3_
20	19.6, CH_3_	19.6, CH_3_	19.8, CH_3_	19.6, CH_3_	20.7, CH_3_
21	29.6, CH_2_	34.0, CH_2_	33.8, CH_2_	34.2, CH_2_	34.2, CH_2_
22	124.7, CH	74.8, CH	66.9, CH	77.7, CH	124.9, CH
23	139.2, C	74.2, C	74.2, C	74.0, C	130.2, C
24	63.5, CH_2_	19.1, CH_3_	21.0, CH_3_	22.8, CH_3_	17.7, CH_3_
25	57.1, CH_2_	67.6, CH_2_	67.5, CH_2_	68.1, CH_2_	25.6, CH_3_
26					34.5, CH_2_
27					79.0, CH
28					72.2, C
29					23.6, CH_3_
30					27.0, CH_3_

^a^ Measured at 125 MHz in DMSO-*d*_6_.

**Table 3:** Antimicrobial Activities of Compounds **1–6** (MIC, μg/mL).

Strain	Compound						
	1	2	3	4	5	6	Chloramphenicol ^b^
*Vibrio harveyi*	16	32	8	32	16	-	2
*Edwardsiella tarda*	-^a^	16	-	-	32	-	8
*Aeromonas hydrophila*	-	32	-	-	32	4	2
*Escherichia coli*	4	16	32	32	32	8	2
*Micrococcus luteus*	-	32	-	-	16	-	2

^a^ (–) = MIC > 32 μg/mL. ^b^ Chloramphenicol as positive control.

## Supplementary Materials

The following are available online at https://www.mdpi.com/article/10.3390/md21030195/s1. Figures S1–S69: The chiral HPLC analysis of the acidic hydrolysate, the 1D and 2D NMR spectra, HRESIMS, the ECDs of compounds **1**–**5**, the 1D and 2D NMR spectra of acetonide 7, and the optimized conformers for the candidate isomers and the results of DP4+ probability analysis of compounds **2**–**4**; Tables S1–S27: The DP4+ probability analysis, calculated shielding tensors of each conformer for the candidate isomers, and cartesian coordinates of the lowest energy conformers for the candidate isomers of compounds **2**–**4.**
