# Supplementary material for: Antibacterial Indole Diketopiperazine Alkaloids from the Deep-Sea Cold Seep-Derived Fungus Aspergillus chevalieri
Source: Mar Drugs. 2023 Mar 22;21(3):195. doi: 10.3390/md21030195 (PMC10059655; doi:10.3390/md21030195)
Supplement: Supplementary file 1 [file marinedrugs-21-00195-s001.zip › marinedrugs-2206988-supplementary.pdf]

Supplementary Materials

Antibacterial indole diketopiperazine alkaloids from the Deep-sea Cold Seep-Derived Fungus *Aspergillus chevalieri*

Li-Hong Yan [1,2,3], Feng-Yu Du [5], Xiao-Ming Li [1,2,4], Sui-Qun Yang [1,2,4], Bin-Gui Wang [1,2,3,4,*] and Xin Li [1,2,4,*]

[1] CAS and Shandong Province Key Laboratory of Experimental Marine Biology, Institute of Oceanology, Chinese Academy of Sciences, Nanhai Road 7, Qingdao 266071, China

[2] Laboratory of Marine Biology and Biotechnology, Qingdao National Laboratory for Marine Science and Technology, Wenhai Road 1, Qingdao 266237, China

[3] University of Chinese Academy of Sciences, Yuquan Road 19A, Beijing 100049, China

[4] Center for Ocean Mega-Science, Chinese Academy of Sciences, Nanhai Road 7, Qingdao 266071, China

[5] College of Chemistry and Pharmacy, Qingdao Agricultural University, Changcheng Road 700, Qingdao 266109, China

Content

Figure S1. Chiral HPLC analysis of the acidic hydrolysate of compound **1**;

Figure S2. ^{1}H NMR (500 MHz, DMSO-d_6) spectrum of compound **1**;

Figure S3. ^{13}C NMR (125 MHz, DMSO-d_6) and DEPT spectra of compound **1**;

Figure S4. COSY spectrum of compound **1**;

Figure S5. HSQC spectrum of compound **1**;

Figure S6. HMBC spectrum of compound **1**;

Figure S7. HR-ESI-MS spectrum of compound **1**;

Figure S8. ECD spectrum of compound **1**;

Figure S9. Chiral HPLC analysis of the acidic hydrolysate of compound **2**;

Figure S10. ^{1}H NMR (500 MHz, DMSO-d_6) spectrum of compound **2**;

Figure S11. ^{13}C NMR (125 MHz, DMSO-d_6) and DEPT spectra of compound **2**;

Figure S12. COSY spectrum of compound **2**;

Figure S13. HSQC spectrum of compound **2**;

Figure S14. HMBC spectrum of compound **2**;

Figure S15. NOESY spectrum of compound **2**;

Figure S16. HR-ESI-MS spectrum of compound **2**;

Figure S17. ECD spectrum of compound **2**;

Figure S18. Structures of four possible isomers for DP4+ probability analysis of compound **2**;

Figure S19. Optimized geometries of predominant conformers (weighting factors) for isomer **2a** at the B3LYP/6-31+g(d,p) level. A total of 152 conformers were searched and 8 of them possess Boltzmann distributions over 2%.

Figure S20. Optimized geometries of predominant conformers (weighting factors) for isomer **2b** at the B3LYP/6-31+g(d,p) level. A total of 132 conformers were searched and 7 of them possess Boltzmann distributions over 2%.

Figure S21. Optimized geometries of predominant conformers (weighting factors) for isomer **2c** at the B3LYP/6-31+g(d,p) level. A total of 178 conformers were searched and 8 of them possess Boltzmann distributions over 2%.

Figure S22. Optimized geometries of predominant conformers (weighting factors) for isomer **2d** at the B3LYP/6-31+g(d,p) level. A total of 177 conformers were searched and 7 of them possess Boltzmann distributions over 2%.

Figure S23. Chiral HPLC analysis of the acidic hydrolysate of compound **3**;

Figure S24. ^{1}H NMR (500 MHz, DMSO-d_6) spectrum of compound **3**;

Figure S25. ^{13}C NMR (125 MHz, DMSO-d_6) and DEPT spectra of compound **3**;

Figure S26. COSY spectrum of compound **3**;

Figure S27. HSQC spectrum of compound **3**;

Figure S28. HMBC spectrum of compound **3**;

Figure S29. NOESY spectrum of compound **3**;

Figure S30. HR-ESI-MS spectrum of compound **3**;

Figure S31. ECD spectrum of compound **3**;

Figure S32. Structures of four possible isomers for DP4+ probability analysis of compound **3**;

Figure S33. Optimized geometries of predominant conformers (weighting factors) for isomer **3a** at the B3LYP/6-31+g(d,p) level. A total of 155 conformers were searched and 6 of them possess Boltzmann distributions over 2%.

Figure S34. Optimized geometries of predominant conformers (weighting factors) for isomer **3b** at the B3LYP/6-31+g(d,p) level. A total of 168 conformers were searched and 8 of them possess Boltzmann distributions over 2%.

Figure S35. Optimized geometries of predominant conformers (weighting factors) for isomer **3c** at the B3LYP/6-31+g(d,p) level. A total of 176 conformers were searched and 8 of them possess Boltzmann distributions over 2%.

Figure S36. Optimized geometries of predominant conformers (weighting factors) for isomer **3d** at the B3LYP/6-31+g(d,p) level. A total of 146 conformers were searched and 8 of them possess Boltzmann distributions over 2%.

Figure S37. Chiral HPLC analysis of the acidic hydrolysate of compound **4**;

Figure S38. ^{1}H NMR (500 MHz, DMSO-d_6) spectrum of compound **4**;

Figure S39. ^{13}C NMR (125 MHz, DMSO-d_6) and DEPT spectra of compound **4**;

Figure S40. COSY spectrum of compound **4**;

Figure S41. HSQC spectrum of compound **4**;

Figure S42. HMBC spectrum of compound **4**;

Figure S43. NOESY spectrum of compound **4;**

Figure S44. HR-ESI-MS spectrum of compound **4**;

Figure S45. ECD spectrum of compound **4**;

Figure S46. Structures of four possible isomers for DP4+ probability analysis of compound **4**;

Figure S47. Optimized geometries of predominant conformers (weighting factors) for isomer **4a** at the B3LYP/6-31+g(d,p) level. A total of 270 conformers were searched and 7 of them possess Boltzmann distributions over 2%.

Figure S48. Optimized geometries of predominant conformers (weighting factors) for isomer **4b** at the B3LYP/6-31+g(d,p) level. A total of 349 conformers were searched and 4 of them possess Boltzmann distributions over 2%.

Figure S49. Optimized geometries of predominant conformers (weighting factors) for isomer **4c** at the B3LYP/6-31+g(d,p) level. A total of 327 conformers were searched and 6 of them possess Boltzmann distributions over 2%.

Figure S50. Optimized geometries of predominant conformers (weighting factors) for isomer **4d** at the B3LYP/6-31+g(d,p) level. A total of 281 conformers were searched and 6 of them possess

Boltzmann distributions over 2%.

Figure S51. Chiral HPLC analysis of the acidic hydrolysate of compound **5**;

Figure S52. ^{1}H NMR (500 MHz, DMSO-d_6) spectrum of compound **5**;

Figure S53. ^{13}C NMR (125 MHz, DMSO-d_6) and DEPT spectra of compound **5**;

Figure S54. COSY spectrum of compound **5**;

Figure S55. HSQC spectrum of compound **5**;

Figure S56. HMBC spectrum of compound **5**;

Figure S57. NOESY spectrum of compound **5**;

Figure S58. HR-ESI-MS spectrum of compound **5**;

Figure S59. ECD spectrum of compound **5**;

Figure S60. ^{1}H NMR (500 MHz, DMSO-d_6) spectrum (δ_H range from 0.8 to 2.0 ppm) of compound **7** compared with **4**;

Figure S61. ^{1}H NMR (500 MHz, DMSO-d_6) spectrum (δ_H range from 2.5 to 4.5 ppm) of compound **7** compared with **4**;

Figure S62. ^{13}C NMR (125 MHz, DMSO-d_6) spectrum (δ_C range from 15.0 to 33.0 ppm) of compound **7** compared with **4**;

Figure S63. Enlarged HMBC spectrum of compound **7**;

Figure S64. Enlarged NOESY spectrum of compound **7**;

Figure S65. ^{1}H NMR (500 MHz, DMSO-d_6) spectrum of compound **7**;

Figure S66. ^{13}C NMR (125 MHz, DMSO-d_6) spectrum of compound **7**;

Figure S67. HSQC spectrum of compound **7**;

Figure S68. HMBC spectrum of compound **7**;

Figure S69. NOESY spectrum of compound **7**;

Table S1. DP4+ probability analysis of compound **2** (mPW1PW91/6-31+G(d, p) level);

Table S2. DP4+ probability analysis of compound **3** (mPW1PW91/6-31+G(d, p) level);

Table S3. DP4+ probability analysis of compound **4** (mPW1PW91/6-31+G(d, p) level);

Table S4. The calculated shielding tensors of each conformer (>2%) for isomer **2a**;

Table S5. The calculated shielding tensors of each conformer (>2%) for isomer **2b**;

Table S6. The calculated shielding tensors of each conformer (>2%) for isomer **2c**;

Table S7. The calculated shielding tensors of each conformer (>2%) for isomer **2d**;

Table S8. The calculated shielding tensors of each conformer (>2%) for isomer **3a**;

Table S9. The calculated shielding tensors of each conformer (>2%) for isomer **3b**;

Table S10. The calculated shielding tensors of each conformer (>2%) for isomer **3c**;

Table S11. The calculated shielding tensors of each conformer (>2%) for isomer **3d**;

Table S12. The calculated shielding tensors of each conformer (>2%) for isomer **4a**;

Table S13. The calculated shielding tensors of each conformer (>2%) for isomer **4b**;

Table S14. The calculated shielding tensors of each conformer (>2%) for isomer **4c**;

Table S15. The calculated shielding tensors of each conformer (>2%) for isomer **4d**;

Table S17. The Cartesian coordinates of the lowest energy conformers (Conf. **1**-**8**) for isomer **2a**;

Table S17. The Cartesian coordinates of the lowest energy conformers (Conf. **1**-**7**) for isomer **2b**;

Table S18. The Cartesian coordinates of the lowest energy conformers (Conf. **1**-**8**) for isomer **2c**;

Table S19. The Cartesian coordinates of the lowest energy conformers (Conf. **1**-**7)** for isomer **2d**;

Table S20. The Cartesian coordinates of the lowest energy conformers (Conf. **1**-**6**) for isomer **3a**;

Table S21. The Cartesian coordinates of the lowest energy conformers (Conf. **1**-**8**) for isomer **3b**;

Table S22. The Cartesian coordinates of the lowest energy conformers (Conf. **1**-**8**) for isomer **3c**;

Table S23. The Cartesian coordinates of the lowest energy conformers (Conf. **1**-**8**) for isomer **3d**;

Table S24. The Cartesian coordinates of the lowest energy conformers (Conf. **1**-**7**) for isomer **4a**;

Table S25. The Cartesian coordinates of the lowest energy conformers (Conf. **1**-**4**) for isomer **4b**;

Table S26. The Cartesian coordinates of the lowest energy conformers (Conf. **1**-**6**) for isomer **4c**;

Table S27. The Cartesian coordinates of the lowest energy conformers (Conf. **1**-**6**) for isomer **4d**.

Figure S1. Chiral HPLC analysis of the acidic hydrolysate of compound **1**.

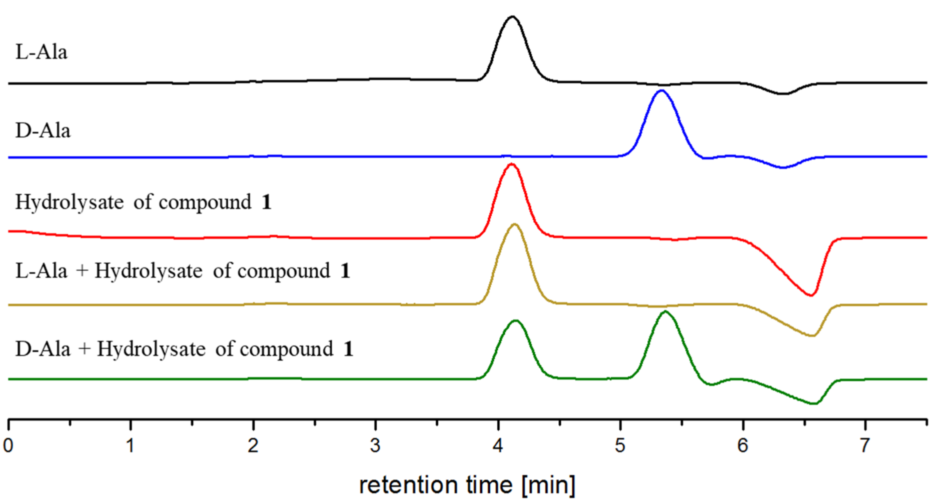

Figure S2. ^{1}H NMR (500 MHz, DMSO-d_6) spectrum of compound **1**.

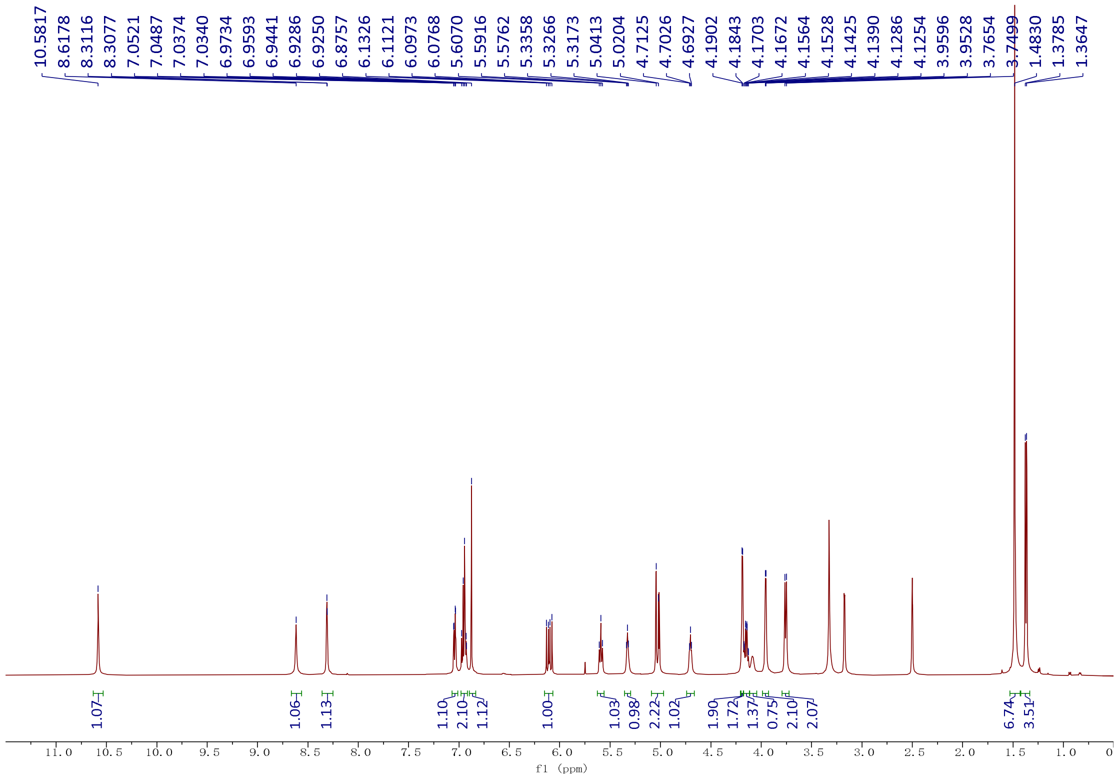

Figure S3. ^{13}C NMR (125 MHz, DMSO-d_6) and DEPT spectra of compound **1**.

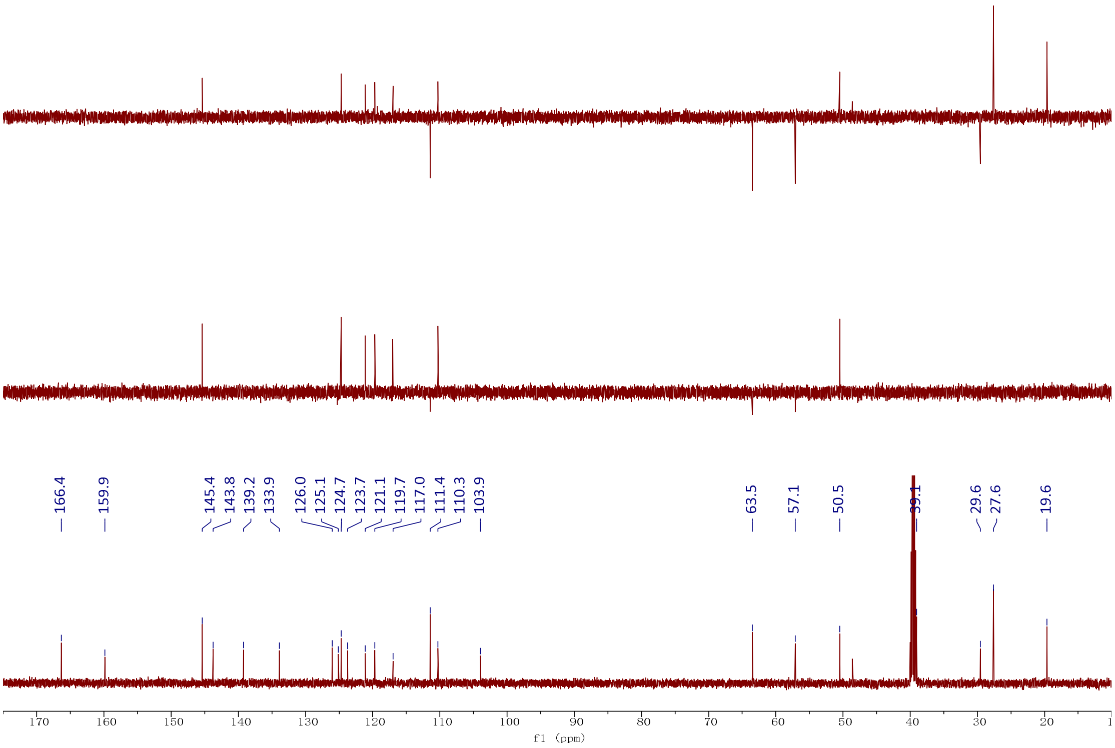

Figure S4. COSY spectrum of compound **1**.

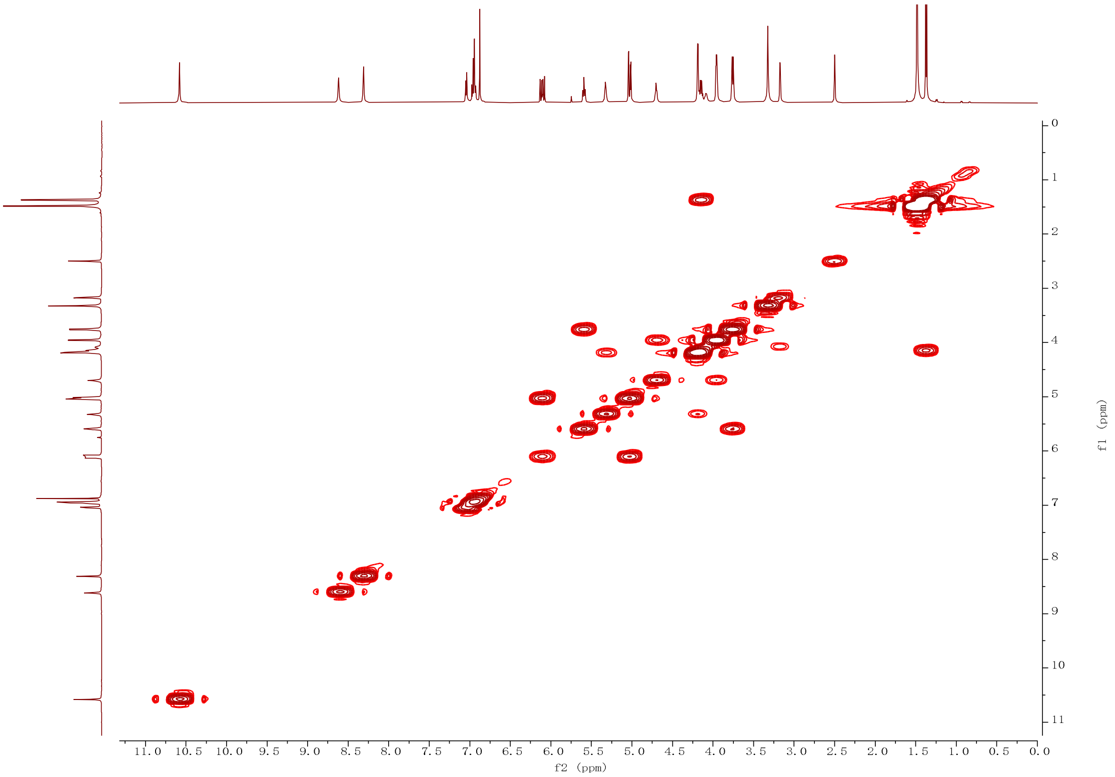

Figure S5. HSQC spectrum of compound **1**.

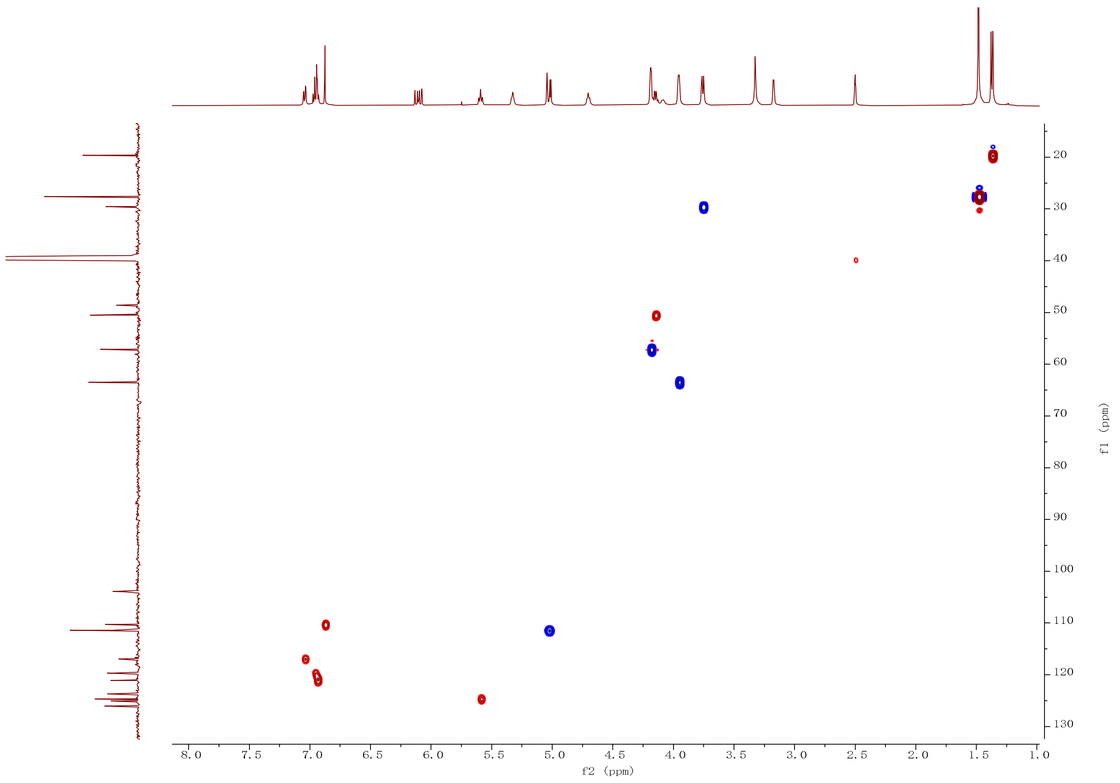

Figure S6. HMBC spectrum of compound **1**.

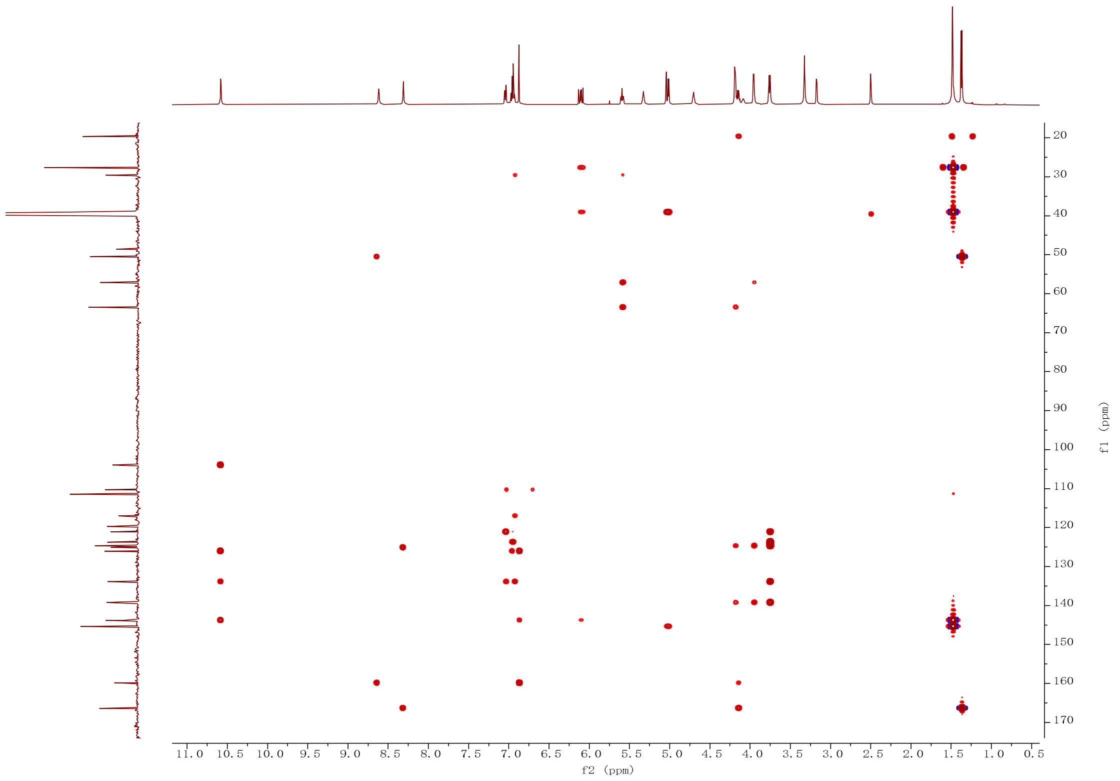

Figure S7. HR-ESI-MS spectrum of compound **1**.

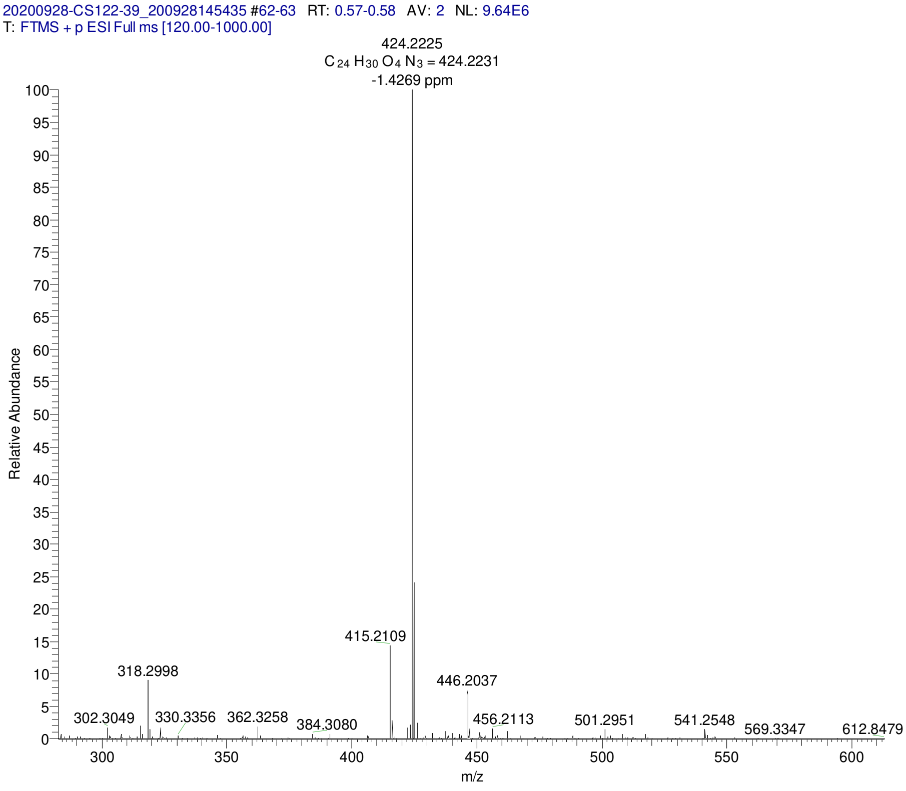

Figure S8. ECD spectrum of compound **1**.

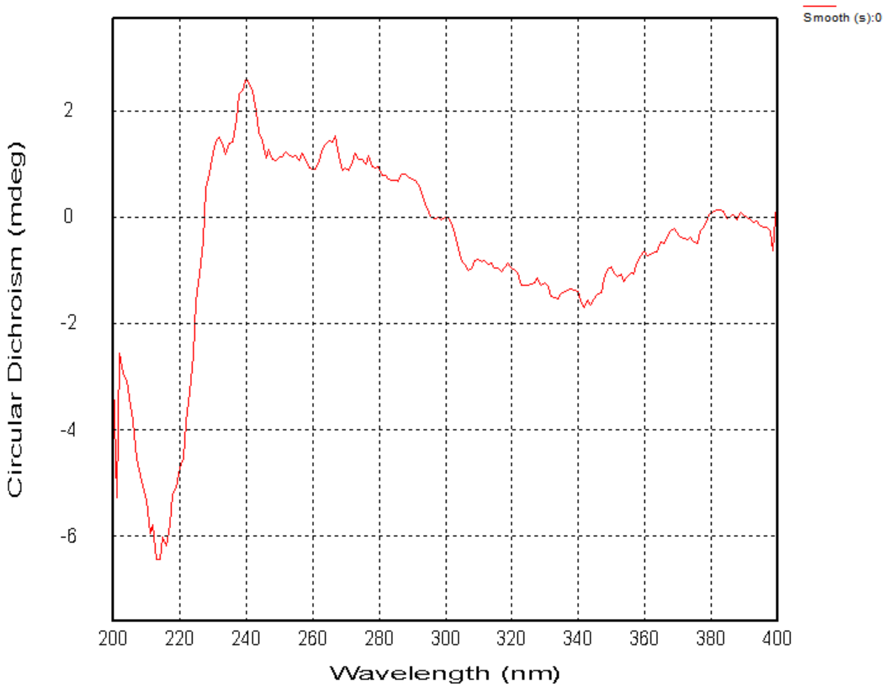

Figure S9. Chiral HPLC analysis of the acidic hydrolysate of compound **2**.

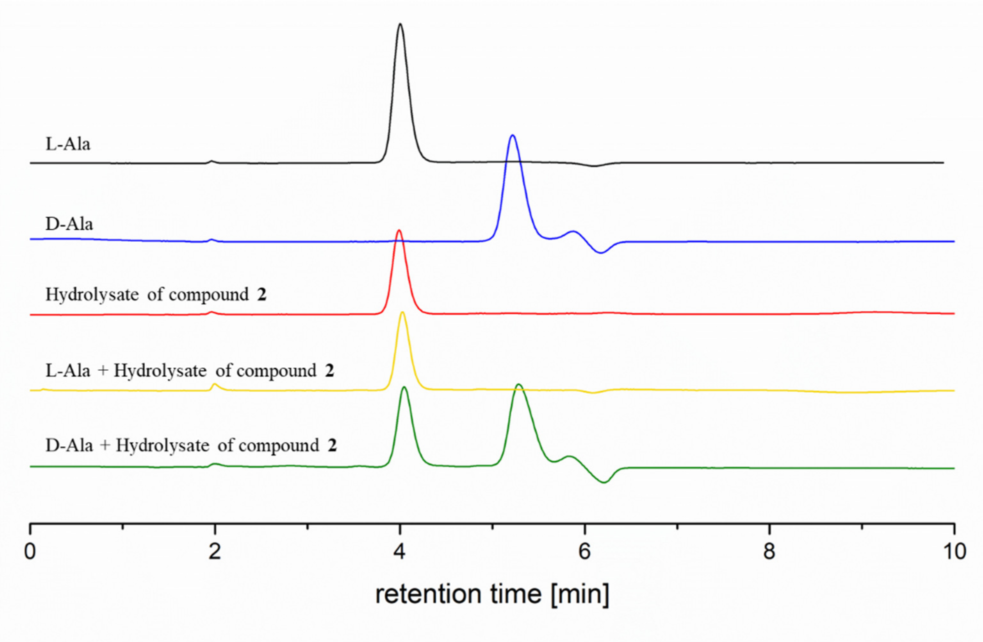

Figure S10. ^1H NMR (500 MHz, DMSO-d_6) spectrum of compound **2**.

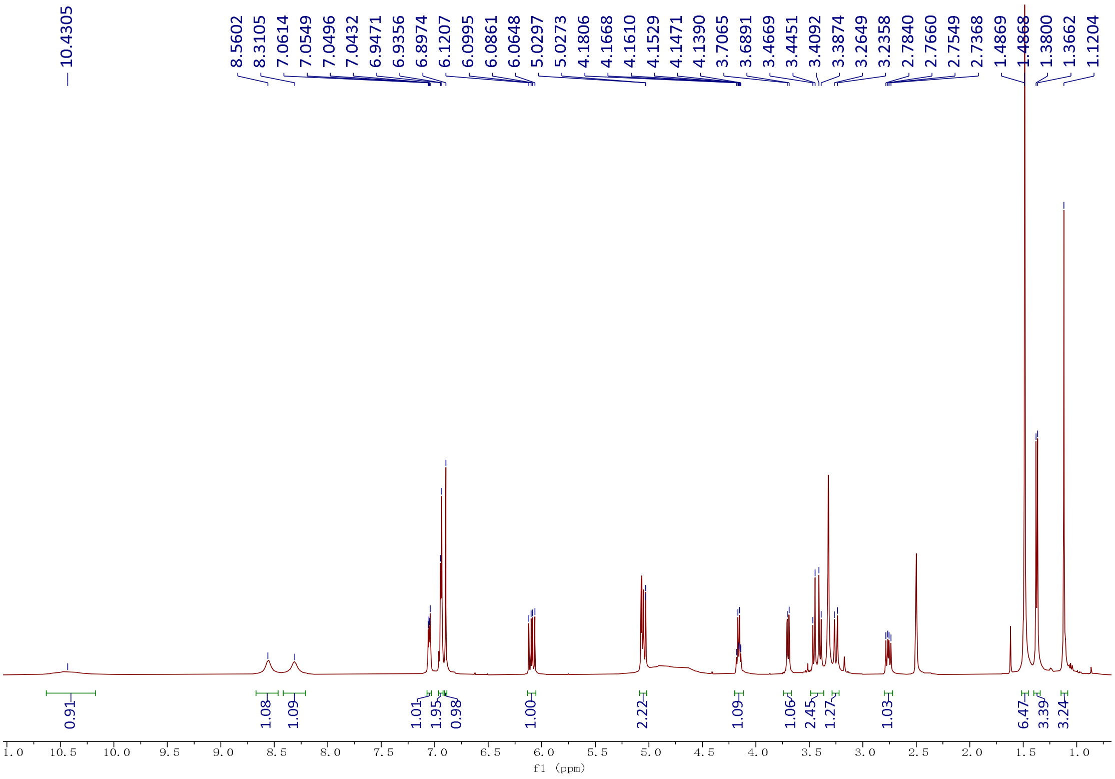

Figure S11. ^{13}C NMR (125 MHz, DMSO-*d6*) and DEPT spectra of compound **2**.

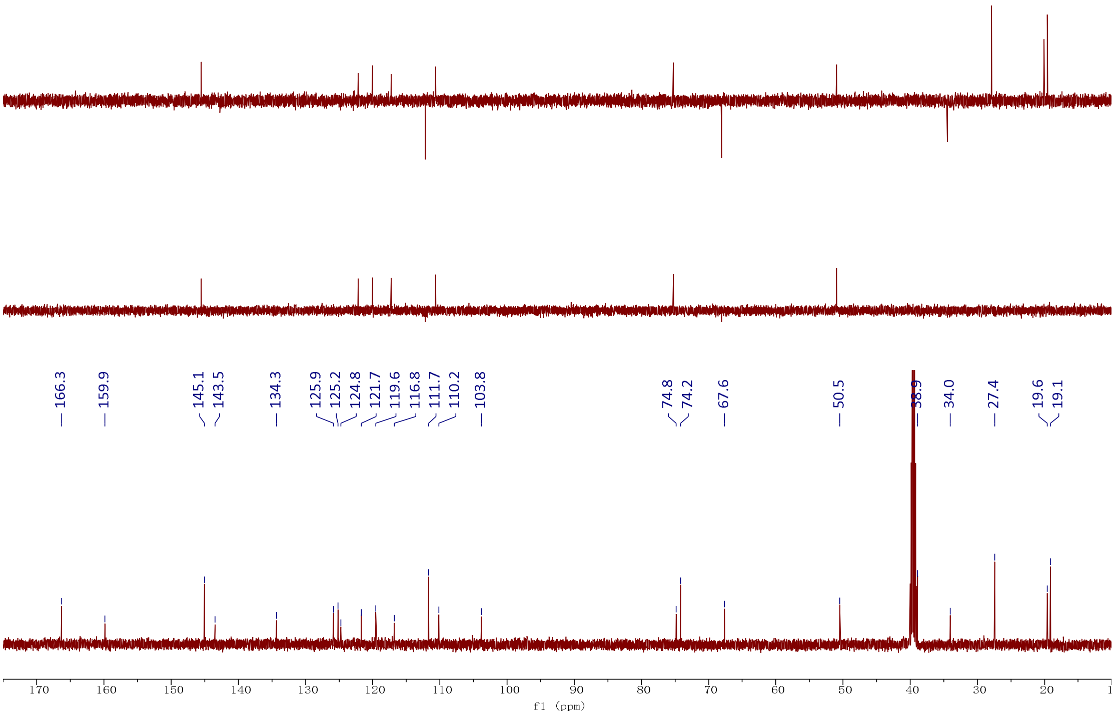

Figure S12. COSY spectrum of compound **2**.

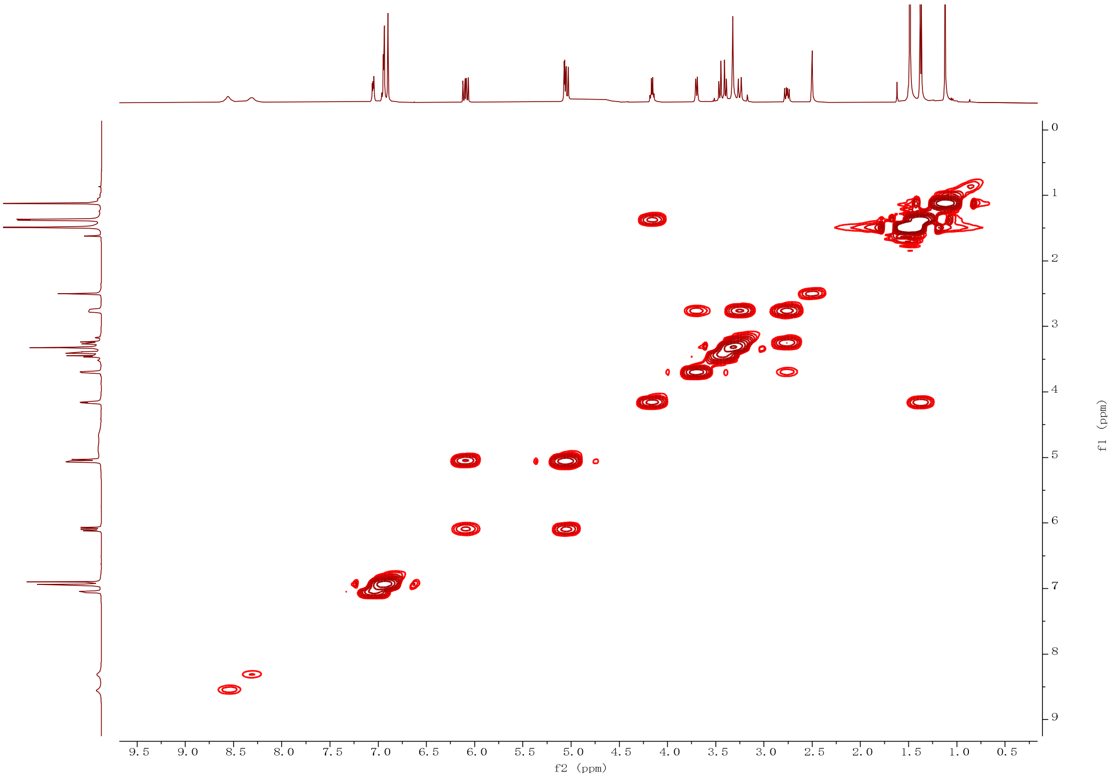

Figure S13. HSQC spectrum of compound **2**.

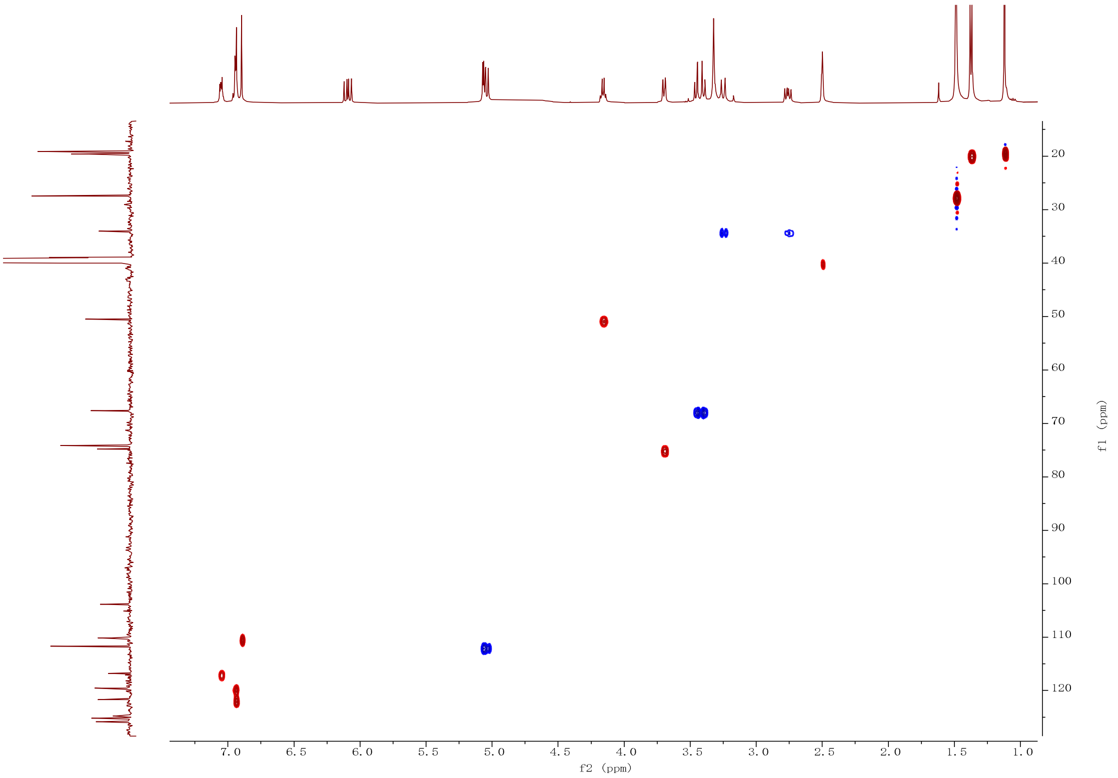

Figure S14. HMBC spectrum of compound **2**.

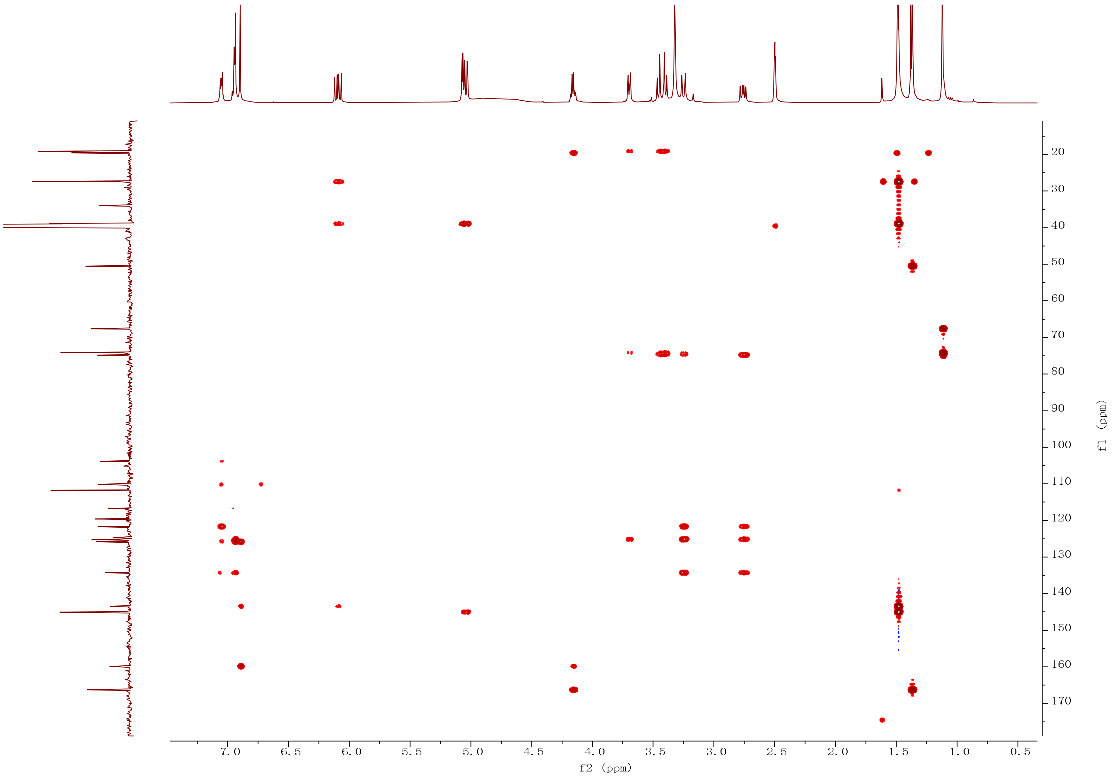

Figure S15. NOESY spectrum of compound **2**.

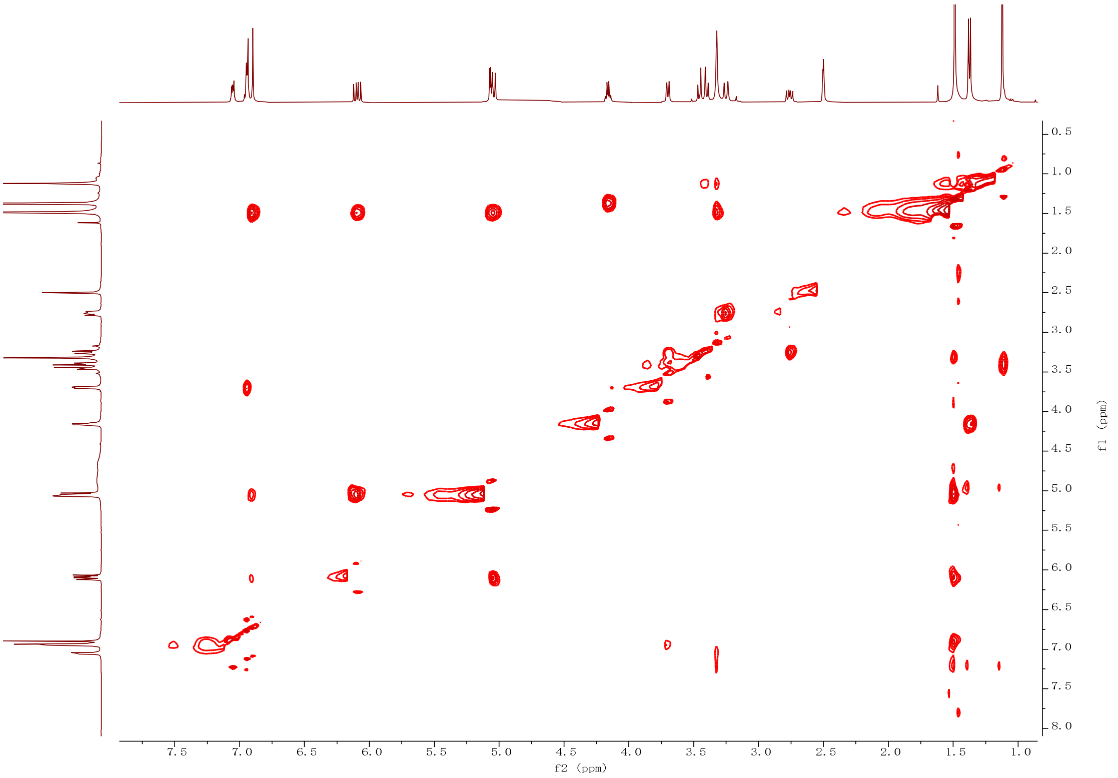

Figure S16. HR-ESI-MS spectrum of compound **2**.

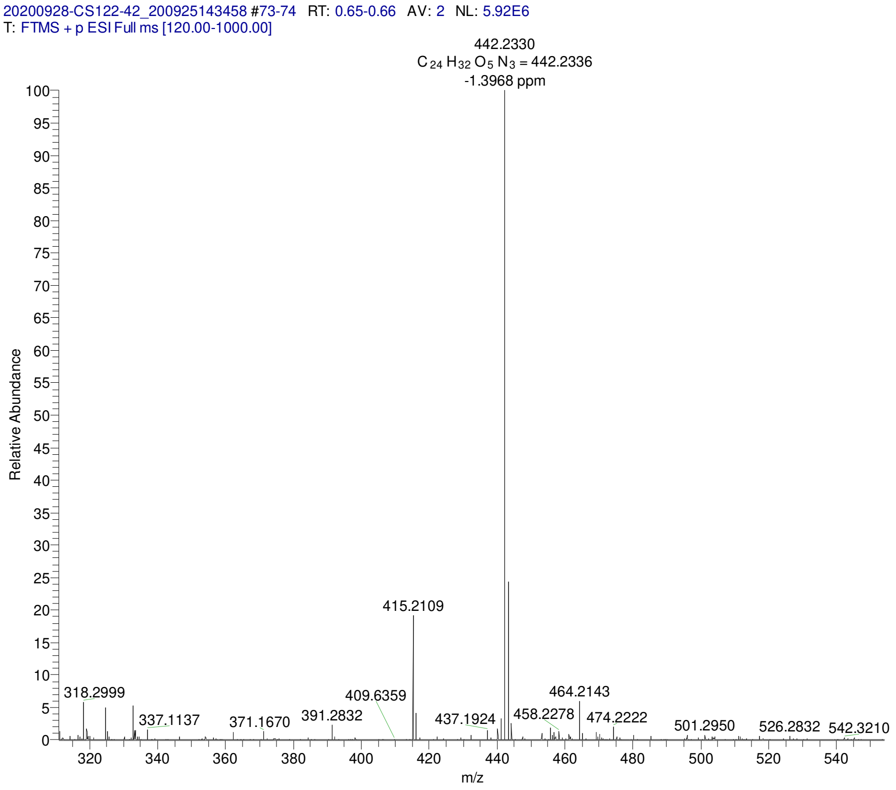

Figure S17. ECD spectrum of compound **2**.

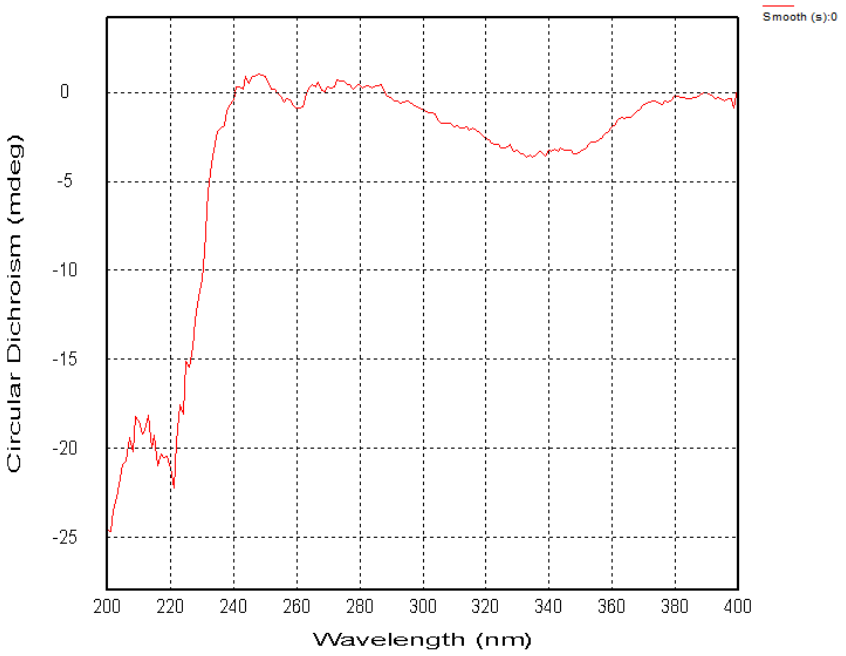

Figure S18. Structures of four possible isomers for DP4+ probability analysis of compound **2**.

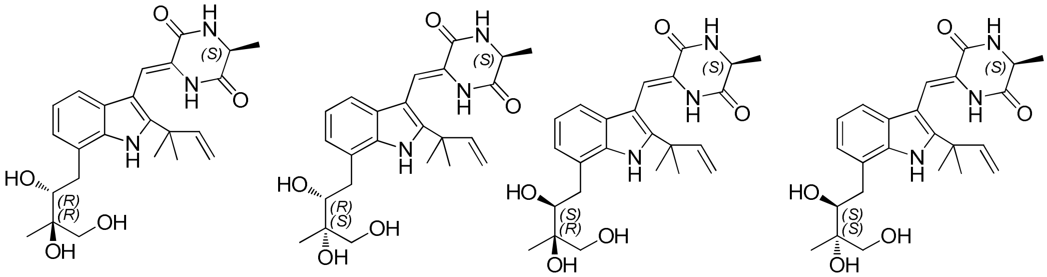

Figure S19. Optimized geometries of predominant conformers (weighting factors) for isomer **2a** at the B3LYP/6-31+g(d,p) level. A total of 152 conformers were searched and 8 of them possess Boltzmann distributions over 2%.

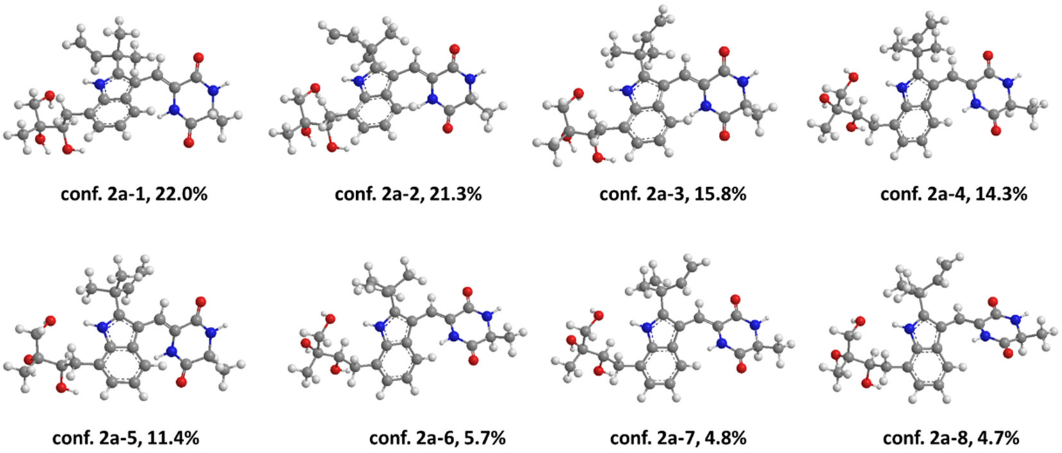

Figure S20. Optimized geometries of predominant conformers (weighting factors) for isomer **2b** at the B3LYP/6-31+g(d,p) level. A total of 132 conformers were searched and 7 of them possess Boltzmann distributions over 2%.

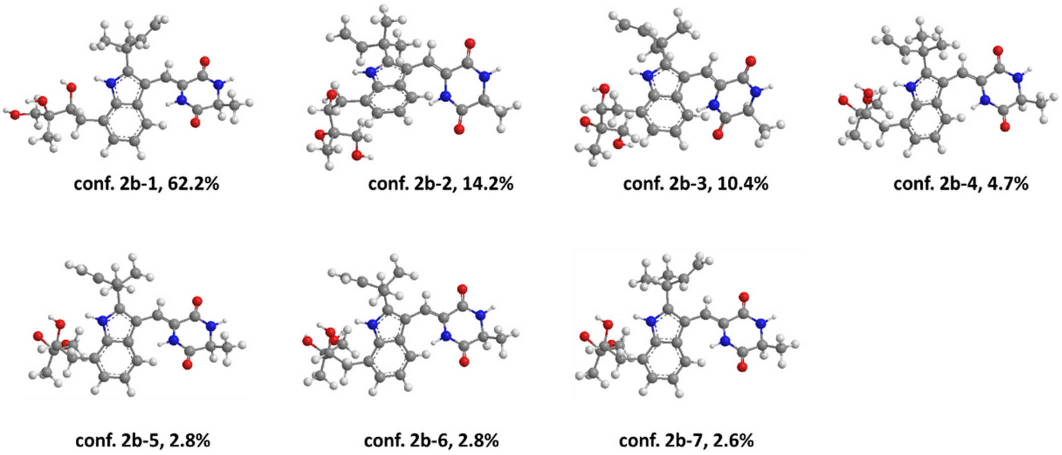

Figure S21. Optimized geometries of predominant conformers (weighting factors) for isomer **2c** at the B3LYP/6-31+g(d,p) level. A total of 178 conformers were searched and 8 of them possess Boltzmann distributions over 2%.

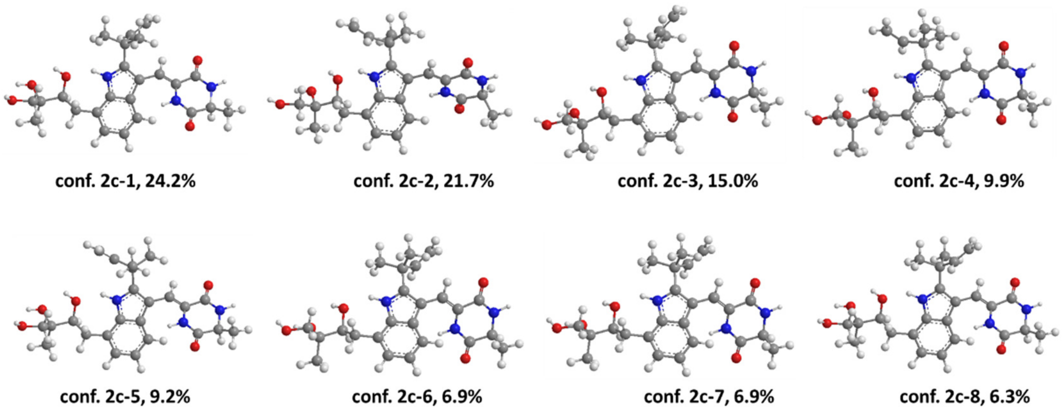

Figure S22. Optimized geometries of predominant conformers (weighting factors) for isomer **2d** at the B3LYP/6-31+g(d,p) level. A total of 177 conformers were searched and 7 of them possess Boltzmann distributions over 2%.

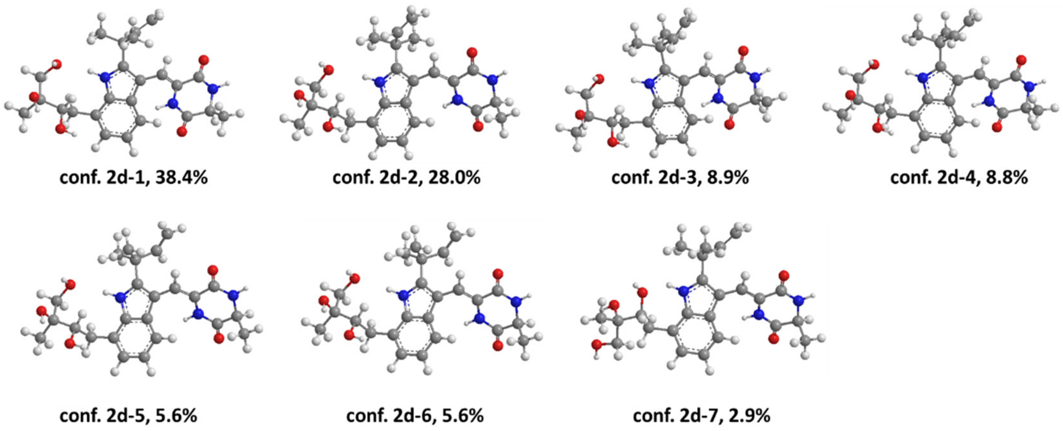

Figure S23. Chiral HPLC analysis of the acidic hydrolysate of compound **3**.

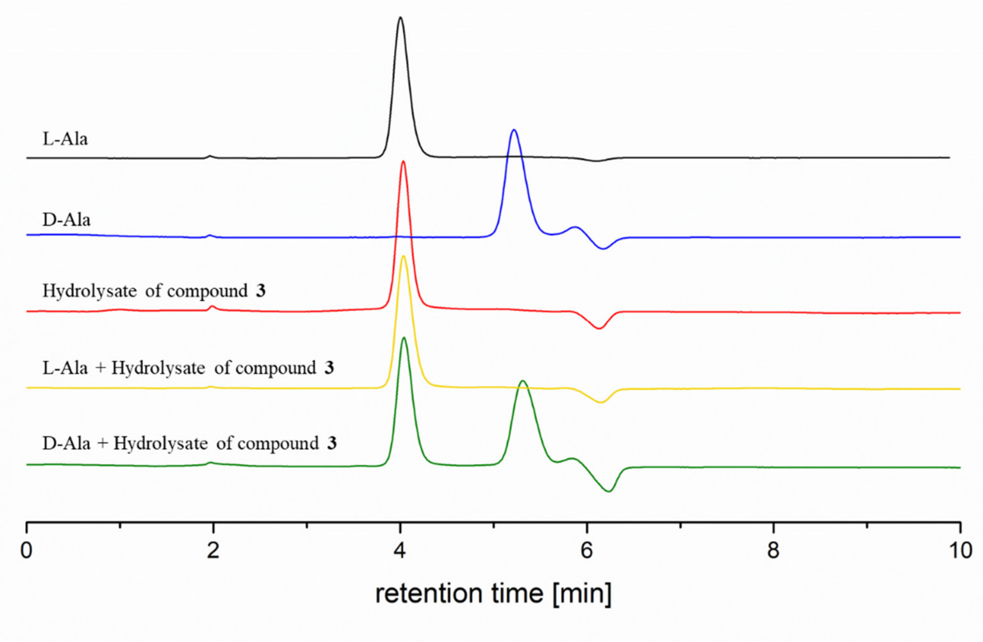

Figure S24. ^{1}H NMR (500 MHz, DMSO-d_6) spectrum of compound **3**.

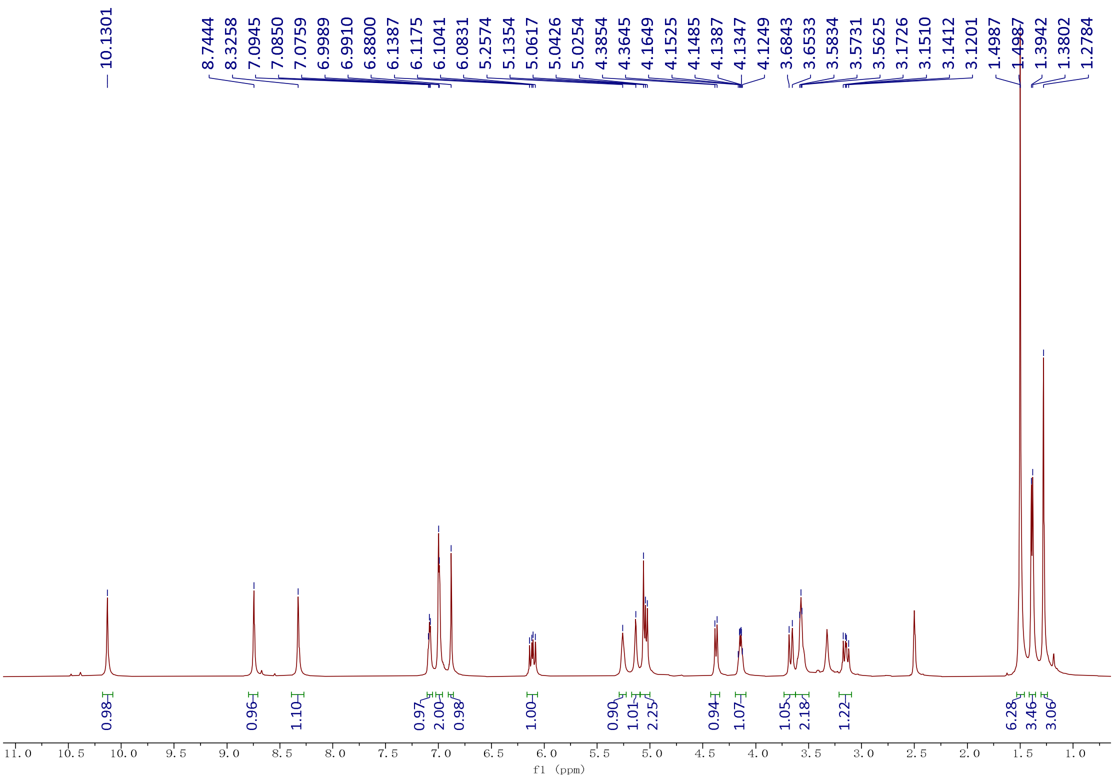

Figure S25. ^{13}C NMR (125 MHz, DMSO-*d*$_6$) and DEPT spectra of compound **3**.

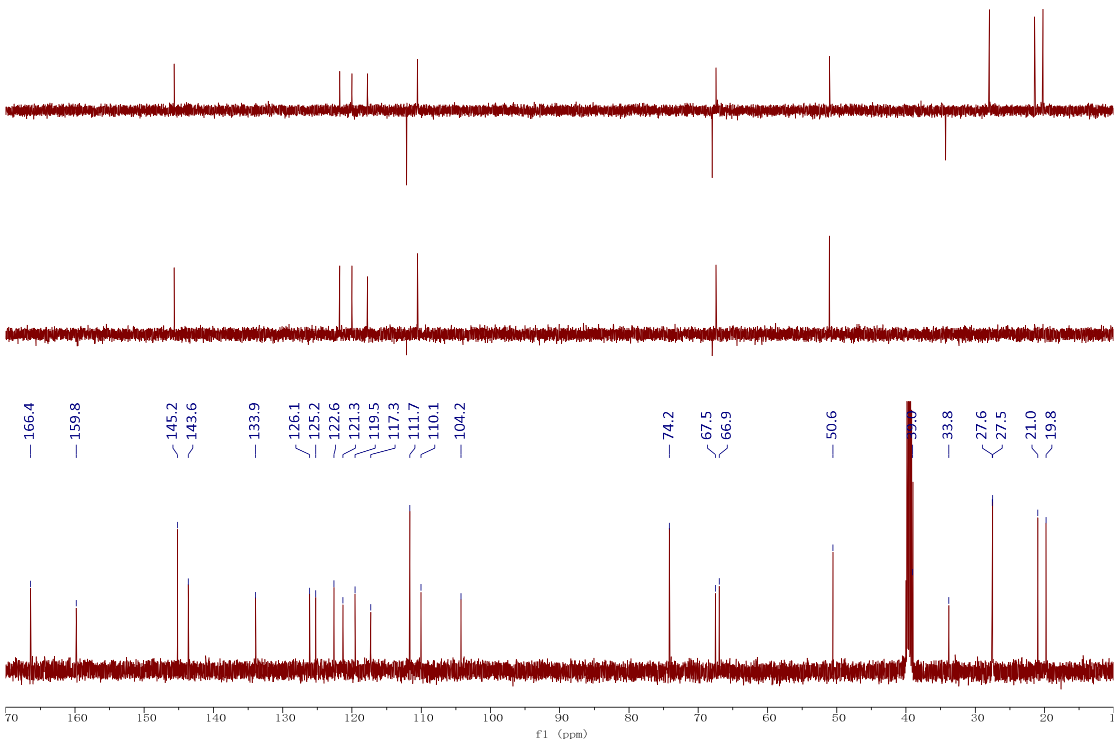

Figure S26. COSY spectrum of compound **3**.

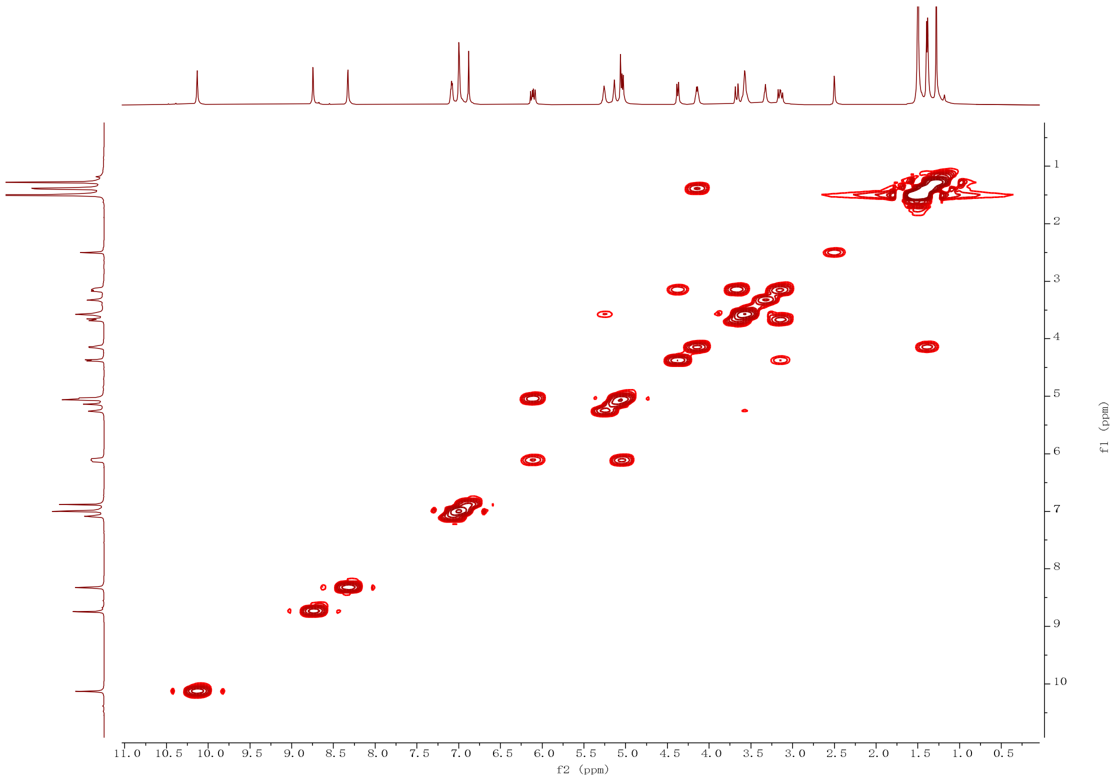

Figure S27. HSQC spectrum of compound **3**.

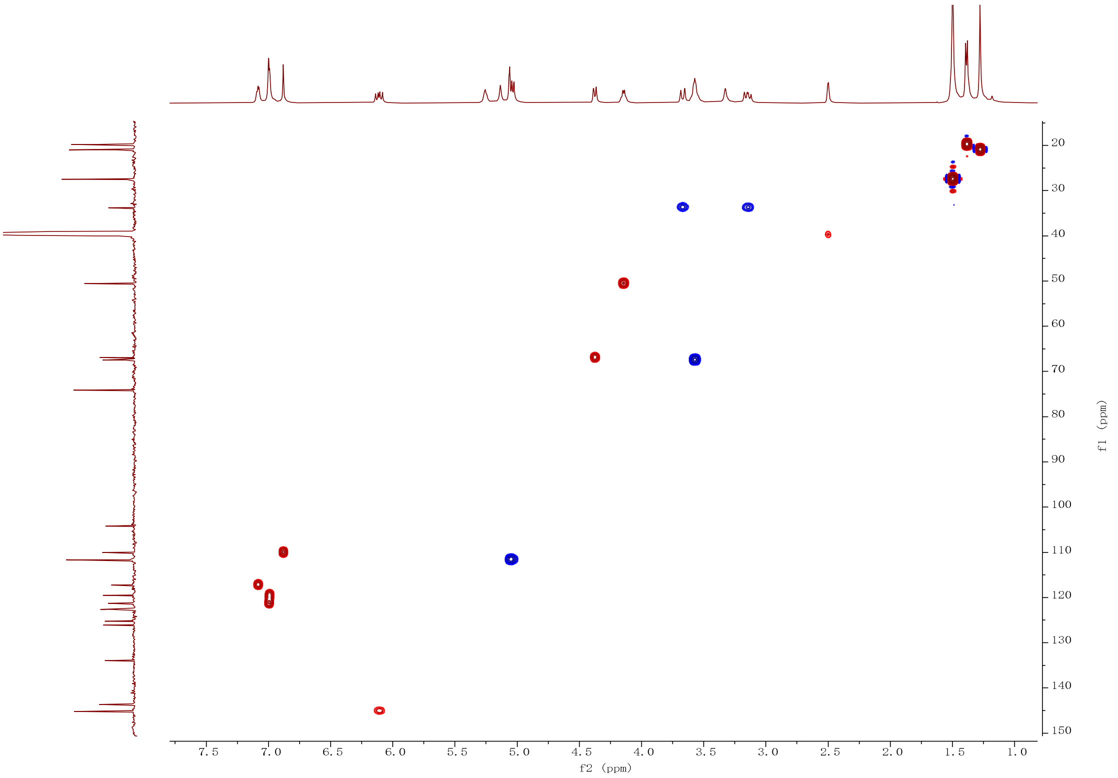

Figure S28. HMBC spectrum of compound **3**.

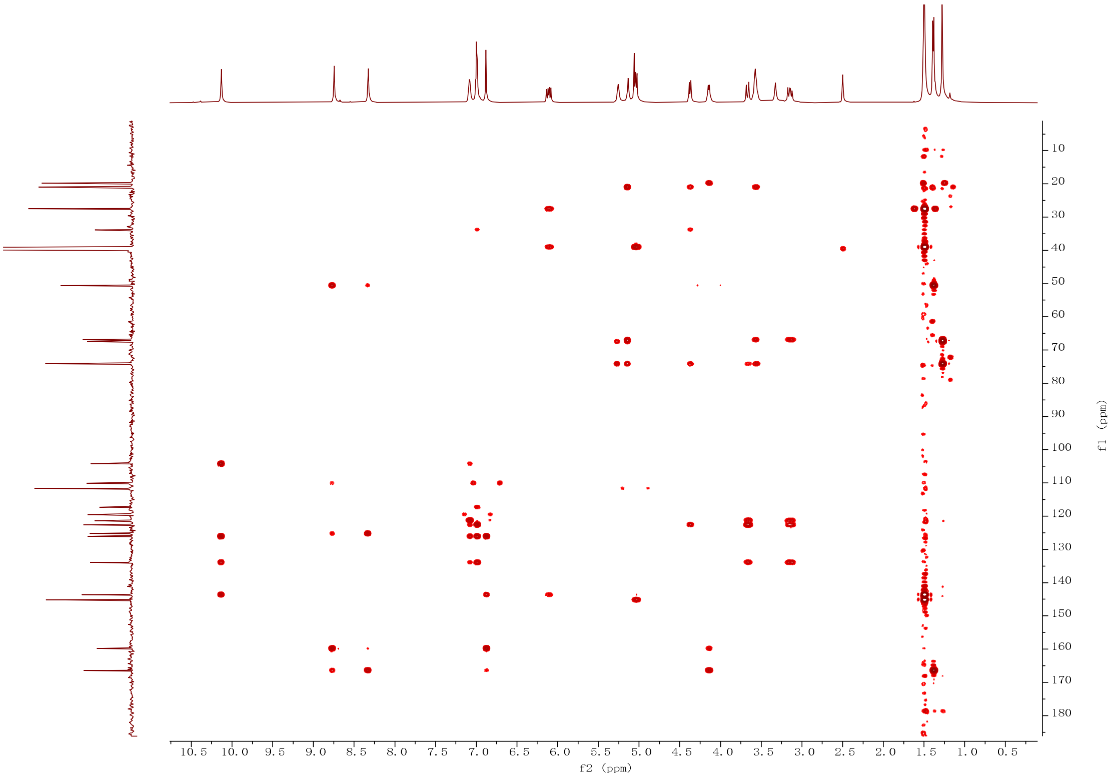

Figure S29. NOESY spectrum of compound **3**.

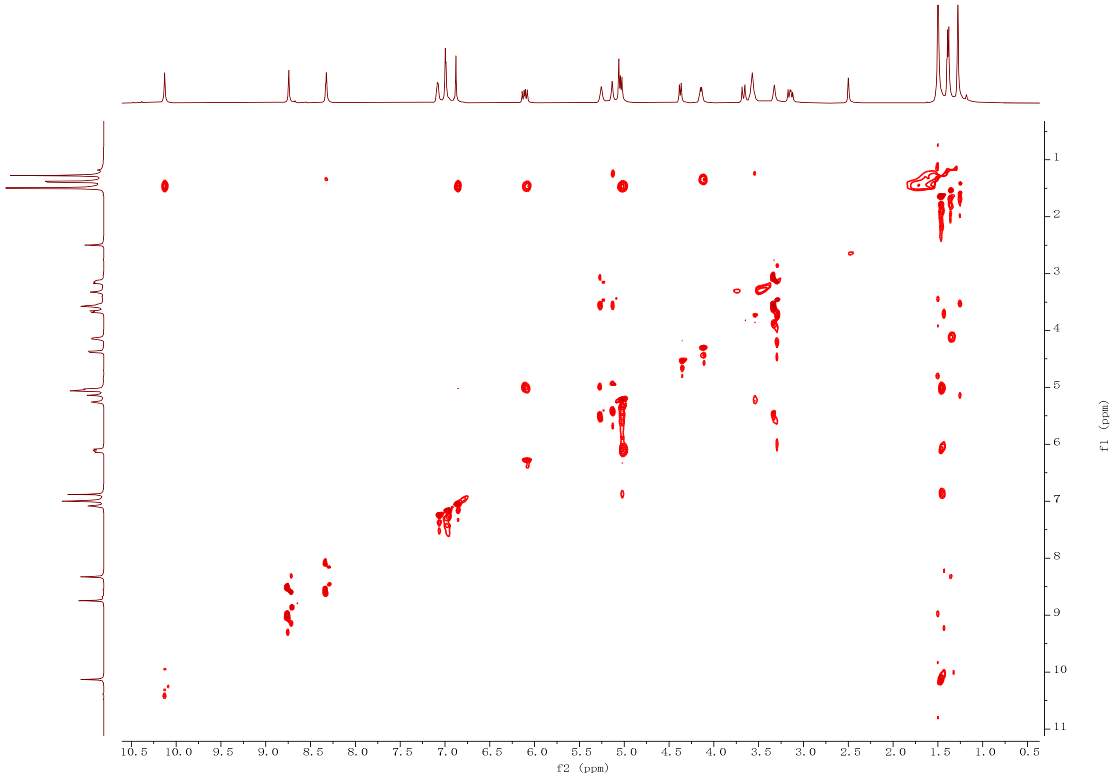

Figure S30. HR-ESI-MS spectrum of compound **3**.

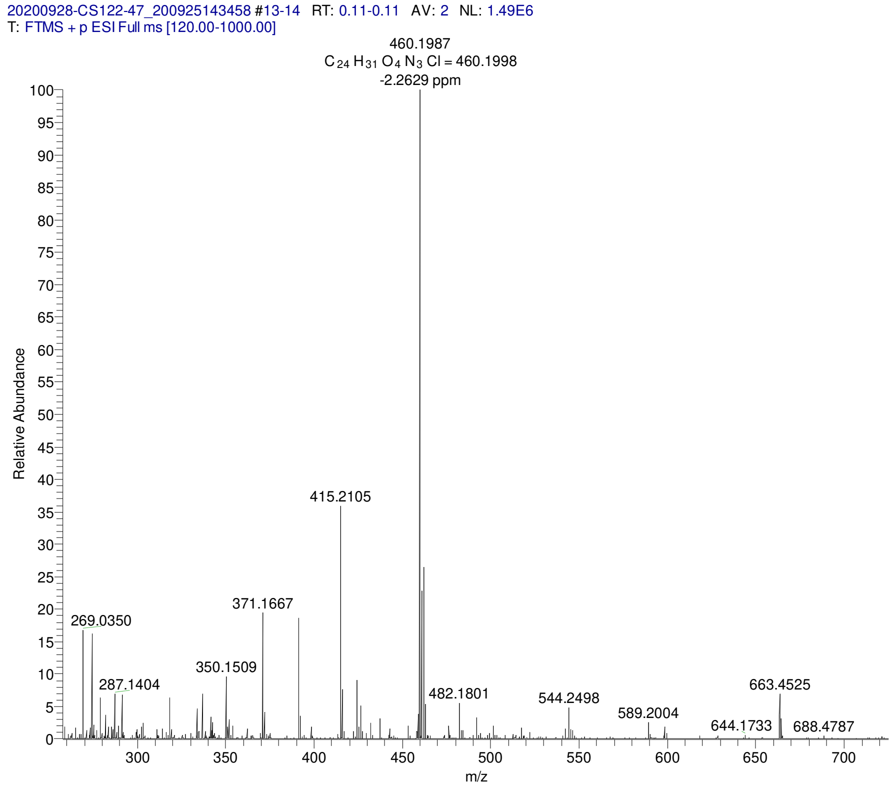

Figure S31. ECD spectrum of compound **3**.

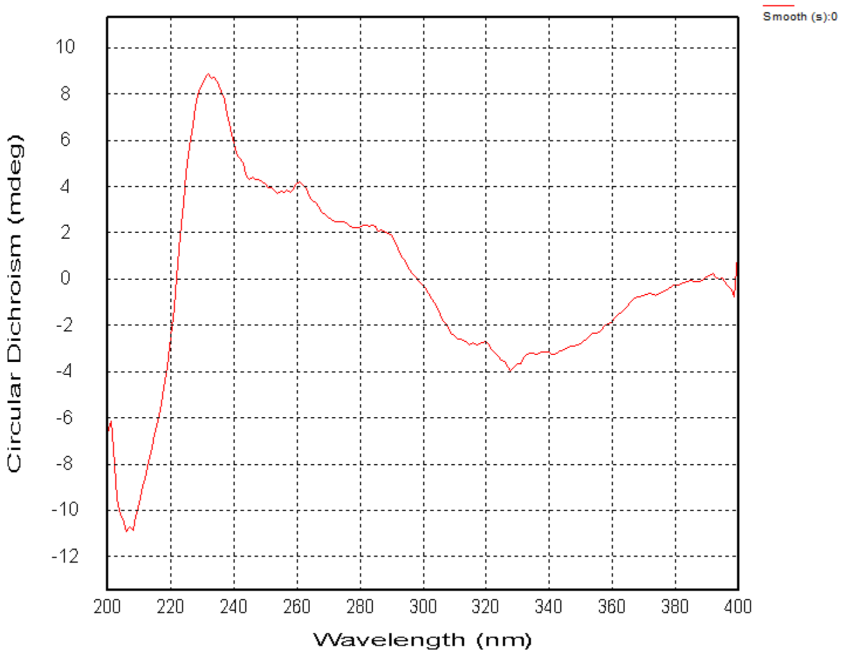

Figure S32. Structures of four possible isomers for DP4+ probability analysis of compound **3**.

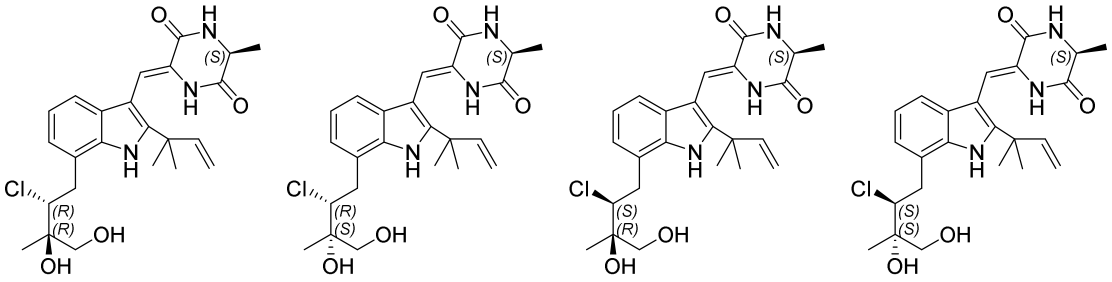

Figure S33. Optimized geometries of predominant conformers (weighting factors) for isomer **3a** at the B3LYP/6-31+g(d,p) level. A total of 155 conformers were searched and 6 of them possess Boltzmann distributions over 2%.

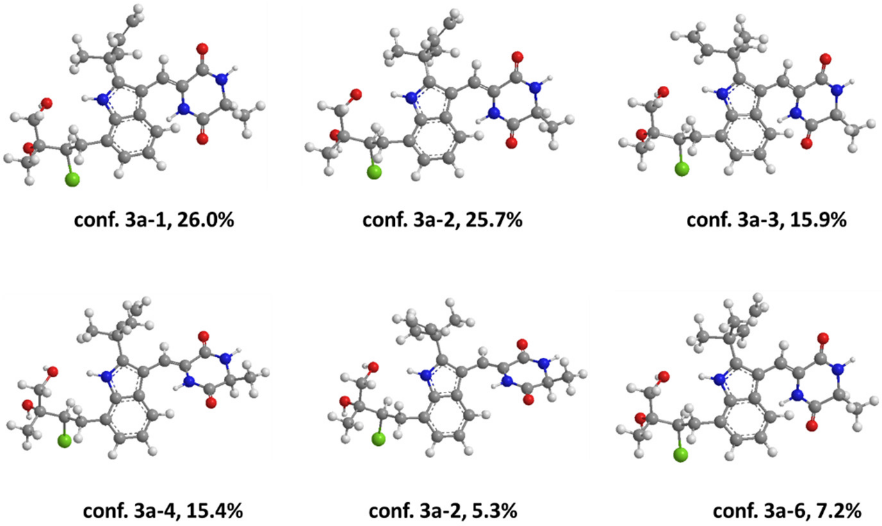

Figure S34. Optimized geometries of predominant conformers (weighting factors) for isomer **3b** at the B3LYP/6-31+g(d,p) level. A total of 168 conformers were searched and 8 of them possess Boltzmann distributions over 2%.

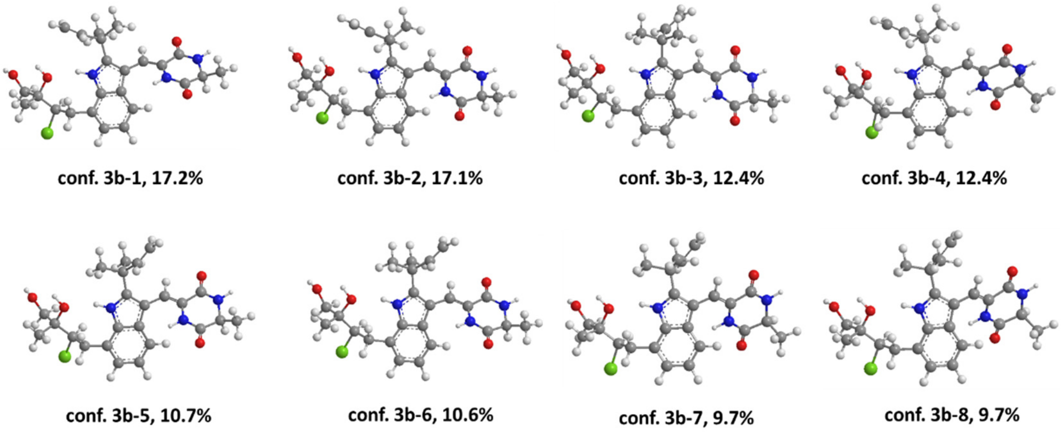

Figure S35. Optimized geometries of predominant conformers (weighting factors) for isomer **3c** at the B3LYP/6-31+g(d,p) level. A total of 176 conformers were searched and 8 of them possess Boltzmann distributions over 2%.

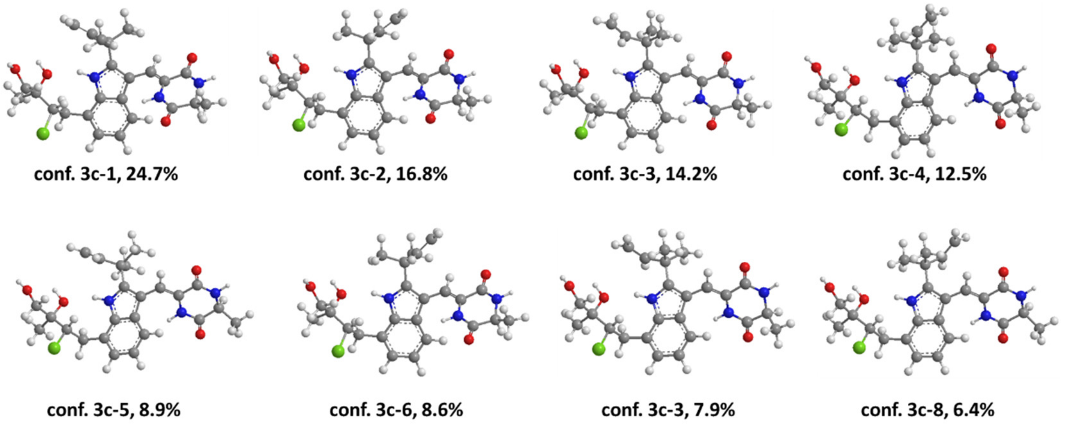

Figure S36. Optimized geometries of predominant conformers (weighting factors) for isomer **3d** at the B3LYP/6-31+g(d,p) level. A total of 146 conformers were searched and 8 of them possess Boltzmann distributions over 2%.

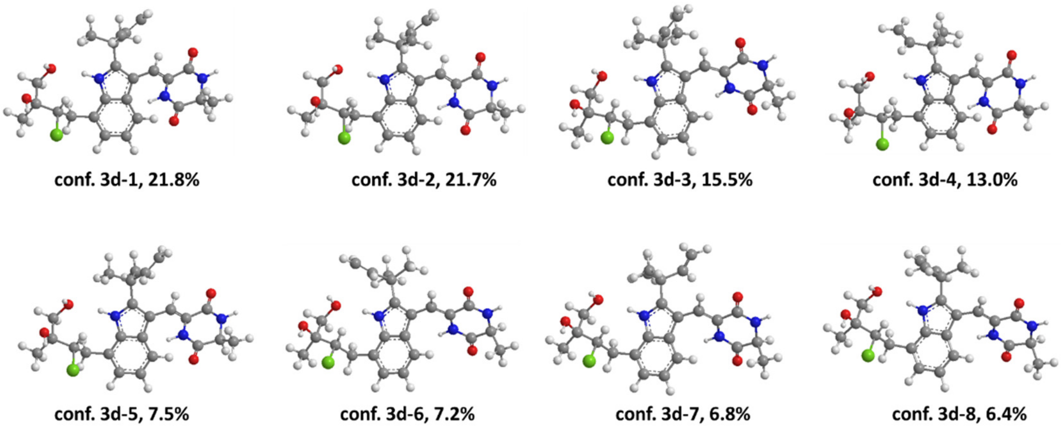

Figure S37. Chiral HPLC analysis of the acidic hydrolysate of compound **4**.

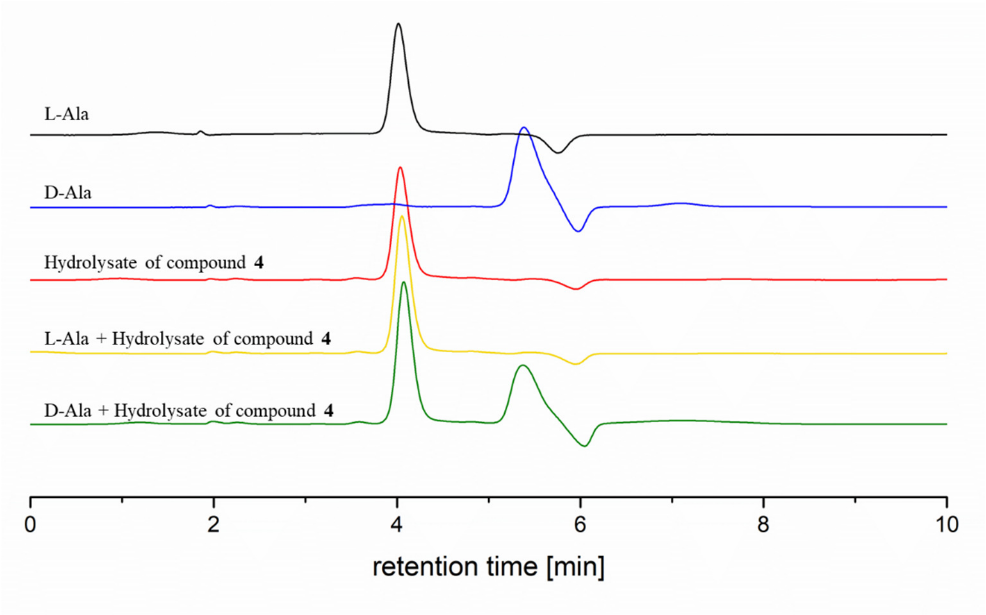

Figure S38. ^{1}H NMR (500 MHz, DMSO-d_6) spectrum of compound **4**.

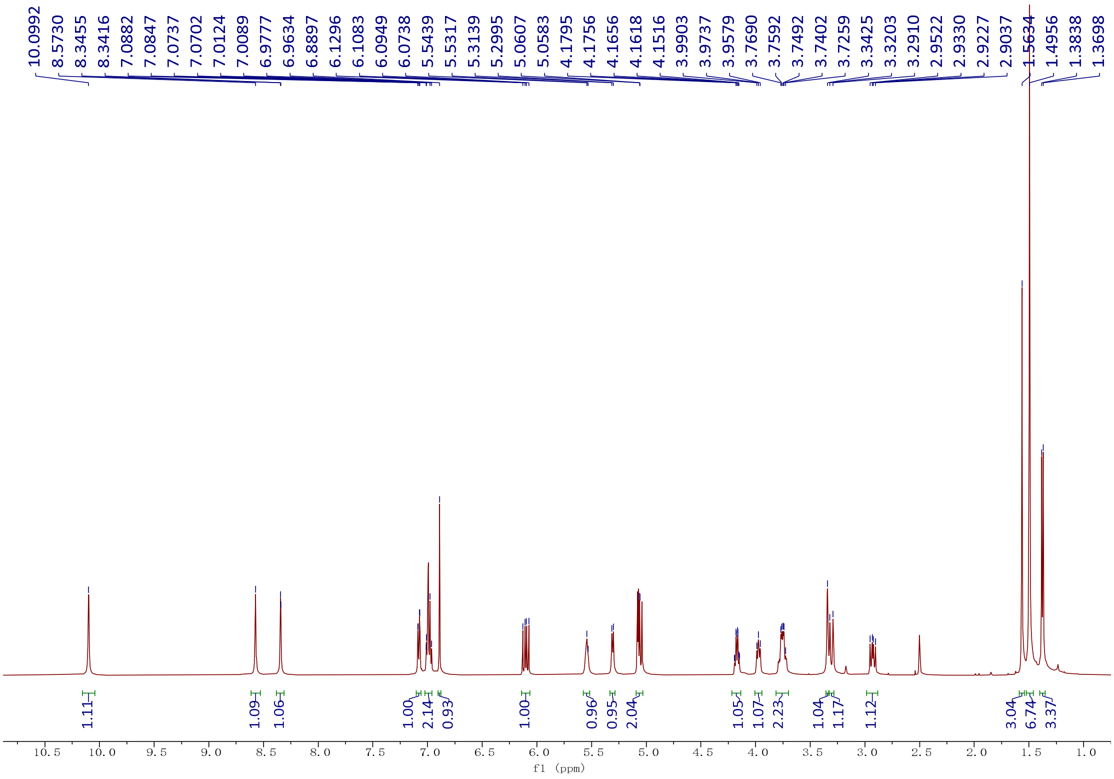

Figure S39. ^{13}C NMR (125 MHz, DMSO-d_6) and DEPT spectra of compound **4**.

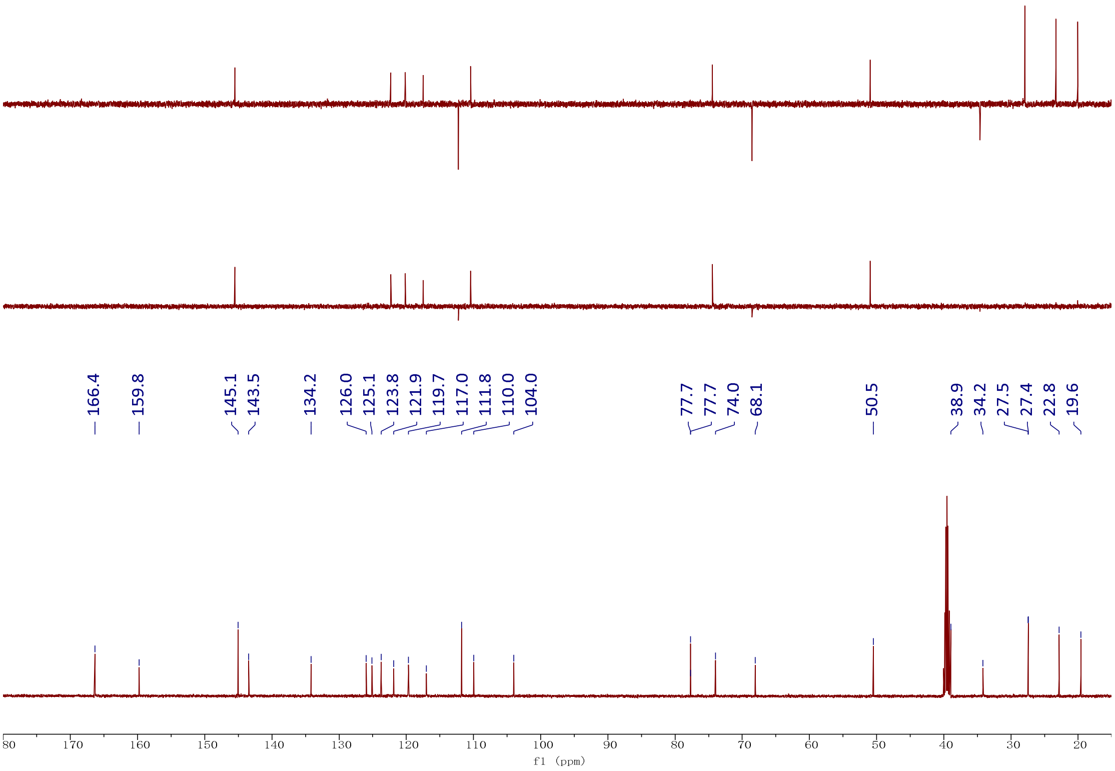

Figure S40. COSY spectrum of compound **4**.

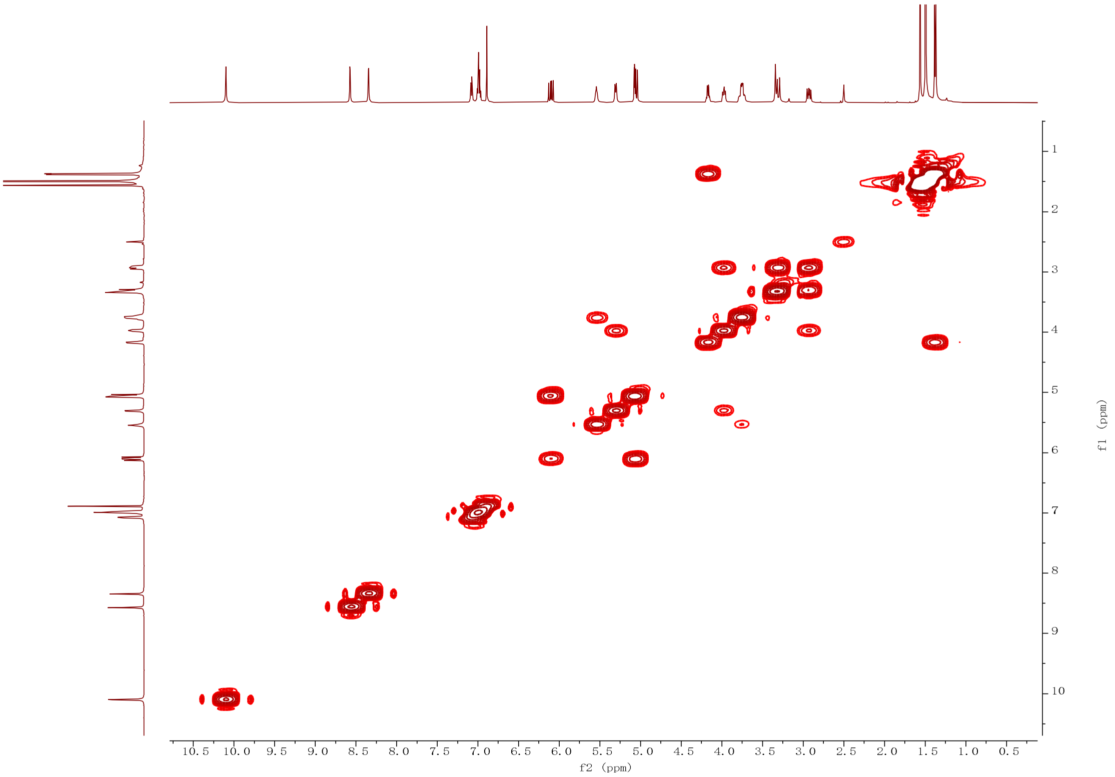

Figure S41. HSQC spectrum of compound **4**.

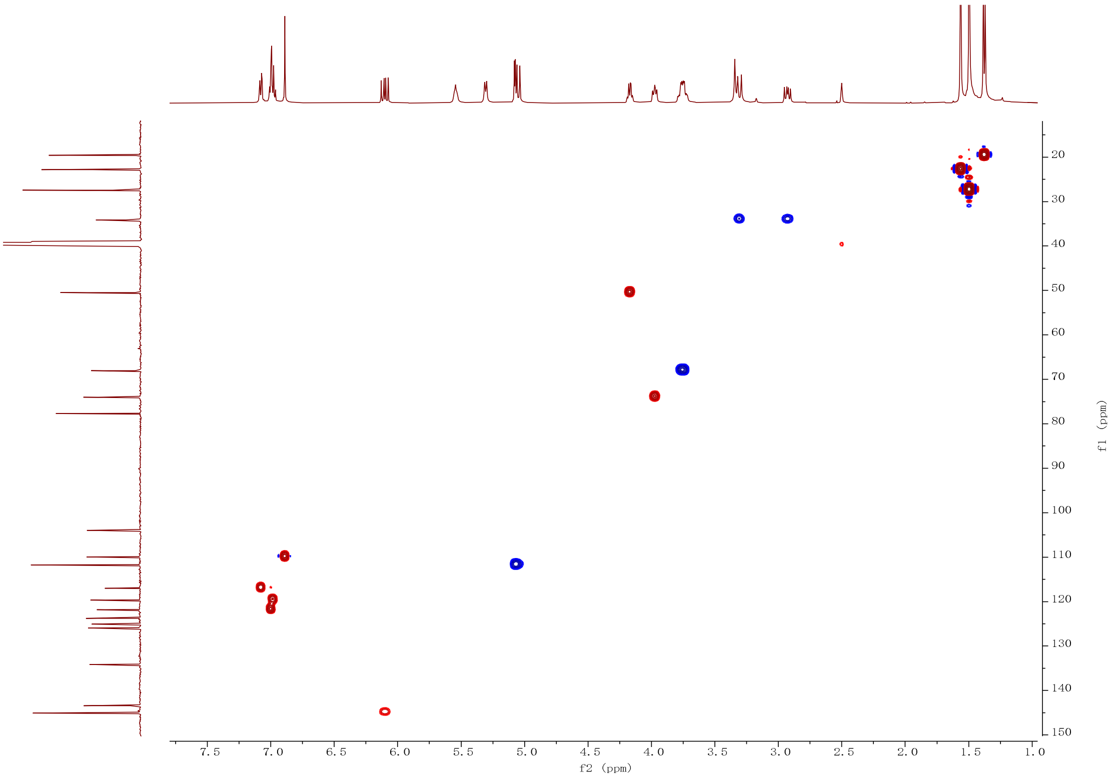

Figure S42. HMBC spectrum of compound **4**.

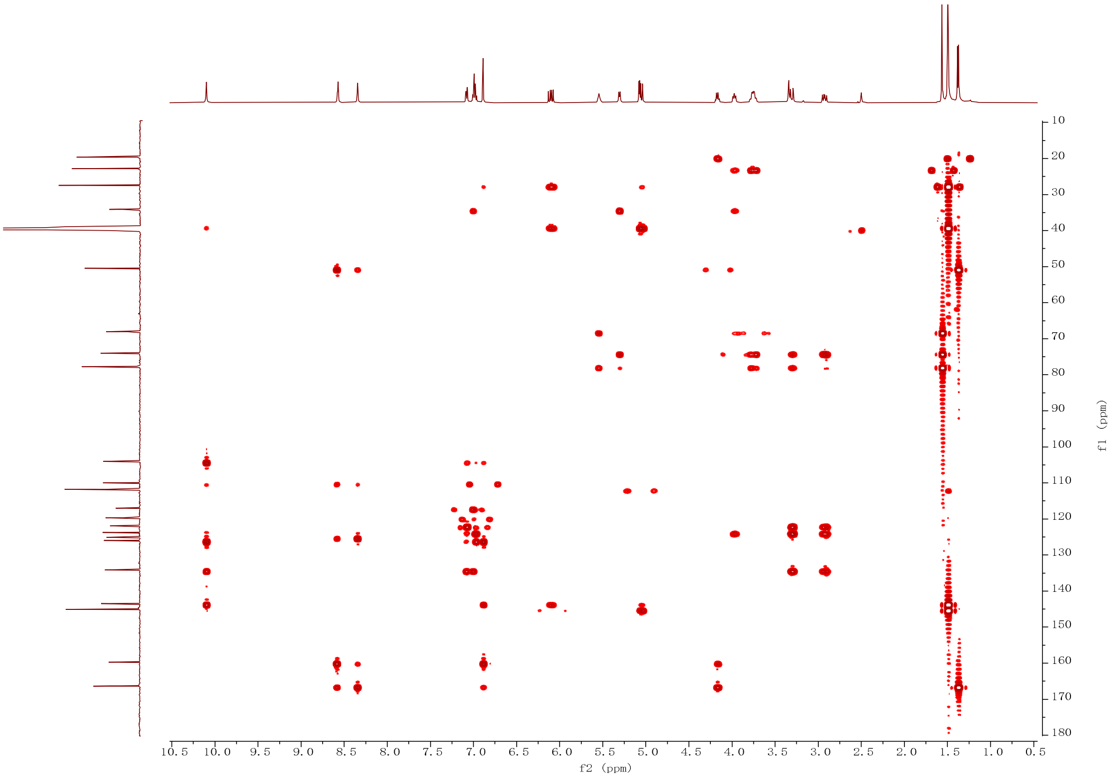

Figure S43. NOESY spectrum of compound **4**.

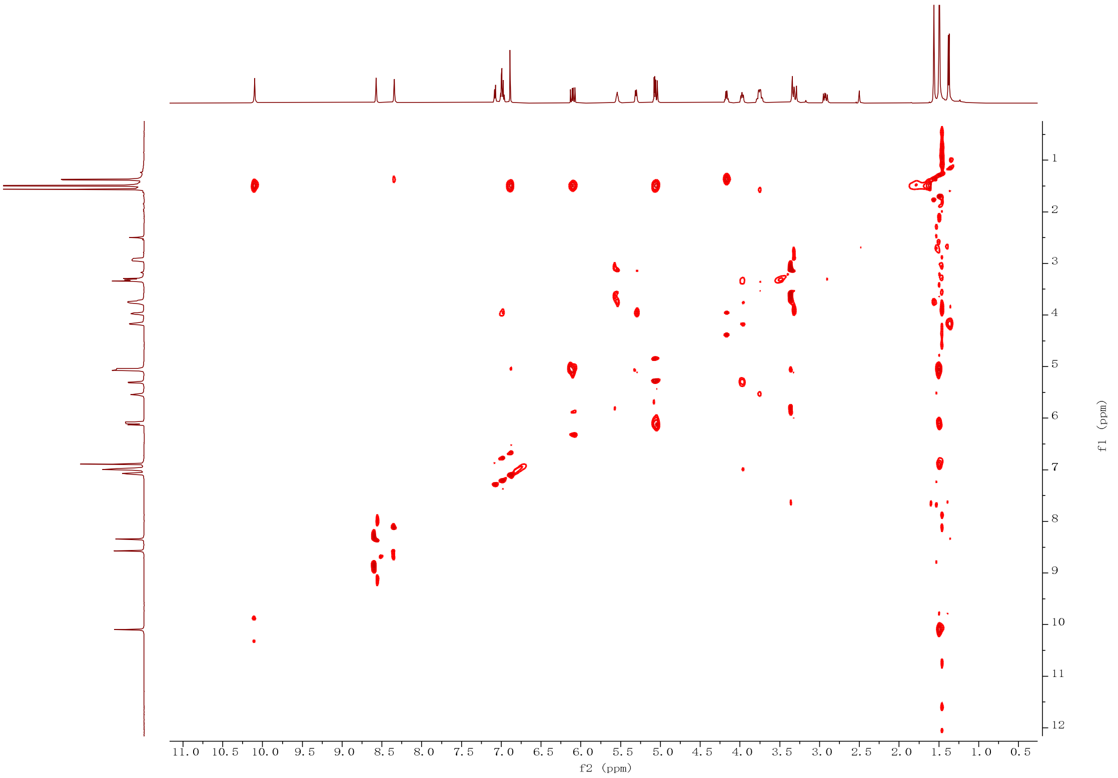

Figure S44. HR-ESI-MS spectrum of compound **4**.

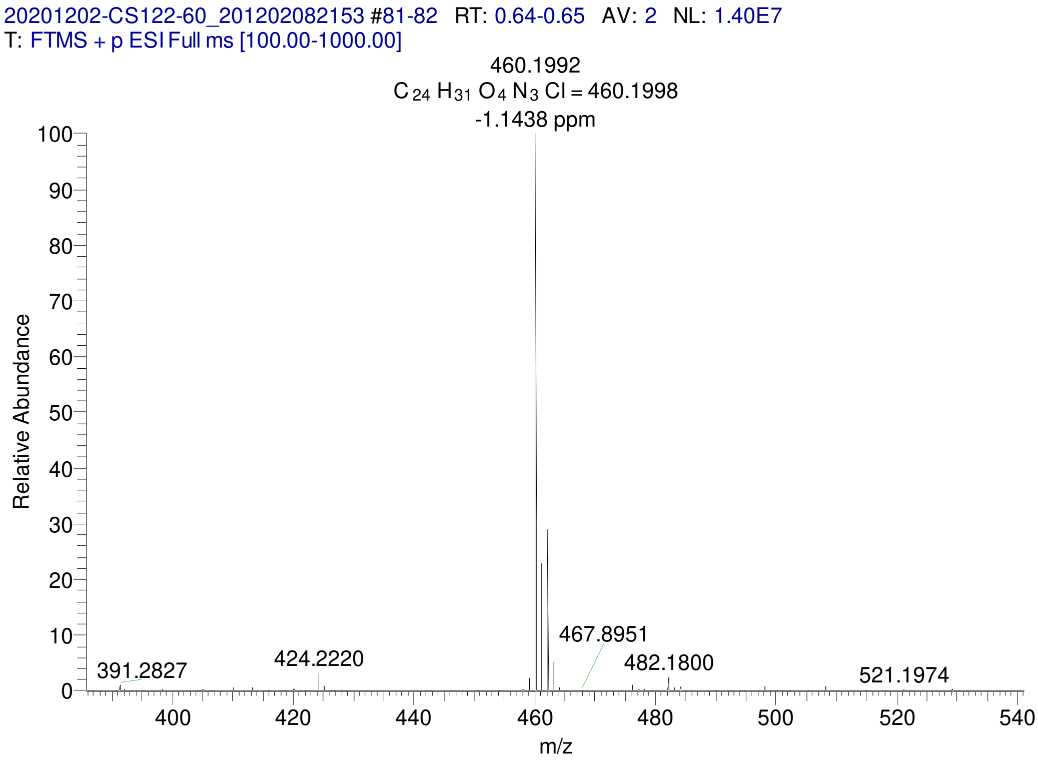

Figure S45. ECD spectrum of compound **4**.

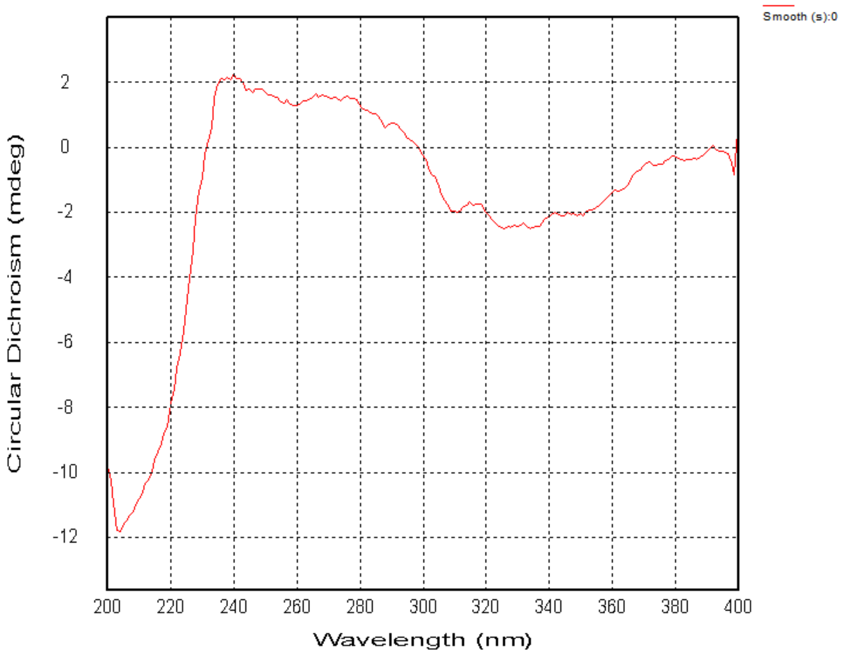

Figure S46. Structures of four possible isomers for DP4+ probability analysis of compound **4**.

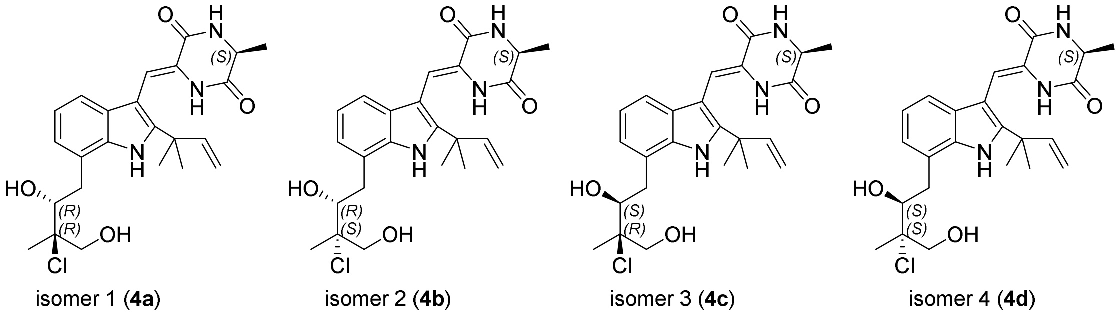

Figure S47. Optimized geometries of predominant conformers (weighting factors) for isomer **4a** at the B3LYP/6-31+g(d,p) level. A total of 270 conformers were searched and 7 of them possess Boltzmann distributions over 2%.

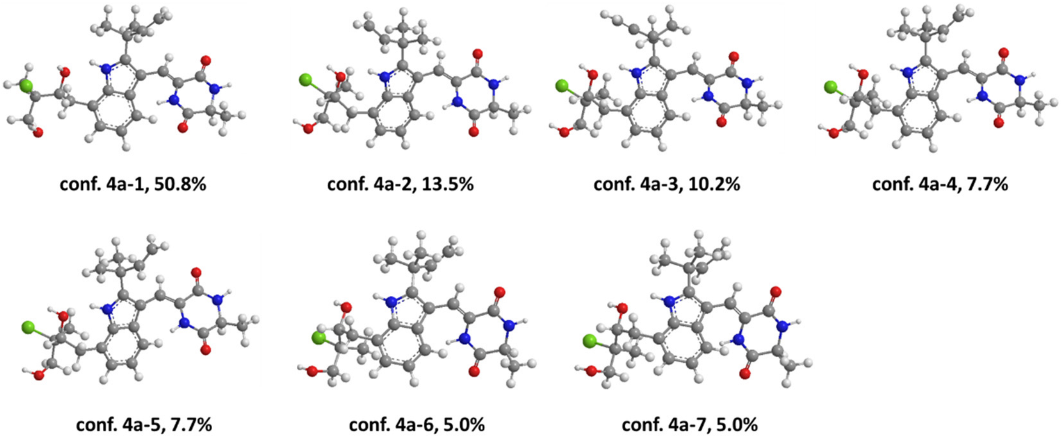

Figure S48. Optimized geometries of predominant conformers (weighting factors) for isomer **4b** at the B3LYP/6-31+g(d,p) level. A total of 349 conformers were searched and 4 of them possess Boltzmann distributions over 2%.

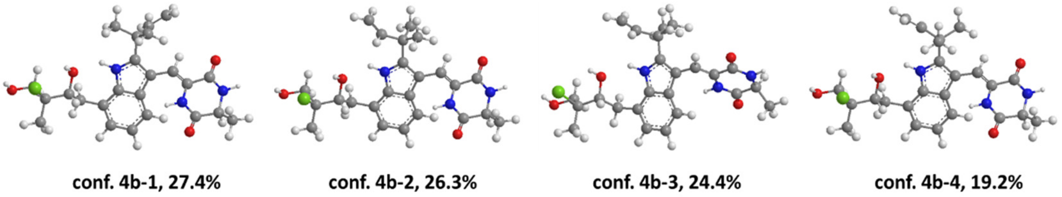

Figure S49. Optimized geometries of predominant conformers (weighting factors) for isomer **4c** at the B3LYP/6-31+g(d,p) level. A total of 327 conformers were searched and 6 of them possess Boltzmann distributions over 2%.

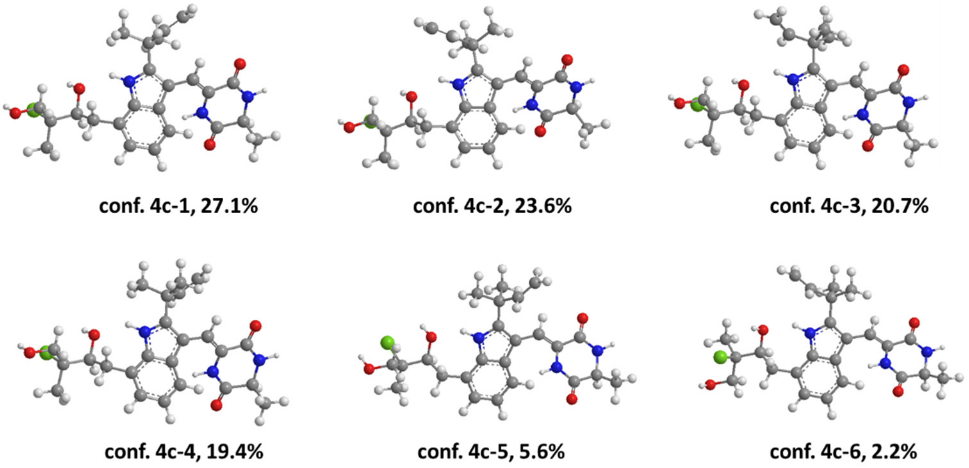

Figure S50. Optimized geometries of predominant conformers (weighting factors) for isomer **4d** at the B3LYP/6-31+g(d,p) level. A total of 281 conformers were searched and 6 of them possess Boltzmann distributions over 2%.

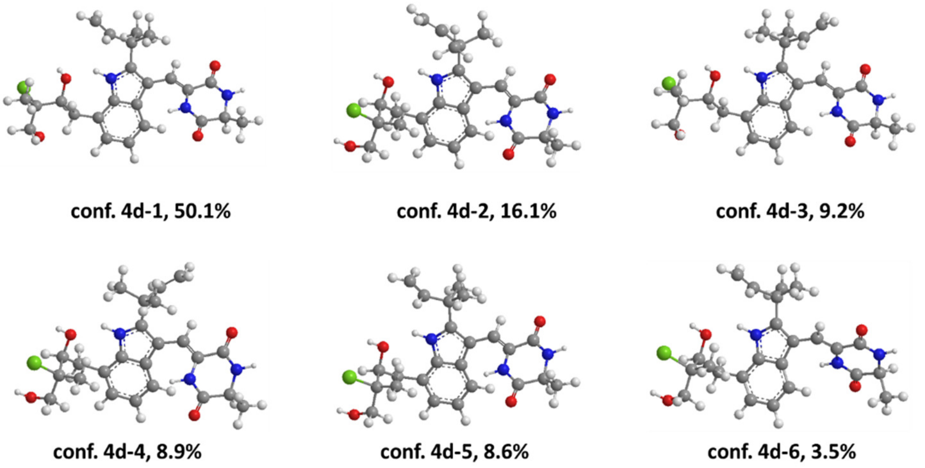

Figure S51. Chiral HPLC analysis of the acidic hydrolysate of compound **5**.

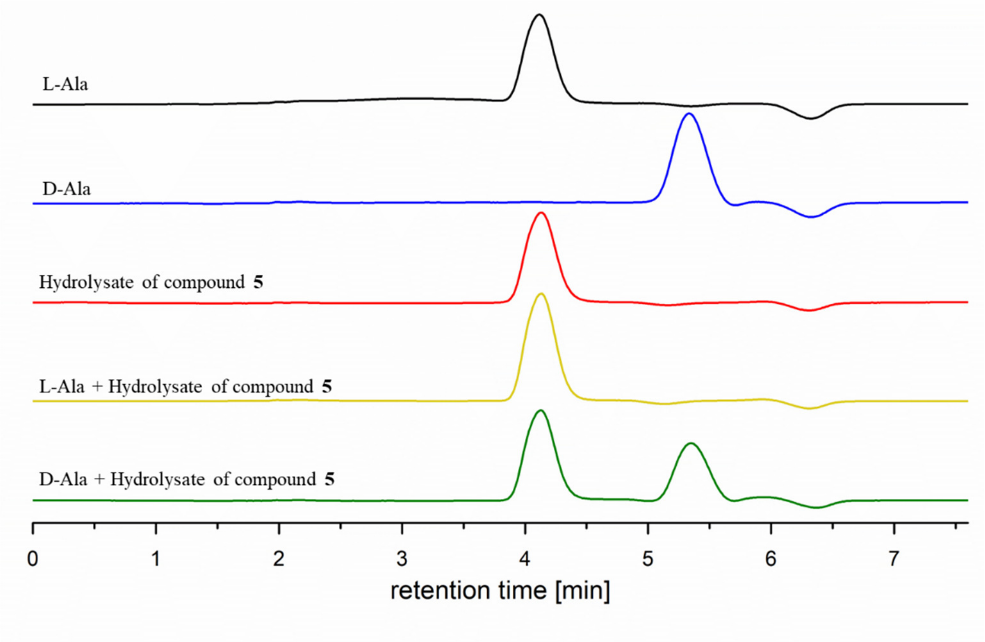

Figure S52. ^{1}H NMR (500 MHz, DMSO-d_6) spectrum of compound **5**.

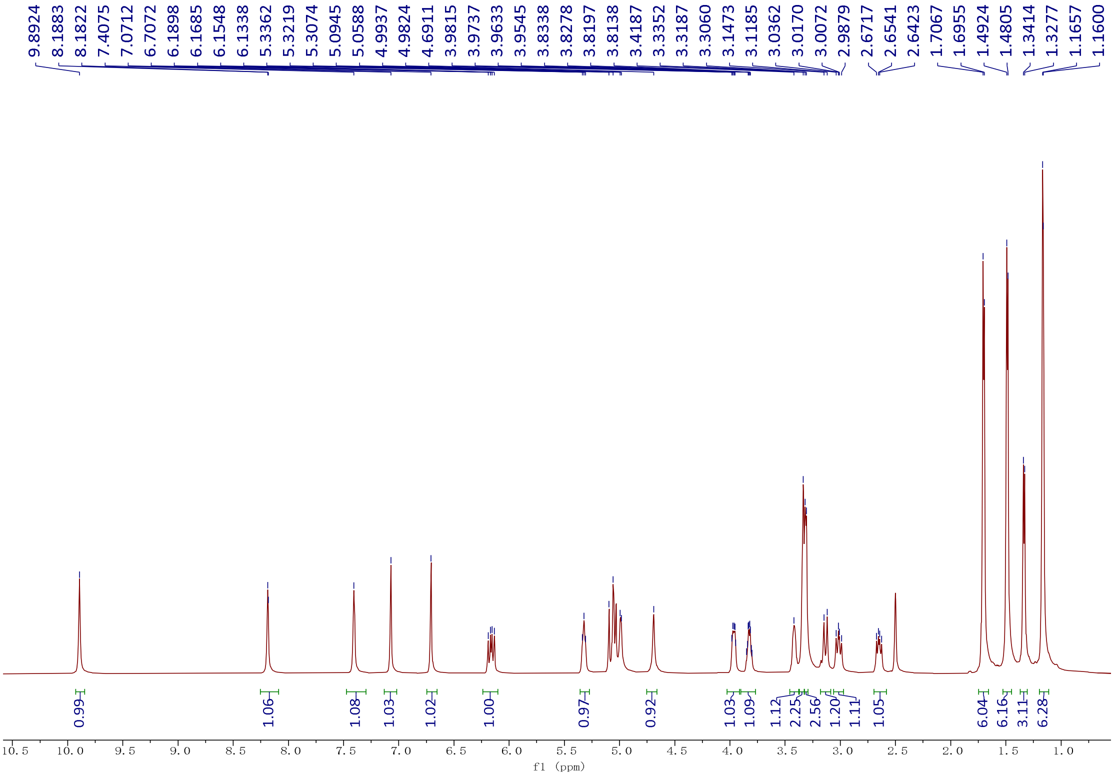

Figure S53. ^{13}C NMR (125 MHz, DMSO-d_6) and DEPT spectra of compound **5**.

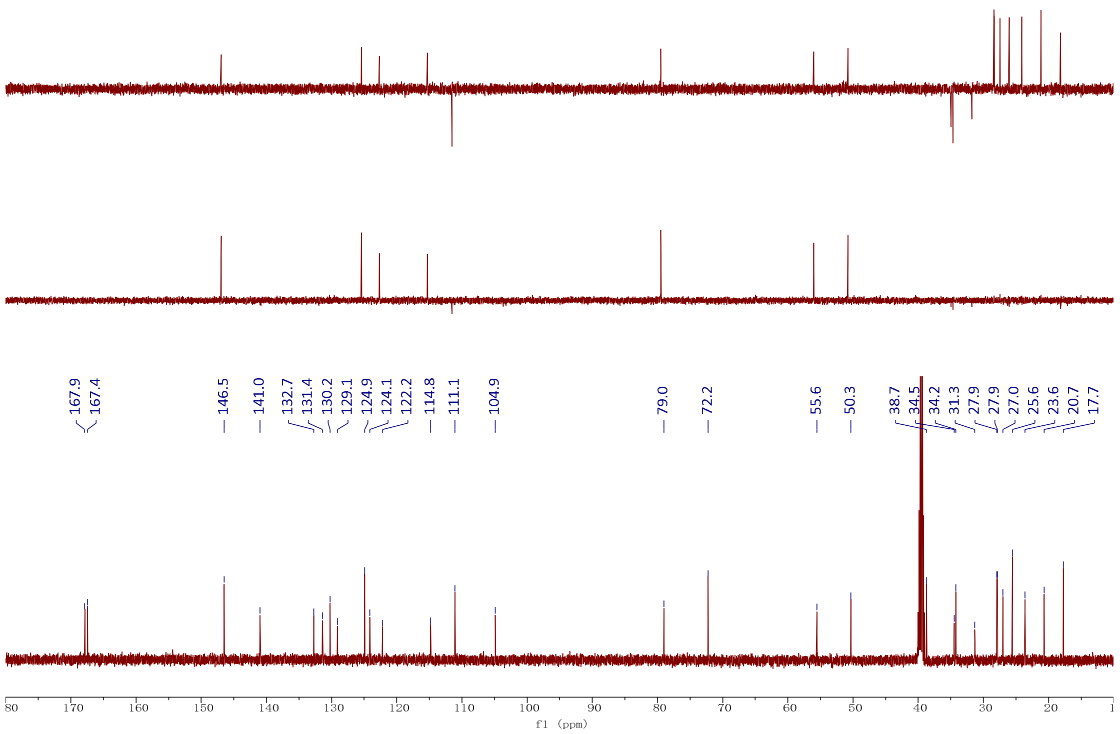

Figure S54. COSY spectrum of compound **5**.

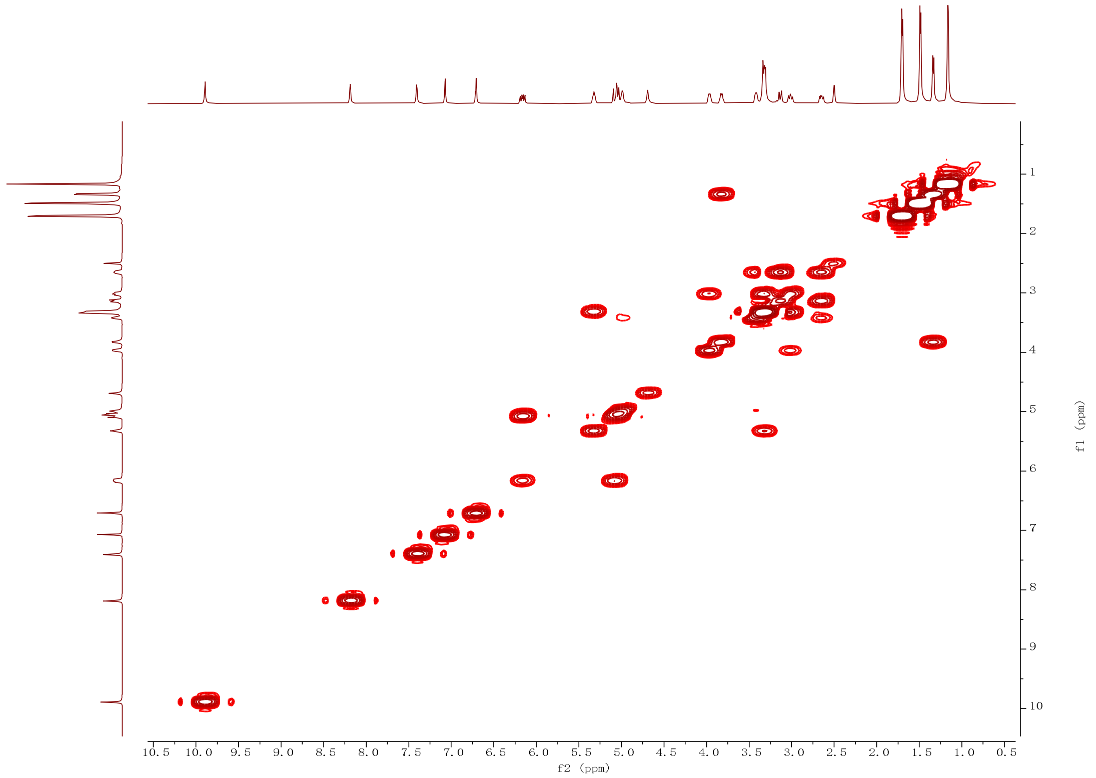

Figure S55. HSQC spectrum of compound **5**.

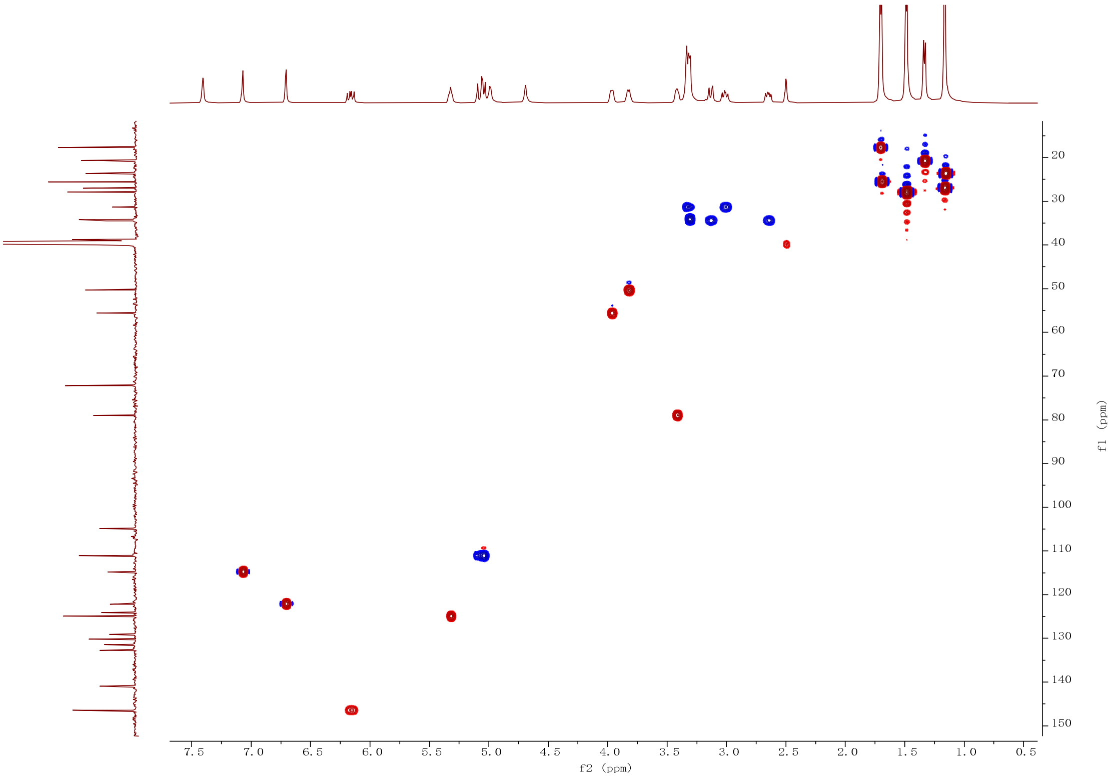

Figure S56. HMBC spectrum of compound **5**.

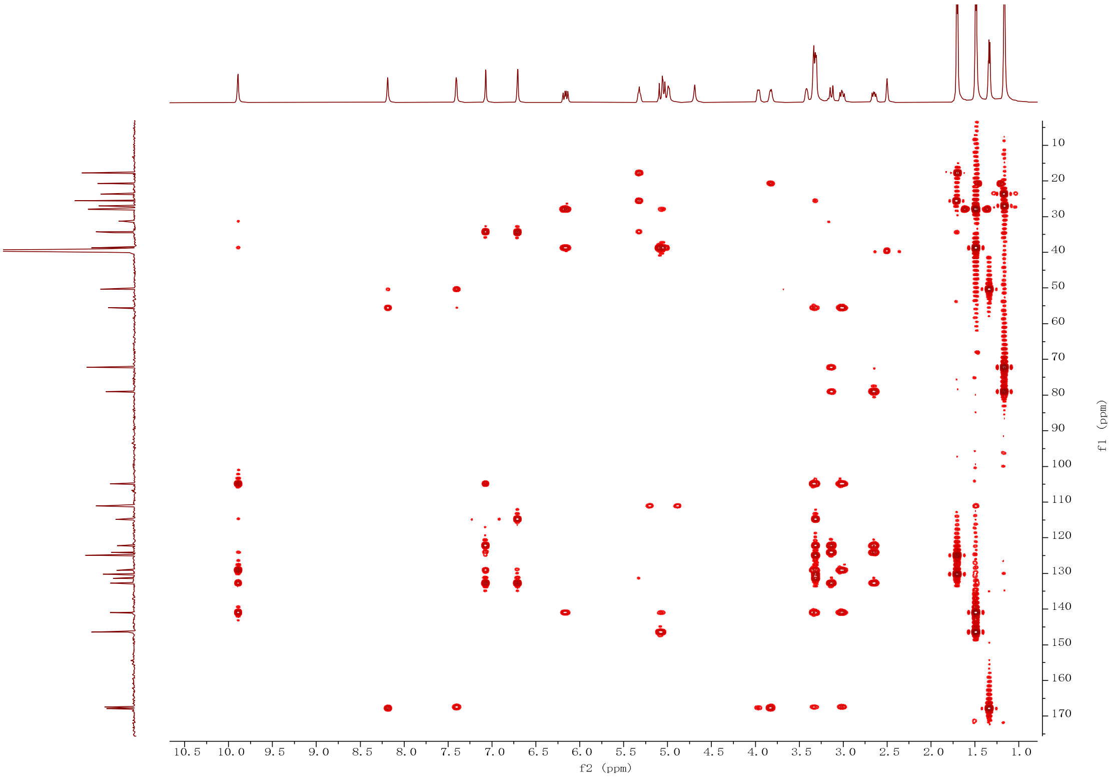

Figure S57. NOESY spectrum of compound **5**.

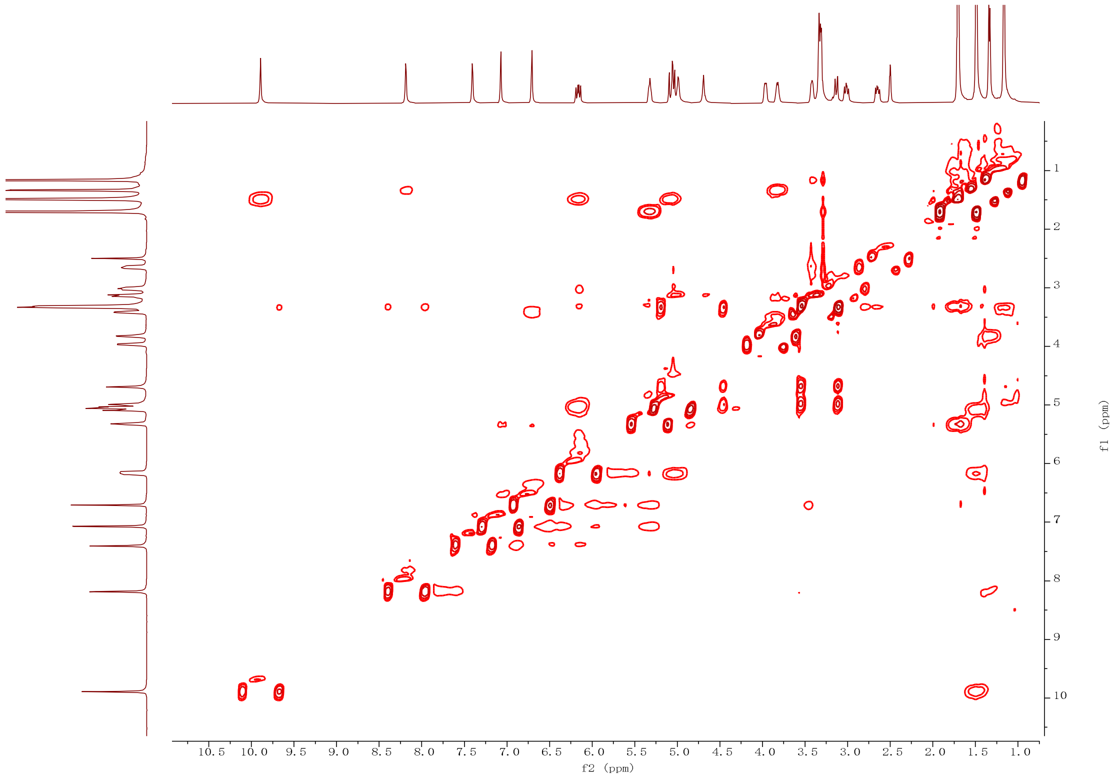

Figure S58. HR-ESI-MS spectrum of compound **5**.

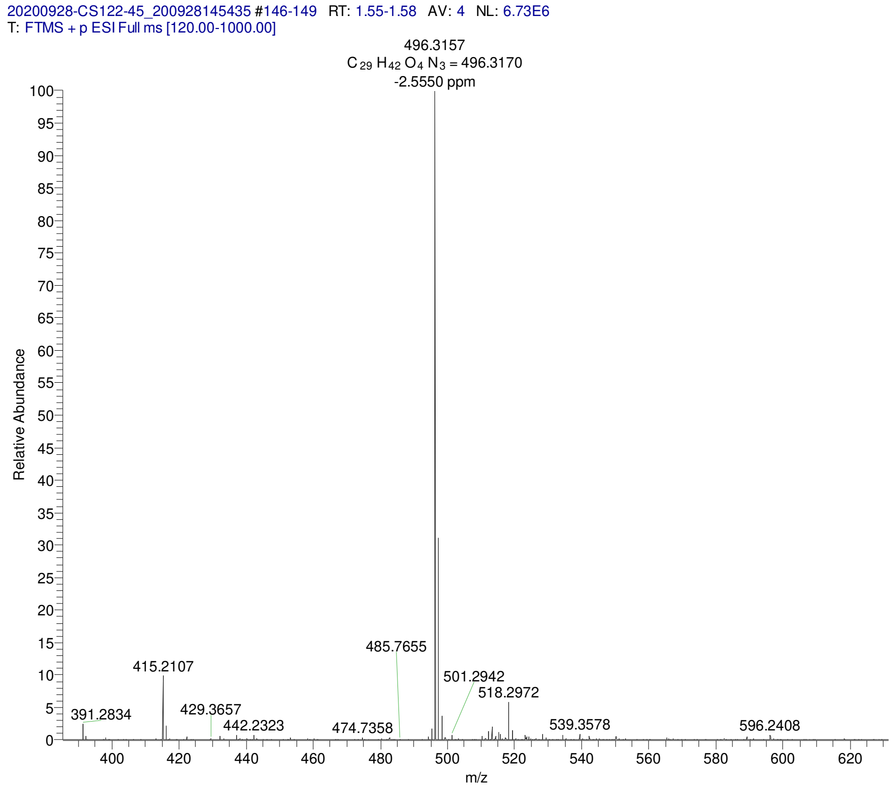

Figure S59. ECD spectrum of compound **5**.

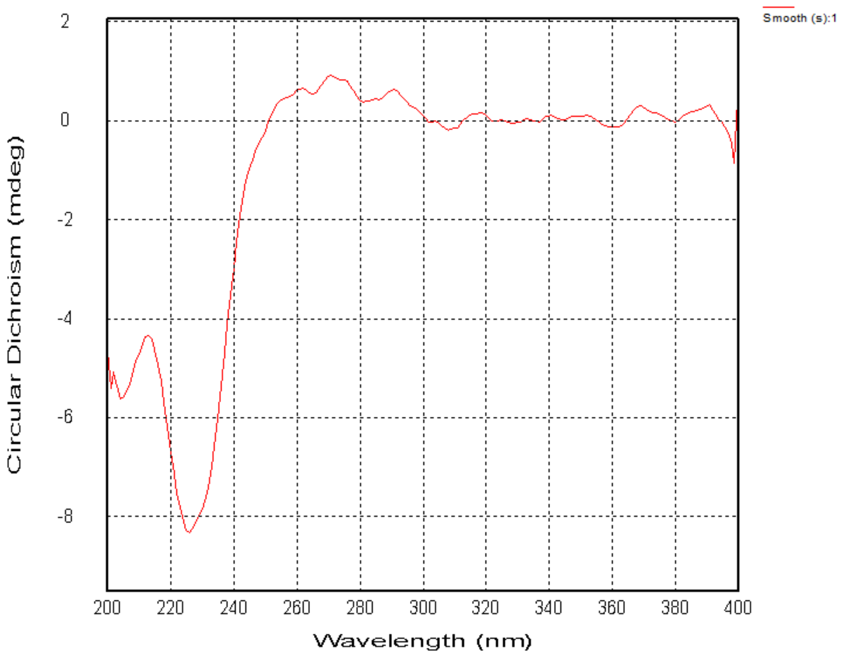

Figure S60. ^{1}H NMR (500 MHz, DMSO-d_6) spectrum (δ_H range from 0.8 to 2.0 ppm) of compound **7** compared with **4**.

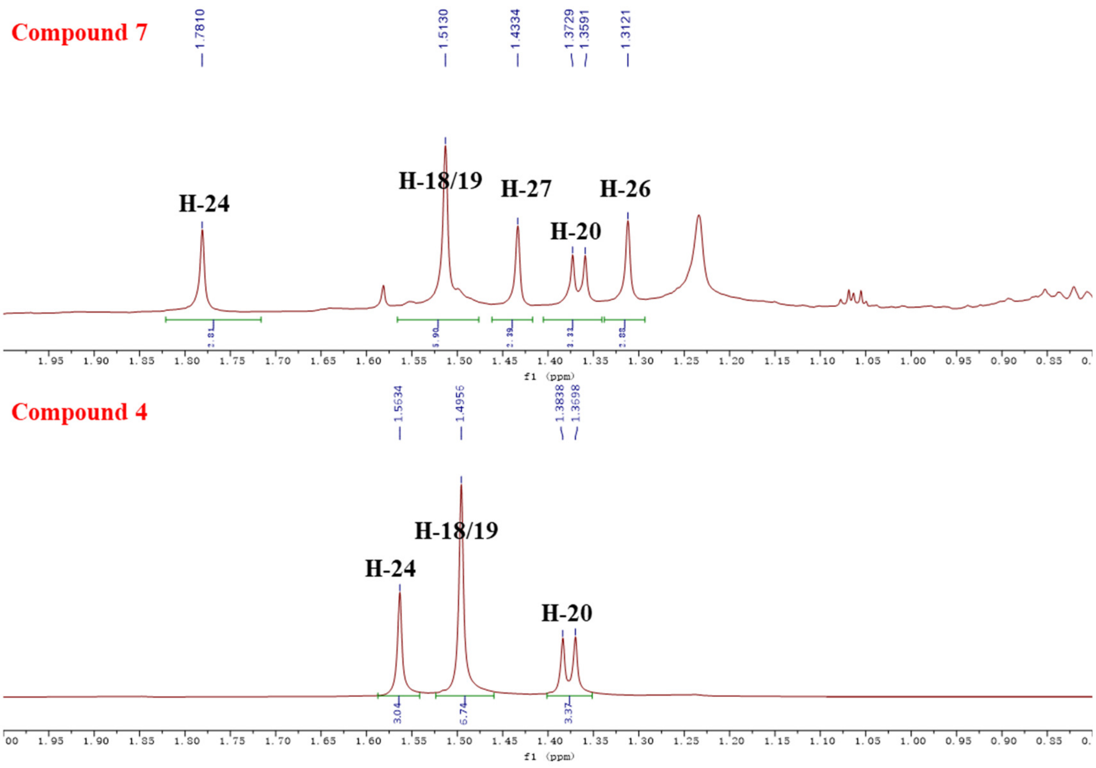

Figure S61. ^{1}H NMR (500 MHz, DMSO-d_6) spectrum (δ_H range from 2.5 to 4.5 ppm) of compound **7** compared with **4**.

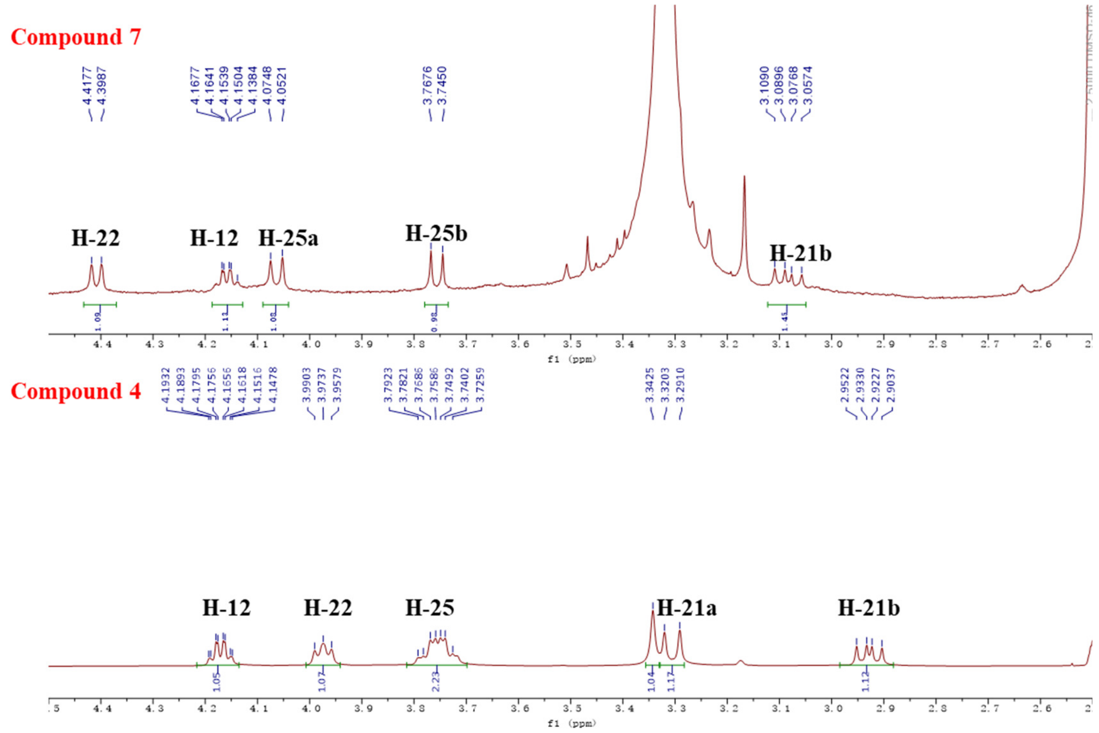

Figure S62. ^{13}C NMR (125 MHz, DMSO-d_6) spectrum (δ_C range from 15.0 to 33.0 ppm) of compound **7** compared with **4**.

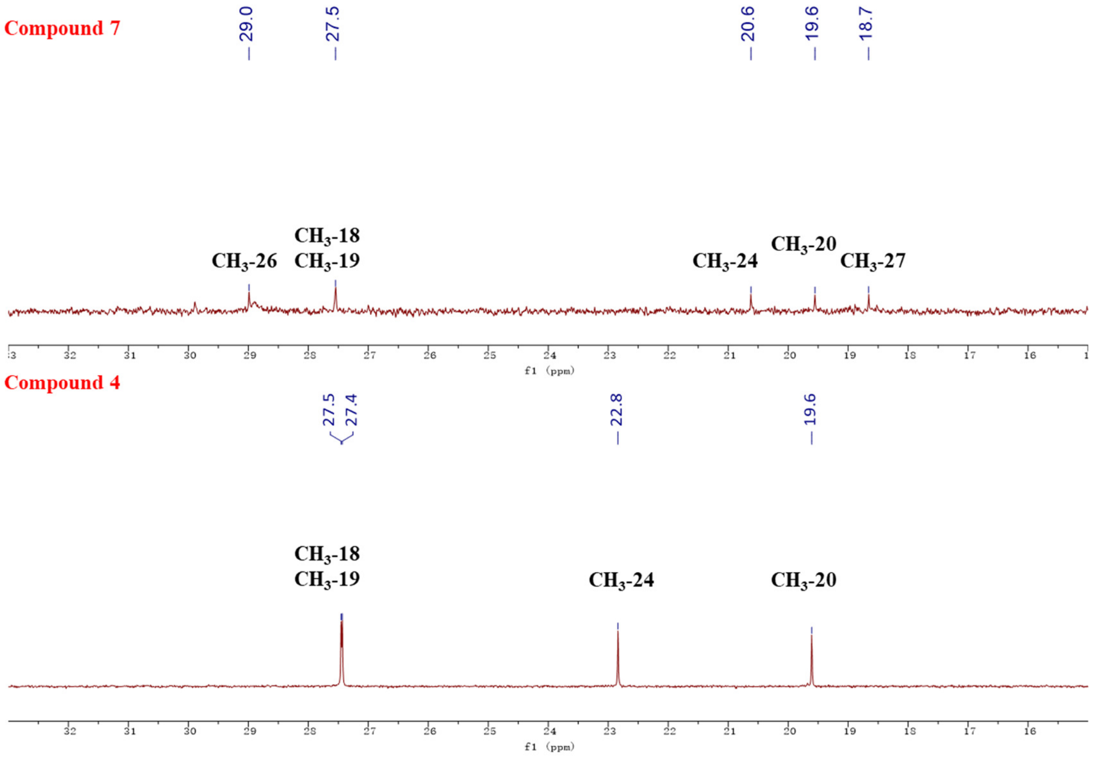

Figure S63. Enlarged HMBC spectrum of compound **7**.

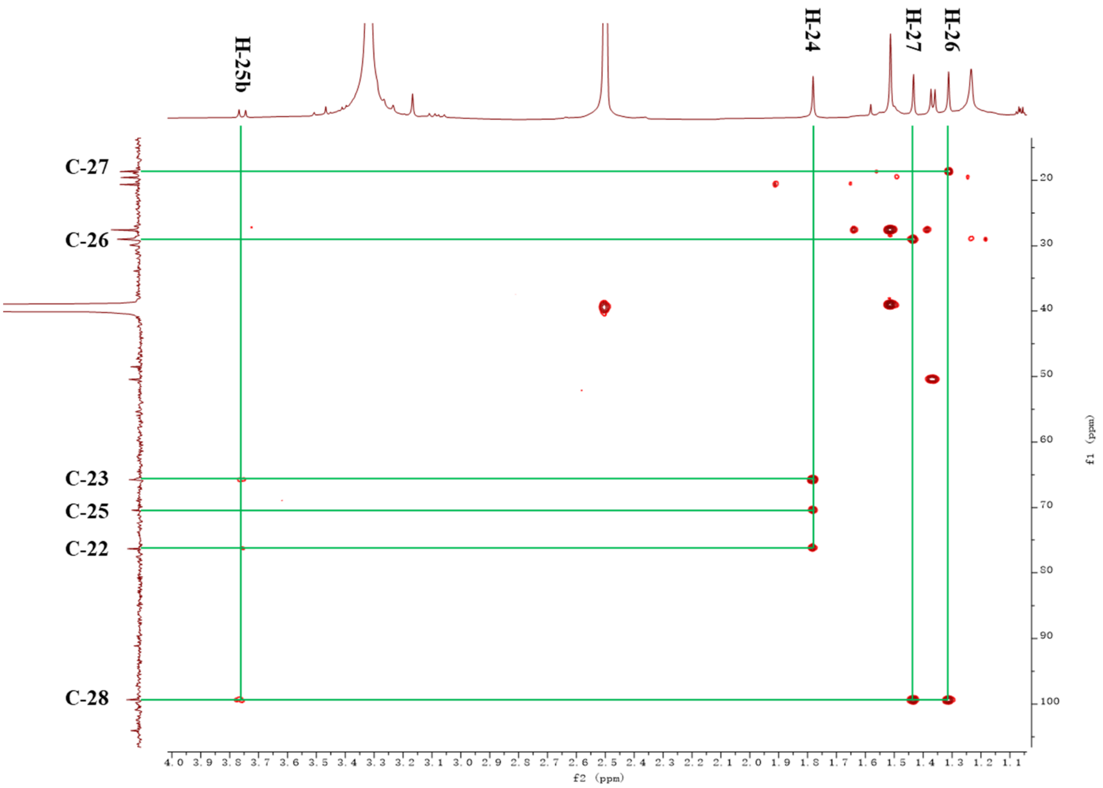

Figure S64. Enlarged NOESY spectrum of compound **7**.

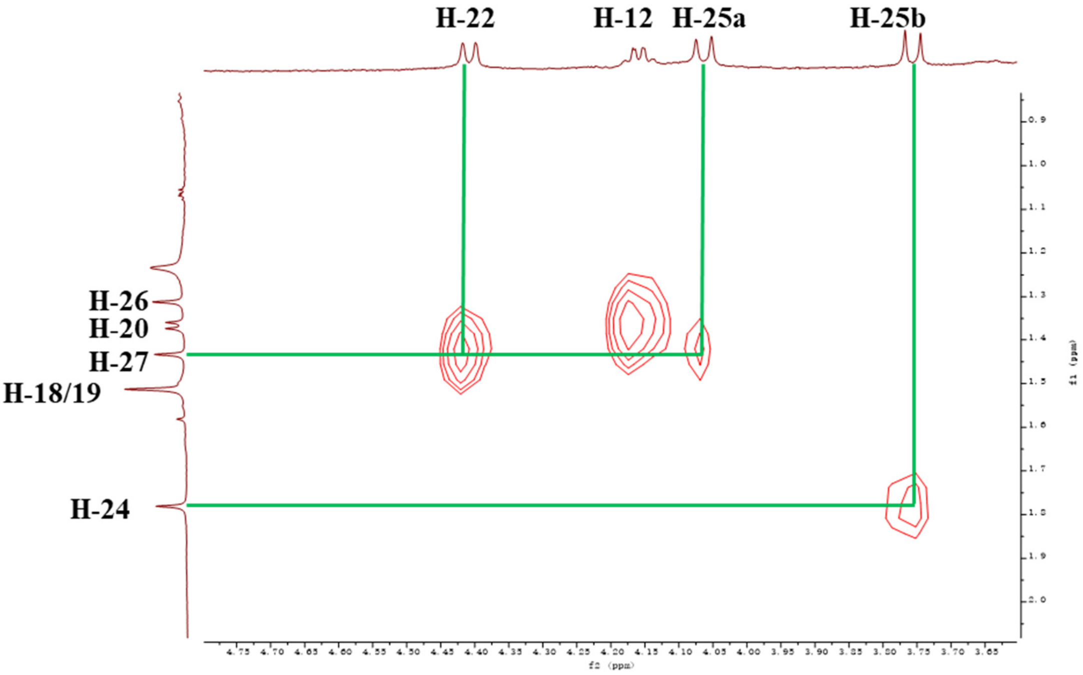

Figure S65. ^{1}H NMR (500 MHz, DMSO-d_6) spectrum of compound **7**.

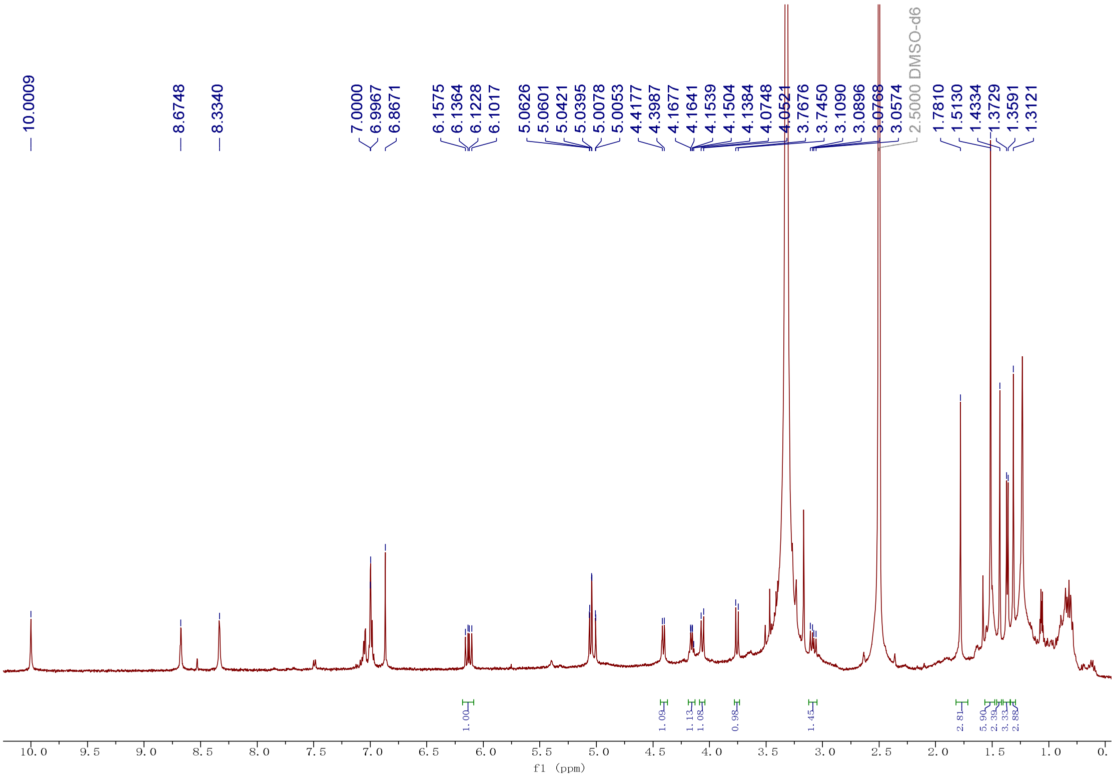

Figure S66. ^{13}C NMR (125 MHz, DMSO-d_6) spectrum of compound **7**.

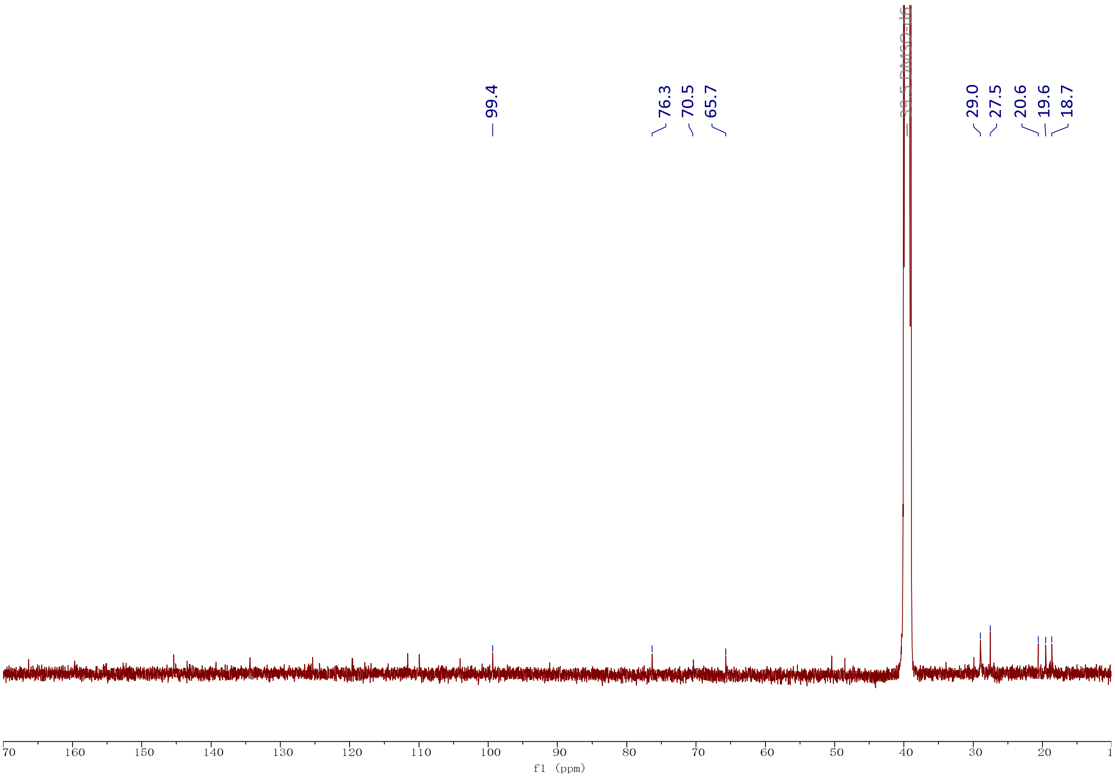

Figure S67. HSQC spectrum of compound **7**.

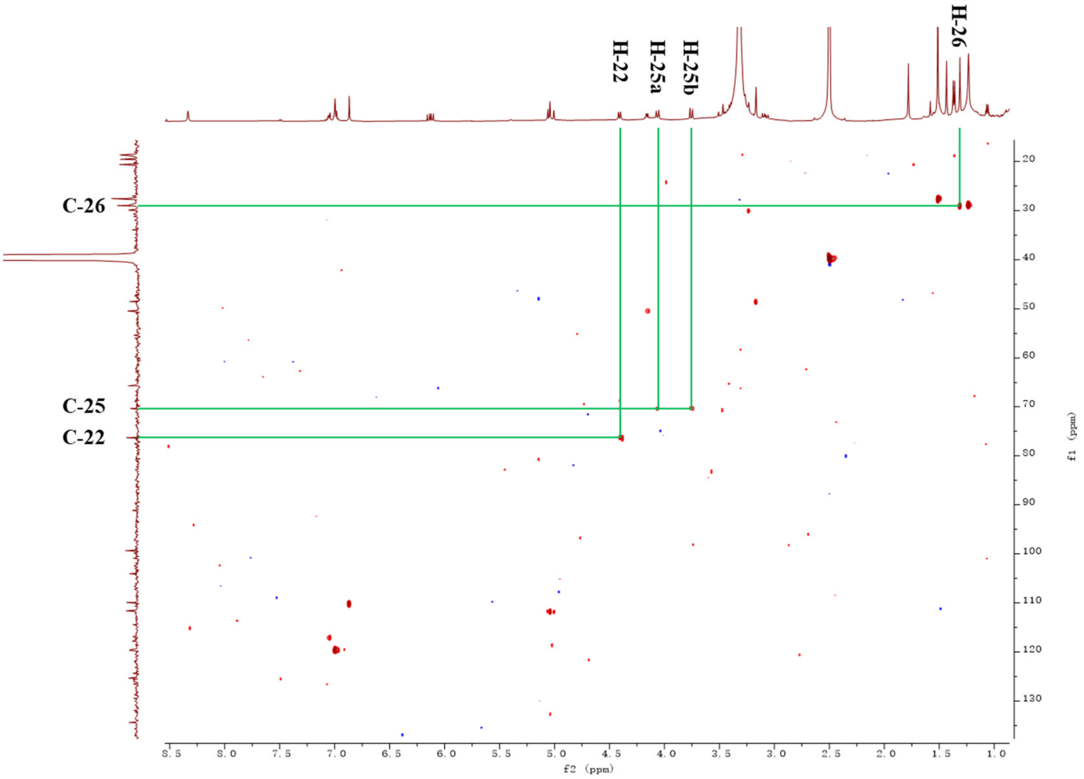

Figure S68. HMBC spectrum of compound **7**.

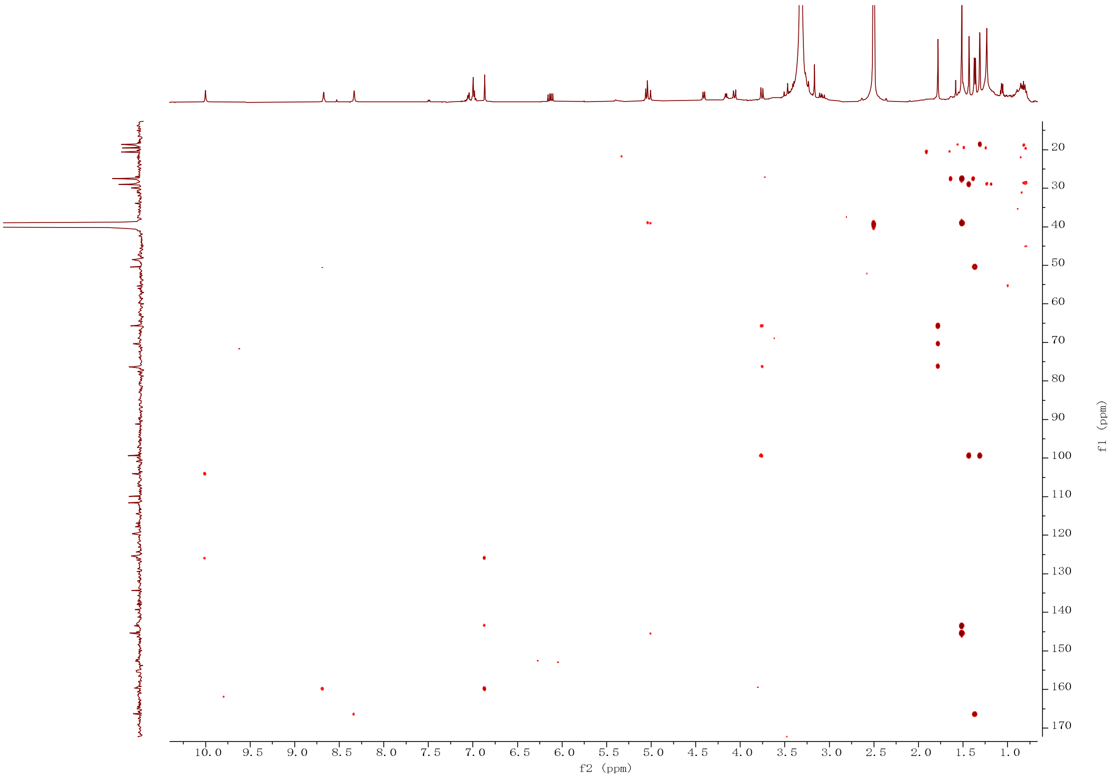

Figure S69. NOESY spectrum of compound **7**.

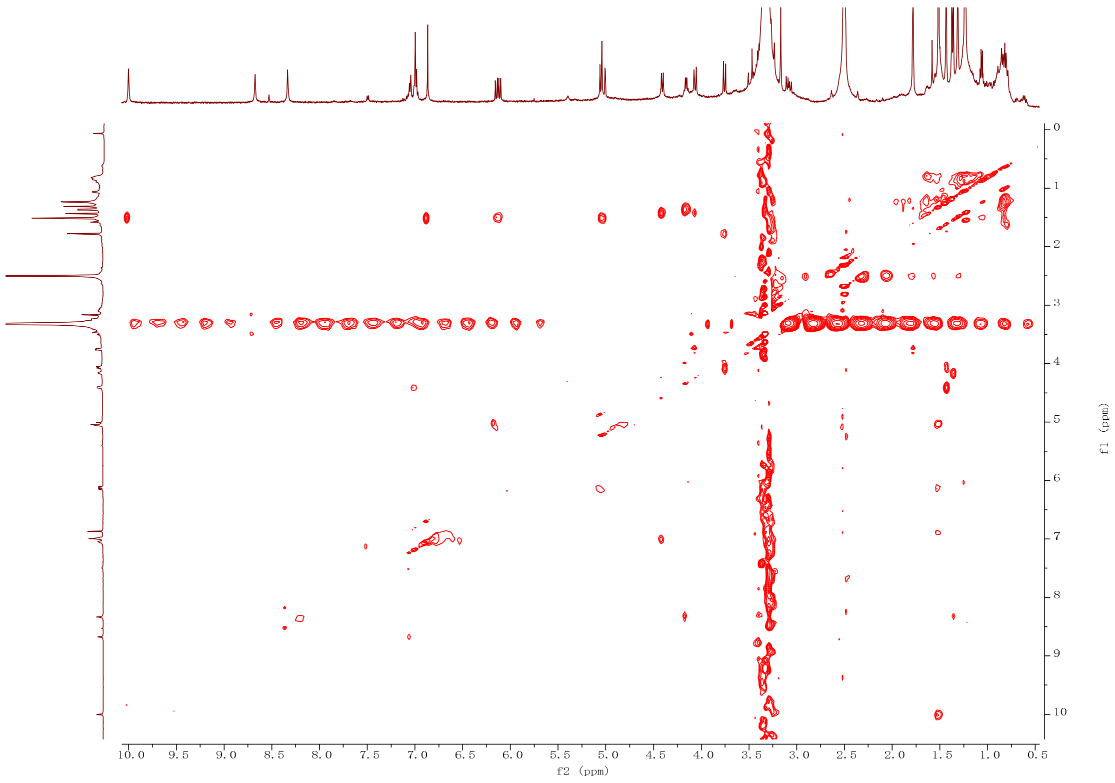

Table S1. DP4+ probability analysis of compound **2** (mPW1PW91/6-31+G(d, p) level).

Functional		Solvent?		Basis Set		Type of Data	
mPW1PW91		PCM		6-31+G(d,p)		Shielding Tensors	
		DP4+	0.00%	0.38%	99.62%	0.00%	-
Nuclei	sp2?	xperimenta	Isomer 1	Isomer 2	Isomer 3	Isomer 4	Isomer 5
C	x	143.5	51.8386636	51.942005	51.96915	51.2497181	
C	x	103.8	90.7204915	92.9918721	91.4732304	89.1753562	
C	x	125.9	71.485432	72.0017629	71.3749833	71.7233817	
C	x	116.8	79.8235558	80.3828952	80.3978348	79.5184769	
C	x	119.6	77.7287279	78.0980488	78.6069989	77.8820111	
C	x	121.7	74.7762016	74.8561883	75.010802	74.9109642	
C	x	125.2	74.3547603	72.6945746	72.113262	73.769242	
C	x	134.3	65.8867993	64.6023888	65.1395048	65.6575543	
C	x	110.2	84.4378042	82.5907408	84.0305683	84.3461953	
C	x	124.8	73.1191796	74.1408626	73.7666533	73.5209691	
C	x	159.9	40.3366818	39.1615132	39.8607704	39.4278454	
C		50.5	140.310587	141.206967	140.184862	140.529603	
C	x	166.3	33.3462972	33.6760387	33.4575845	33.3987039	
C		38.9	151.907812	151.781425	152.055038	152.403753	
C	x	145.1	52.3432353	50.7722723	52.8714561	53.7939735	
C	x	111.7	85.2279852	86.2691147	85.6390444	86.0249954	
C		27.4	169.448557	171.603335	168.968803	169.062849	
C		27.4	168.089329	167.641092	169.247044	168.728237	
C		19.6	174.322475	176.160457	174.335082	174.982002	
C		34	158.260777	158.309472	158.288694	157.684863	
C		74.8	119.790755	117.107817	117.098786	119.264636	
C		74.2	118.135653	118.996386	119.546442	118.269416	
C		19.1	177.805706	173.809046	173.917381	177.920105	
C		67.6	126.67918	131.63757	131.805373	126.60649	
H	x	7.05	23.9152366	23.9025619	24.0299127	23.9131134	
H	x	6.94	24.0265437	24.1085376	24.0899373	24.0481236	
H	x	6.94	24.1129209	24.1872617	24.189517	24.128685	
H	x	6.9	23.9628216	24.0501314	23.9521064	23.9546333	
H		4.16	27.2274609	27.0638161	27.2289109	27.1699612	
H	x	6.09	24.9962902	24.8930568	24.9910864	25.0835124	
H	x	5.06	25.9010196	26.0487052	25.8439787	25.9158772	
H	x	5.05	25.8006602	25.8758917	25.9108965	25.9946065	
H		1.49	29.9240462	29.6821444	30.0254691	29.9539387	
H		1.49	30.2391798	30.3660412	30.1652495	30.2476075	
H		1.49	29.8486395	29.9817704	29.8161691	29.8130073	
H		1.49	29.851849	29.753197	30.2535647	30.2325783	
H		1.49	30.24551	30.0077642	29.824858	29.9755997	
H		1.49	29.7699658	30.0220694	29.8339989	29.7123325	
H		1.37	30.1227543	30.2553353	29.7691759	29.7520675	
H		1.37	30.140022	30.3123509	30.1423697	30.1961131	
H		1.37	29.7578552	29.69268	30.1047857	30.1492898	
H		3.25	28.915272	28.5092628	28.4945056	28.8939923	
H		2.76	28.0662182	28.4408876	28.6948473	28.0248001	
H		3.7	27.130891	27.452943	27.5457912	27.0961171	
H		1.12	30.6733066	29.7512454	29.7301626	30.6207949	
H		1.12	30.5271427	30.5769952	30.6160341	29.9496672	
H		1.12	29.9345811	30.2234885	30.2037262	30.5280683	
H		3.43	27.5801144	28.1272263	27.3830356	27.5863057	
H		3.43	27.6729044	27.8031135	27.7231963	27.5573394	

Table S2. DP4+ probability analysis of compound **3** (mPW1PW91/6-31+G(d, p) level).

Functional		Solvent?		Basis Set		Type of Data	
mPW1PW91		PCM		6-31+G(d,p)		Shielding Tensors	
		DP4+	0.00%	3.00%	95.91%	1.09%	-
Nuclei	sp2?	xperimenta	Isomer 1	Isomer 2	Isomer 3	Isomer 4	Isomer 5
C	x	143.6	51.5097674	51.4078495	51.4186336	52.106997	
C	x	104.2	89.1859653	90.1692001	90.3230941	90.3546859	
C	x	126.1	71.0011924	70.8040828	71.3094814	71.6299773	
C	x	117.3	79.6060935	79.3540908	79.8976343	79.6087482	
C	x	119.5	77.6223736	78.2049235	78.0279965	78.1417713	
C	x	121.3	73.6868433	75.4398996	75.4303318	73.9884158	
C	x	122.6	74.9826265	74.6776057	74.178389	75.6665042	
C	x	133.9	65.5796894	65.3030369	65.2423812	65.6786562	
C	x	110.1	84.9694731	84.2375814	84.0625539	84.9597906	
C	x	125.2	72.4625355	73.9810307	73.3974881	73.0286133	
C	x	159.8	39.8508536	39.4310652	39.6327397	39.4154573	
C		50.6	139.108387	140.884014	140.92155	140.666633	
C	x	166.4	32.902641	33.4465009	33.6469913	33.5156638	
C		39	151.286557	152.174569	151.961203	151.897633	
C	x	145.2	54.0626186	51.246986	52.5916096	53.5450179	
C	x	111.7	84.9005454	85.3882208	85.027459	85.2144998	
C		27.6	168.739737	168.347337	169.030072	169.394297	
C		27.5	166.726261	169.880367	168.494833	167.11384	
C		19.8	172.883515	175.543575	175.594278	175.299109	
C		33.8	155.952963	156.154338	156.599329	156.613256	
C		66.9	114.891393	119.60513	119.814601	115.326356	
C		74.2	117.18842	117.848366	117.834332	117.694844	
C		21	176.45628	178.916054	179.093942	177.143724	
C		67.5	125.169721	126.4838	126.302857	125.737449	
H	x	7.09	23.7942208	23.941482	23.9116187	23.8933589	
H	x	6.99	23.9370573	24.0196821	24.0311279	24.0560603	
H	x	7	23.9599206	24.1266244	24.0794407	24.110067	
H	x	6.88	23.8028517	23.9904471	23.9294121	23.9040008	
H		4.14	27.170209	27.1125181	27.1278105	27.1453788	
H	x	6.11	25.0220641	24.9368016	24.9819495	25.0348375	
H	x	5.04	25.859267	25.9437073	25.8252368	25.9004831	
H	x	5.05	25.7829311	25.8322744	25.8938267	25.9612471	
H		1.5	29.7256622	29.8389564	29.8129145	29.9038653	
H		1.5	30.1122629	29.8532851	30.2593649	30.1836405	
H		1.5	29.7951421	30.2569389	29.8597624	29.8021514	
H		1.5	29.8240103	29.8950565	30.2248515	30.2658449	
H		1.5	30.0995611	29.8675952	29.9541856	29.8902197	
H		1.5	29.5900842	30.1971066	29.9012188	29.7322133	
H		1.39	29.9140073	30.201391	29.7123011	29.7303786	
H		1.39	29.8806364	30.2619261	30.2507909	30.2212625	
H		1.39	29.7274469	29.7005133	30.1985254	30.18095	
H		3.15	28.5097383	28.6254049	28.6652944	28.6111085	
H		3.67	27.8016374	27.4594928	27.5045139	27.9053828	
H		4.47	26.5102713	27.5953712	27.6350966	26.5920852	
H		1.28	30.2872501	29.8149178	29.8216038	30.5614589	
H		1.28	29.7813227	30.1713484	30.1901816	29.9081806	
H		1.28	30.4444082	30.1822099	30.2106445	30.4118784	
H		3.57	27.3170547	27.54234	28.0016173	27.506387	
H		3.57	27.4199914	27.9952557	27.5257023	27.4218249	

Table S3. DP4+ probability analysis of compound **4** (mPW1PW91/6-31+G(d, p) level).

Functional		Solvent?		Basis Set		Type of Data	
mPW1PW91		PCM		6-31+G(d,p)		Shielding Tensors	
		DP4+	0.00%	0.86%	99.14%	0.00%	-
**Nuclei**	**sp2?**	**Experimental**	**Isomer 1**	**Isomer 2**	**Isomer 3**	**Isomer 4**	**Isomer 5**
C	x	143.5	51.4165954	52.4250528	51.9382329	51.0305761	
C	x	104	91.8812043	93.1759889	91.4575456	91.6799047	
C	x	126	71.170289	72.0638738	71.6118975	71.8979525	
C	x	117	79.9252163	79.9522164	80.0848993	79.5240436	
C	x	119.7	77.7766143	78.4244751	77.8284114	78.5067977	
C	x	121.9	74.1597627	74.6581274	74.870796	74.4356763	
C	x	123.8	73.0634901	72.6354693	72.6786032	73.8514978	
C	x	134.2	65.0382498	65.1353141	65.2278177	64.7790458	
C	x	110	83.1105597	84.3615966	84.3829949	85.2005312	
C	x	125.1	74.70987	72.6450862	74.0119729	74.0871338	
C	x	159.8	38.6051629	39.4468951	40.503148	38.701521	
C		50.5	141.184375	141.116074	139.459195	141.194031	
C	x	166.4	33.5231571	33.6797135	33.2742286	33.4661147	
C		38.9	152.058389	151.860883	151.99495	151.880139	
C	x	145.1	50.3229216	53.2174309	52.6304222	52.2405177	
C	x	111.8	86.3786563	84.5745504	85.3864848	84.3942213	
C		27.5	170.549411	167.524369	170.047465	171.397753	
C		27.4	169.256937	170.109166	168.651561	166.409951	
C		19.6	176.372265	176.173227	173.020451	176.522768	
C		34.2	158.632902	157.843881	157.762331	157.914792	
C		77.7	117.426459	113.295216	112.980437	117.979865	
C		74	97.2871928	97.3723827	97.3264919	97.4551892	
C		68.1	125.232148	129.532416	129.495255	125.237926	
C		22.8	174.806695	171.038073	170.925806	175.158699	
H	x	7.08	23.8466493	23.909666	23.9214107	23.889799	
H	x	6.98	24.0814297	24.0715408	24.0685306	24.0435268	
H	x	7	24.1488858	24.1549762	23.9859259	24.1734131	
H	x	6.89	24.0980849	23.9190373	24.1407665	23.9308788	
H		4.17	27.0462414	27.0534916	27.3558786	26.9991985	
H	x	6.1	24.9040707	24.9519575	24.970643	24.7315339	
H	x	5.06	26.0493939	25.8604939	25.9511391	25.8011929	
H	x	5.07	25.8463578	25.8305083	25.8636533	25.8189205	
H		1.5	29.707401	29.9654586	29.9811657	29.6678443	
H		1.5	30.0734824	30.1379693	30.1976843	30.1627381	
H		1.5	30.0787128	29.889605	29.8358771	29.9983024	
H		1.5	29.665238	29.8731393	29.8878713	30.2792243	
H		1.5	30.3094958	30.3032673	30.2202591	29.7489677	
H		1.5	29.8819208	29.8242639	29.8105649	29.945821	
H		1.38	30.2439099	30.2326211	30.0119762	29.6787193	
H		1.38	30.3234202	30.2959491	30.0260651	30.3379072	
H		1.38	29.689387	29.6716511	29.8289341	30.2526726	
H		2.93	28.3851685	28.4255317	28.5399329	28.4371255	
H		3.31	28.1893116	28.5601528	28.4370926	28.3334925	
H		3.97	26.8639115	27.3385841	27.3273843	26.9571858	
H		3.74	27.3671639	27.7113912	27.1619442	27.6160339	
H		3.75	27.6233343	27.1718937	27.7229194	27.4053032	
H		1.56	30.4838942	29.1988593	29.1957794	30.4463717	
H		1.56	30.9414039	30.0027325	29.8836677	29.6441782	
H		1.56	29.7045103	29.8902255	29.9852188	30.7289172	

Table S4. The calculated shielding tensors of each conformer (>2%) for isomer **2a**.

no. C	calcd. shileding tensors of C for each conformer							
	**2a-1**	**2a-2**	**2a-3**	**2a-4**	**2a-5**	**2a-6**	**2a-7**	**2a-8**
2	51.9468	53.6952	51.2111	51.4672	50.2789	53.275	50.0626	50.0542
3	92.7492	93.5077	88.2359	86.5681	91.465	91.5601	88.7655	88.7626
3a	72.5496	71.119	72.3796	69.3762	73.2043	71.3219	69.5705	69.572
4	79.4799	80.2963	80.254	79.5771	79.5889	79.1669	79.8968	79.8905
5	77.0766	77.8336	77.2037	78.6136	77.6038	78.0424	78.5049	78.5012
6	74.6311	74.649	74.9709	74.7571	74.9918	74.4446	75.0395	75.0421
7	74.9774	74.6491	73.3548	74.1198	73.8846	75.2051	74.3241	74.3203
7a	67.0847	65.3518	65.4231	65.841	65.3449	65.7257	65.8955	65.8916
8	84.9448	83.662	85.5817	84.5219	81.8602	86.5385	84.8234	84.8141
9	73.6988	71.6177	72.6061	73.8561	74.0571	71.8672	74.4919	74.4908
10	41.4141	40.9395	40.4664	38.2505	41.3722	39.7031	38.561	38.554
12	139.4526	141.0848	139.133	141.4667	139.3183	141.2407	141.4094	141.4126
13	33.3181	33.8245	32.8569	33.143	33.3686	33.6041	33.28	33.2768
15	152.0741	150.4212	152.5496	152.2847	152.3201	151.5974	152.903	152.9032
16	52.3114	50.9652	55.4978	57.0284	48.5202	50.0093	49.2225	49.2228
17	82.3835	85.1775	86.1474	84.989	87.0654	86.55	87.7571	87.7588
18	172.6073	167.5766	169.8312	167.9276	167.3544	167.3592	171.7289	171.7303
19	165.634	167.9008	167.1978	169.9065	171.9455	169.3732	167.5424	167.541
20	172.4491	175.1084	172.434	176.898	172.5946	177.0106	176.6145	176.6265
21	159.146	159.0676	157.7115	157.9651	157.1	157.456	157.6369	157.6412
22	121.2344	119.449	119.3315	119.4972	119.0581	119.7768	119.3068	119.3028
23	117.868	118.0574	118.2915	118.3018	118.1293	118.5068	118.2066	118.2096
24	177.5928	177.831	178.0356	177.7481	177.9844	177.8464	177.7094	177.7092
25	127.168	126.6971	126.6796	126.2731	126.3597	126.9624	126.3326	126.3342

no. H	calcd. shileding tensors of H for each conformer							
	**2a-1**	**2a-2**	**2a-3**	**2a-4**	**2a-5**	**2a-6**	**2a-7**	**2a-8**
4	23.7727	23.9751	23.8393	24.0698	23.8354	23.965	24.0704	24.0704
5	23.9793	24.0097	24.0485	24.0484	24.0173	24.0789	24.084	24.084
6	24.0828	24.1217	24.1034	24.1467	24.0642	24.1339	24.1738	24.1738
8	23.8741	23.9578	23.8895	23.9917	24.1091	23.87	24.1367	24.1364
12	27.3163	27.2455	27.3898	26.9769	27.3381	27.103	27.0314	27.0315
16	24.8937	24.9245	25.2457	25.1126	24.9705	25.1457	24.749	24.7488
17	25.6322	25.8601	26.0152	25.8504	26.1395	26.0837	26.1057	26.1056
17	25.7122	25.6495	25.8908	25.8918	25.9038	25.8054	25.9298	25.9299
18	29.5907	30.2301	29.8312	30.251	30.2761	30.0257	29.1925	29.1924
18	30.4616	30.1077	30.2876	30.1621	29.9631	30.0488	30.4982	30.4985
18	30.2333	29.7706	29.6458	29.7455	29.7446	29.6577	29.8625	29.8626
19	30.069	29.7826	29.8017	29.5854	30.1038	29.7209	29.7634	29.7636
19	30.0477	30.4208	30.1605	30.3666	30.5704	30.4066	29.7855	29.7852

19	29.6203	29.6296	30.262	29.7667	29.4026	29.7513	30.0716	30.0718
20	29.9971	30.2254	29.9858	30.2593	29.9871	30.248	30.2935	30.2931
20	29.9652	30.241	29.9642	30.3412	29.9627	30.3642	30.3861	30.386
20	29.8143	29.7017	29.8484	29.6749	29.8458	29.6408	29.6919	29.692
21	28.9172	28.906	28.992	28.86	28.9502	28.8999	28.8548	28.8552
21	28.164	28.1559	27.9888	27.9879	27.9799	28.105	27.9661	27.9664
22	27.1848	27.2049	27.0564	27.1212	27.0396	27.1277	27.0899	27.0907
24	30.7056	30.6951	30.6502	30.6429	30.6922	30.6621	30.618	30.618
24	30.5137	30.5222	30.5444	30.5195	30.5365	30.5636	30.5192	30.5193
24	29.9019	29.9431	29.946	29.9372	29.9536	29.9425	29.9408	29.9407
25	27.6617	27.5894	27.5265	27.5571	27.5596	27.5732	27.5229	27.5228
25	27.7861	27.717	27.5447	27.6587	27.6356	27.6361	27.6131	27.6128

Table S5. The calculated shielding tensors of each conformer (>2%) for isomer **2b**.

no. C	calcd. shileding tensors of C for each conformer						
	**2b-1**	**2b-2**	**2b-3**	**2b-4**	**2b-5**	**2b-6**	**2b-7**
2	51.4005	53.4764	53.1376	51.233	52.2517	52.2489	52.2149
3	93.2999	92.4854	91.5884	92.7973	93.3348	93.3305	93.523
3a	71.8472	71.7773	72.6953	71.8956	73.0956	73.0942	72.3218
4	80.3954	80.4313	80.1867	80.9246	80.2438	80.2448	80.0513
5	78.1947	77.6473	77.9752	78.1162	78.2108	78.2102	78.3918
6	75.3559	74.1377	73.9644	74.1607	73.8634	73.8637	73.991
7	71.8294	73.8614	73.9664	74.0958	75.0994	75.0978	73.8772
7a	64.9693	64.3221	63.2477	64.6228	63.8673	63.8653	64.7245
8	81.5137	84.5224	84.4832	85.0853	83.8649	83.8684	83.1967
9	74.9293	73.5865	71.1125	74.2567	71.3328	71.329	76.8435
10	38.5865	40.715	40.5059	38.9727	39.4413	39.4389	38.5905
12	141.4873	140.3862	140.5895	141.2748	141.1468	141.1476	141.5013
13	33.5849	33.786	34.0736	33.4777	33.917	33.9163	33.5872
15	151.9142	151.8555	150.8227	152.2737	151.2734	151.2745	152.2052
16	49.7173	54.1184	51.9524	51.6704	51.439	51.4363	49.8815
17	87.5986	82.3859	85.2933	83.2246	84.8579	84.8562	87.989
18	172.5504	172.5403	169.2769	165.3042	167.7965	167.7954	172.8233
19	167.8528	163.6659	168.8567	172.3036	168.9202	168.9186	168.3796
20	176.6731	174.3737	175.0366	176.5784	176.3686	176.3749	176.9842
21	157.8459	159.8378	159.5209	157.5538	157.7737	157.7744	158.6234
22	117.0865	117.4068	116.6916	116.8225	117.5413	117.5416	117.3338
23	119.2484	118.8399	118.7083	118.2603	118.225	118.2268	118.0171
24	174.2777	173.0761	173.1497	172.8995	172.8813	172.8819	172.9033
25	131.9105	130.751	130.9169	131.8695	131.7487	131.7463	131.9709

no. H	calcd. shileding tensors of H for each conformer						
	**2b-1**	**2b-2**	**2b-3**	**2b-4**	**2b-5**	**2b-6**	**2b-7**
4	23.9014	23.9092	23.9495	23.9009	23.8644	23.8646	23.7762
5	24.1242	24.1053	24.1214	24.0248	24.0327	24.0327	24.0183
6	24.1554	24.2724	24.2829	24.1868	24.155	24.1549	24.1612
8	24.0936	24.0086	24.0145	23.9516	23.7934	23.7935	24.1099
12	27.0053	27.1936	27.2787	26.9979	27.0429	27.0428	27.0329
16	24.9652	24.7163	24.7401	24.7296	24.9172	24.9172	24.9798
17	26.1942	25.7187	25.8273	25.6906	25.9008	25.9008	26.2229
17	25.9338	25.767	25.8032	25.6909	25.721	25.7209	26.0411
18	29.6126	29.5005	30.3582	29.7121	29.5833	29.5831	29.7632
18	30.4156	30.484	30.0226	30.1536	30.3184	30.3185	30.3836
18	29.9439	30.1676	30.0324	30.0991	29.8085	29.8086	29.8471
19	29.6047	30.1746	29.8701	30.0774	29.9293	29.9287	29.5782
19	29.8623	30.2162	30.3342	30.4905	30.1052	30.1053	29.9682
19	30.1889	29.7121	29.4285	29.6446	30.2608	30.2606	30.2832
20	30.2854	30.159	30.2001	30.2632	30.2633	30.2636	30.257
20	30.3599	30.1238	30.2413	30.336	30.353	30.3533	30.3556
20	29.6951	29.6991	29.7187	29.6456	29.6585	29.6586	29.6422
21	28.4508	28.5939	28.6117	28.6153	28.6438	28.644	28.5496
21	28.7486	27.8857	27.9369	27.983	28.0341	28.0343	27.8819
22	27.542	27.2902	27.3188	27.3052	27.356	27.3558	27.2343
24	29.7014	29.8017	29.8129	29.8847	29.8886	29.8888	29.8594
24	30.6104	30.5162	30.5389	30.5059	30.5225	30.5226	30.5047
24	30.2026	30.2136	30.2462	30.3118	30.3275	30.3277	30.283
25	27.6903	28.7046	28.7386	29.0489	29.0765	29.0764	29.0805
25	27.3757	28.487	28.4864	28.5152	28.5715	28.5715	28.4756

Table S6. The calculated shielding tensors of each conformer (>2%) for isomer **2c**.

no. C	calcd. shileding tensors of C for each conformer							
	**2c-1**	**2c-2**	**2c-3**	**2c-4**	**2c-5**	**2c-6**	**2c-7**	**2c-8**
2	52.1424	52.2495	52.5739	51.3513	52.4005	51.2141	51.2142	50.897
3	90.6843	93.8282	89.721	93.6284	91.7678	90.5926	90.5912	88.6252
3a	70.743	71.9117	72.5414	72.4371	70.2074	71.4397	71.4377	69.0592
4	80.3107	80.7891	80.6546	80.4262	79.679	80.1416	80.1429	80.33
5	79.2844	78.4643	77.8424	77.9555	79.7751	77.8854	77.8845	79.2221
6	74.9091	74.9475	75.3143	75.1189	74.3468	75.3347	75.3341	74.9872
7	71.595	72.3776	71.6678	72.4523	72.2542	72.5091	72.5094	72.643
7a	66.0549	64.5313	64.666	65.4619	65.2122	64.474	64.474	65.6881
8	83.7559	84.4061	84.7446	83.3399	86.3152	82.3085	82.3053	83.6246
9	74.0768	73.2488	72.9398	74.3633	71.4952	75.297	75.2993	75.3444
10	37.7245	41.6908	40.2228	40.86	39.6384	40.4494	40.4431	38.3502
12	141.5712	139.7822	139.0862	139.1987	140.992	139.1707	139.1704	141.4609

13	33.3652	33.9427	32.839	33.4548	33.7922	33.3114	33.309	33.4461
15	151.8289	152.1437	152.0576	151.6701	152.0153	152.1966	152.195	152.9691
16	56.2918	50.6786	56.158	52.2362	51.3546	49.6843	49.6836	49.6759
17	85.3243	85.7625	85.8368	83.6195	84.4636	87.1342	87.136	87.5833
18	170.3907	168.1915	167.8415	165.2761	168.6462	172.3506	172.3497	167.7909
19	168.2713	168.6099	170.3674	172.8397	167.4309	167.5411	167.54	173.223
20	177.0808	172.5179	172.6759	172.8083	176.3974	172.8191	172.8181	176.7329
21	158.9267	157.7524	157.9534	157.9729	158.9366	157.9841	157.9827	158.7107
22	117.0598	117.0616	116.9918	116.946	117.5725	117.0716	117.0689	117.2457
23	119.5237	119.6584	119.4347	119.8863	119.6583	119.167	119.1668	119.6418
24	173.9375	173.7166	173.9764	173.658	173.7745	174.4458	174.4453	173.8575
25	131.7013	131.7872	131.8975	131.8227	131.7479	131.9455	131.9442	131.8005

no. H	calcd. shileding tensors of H for each conformer							
	**2c-1**	**2c-2**	**2c-3**	**2c-4**	**2c-5**	**2c-6**	**2c-7**	**2c-8**
4	24.1103	24.0451	23.888	23.9227	24.1968	23.9432	23.9433	24.1218
5	24.0785	24.0812	24.0751	24.0883	24.115	24.1012	24.1011	24.1409
6	24.1675	24.2089	24.1549	24.1992	24.236	24.1889	24.1888	24.2079
8	23.9138	23.9381	23.9392	23.8399	23.8725	24.1049	24.1049	24.1375
12	27.021	27.2794	27.4025	27.3936	27.0337	27.4335	27.4338	27.0172
16	25.1626	24.8035	25.2409	24.804	24.8768	24.9588	24.9588	24.9192
17	25.8962	25.7718	25.9754	25.7597	25.6518	25.8973	25.8974	25.8766
17	25.848	25.8483	26.02	25.6989	25.7555	26.1835	26.1835	26.0749
18	30.2434	30.3373	29.8849	29.5876	29.4867	30.1815	30.1815	29.5805
18	30.1073	30.0944	30.2224	30.474	30.3122	29.8788	29.879	30.4204
18	29.8368	29.9946	29.5506	30.1074	29.7831	29.656	29.6561	29.6894
19	30.407	30.3391	30.1409	30.0355	30.0361	30.417	30.4169	29.9408
19	29.7369	29.4817	30.2418	29.6746	30.4236	29.6231	29.6227	30.1646
19	29.7449	29.7843	29.8253	30.0594	29.9541	29.9929	29.9931	29.4906
20	29.6669	29.808	29.8243	29.8437	29.6663	29.8644	29.8645	29.7207
20	30.3658	30.0235	29.9634	29.9704	30.3495	29.9972	29.997	30.4077
20	30.2842	30.0248	29.9724	29.9559	30.2523	29.9764	29.976	30.308
21	28.4447	28.5629	28.4732	28.5798	28.4645	28.4703	28.4706	28.4616
21	28.6254	28.7532	28.6803	28.7307	28.7191	28.7424	28.7427	28.5985
22	27.5242	27.5541	27.5101	27.5723	27.6114	27.5742	27.574	27.4857
24	29.7175	29.7527	29.7165	29.7595	29.7461	29.7068	29.7069	29.7148
24	30.6184	30.619	30.609	30.6241	30.619	30.6167	30.6169	30.5947
24	30.2026	30.2022	30.1881	30.2192	30.2328	30.2041	30.2042	30.1828
25	27.3402	27.4359	27.3864	27.4255	27.3368	27.3918	27.3917	27.3381
25	27.7055	27.7563	27.7118	27.7664	27.7096	27.6999	27.6999	27.7063

Table S7. The calculated shielding tensors of each conformer (>2%) for isomer **2d**.

no. C	calcd. shileding tensors of C for each conformer						
	**2d-1**	**2d-2**	**2d-3**	**2d-4**	**2d-5**	**2d-6**	**2d-7**
2	51.6209	51.6279	50.18	50.1843	50.6076	50.6016	51.6279
3	90.6728	84.39	93.1045	93.1198	89.2544	89.2538	84.39
3a	73.1711	69.4677	73.2933	73.2978	70.4987	70.4937	69.4677
4	79.2293	79.635	79.601	79.6058	79.7818	79.7833	79.635
5	77.3481	78.3903	77.8093	77.8113	78.4168	78.415	78.3903
6	74.8389	74.8673	74.9221	74.9212	75.118	75.1227	74.8673
7	73.4424	74.2976	73.5976	73.599	74.4078	74.4012	74.2976
7a	65.6499	65.7781	65.3516	65.3525	65.9273	65.9234	65.7781
8	84.9854	85.4419	81.076	81.0722	85.0884	85.0857	85.4419
9	72.5101	73.7507	73.8304	73.8269	74.9076	74.9044	73.7507
10	38.6118	40.2409	39.4371	39.4326	40.2302	40.224	40.2409
12	141.5011	139.1316	141.3683	141.3666	139.5728	139.5717	139.1316
13	33.244	33.3618	33.644	33.6381	33.6642	33.6634	33.3618
15	152.5051	152.2205	152.1055	152.1058	153.0827	153.0824	152.2205
16	55.2774	57.4436	48.7696	48.7686	49.1846	49.1831	57.4436
17	85.6578	84.7706	87.44	87.4423	87.5925	87.5933	84.7706
18	170.1248	167.6962	166.9578	166.9564	171.5034	171.5065	167.6962
19	166.6202	170.21	171.52	171.5158	167.4351	167.434	170.21
20	177.0735	172.3185	176.386	176.377	172.5399	172.5352	172.3185
21	158.028	157.7652	157.2084	157.2041	157.5459	157.5469	157.7652
22	119.5698	119.4077	119.1909	119.1883	119.1876	119.181	119.4077
23	118.1234	118.3669	118.189	118.1892	118.2997	118.2997	118.3669
24	177.9581	177.7248	178.0781	178.079	177.7183	177.718	177.7248
25	126.6871	126.3829	126.576	126.5747	126.4664	126.4655	126.3829

no. H	calcd. shileding tensors of H for each conformer						
	**2d-1**	**2d-2**	**2d-3**	**2d-4**	**2d-5**	**2d-6**	**2d-7**
4	23.7799	24.0734	23.8523	23.8526	24.0269	24.027	24.0734
5	24.0321	24.0582	24.0385	24.0385	24.0553	24.0554	24.0582
6	24.1079	24.1542	24.0833	24.0835	24.151	24.151	24.1542
8	23.8274	23.9815	24.0446	24.0448	24.1144	24.1142	23.9815
12	27.0159	27.3762	27.0106	27.0109	27.3815	27.3826	27.3762
16	25.2277	25.1126	24.951	24.9511	24.7897	24.7897	25.1126
17	25.906	25.9603	25.882	25.8816	25.8935	25.8938	25.9603
17	25.9615	25.8465	26.1833	26.184	26.1142	26.1146	25.8465
18	30.2984	29.7656	29.4132	29.4132	30.0978	30.098	29.7656
18	30.1903	30.3449	30.5257	30.5256	29.8224	29.822	30.3449
18	29.8596	29.5883	30.1163	30.1168	29.8007	29.8006	29.5883
19	30.3218	30.1755	29.9453	29.9452	30.531	30.531	30.1755
19	29.8726	30.2647	30.2688	30.2691	29.2071	29.207	30.2647

19	29.645	29.7444	29.6595	29.66	29.9132	29.9128	29.7444
20	29.6414	29.8834	29.6705	29.6702	29.9076	29.9085	29.8834
20	30.3498	29.9625	30.3373	30.3369	30.0396	30.0392	29.9625
20	30.2679	29.9581	30.2684	30.2684	30.0487	30.0484	29.9581
21	28.9566	28.8617	28.9851	28.9849	28.8591	28.8593	28.8617
21	27.991	27.9797	27.9705	27.9701	27.9688	27.9691	27.9797
22	27.0656	27.0952	27.0232	27.023	27.0858	27.0873	27.0952
24	30.6527	30.6427	30.6833	30.6831	30.6258	30.6258	30.6427
24	29.9484	29.9345	29.9638	29.9637	29.9428	29.9429	29.9345
24	30.5365	30.5259	30.5306	30.5306	30.5329	30.5328	30.5259
25	27.5549	27.6399	27.5882	27.5884	27.6016	27.6013	27.6399
25	27.523	27.5497	27.5434	27.5432	27.5264	27.5262	27.5497

Table S8. The calculated shielding tensors of each conformer (>2%) for isomer **3a**.

no. C	calcd. shileding tensors of C for each conformer					
	**3a-1**	**3a-2**	**3a-3**	**3a-4**	**3a-5**	**3a-6**
2	52.08	52.0831	50.6424	51.78	52.8248	49.9961
3	88.8656	88.8689	92.2731	86.2564	92.4005	91.9162
3a	71.7676	71.7675	71.3147	69.0942	71.889	71.8292
4	79.9688	79.9724	80.4153	79.6546	79.2332	80.1507
5	77.656	77.6579	77.7638	78.7178	78.5788	77.935
6	74.0863	74.0883	73.7651	73.8413	73.918	74.2414
7	74.641	74.6399	75.587	75.7479	78.0002	74.8717
7a	65.7649	65.7615	66.3048	66.2706	64.9629	65.74
8	85.4699	85.469	84.6037	86.2146	86.646	82.2225
9	72.27	72.2655	73.1846	73.6391	72.3167	74.0959
10	40.3053	40.301	40.794	38.2077	39.4315	40.8764
12	139.0974	139.097	139.2315	141.4319	141.1842	139.2404
13	32.8731	32.8769	33.1328	33.0568	33.6579	33.1769
15	151.9838	151.984	152.2042	151.739	151.1975	152.0852
16	55.7643	55.7696	51.6205	57.4519	49.7498	48.6064
17	85.858	85.8551	82.1305	84.3639	86.8666	87.6527
18	170.128	170.1285	172.2701	166.9036	166.6537	167.2848
19	166.3853	166.3844	165.2619	169.867	169.2425	172.0173
20	172.4848	172.4805	172.4713	176.8663	177.0492	172.6773
21	156.578	156.5793	156.9448	156.659	156.2642	156.308
22	115.5413	115.5417	114.3026	115.6614	115.6463	115.4967
23	117.6026	117.6024	118.0509	117.7258	117.4941	117.4917
24	177.1636	177.1622	177.2155	177.1728	177.3598	177.1352
25	125.3611	125.3648	126.9581	125.6253	125.9525	125.0294

no. H	calcd. shileding tensors of H for each conformer					
	**3a-1**	**3a-2**	**3a-3**	**3a-4**	**3a-5**	**3a-6**
4	23.8513	23.8516	23.8741	24.0356	23.9297	23.8806
5	24.0275	24.0279	24.0438	24.061	24.0433	24.022
6	24.0252	24.0255	24.1042	24.0926	24.148	24.0198
8	23.9042	23.9045	23.849	23.8749	23.8621	24.1087
12	27.3761	27.3768	27.3541	26.976	27.0845	27.3709
16	25.2467	25.2464	24.8896	25.0628	25.1455	24.8924
17	26.0463	26.0465	25.6911	25.8235	26.0736	26.1729
17	25.9531	25.9536	25.663	25.9716	25.7804	25.9036
18	29.7674	29.7669	30.2193	29.6769	29.7784	30.0814
18	30.163	30.1633	30.1323	30.3249	30.4468	30.5449
18	30.2344	30.2344	29.5089	29.6772	29.724	29.3277
19	29.9463	29.946	29.5534	30.2024	30.0126	30.2034
19	30.2503	30.2503	30.4669	30.1341	30.0626	29.9068
19	29.5661	29.5658	30.2054	29.7269	29.6303	29.7579
20	29.9795	29.9592	29.9578	30.2642	30.2362	29.9701
20	29.9592	29.834	29.8236	30.3587	30.3598	29.9615
20	29.8334	29.9792	29.9724	29.6795	29.6348	29.8344
21	28.6161	28.6162	28.7228	28.5764	28.6107	28.6389
21	27.9159	27.9164	28.1138	27.7128	28.0046	27.8145
22	26.6431	26.6439	26.7025	26.4607	26.6459	26.5825
24	30.4131	30.4129	30.481	30.3652	30.3849	30.4023
24	29.8721	29.8717	29.9299	29.9824	29.9157	29.9082
24	30.5661	30.566	30.5922	30.5393	30.5955	30.5943
25	27.4046	27.4044	27.5546	27.4232	27.392	27.4086
25	27.5292	27.5288	27.5218	27.5038	27.601	27.5737

Table S9. The calculated shielding tensors of each conformer (>2%) for isomer **3b**.

no. C	calcd. shileding tensors of C for each conformer							
	**3b-1**	**3b-2**	**3b-3**	**3b-4**	**3b-5**	**3b-6**	**3b-7**	**3b-8**
2	50.7364	52.2733	51.8057	52.057	51.5219	51.5157	50.4491	50.449
3	91.1115	90.7636	85.7884	92.8305	88.6581	88.652	91.5759	91.5768
3a	71.0541	70.6687	69.4406	71.3359	69.1776	69.1783	73.0177	73.0166
4	79.3216	78.5623	78.635	80.7013	78.8676	78.8613	80.2113	80.2156
5	78.1424	78.3617	79.0345	78.0332	78.0788	78.0811	77.8391	77.8394
6	75.6414	75.0974	75.1152	75.2866	75.3221	75.3258	75.9964	75.9947
7	74.5422	75.7137	74.1806	74.0131	75.4625	75.4655	73.7679	73.7659
7a	65.4619	64.9855	65.6751	65.0761	65.904	65.9022	64.6919	64.6904
8	85.7835	86.4168	84.0307	83.8252	83.1092	83.1027	82.5994	82.6005
9	74.4837	72.2	74.0947	72.2278	75.3133	75.3183	74.686	74.6844
10	38.5295	39.4671	38.2855	40.9856	38.5278	38.5314	40.9239	40.9174
12	141.2731	141.3	141.4172	140.9244	141.3821	141.3861	139.2621	139.261

13	33.3154	33.6897	33.3075	33.8934	33.3839	33.3875	33.2195	33.2175
15	151.8263	151.9319	152.4786	151.4446	152.4928	152.4899	152.6199	152.6179
16	51.6237	50.8282	56.4514	50.9369	49.4554	49.4571	50.1205	50.1207
17	83.2461	84.4944	85.3933	84.9559	87.0227	87.0203	86.5406	86.5411
18	164.3951	168.1318	168.3584	168.1275	172.736	172.7356	167.3365	167.3388
19	172.3802	167.7865	169.8729	168.478	167.6996	167.6965	172.8116	172.8096
20	176.4472	176.7986	176.7427	174.6957	176.3974	176.4112	172.4783	172.4779
21	155.9399	155.6848	156.1591	156.7306	155.9079	155.9046	156.6556	156.6569
22	119.4997	119.4095	119.6442	120.0153	119.6404	119.6421	119.5419	119.5444
23	118.2663	117.8901	117.4446	118.253	117.5954	117.5872	117.7238	117.7263
24	178.6997	178.798	178.701	179.351	178.8314	178.8276	179.1632	179.1661
25	126.4256	126.4082	126.8266	126.1402	126.6094	126.6123	126.4641	126.4611

no. H	calcd. shileding tensors of H for each conformer							
	**3b-1**	**3b-2**	**3b-3**	**3b-4**	**3b-5**	**3b-6**	**3b-7**	**3b-8**
4	23.8885	24.0077	24.066	23.9744	24.0211	24.0211	23.7419	23.7424
5	24.029	24.0143	24.015	24.0504	24.0114	24.0115	24.0087	24.0085
6	24.181	24.1884	24.1534	24.0558	24.1951	24.1954	23.9775	23.9773
8	23.8541	23.8576	24.0062	23.9161	24.1398	24.14	24.1007	24.1006
12	26.984	27.0728	26.9858	27.2228	27.0209	27.0201	27.3718	27.3721
16	24.7695	24.8448	25.14	24.889	24.9861	24.9853	25.0119	25.0121
17	25.732	25.7918	25.879	25.8593	26.1599	26.1594	26.1218	26.122
17	25.7108	25.7052	25.8427	25.7655	25.8961	25.8967	26.0166	26.0163
18	30.0671	30.004	29.615	29.8231	29.5594	29.5599	29.9524	29.9529
18	29.4357	30.3955	29.7489	29.4749	30.1305	30.1296	29.7498	29.75
18	30.5181	30.0116	30.3644	30.4274	29.9366	29.9365	30.4161	30.4162
19	29.7287	29.4543	30.2615	30.3177	29.5977	29.5969	30.251	30.2509
19	30.1126	29.7613	29.799	30.0368	29.8856	29.8857	29.6611	29.6611
19	30.1062	30.4259	30.1279	30.0672	30.4427	30.4424	29.9344	29.9343
20	30.2616	30.2446	30.2679	30.1968	30.2789	30.279	29.9857	29.986
20	30.3617	30.3466	30.3425	30.2032	30.3713	30.3714	29.9656	29.966
20	29.6367	29.633	29.6859	29.6971	29.6703	29.6705	29.8604	29.8606
21	28.6687	28.6608	28.5185	28.7023	28.5478	28.5474	28.6605	28.6609
21	27.5002	27.46	27.3333	27.598	27.3513	27.3509	27.5341	27.5343
22	27.706	27.6211	27.5713	27.6835	27.4279	27.427	27.6179	27.6193
24	29.8592	29.8399	29.7518	29.8438	29.7718	29.7717	29.8231	29.8233
24	30.1835	30.1762	30.0775	30.2512	30.1091	30.1089	30.2336	30.2338
24	30.2149	30.1966	30.1379	30.235	30.145	30.1446	30.1763	30.1765
25	27.5732	27.5578	27.5293	27.5233	27.5202	27.5205	27.5462	27.5462
25	28.0794	28.0242	27.9354	28.039	27.9055	27.9049	28.0046	28.005

Table S10. The calculated shielding tensors of each conformer (>2%) for isomer **3c**.

no. C	calcd. shileding tensors of C for each conformer							
	**3c-1**	**3c-2**	**3c-3**	**3c-4**	**3c-5**	**3c-6**	**3c-7**	**3c-8**
2	51.4376	51.2199	50.7802	52.0254	52.7463	50.4582	50.9579	52.1124
3	92.5549	88.3401	92.7247	84.2034	91.1759	91.0928	91.7509	89.5596
3a	71.7568	72.0985	71.8021	69.6463	70.733	72.6254	70.9604	69.1078
4	80.3524	79.8979	80.6065	78.8958	79.2401	80.0076	79.6436	79.6064
5	78.0001	77.6965	77.4913	78.7682	78.359	77.9339	78.1538	78.2663
6	75.3344	75.5007	75.5357	75.2677	75.0923	75.861	75.6082	75.371
7	74.1227	73.3004	73.718	74.7591	75.5121	73.3269	74.627	75.3323
7a	64.9475	65.2521	65.447	65.4605	64.9618	64.9884	65.5363	65.8502
8	83.3495	85.0719	83.3083	84.7911	86.1071	81.7305	85.3985	83.0453
9	72.2432	72.7571	73.8337	74.5337	71.3578	74.1122	76.1097	74.9071
10	39.8886	38.5822	39.5025	39.9844	40.4394	38.9449	39.6199	40.8375
12	141.4089	141.5821	141.3827	139.1856	140.8919	141.5827	139.4774	140.5924
13	33.9148	33.1744	33.7006	33.3802	34.0481	33.5898	33.5203	33.9342
15	151.3177	152.7446	151.3086	152.6279	152.065	152.3488	151.5753	152.3372
16	50.8881	55.7636	52.6774	56.8472	50.8291	50.0649	51.6562	49.3129
17	84.945	85.5975	83.4822	85.2306	84.5317	86.5519	83.3883	87.5499
18	168.1403	169.8795	172.6632	168.221	168.2783	167.4377	164.3101	172.7583
19	168.3417	167.325	164.859	170.0193	167.7342	172.7395	172.4313	167.7592
20	176.5496	177.1441	176.6065	172.346	175.4354	176.8031	172.4292	174.4017
21	156.8024	157.0948	157.024	156.0379	155.6814	156.9745	156.2517	155.8653
22	119.9863	119.8683	119.8038	119.7723	119.4616	119.5194	120.0176	119.7596
23	118.2665	117.548	117.8047	117.3651	117.8786	117.6758	118.2206	117.5792
24	179.3649	179.2485	179.293	178.5639	178.826	179.3605	178.6136	178.8394
25	126.1362	126.1977	126.1828	126.6801	126.4074	126.3544	126.2485	126.6054

no. H	calcd. shileding tensors of H for each conformer							
	**3c-1**	**3c-2**	**3c-3**	**3c-4**	**3c-5**	**3c-6**	**3c-7**	**3c-8**
4	23.9329	23.7956	23.8379	24.0543	24.0353	23.7932	23.8922	24.0316
5	24.0846	23.9954	24.0444	24.0097	23.9803	24.0141	24.0501	24.0019
6	24.0727	23.9712	24.0543	24.1661	24.1839	23.9624	24.1525	24.1997
8	23.8699	23.852	23.8961	23.9974	23.9259	24.1199	23.8235	24.1837
12	27.0374	27.0301	27.0153	27.3788	27.1932	27.0408	27.3694	27.2229
16	24.8888	25.2793	24.8361	25.1248	24.8483	25.0522	24.7545	24.9753
17	25.7583	25.8806	25.8036	25.9005	25.7151	25.9612	25.7235	25.9351
17	25.8552	25.9526	25.7512	25.873	25.7972	26.1589	25.7434	26.2101
18	29.4773	30.277	29.6612	29.7515	30.3991	29.7554	29.3845	30.1289
18	30.4138	30.1758	30.0696	30.3487	30.0314	30.4295	30.5023	29.9196
18	29.8007	29.8018	30.1164	29.5907	30.0118	29.9821	30.0736	29.5527
19	30.0614	30.304	30.5093	30.1418	30.4536	29.9094	30.1095	30.4255
19	30.3211	29.8947	29.5574	30.2496	29.4657	30.2421	29.6991	29.6074
19	30.037	29.7055	30.1315	29.7686	29.7857	29.6345	30.1084	29.9067

20	29.6728	29.6278	29.6477	29.8955	29.7172	29.6473	29.8984	29.7249
20	30.351	30.3442	30.3373	29.9719	30.2453	30.3334	29.9891	30.1886
20	30.2656	30.2629	30.259	29.9698	30.2285	30.2667	30.0006	30.1922
21	28.7148	28.648	28.733	28.5346	28.6537	28.6901	28.7116	28.5498
21	27.5956	27.4958	27.5715	27.3386	27.4607	27.5287	27.5255	27.3533
22	27.6871	27.6461	27.714	27.4777	27.6124	27.6692	27.7218	27.4154
24	29.8463	29.8068	29.8239	29.7627	29.8376	29.8266	29.8802	29.7738
24	30.238	30.1405	30.2093	30.1442	30.1949	30.1634	30.2439	30.1471
24	30.2605	30.2398	30.2569	30.0785	30.1748	30.2735	30.1723	30.1088
25	28.0411	27.9792	27.9948	27.9217	28.0232	28.0148	28.1041	27.905
25	27.5235	27.4982	27.5092	27.5246	27.5577	27.5184	27.5983	27.5212

Table S11. The calculated shielding tensors of each conformer (>2%) for isomer **3d**.

no. C	calcd. shileding tensors of C for each conformer							
	**3d-1**	**3d-2**	**3d-3**	**3d-4**	**3d-5**	**3d-6**	**3d-7**	**3d-8**
2	52.6293	52.6292	51.8127	51.3918	50.4402	52.996	50.8581	53.0119
3	90.1712	90.1655	84.6604	93.042	93.7411	92.4011	91.7312	92.2093
3a	72.4027	72.4042	69.1986	71.6137	72.2954	71.4374	71.728	71.6348
4	79.6502	79.6539	79.6852	80.2012	79.8889	78.4284	79.071	79.4962
5	77.7236	77.7212	78.5336	77.9657	78.2613	78.7312	79.0083	78.6792
6	74.0617	74.0616	73.9424	73.6842	74.1416	74.0553	74.0074	73.9453
7	74.7619	74.7614	76.4498	75.3857	74.6533	77.5249	77.0495	78.1315
7a	65.5475	65.5453	66.2043	66.1512	65.7627	65.2212	65.4682	64.9769
8	84.7937	84.7886	87.0398	83.188	81.4226	85.9947	85.843	86.7439
9	72.4958	72.4907	73.672	72.8505	73.998	72.9558	74.1	73.278
10	38.5803	38.5767	40.2122	39.0342	38.8969	41.3602	40.4213	41.3052
12	141.4212	141.4246	139.1681	141.3505	141.45	139.5767	139.4528	139.3548
13	33.4117	33.4107	33.5413	33.6467	33.6444	33.6835	33.5105	33.5605
15	152.2218	152.2281	151.5276	151.6649	151.7146	151.0103	152.7926	151.3158
16	55.5587	55.5603	57.5833	52.5272	48.834	48.9158	47.2176	49.5882
17	85.4766	85.4826	84.3667	81.7396	87.968	86.4512	86.1005	86.9753
18	170.317	170.3178	166.9431	172.2598	167.1395	168.1381	169.9286	166.73
19	165.9621	165.953	170.1114	164.9026	171.6974	166.5357	165.2413	169.4491
20	177.0444	177.0576	172.3731	176.7215	176.5991	172.2571	172.5326	172.4121
21	156.6561	156.6548	156.7172	157.1524	156.372	156.0295	156.5482	155.9865
22	115.2847	115.286	115.8477	114.3703	115.3567	115.5721	115.7927	115.4816
23	117.6662	117.6654	117.7605	117.8811	117.4575	117.9642	117.4676	117.5678
24	177.1386	177.1382	177.1487	177.1435	177.1757	176.7417	177.343	177.3766
25	125.6156	125.606	125.4904	126.8332	125.1042	125.6223	125.5636	126.0281

no. H	calcd. shileding tensors of H for each conformer

	3d-1	3d-2	3d-3	3d-4	3d-5	3d-6	3d-7	3d-8
4	23.8239	23.8246	24.0346	23.8861	23.9135	23.9507	23.9314	23.9064
5	24.0695	24.0697	24.0514	24.0607	24.0454	24.0696	23.9917	24.031
6	24.107	24.1068	24.0932	24.1428	24.0435	24.1806	24.0845	24.1316
8	23.9038	23.9042	23.8412	23.8727	24.0871	23.8301	23.9792	23.9085
12	27.0462	27.0459	27.3786	26.9926	27.0051	27.2992	27.367	27.3239
16	25.2017	25.2021	25.0434	24.8027	24.8599	24.8848	24.6215	25.1652
17	25.9667	25.9673	25.9992	25.7103	25.9101	25.8016	25.797	25.8057
17	26.0095	26.0103	25.8383	25.6738	26.1777	25.9443	26.1291	26.1018
18	30.173	30.1728	29.6442	29.4703	29.3223	30.1614	30.1136	29.7598
18	30.1472	30.1475	30.2874	30.0919	30.4739	30.0798	29.8874	30.4556
18	29.7011	29.7	29.6798	30.1663	30.0558	29.6489	29.9413	29.7751
19	30.2688	30.2685	30.1392	30.4379	29.8604	30.4879	30.5931	30.0831
19	29.9326	29.9332	30.2185	29.5526	30.1575	29.7325	29.241	30.042
19	29.5716	29.5703	29.7427	30.1678	29.6669	29.7627	30.0283	29.6446
20	29.6457	29.6461	29.8975	29.6437	29.6724	29.8416	29.8882	29.852
20	30.3565	30.3569	29.9644	30.3247	30.3394	30.0271	29.9997	30.0268
20	30.2834	30.2838	29.9777	30.252	30.2726	30.0505	30.0276	30.0325
21	28.608	28.6082	28.5648	28.6971	28.6373	28.5981	28.5379	28.6301
21	27.9518	27.9514	27.6963	28.0651	27.8567	27.8051	27.7752	28.0828
22	26.6343	26.6338	26.4281	26.6531	26.6163	26.5391	26.5113	26.6979
24	30.5544	30.5547	30.5351	30.589	30.5853	30.5791	30.5392	30.5919
24	29.8613	29.8612	29.9749	29.9291	29.8768	30.061	29.8984	29.8969
24	30.4273	30.4275	30.366	30.4637	30.4095	30.405	30.3387	30.4
25	27.4771	27.4768	27.5119	27.5299	27.5482	27.5217	27.4889	27.5976
25	27.408	27.4079	27.4108	27.5258	27.3965	27.4652	27.3191	27.4204

Table S12. The calculated shielding tensors of each conformer (>2%) for isomer **4a**.

no. C	calcd. shileding tensors of C for each conformer						
	**4a-1**	**4a-2**	**4a-3**	**4a-4**	**4a-5**	**4a-6**	**4a-7**
2	51.2703	50.8165	52.1606	51.9316	51.9352	51.4138	51.4148
3	91.9025	92.398	93.0215	91.7782	91.7809	90.0542	90.0548
3a	70.923	71.5979	72.7644	71.3763	71.3754	69.8896	69.8966
4	80.0138	79.9306	79.6365	79.7872	79.7895	79.9744	79.975
5	77.2948	78.556	78.7201	78.378	78.3805	77.269	77.2727
6	74.4774	73.8789	73.3168	73.7611	73.7586	74.4069	74.4077
7	72.0295	74.3724	75.131	73.6484	73.6505	73.5241	73.5269
7a	65.1467	65.0085	64.3054	65.3255	65.3265	64.8335	64.8316
8	81.7592	86.2191	85.7293	84.1063	84.1057	81.5406	81.5421
9	74.659	74.3879	71.4259	76.7058	76.7007	75.6951	75.6923
10	38.2245	38.8782	39.2499	38.6049	38.6029	39.5156	39.5154
12	141.4701	141.2862	141.151	141.4593	141.4597	139.1952	139.1956
13	33.5184	33.3271	33.7692	33.5305	33.5282	33.5524	33.5512

15	152.1553	152.1618	150.8621	152.3533	152.3511	152.1976	152.197
16	50.0037	51.9454	51.4973	49.7624	49.7603	49.4077	49.406
17	87.1401	82.9347	84.6438	87.7674	87.7693	86.8094	86.8101
18	172.1797	165.1248	168.1977	173.4001	173.3962	167.6177	167.6121
19	168.0748	173.0496	169.1796	168.4233	168.4238	171.5014	171.4961
20	176.8087	176.7483	176.4958	177.0697	177.0654	172.4273	172.4294
21	159.0376	157.4846	157.5809	158.9249	158.9244	158.7594	158.76
22	116.5986	118.3981	118.8588	118.3899	118.3932	117.3591	117.3625
23	98.1172	96.5094	96.3391	96.3841	96.3982	96.4739	96.4726
24	174.9433	174.6823	174.654	174.699	174.6976	174.6051	174.6025
25	125.3143	125.2069	125.1649	125.1044	125.1052	125.1137	125.1138

no. H	calcd. shileding tensors of H for each conformer						
	**4a-1**	**4a-2**	**4a-3**	**4a-4**	**4a-5**	**4a-6**	**4a-7**
4	23.8274	23.8614	23.8401	23.8229	23.8231	23.9681	23.9683
5	24.1005	24.0474	24.0361	24.0492	24.0491	24.1272	24.1269
6	24.1036	24.2069	24.1918	24.1805	24.1806	24.2077	24.208
8	24.1202	23.9903	23.8318	24.1716	24.1718	24.2915	24.2916
12	26.9929	26.9784	27.0088	27.0442	27.0441	27.453	27.4521
16	24.9623	24.6932	24.846	24.9587	24.9585	24.8692	24.869
17	26.1459	25.6842	25.8218	26.1615	26.1615	26.1141	26.1142
17	25.9001	25.6564	25.6622	25.9914	25.9908	25.7958	25.7954
18	29.5973	30.0426	29.9026	29.5354	29.5361	29.8783	29.8786
18	29.9353	30.4683	30.0879	29.9759	29.9756	30.3778	30.3781
18	30.2224	29.6019	30.2845	30.2476	30.2477	29.5208	29.5213
19	29.5777	29.7139	29.5298	29.6544	29.6545	30.2018	30.2016
19	30.4083	30.141	30.296	30.3707	30.3707	29.9537	29.9535
19	29.9444	30.0703	29.8001	29.7464	29.7467	29.6004	29.6007
20	30.2875	30.2391	30.2542	30.2559	30.256	29.9987	29.9988
20	30.3581	30.3231	30.3386	30.3628	30.3627	30.0702	30.0704
20	29.687	29.631	29.6567	29.6383	29.6384	29.894	29.8935
21	28.3909	28.4342	28.4487	28.3246	28.3246	28.3177	28.3178
21	28.6356	27.7594	27.8215	27.6325	27.6324	27.7381	27.7379
22	26.9771	26.7756	26.8168	26.7146	26.7146	26.6864	26.6862
24	30.1778	30.8197	30.8082	30.7872	30.7871	30.7857	30.7855
24	30.1843	31.7632	31.7927	31.774	31.7737	31.5218	31.5211
24	29.2812	30.1847	30.1771	30.1226	30.1226	30.0775	30.0775
25	27.3196	27.4167	27.435	27.3704	27.37	27.4679	27.4682
25	27.5166	27.7501	27.7439	27.702	27.7022	27.7496	27.7494

Table S13. The calculated shielding tensors of each conformer (>2%) for isomer **4b**.

no. C	calcd. shileding tensors of C for each conformer			
	**4b-1**	**4b-2**	**4b-3**	**4b-4**
2	52.8025	51.9698	53.1163	51.7407
3	91.6041	95.6305	92.3622	93.2989
3a	72.9794	72.9764	70.2015	71.9843
4	79.9363	79.9179	79.5474	80.4804
5	77.5812	77.9763	79.8196	78.4494
6	74.8674	74.7205	74.1996	74.732
7	72.3156	72.6646	72.8394	72.6404
7a	64.8604	65.8523	65.0618	64.5892
8	84.1982	82.1555	86.6446	84.8388
9	72.7915	73.5648	71.704	72.1901
10	38.3647	39.0135	41.1852	39.3694
12	141.3428	141.3268	140.7096	141.1057
13	33.5273	33.4981	34.0532	33.6857
15	152.3048	151.5231	152.1541	151.2819
16	56.6438	52.8565	51.3338	51.3786
17	85.2159	83.2812	84.4813	85.4765
18	167.7026	165.3535	168.6415	168.9531
19	170.5608	172.7035	167.4264	169.0261
20	176.9672	176.5649	174.8145	176.4032
21	158.0343	157.7967	158.3218	157.6531
22	112.731	112.897	114.3469	112.9042
23	97.3081	97.1692	97.2062	97.1635
24	171.0026	170.9191	171.2892	170.856
25	129.6521	129.6189	129.7608	129.6758

no. H	calcd. shileding tensors of H for each conformer			
	**4b-1**	**4b-2**	**4b-3**	**4b-4**
4	23.7416	23.8381	24.0926	24.0025
5	24.1015	24.0546	24.0527	24.0725
6	24.1433	24.1454	24.1816	24.1448
8	23.9584	23.8765	23.8759	23.9602
12	27.0224	26.9633	27.2241	26.9911
16	25.1881	24.8856	24.9165	24.7654
17	26.0367	25.7396	25.7875	25.8646
17	26.0259	25.7918	25.6705	25.8076
18	30.1984	29.6789	30.4271	29.4399
18	30.1669	30.0715	30.0659	30.2934
18	29.7459	30.0587	29.9616	29.7825
19	29.5823	30.1553	29.7832	30.0146
19	30.2505	30.4828	30.3092	30.1083

19	29.9793	29.6472	29.4919	30.3021
20	30.2781	30.2477	30.1605	30.2487
20	30.3427	30.3256	30.1811	30.3467
20	29.6451	29.665	29.6976	29.6729
21	28.3965	28.4941	28.3279	28.476
21	28.5464	28.5756	28.5991	28.5583
22	27.2843	27.3356	27.4014	27.3056
24	29.189	29.1769	29.1941	29.199
24	30.0195	30.0133	30.0187	30.028
24	29.8518	29.8667	29.926	29.8647
25	27.6842	27.7272	27.6992	27.7318
25	27.1418	27.1727	27.1724	27.1887

Table S14. The calculated shielding tensors of each conformer (>2%) for isomer **4c**.

no. C	calcd. shileding tensors of C for each conformer					
	**4c-1**	**4c-2**	**4c-3**	**4c-4**	**4c-5**	**4c-6**
2	52.6929	52.0211	51.7131	51.5062	51.096	50.0336
3	88.816	93.4616	93.7717	90.6839	89.4069	91.9366
3a	71.9368	71.7367	72.1287	71.3256	68.9264	71.0102
4	80.0578	80.4881	80.0097	79.7015	80.0461	80.0032
5	77.4619	78.2657	77.6321	77.4414	79.1626	79.1489
6	74.8667	74.7278	74.8544	75.0107	74.7534	75.391
7	72.1887	72.8281	72.8474	72.6649	73.0343	74.382
7a	65.12	64.7372	65.8487	65.0253	66.0783	65.4396
8	84.9662	85.2891	83.9627	82.7369	84.2615	86.5474
9	72.9199	73.0862	74.5326	75.5703	75.7019	74.3293
10	40.0143	41.6649	40.7396	40.3549	38.4276	38.6592
12	139.0776	139.6695	139.2181	139.2048	141.4635	141.2189
13	32.9346	33.6836	33.2767	33.1985	33.4518	33.2831
15	152.1187	151.9244	151.6572	152.0007	152.8979	152.1141
16	56.4524	51.31	52.4217	50.0258	50.0984	51.6318
17	85.5987	85.2456	83.4612	86.944	87.7562	83.4281
18	170.4937	169.0485	172.4292	167.6942	173.1443	164.0091
19	168.2136	168.7829	165.6039	172.2783	168.0669	172.5519
20	172.6949	172.4955	172.8184	172.7985	176.7451	176.7078
21	158.1045	157.7276	158.0167	157.8679	157.8957	153.0169
22	112.6309	112.8359	112.8119	113.0443	113.6127	116.2569
23	97.2619	97.1523	97.1373	97.3924	96.9571	100.2926
24	170.9992	170.7703	170.8568	170.8052	171.5057	171.6712
25	129.6143	129.6297	129.5763	129.6672	129.6368	126.1513

no. H	calcd. shileding tensors of H for each conformer					
	**4c-1**	**4c-2**	**4c-3**	**4c-4**	**4c-5**	**4c-6**
4	23.8698	23.9974	23.8791	23.8922	24.0656	23.9905
5	24.0527	24.0642	24.08	24.0736	24.0967	24.0589
6	23.9828	23.9601	23.8645	24.1211	24.1404	23.8583
8	24.1035	24.1477	24.1631	24.1397	24.1413	24.2658
12	27.4062	27.3017	27.4085	27.4373	27.0174	26.9792
16	25.2411	24.7917	24.8897	24.9549	24.9102	24.7566
17	26.0529	25.8445	25.7152	26.1884	26.0943	25.714
17	25.9858	25.7963	25.7652	25.8937	25.9077	25.6729
18	29.9412	30.3364	29.5729	30.1975	29.6273	29.7729
18	30.2177	30.1307	30.4713	29.897	30.3947	30.1071
18	29.5589	30.0423	30.1098	29.6922	29.6754	30.0409
19	29.7991	29.8138	30.0234	30.0551	29.4835	30.0195
19	30.1679	30.3262	30.0518	30.4153	29.9387	30.4277
19	30.2567	29.4541	29.7231	29.6453	30.1881	29.4876
20	29.976	30.028	29.9645	29.9794	30.3022	30.2672
20	29.9716	30.0217	29.9817	30.0037	30.4053	30.3669
20	29.8339	29.8097	29.8506	29.8744	29.712	29.6777
21	28.5029	28.5568	28.5612	28.5669	28.4943	28.5062
21	28.3977	28.49	28.4959	28.3921	28.3371	28.4898
22	27.2731	27.3239	27.3444	27.3292	27.2558	27.8242
24	29.1771	29.2001	29.1751	29.2341	29.133	29.351
24	29.8426	29.8672	29.8664	29.8818	29.856	30.4908
24	29.9927	30.023	30.0023	30.0249	29.9367	29.4821
25	27.1302	27.194	27.1628	27.1491	27.1445	27.2821
25	27.6916	27.7453	27.7377	27.6979	27.6674	27.9861

Table S15. The calculated shielding tensors of each conformer (>2%) for isomer **4d**.

no. C	calcd. shileding tensors of C for each conformer					
	**4d-1**	**4d-2**	**4d-3**	**4d-4**	**4d-5**	**4d-6**
2	49.989	51.7346	51.0222	53.7453	52.2117	52.3079
3	91.7609	90.9467	90.276	92.5511	92.3163	92.8558
3a	71.2261	74.0272	69.6386	73.3501	72.4839	72.8384
4	79.3942	79.3327	79.9083	80.105	79.5181	79.5621
5	78.8127	78.3787	78.8158	77.2869	77.8846	78.8837
6	74.523	74.3515	74.3774	74.4778	74.5662	73.502
7	73.1877	75.2802	73.1132	74.2248	74.807	75.4872
7a	64.7807	64.029	66.1144	64.5836	64.9482	64.4707
8	86.2407	84.5921	85.0732	84.0913	82.4059	85.7941
9	74.8154	71.7718	76.1726	73.415	73.0773	71.2322
10	38.4944	38.8693	38.4619	38.782	38.7073	40.8675
12	141.3123	141.2049	141.4139	141.3375	141.1696	139.5232

13	33.2322	33.7957	33.466	33.7614	33.8498	33.4445
15	152.1567	150.1914	153.0903	152.3972	151.6205	151.1992
16	51.7567	51.6212	50.1684	58.1569	53.6778	51.4754
17	83.4631	85.7002	87.7077	84.2973	82.6168	84.6799
18	172.8832	170.0261	168.0912	169.66	172.429	168.0879
19	164.0097	169.2142	173.0195	167.7814	164.1066	169.3494
20	176.7967	176.4598	176.9011	176.8806	176.6581	172.6002
21	157.5277	157.7436	158.6279	159.2302	158.3057	157.2062
22	118.1636	117.5382	117.4929	117.8898	118.1463	118.6716
23	98.1329	96.4722	98.1965	96.5152	96.3008	96.4649
24	175.47	174.7376	175.4211	174.7073	174.7222	174.6641
25	125.3508	125.1375	125.2197	125.057	125.0718	125.1566

no. H	calcd. shileding tensors of H for each conformer					
	**4d-1**	**4d-2**	**4d-3**	**4d-4**	**4d-5**	**4d-6**
4	23.9073	23.8604	24.0299	23.7676	23.8572	23.8446
5	24.007	24.1076	24.077	24.0497	24.0673	24.0448
6	24.1552	24.2364	24.0972	24.191	24.2185	24.1762
8	23.8316	24.0524	24.0954	23.999	23.9937	23.799
12	26.9618	26.9706	27.0364	27.0255	26.9792	27.3275
16	24.6592	24.6553	24.8949	25.0484	24.676	24.8367
17	25.7375	25.7664	25.9497	26.1106	25.76	25.6714
17	25.7401	25.7854	26.0782	26.0544	25.7017	25.8146
18	29.6042	29.4043	29.6351	30.23	29.6482	30.2837
18	30.1129	30.2232	30.3847	30.1345	30.1545	30.0917
18	30.1565	29.8558	29.6664	29.7951	30.069	29.9241
19	30.4398	29.9572	29.9343	30.2027	30.4374	30.2827
19	29.4932	30.373	30.191	29.8613	29.5058	29.5233
19	30.0786	30.0455	29.4785	29.5143	30.11	29.8059
20	29.6546	29.7011	29.6936	29.6673	29.6697	29.837
20	30.3512	30.3461	30.4073	30.3657	30.344	29.9755
20	30.2502	30.2717	30.2975	30.2943	30.2727	30.02
21	28.4595	28.4357	28.4214	28.3792	28.4278	28.4476
21	28.6745	27.9161	28.6283	27.7716	27.873	27.8031
22	27.0837	26.8099	27.022	26.7547	26.7994	26.8141
24	30.2588	30.7264	30.2213	30.7136	30.708	30.7865
24	29.376	30.0514	29.2685	30.0457	30.0459	30.1461
24	30.2501	31.4021	30.1941	31.3772	31.3974	31.7193
25	27.552	27.6968	27.6224	27.6874	27.6708	27.7314
25	27.3608	27.4637	27.4455	27.4651	27.4408	27.4221

Table S16. The Cartesian coordinates of the lowest energy conformers (Conf. **1-8**) for isomer **2a**.

**conf. 2a-1**	**X axis(Å)**	**Y axis(Å)**	**Z axis(Å)**	**conf. 2a-1**	**X axis(Å)**	**Y axis(Å)**	**Z axis(Å)**
C	-0.172706	-2.791617	-1.786573	C	-0.172255	-2.72506	-2.073875
C	1.224944	-2.621709	-1.694125	C	1.223092	-2.606481	-1.897462
C	1.795302	-1.416886	-1.276979	C	1.806416	-1.445824	-1.386216
C	0.894301	-0.389725	-0.940288	C	0.922081	-0.405944	-1.039956
C	-0.515822	-0.541231	-0.995654	C	-0.484494	-0.50603	-1.189927
C	-1.047045	-1.764988	-1.449921	C	-1.030122	-1.685669	-1.73381
N	1.145319	0.912109	-0.552611	N	1.187932	0.861864	-0.564323
C	-0.035813	1.598514	-0.359045	C	0.01457	1.579445	-0.408877
C	-1.100011	0.730423	-0.611036	C	-1.055158	0.76267	-0.774057
C	-2.520834	1.060548	-0.563359	C	-2.485919	1.077342	-0.779659
C	-3.515265	0.320929	-0.021132	C	-3.466724	0.330618	-0.224861
C	-4.9203	0.822564	-0.089969	C	-4.890228	0.75616	-0.378253
N	-5.863135	0.027274	0.499193	N	-5.814892	-0.034534	0.246242
C	-5.700617	-1.335543	1.002048	C	-5.579934	-1.086044	1.234277
C	-4.236536	-1.696927	1.240007	C	-4.129067	-1.568467	1.241348
N	-3.294836	-0.873939	0.689367	N	-3.225112	-0.837659	0.522773
C	-0.007108	3.079034	0.023905	C	0.119896	2.995039	0.155689
C	1.386472	3.438882	0.524861	C	1.139905	3.754392	-0.689832
C	2.26542	4.272276	-0.038959	C	2.301608	4.277713	-0.288284
C	-0.986834	3.362276	1.193983	C	0.530334	2.910809	1.645514
C	-0.416049	3.922073	-1.206707	C	-1.213294	3.770283	0.059928
C	-6.360305	-2.378507	0.079971	C	-6.544622	-2.258593	1.019774
C	3.291042	-1.226615	-1.161238	C	3.297095	-1.299864	-1.184346
C	3.765276	-1.335038	0.301667	C	3.681501	-1.425614	0.302995
C	5.239524	-0.951686	0.565259	C	5.089955	-0.919503	0.68362
C	6.23654	-1.7873	-0.245588	C	6.218494	-1.656008	-0.046378
C	5.486173	0.547378	0.336527	C	5.216882	0.596609	0.472043
O	4.583455	1.370936	1.070206	O	4.14242	1.329469	1.063562
O	3.624989	-2.679797	0.789279	O	3.626067	-2.794402	0.739164
O	5.477998	-1.13012	1.975765	O	5.228723	-1.085985	2.108759
O	-3.935269	-2.700742	1.876641	O	-3.795687	-2.549108	1.897714
O	-5.215469	1.897239	-0.61322	O	-5.221717	1.73536	-1.045692
H	-0.568448	-3.741218	-2.136644	H	-0.577362	-3.642119	-2.493076
H	1.880666	-3.441631	-1.979052	H	1.868119	-3.432356	-2.190123
H	-2.11927	-1.90282	-1.55017	H	-2.099873	-1.777775	-1.898609
H	2.056111	1.316773	-0.372242	H	2.098999	1.216146	-0.291156
H	-2.857772	2.00158	-0.988214	H	-2.839115	1.970407	-1.283867
H	-6.808673	0.386321	0.418803	H	-6.768982	0.294134	0.139358
H	-6.17575	-1.395057	1.988888	H	-5.74871	-0.681234	2.245727
H	-2.332709	-1.156894	0.854008	H	-2.264865	-1.162658	0.594995

H	1.668109	2.951242	1.460273	H	0.845079	3.870303	-1.734413
H	3.231833	4.452262	0.424095	H	2.939167	4.814848	-0.986218
H	2.064736	4.80021	-0.967444	H	2.668799	4.190624	0.730133
H	-2.020334	3.133621	0.922035	H	-0.226666	2.355029	2.20911
H	-0.930371	4.42121	1.47036	H	0.608662	3.915322	2.07696
H	-0.728841	2.764124	2.075362	H	1.492223	2.406215	1.781526
H	0.251127	3.742977	-2.056741	H	-1.550824	3.875196	-0.976476
H	-0.391124	4.990525	-0.963449	H	-1.067818	4.777269	0.465802
H	-1.432957	3.674836	-1.524652	H	-2.00736	3.282798	0.633405
H	-7.429757	-2.163475	-0.026868	H	-7.582405	-1.915166	1.10425
H	-5.905545	-2.361154	-0.916679	H	-6.403556	-2.698229	0.026695
H	-6.245855	-3.37868	0.508087	H	-6.367061	-3.026715	1.776194
H	3.807957	-1.982078	-1.762464	H	3.833166	-2.064934	-1.756164
H	3.574634	-0.244497	-1.56295	H	3.62274	-0.323854	-1.567856
H	3.152239	-0.671474	0.92373	H	2.972232	-0.84317	0.901999
H	7.256902	-1.548997	0.072049	H	7.18732	-1.306578	0.325882
H	6.153861	-1.587677	-1.320838	H	6.180367	-1.484126	-1.128843
H	6.064043	-2.855357	-0.081757	H	6.148774	-2.733495	0.130537
H	5.361349	0.814135	-0.71634	H	5.211646	0.852488	-0.590322
H	6.525151	0.766055	0.621296	H	6.178038	0.918693	0.895423
H	4.65664	1.092134	1.999794	H	4.126959	1.081846	2.004505
H	2.685927	-2.925469	0.751692	H	2.707223	-3.101694	0.672474
H	5.060565	-1.972529	2.229078	H	4.881056	-1.969442	2.324873
Free Energy = -1473.195673 Hartree				Free Energy = -1473.19564 Hartree			

conf. 2a-3	X axis(Å)	Y axis(Å)	Z axis(Å)	conf. 2a-4	X axis(Å)	Y axis(Å)	Z axis(Å)
C	0.036542	-2.88822	-1.684376	C	-0.089921	-3.199038	-0.735288
C	1.429196	-2.683631	-1.595836	C	-1.412665	-3.06301	-0.264795
C	1.970301	-1.465276	-1.181198	C	-1.920942	-1.834071	0.160387
C	1.04832	-0.459409	-0.831981	C	-1.035934	-0.740252	0.10466
C	-0.357305	-0.64562	-0.892657	C	0.313465	-0.859092	-0.31819
C	-0.859715	-1.880458	-1.350233	C	0.775083	-2.112155	-0.769347
N	1.272779	0.852183	-0.445868	N	-1.253033	0.592187	0.414767
C	0.070031	1.507443	-0.26192	C	-0.102142	1.325841	0.201337
C	-0.971701	0.612638	-0.517353	C	0.904807	0.461988	-0.236843
C	-2.393557	0.928046	-0.464547	C	2.264542	0.854316	-0.582498
C	-3.388825	0.165922	0.045877	C	3.41473	0.196825	-0.303493
C	-4.795264	0.664411	-0.008337	C	4.714822	0.767114	-0.76606
N	-5.738184	-0.169179	0.530103	N	5.822168	0.021328	-0.459045
C	-5.570933	-1.575305	0.892189	C	5.837486	-1.385876	-0.059618
C	-4.113737	-1.919356	1.19017	C	4.569509	-1.758004	0.713234
N	-3.168827	-1.051711	0.720721	N	3.479337	-0.96215	0.495651

C	0.004365	2.979969	0.143288	C	-0.041284	2.829844	0.468602
C	-0.639408	3.745817	-1.010276	C	0.229099	3.530977	-0.860396
C	-1.73723	4.50558	-0.969472	C	1.243733	4.347432	-1.157458
C	1.418506	3.575706	0.346264	C	1.038404	3.124627	1.534123
C	-0.773616	3.126484	1.469989	C	-1.393456	3.365166	0.999399
C	-6.132536	-2.530373	-0.17914	C	7.095358	-1.699104	0.752522
C	3.459994	-1.22037	-1.119206	C	-3.348336	-1.667757	0.627477
C	3.984717	-1.193972	0.329552	C	-4.23026	-1.005267	-0.448869
C	5.377966	-0.558974	0.527133	C	-5.580619	-0.44076	0.042976
C	6.494819	-1.305743	-0.210806	C	-6.512885	-1.512054	0.619435
C	5.37689	0.923246	0.127689	C	-5.373729	0.688639	1.06213
O	4.269916	1.646564	0.673425	O	-4.403793	1.650377	0.638392
O	4.075942	-2.518996	0.878651	O	-4.550042	-1.927612	-1.503528
O	5.640987	-0.543562	1.943991	O	-6.205006	0.208816	-1.082017
O	-3.819719	-2.94607	1.793693	O	4.523407	-2.741358	1.443913
O	-5.094491	1.766193	-0.468609	O	4.803167	1.851298	-1.341736
H	-0.337655	-3.846575	-2.034283	H	0.257328	-4.173354	-1.068472
H	2.104722	-3.48485	-1.887959	H	-2.055645	-3.939629	-0.221554
H	-1.928084	-2.040826	-1.455541	H	1.786932	-2.229039	-1.144294
H	2.180392	1.243997	-0.212157	H	-2.149384	0.993283	0.671815
H	-2.717924	1.891915	-0.845958	H	2.404834	1.797713	-1.102147
H	-6.686215	0.183602	0.451316	H	6.687483	0.409163	-0.82024
H	-6.107796	-1.75051	1.831579	H	5.833228	-2.028926	-0.957402
H	-2.208074	-1.322465	0.910718	H	2.634701	-1.254411	0.978847
H	-0.10401	3.647272	-1.956327	H	-0.522374	3.328894	-1.62611
H	-2.092661	5.017637	-1.859877	H	1.32195	4.802473	-2.141422
H	-2.3309	4.637917	-0.069276	H	2.037239	4.583583	-0.45397
H	1.963001	3.06696	1.149517	H	0.794566	2.602422	2.465971
H	1.32504	4.631022	0.621354	H	1.078396	4.1989	1.747997
H	2.026491	3.525955	-0.563863	H	2.032335	2.798996	1.216625
H	-1.797355	2.751143	1.392005	H	-2.210057	3.224582	0.283531
H	-0.813524	4.178869	1.773999	H	-1.30332	4.439831	1.18638
H	-0.268848	2.564045	2.263446	H	-1.677732	2.88292	1.942123
H	-7.198496	-2.331369	-0.338839	H	7.992292	-1.503122	0.153031
H	-5.612209	-2.395405	-1.133941	H	7.135136	-1.081904	1.656086
H	-6.016022	-3.568136	0.147755	H	7.093945	-2.750717	1.047553
H	3.992246	-2.000027	-1.674819	H	-3.775771	-2.643071	0.884385
H	3.686857	-0.265003	-1.610265	H	-3.363559	-1.05953	1.541095
H	3.290372	-0.613137	0.947226	H	-3.683283	-0.16147	-0.884551
H	7.461245	-0.84236	0.014514	H	-7.472047	-1.059286	0.892446
H	6.344375	-1.285673	-1.296821	H	-6.084108	-1.982929	1.512136
H	6.529425	-2.351424	0.108562	H	-6.698537	-2.294664	-0.122093
H	5.310295	1.041486	-0.956094	H	-5.016387	0.299616	2.018241

H	6.323764	1.367316	0.462163	H	-6.343351	1.174917	1.233026
H	4.332691	1.564328	1.640611	H	-4.725013	2.01723	-0.203292
H	3.182651	-2.897418	0.921793	H	-3.724914	-2.18794	-1.944737
H	5.397816	-1.421696	2.286691	H	-6.119246	-0.394234	-1.841374
Free Energy = -1473.195357 Hartree				Free Energy = -1473.195266 Hartree			

conf. 2a-5	X axis(Å)	Y axis(Å)	Z axis(Å)	conf. 2a-6	X axis(Å)	Y axis(Å)	Z axis(Å)
C	0.037426	-2.993093	-1.557442	C	0.152832	-3.261188	-0.559316
C	1.430719	-2.796553	-1.459677	C	-1.1666	-3.126557	-0.076943
C	1.978486	-1.560123	-1.113248	C	-1.724977	-1.879123	0.206639
C	1.061785	-0.525036	-0.843292	C	-0.892903	-0.762494	-0.005549
C	-0.34428	-0.702499	-0.911806	C	0.449316	-0.873047	-0.447825
C	-0.853185	-1.958263	-1.299099	C	0.964103	-2.148897	-0.749918
N	1.293119	0.80352	-0.527191	N	-1.15817	0.583004	0.149995
C	0.09299	1.476825	-0.39098	C	-0.044739	1.339513	-0.171875
C	-0.951778	0.580249	-0.614774	C	0.987271	0.473188	-0.536569
C	-2.375634	0.901978	-0.625604	C	2.345227	0.805102	-0.97019
C	-3.380938	0.179446	-0.08086	C	3.491197	0.244013	-0.522422
C	-4.788241	0.655955	-0.225465	C	4.796766	0.681343	-1.102898
N	-5.740175	-0.117767	0.381988	N	5.904439	0.066751	-0.585896
C	-5.578138	-1.466505	0.921439	C	5.941306	-1.150843	0.223773
C	-4.122463	-1.78314	1.2547	C	4.636464	-1.359946	0.995817
N	-3.171788	-0.971582	0.704506	N	3.542123	-0.679812	0.538186
C	0.04843	2.974969	-0.100227	C	-0.146562	2.855756	-0.005834
C	-1.06879	3.267986	0.894465	C	-0.417902	3.126849	1.472994
C	-2.019403	4.199795	0.7927	C	-1.492847	3.711364	2.009625
C	-0.126865	3.733752	-1.437594	C	-1.26805	3.40035	-0.921871
C	1.363957	3.455155	0.56828	C	1.165512	3.585066	-0.37215
C	-6.152495	-2.545448	-0.016988	C	7.145139	-1.13129	1.169442
C	3.469555	-1.324134	-1.045063	C	-3.144822	-1.711592	0.69634
C	3.967162	-1.156947	0.403605	C	-4.073739	-1.171882	-0.408891
C	5.363274	-0.518281	0.563694	C	-5.417227	-0.580006	0.070076
C	6.489487	-1.356719	-0.051576	C	-6.296331	-1.593161	0.811355
C	5.39238	0.90654	-0.00724	C	-5.202013	0.67266	0.932225
O	4.275033	1.698856	0.407192	O	-4.314052	1.61464	0.325015
O	4.030832	-2.41929	1.086954	O	-4.41308	-2.202014	-1.353017
O	5.590839	-0.340415	1.975801	O	-6.103236	-0.086124	-1.097708
O	-3.834784	-2.744683	1.96022	O	4.571576	-2.134044	1.943853
O	-5.082344	1.692267	-0.820779	O	4.883313	1.563769	-1.956083
H	-0.341347	-3.968012	-1.852335	H	0.539633	-4.253359	-0.775686
H	2.102167	-3.621507	-1.688228	H	-1.765843	-4.019309	0.089852
H	-1.921893	-2.114826	-1.407646	H	1.975464	-2.263221	-1.12826

H	2.204542	1.201642	-0.318844	H	-2.046413	0.982236	0.437909
H	-2.70033	1.823784	-1.096402	H	2.494831	1.567375	-1.726652
H	-6.687451	0.216707	0.239736	H	6.773725	0.36111	-1.01879
H	-6.111376	-1.517881	1.878086	H	6.032597	-2.031284	-0.435533
H	-2.21344	-1.223951	0.929196	H	2.677224	-0.880828	1.032718
H	-1.02713	2.659586	1.799316	H	0.385606	2.796712	2.133897
H	-2.744136	4.343059	1.589593	H	-1.56405	3.862726	3.083942
H	-2.13643	4.833845	-0.081935	H	-2.336632	4.05422	1.417343
H	0.690356	3.482034	-2.122151	H	-1.045178	3.161134	-1.967334
H	-0.107461	4.817344	-1.273599	H	-1.335397	4.490151	-0.828801
H	-1.070598	3.477064	-1.92882	H	-2.248917	2.981801	-0.677981
H	1.549732	2.928951	1.512326	H	2.016755	3.22649	0.214135
H	1.280325	4.522889	0.793824	H	1.042429	4.6546	-0.170359
H	2.236546	3.320567	-0.080029	H	1.406285	3.47026	-1.433683
H	-7.218385	-2.361236	-0.194321	H	8.076637	-1.060949	0.59534
H	-5.637468	-2.535532	-0.984073	H	7.087928	-0.275467	1.850195
H	-6.039696	-3.534022	0.43815	H	7.166266	-2.049318	1.76088
H	4.004222	-2.15946	-1.510089	H	-3.536815	-2.670519	1.052407
H	3.714723	-0.42358	-1.623095	H	-3.152769	-1.024422	1.552683
H	3.269794	-0.508622	0.946224	H	-3.556955	-0.369476	-0.947278
H	7.455273	-0.878613	0.144001	H	-7.25811	-1.133768	1.062777
H	6.367655	-1.463687	-1.136062	H	-5.822336	-1.93673	1.738571
H	6.503564	-2.357866	0.389026	H	-6.484683	-2.467372	0.181105
H	5.364543	0.895278	-1.099099	H	-4.763502	0.420701	1.900392
H	6.331558	1.380989	0.305904	H	-6.182277	1.134552	1.111348
H	4.310868	1.752608	1.377534	H	-4.689807	1.820855	-0.548738
H	3.132774	-2.784355	1.144137	H	-3.600111	-2.488868	-1.800028
H	5.333154	-1.171714	2.411764	H	-6.01901	-0.77052	-1.785042
Free Energy = -1473.195054 Hartree				Free Energy = -1473.194394 Hartree			

conf. 2a-7	X axis(Å)	Y axis(Å)	Z axis(Å)	conf. 2a-8	X axis(Å)	Y axis(Å)	Z axis(Å)
C	0.110963	-3.255811	0.5878	C	0.110655	-3.255777	0.587107
C	1.433852	-3.094866	0.124963	C	1.433617	-3.094879	0.124461
C	1.941472	-1.844557	-0.232642	C	1.94136	-1.844552	-0.232887
C	1.056488	-0.755603	-0.116099	C	1.05642	-0.755557	-0.116297
C	-0.291751	-0.896006	0.302445	C	-0.291869	-0.895895	0.30204
C	-0.753752	-2.171957	0.682826	C	-0.754001	-2.171871	0.68218
N	1.273558	0.589971	-0.357334	N	1.273594	0.590024	-0.357541
C	0.123436	1.312627	-0.103527	C	0.123523	1.312787	-0.103715
C	-0.88121	0.429522	0.294739	C	-0.881225	0.42973	0.294386
C	-2.234033	0.794529	0.701865	C	-2.234044	0.794817	0.701469
C	-3.386873	0.155499	0.396264	C	-3.386926	0.155868	0.395846

C	-4.675691	0.666231	0.949862	C	-4.675745	0.666718	0.949363
N	-5.790477	-0.042328	0.58572	N	-5.790457	-0.042191	0.585655
C	-5.818631	-1.396603	0.033198	C	-5.818235	-1.396884	0.03408
C	-4.559594	-1.693204	-0.785383	C	-4.559604	-1.693142	-0.785279
N	-3.464507	-0.924562	-0.505144	N	-3.464618	-0.924126	-0.505628
C	0.099423	2.832752	-0.245158	C	0.099679	2.832951	-0.245274
C	-1.227466	3.2674	-0.856906	C	-1.227138	3.267799	-0.857022
C	-2.046138	4.225402	-0.416256	C	-2.045659	4.225935	-0.416377
C	1.205998	3.316374	-1.221918	C	1.206305	3.316543	-1.221989
C	0.351151	3.467325	1.143545	C	0.351445	3.467426	1.143474
C	-7.084606	-1.610716	-0.798807	C	-7.084711	-1.612285	-0.796768
C	3.367779	-1.646597	-0.690608	C	3.367686	-1.646531	-0.690751
C	4.240654	-1.018267	0.414038	C	4.240594	-1.018699	0.414152
C	5.566558	-0.380772	-0.054517	C	5.566497	-0.381028	-0.054172
C	6.523535	-1.382039	-0.71056	C	6.523459	-1.382021	-0.710653
C	5.313067	0.806938	-0.993873	C	5.312979	0.80705	-0.99304
O	4.326829	1.713441	-0.490159	O	4.32668	1.713293	-0.489052
O	4.601075	-1.988837	1.410853	O	4.601012	-1.989677	1.410563
O	6.184955	0.213542	1.103815	O	6.18493	0.212785	1.10438
O	-4.52534	-2.600844	-1.608952	O	-4.525528	-2.600747	-1.608877
O	-4.749778	1.675113	1.650358	O	-4.749875	1.675875	1.649452
H	-0.236602	-4.24685	0.866958	H	-0.23704	-4.246829	0.866061
H	2.076811	-3.967944	0.034193	H	2.076497	-3.968012	0.033624
H	-1.76553	-2.309325	1.051093	H	-1.765828	-2.309265	1.050292
H	2.170926	1.004385	-0.589716	H	2.17112	1.004472	-0.58927
H	-2.373262	1.685362	1.305013	H	-2.37323	1.685713	1.304521
H	-6.645921	0.299933	1.010931	H	-6.645983	0.300165	1.010629
H	-5.814046	-2.134551	0.85471	H	-5.812493	-2.134281	0.85609
H	-2.627512	-1.167808	-1.027408	H	-2.627795	-1.167077	-1.028304
H	-1.478891	2.748954	-1.783347	H	-1.47861	2.749451	-1.783504
H	-2.951614	4.476717	-0.961858	H	-2.951085	4.477395	-0.961994
H	-1.872025	4.776593	0.503936	H	-1.871469	4.777085	0.503821
H	2.214759	3.089194	-0.860195	H	2.215052	3.089359	-0.860227
H	1.131547	4.402658	-1.334413	H	1.131883	4.402834	-1.334464
H	1.087354	2.867594	-2.215028	H	1.087684	2.867771	-2.215097
H	-0.433119	3.199911	1.858873	H	-0.432789	3.199919	1.858803
H	0.392148	4.560046	1.06888	H	0.392417	4.560162	1.068922
H	1.309007	3.122505	1.548569	H	1.309332	3.122606	1.548432
H	-7.9758	-1.473014	-0.175018	H	-7.975516	-1.474845	-0.172362
H	-7.125384	-0.899678	-1.630539	H	-7.126609	-0.901737	-1.628868
H	-7.094294	-2.624686	-1.204717	H	-7.094054	-2.626466	-1.202146
H	3.806476	-2.606086	-0.985484	H	3.806292	-2.605934	-0.986024
H	3.376657	-1.003316	-1.580159	H	3.376567	-1.002857	-1.580009

H	3.671814	-0.219679	0.903312	H	3.671741	-0.220302	0.903722
H	7.463589	-0.881993	-0.966914	H	7.46359	-0.881872	-0.966561
H	6.096053	-1.809266	-1.625526	H	6.096118	-1.808703	-1.625936
H	6.745203	-2.203433	-0.022847	H	6.744976	-2.203812	-0.023366
H	4.94792	0.473491	-1.967809	H	4.94788	0.473973	-1.967121
H	6.266053	1.330197	-1.147061	H	6.265946	1.330414	-1.145995
H	4.644843	2.017672	0.377575	H	4.644628	2.017316	0.378769
H	3.789995	-2.295461	1.848241	H	3.789997	-2.29618	1.848146
H	6.12311	-0.438619	1.82398	H	6.123022	-0.439649	1.824288
Free Energy = -1473.194236 Hartree				Free Energy = -1473.194224 Hartree			

Table S17. The Cartesian coordinates of the lowest energy conformers (Conf. **1-7**) for isomer **2b**.

conf. 2b-1	X axis(Å)	Y axis(Å)	Z axis(Å)	conf. 2b-2	X axis(Å)	Y axis(Å)	Z axis(Å)
C	-0.162019	-2.875702	-1.544915	C	-0.089921	-3.199038	-0.735288
C	-1.529082	-2.581669	-1.367809	C	-1.412665	-3.06301	-0.264795
C	-1.974418	-1.31419	-0.985678	C	-1.920942	-1.834071	0.160387
C	-0.969245	-0.356221	-0.756535	C	-1.035934	-0.740252	0.10466
C	0.417655	-0.629537	-0.901538	C	0.313465	-0.859092	-0.31819
C	0.814982	-1.910705	-1.33102	C	0.775083	-2.112155	-0.769347
N	-1.091741	0.978037	-0.423552	N	-1.253033	0.592187	0.414767
C	0.14888	1.56704	-0.335306	C	-0.102142	1.325841	0.201337
C	1.124298	0.606915	-0.620551	C	0.904807	0.461988	-0.236843
C	2.559195	0.8461	-0.68052	C	2.264542	0.854316	-0.582498
C	3.557746	0.040584	-0.248072	C	3.41473	0.196825	-0.303493
C	4.97398	0.467561	-0.436264	C	4.714822	0.767114	-0.76606
N	5.916815	-0.41012	0.032784	N	5.822168	0.021328	-0.459045
C	5.705674	-1.822365	0.350161	C	5.837486	-1.385876	-0.059618
C	4.278416	-2.075647	0.842904	C	4.569509	-1.758004	0.713234
N	3.342586	-1.152166	0.472266	N	3.479337	-0.96215	0.495651
C	0.296022	3.050184	-0.002523	C	-0.041284	2.829844	0.468602
C	1.342947	3.219155	1.092576	C	0.229099	3.530977	-0.860396
C	2.412515	4.017778	1.075909	C	1.243733	4.347432	-1.157458
C	-1.031075	3.624317	0.559662	C	1.038404	3.124627	1.534123
C	0.646669	3.824709	-1.295601	C	-1.393456	3.365166	0.999399
C	6.738188	-2.302068	1.372091	C	7.095358	-1.699104	0.752522
C	-3.45917	-1.007798	-0.913924	C	-3.348336	-1.667757	0.627477
C	-3.993617	-0.529905	0.448301	C	-4.23026	-1.005267	-0.448869
C	-5.505486	-0.79051	0.685192	C	-5.580619	-0.44076	0.042976
O	-5.791113	-0.047962	1.888608	C	-6.512885	-1.512054	0.619435
C	-6.37133	-0.213957	-0.447493	C	-5.373729	0.688639	1.06213
O	-7.722769	-0.199621	0.038634	O	-4.403793	1.650377	0.638392

O	-3.74291	0.881178	0.552373	O	-4.550042	-1.927612	-1.503528
C	-5.798882	-2.275753	0.927624	O	-6.205006	0.208816	-1.082017
O	3.988478	-3.075493	1.49165	O	4.523407	-2.741358	1.443913
O	5.289219	1.546961	-0.937565	O	4.803167	1.851298	-1.341736
H	0.128074	-3.872977	-1.864891	H	0.257328	-4.173354	-1.068472
H	-2.264144	-3.360711	-1.559512	H	-2.055645	-3.939629	-0.221554
H	1.861191	-2.142889	-1.503053	H	1.786932	-2.229039	-1.144294
H	-1.967272	1.36787	-0.085113	H	-2.149384	0.993283	0.671815
H	2.910451	1.787603	-1.089005	H	2.404834	1.797713	-1.102147
H	6.870785	-0.115896	-0.148855	H	6.687483	0.409163	-0.82024
H	5.812927	-2.431055	-0.565058	H	5.833228	-2.028926	-0.957402
H	2.39496	-1.359906	0.774307	H	2.634701	-1.254411	0.978847
H	1.143363	2.62881	1.988499	H	-0.522374	3.328894	-1.62611
H	3.076875	4.075356	1.934004	H	1.32195	4.802473	-2.141422
H	2.687359	4.620336	0.214507	H	2.037239	4.583583	-0.45397
H	-1.843121	3.563936	-0.17515	H	0.794566	2.602422	2.465971
H	-0.888014	4.680003	0.810486	H	1.078396	4.1989	1.747997
H	-1.345061	3.104249	1.472242	H	2.032335	2.798996	1.216625
H	1.598934	3.499775	-1.724618	H	-2.210057	3.224582	0.283531
H	0.711212	4.900666	-1.095585	H	-1.30332	4.439831	1.18638
H	-0.132977	3.666065	-2.04854	H	-1.677732	2.88292	1.942123
H	7.751917	-2.183113	0.971286	H	7.992292	-1.503122	0.153031
H	6.658044	-1.726679	2.300249	H	7.135136	-1.081904	1.656086
H	6.572645	-3.357456	1.599572	H	7.093945	-2.750717	1.047553
H	-3.718372	-0.238309	-1.655731	H	-3.775771	-2.643071	0.884385
H	-3.993321	-1.917102	-1.207262	H	-3.363559	-1.05953	1.541095
H	-3.456427	-1.04609	1.257051	H	-3.683283	-0.16147	-0.884551
H	-6.861083	-2.415737	1.151138	H	-7.472047	-1.059286	0.892446
H	-5.21507	-2.637176	1.780192	H	-6.084108	-1.982929	1.512136
H	-5.55703	-2.889649	0.052701	H	-6.698537	-2.294664	-0.122093
H	-6.296935	-0.839169	-1.3463	H	-5.016387	0.299616	2.018241
H	-6.038096	0.803393	-0.684186	H	-6.343351	1.174917	1.233026
H	-8.274256	0.335176	-0.550986	H	-4.725013	2.01723	-0.203292
H	-4.251398	1.18301	1.328512	H	-3.724914	-2.18794	-1.944737
H	-6.754092	0.097015	1.908988	H	-6.119246	-0.394234	-1.841374
Free Energy = -1473.195332 a.u.				Free Energy = -1473.19394 a.u.			

**conf. 2b-3**	**X axis(Å)**	**Y axis(Å)**	**Z axis(Å)**	**conf. 2b-4**	**X axis(Å)**	**Y axis(Å)**	**Z axis(Å)**
C	-0.346757	-2.064199	2.58795	C	0.229281	-3.102712	-0.013025
C	-1.717418	-1.782741	2.411719	C	1.477877	-2.866863	-0.623594
C	-2.168339	-0.640543	1.744783	C	1.924945	-1.580807	-0.937918
C	-1.163553	0.210993	1.246572	C	1.038046	-0.533705	-0.62189

C	0.224055	-0.052936	1.399001	C	-0.247615	-0.74354	-0.053382
C	0.630504	-1.205658	2.098493	C	-0.637791	-2.056044	0.27788
N	-1.27417	1.423788	0.603312	N	1.196684	0.827829	-0.777088
C	-0.027408	1.945456	0.327998	C	0.086727	1.499652	-0.320342
C	0.940745	1.057996	0.801032	C	-0.854614	0.563132	0.120796
C	2.398225	1.178198	0.732458	C	-2.156677	0.855877	0.705992
C	3.245821	0.239529	0.25418	C	-3.329673	0.216584	0.486662
C	4.718184	0.477462	0.297763	C	-4.553027	0.668098	1.212672
N	5.493658	-0.506794	-0.250784	N	-5.693488	-0.034872	0.932557
C	5.069952	-1.653558	-1.052286	C	-5.777589	-1.342149	0.281501
C	3.563866	-1.903145	-0.971801	C	-4.608755	-1.567794	-0.681208
N	2.809156	-0.96846	-0.323979	N	-3.496135	-0.80154	-0.471602
C	0.055216	3.230341	-0.494275	C	0.047309	3.028365	-0.345888
C	-1.069402	4.156774	-0.044522	C	1.15844	3.547488	-1.249773
C	-2.072094	4.637557	-0.785138	C	2.184348	4.333286	-0.910785
C	-0.047506	2.862716	-1.994166	C	0.175556	3.565575	1.098295
C	1.382485	3.991147	-0.260417	C	-1.28618	3.530888	-0.966142
C	5.850722	-2.913754	-0.656163	C	-7.121333	-1.498973	-0.433856
C	-3.649698	-0.322142	1.669422	C	3.246192	-1.37885	-1.657259
C	-4.357421	-0.245281	0.304651	C	4.383736	-0.577326	-0.99836
C	-4.313524	-1.481435	-0.631826	C	4.9033	-1.019911	0.393643
O	-5.067	-1.027452	-1.77924	O	5.943067	-0.051106	0.65872
C	-2.899202	-1.848477	-1.114902	C	3.857217	-0.902474	1.516187
O	-3.078839	-2.701298	-2.260373	O	4.594503	-0.940967	2.752819
O	-3.886815	0.91626	-0.390544	O	4.011564	0.80642	-0.970095
C	-5.017928	-2.689103	-0.001828	C	5.513033	-2.425974	0.343548
O	3.062964	-2.892888	-1.496916	O	-4.65559	-2.422556	-1.558639
O	5.214319	1.479523	0.811841	O	-4.557555	1.633115	1.977525
H	-0.055481	-2.960078	3.129897	H	-0.061116	-4.123102	0.222834
H	-2.451814	-2.46865	2.829255	H	2.113465	-3.716515	-0.864505
H	1.68367	-1.412729	2.266597	H	-1.592838	-2.251637	0.755413
H	-2.151479	1.770263	0.224938	H	2.09276	1.252449	-1.008097
H	2.885177	2.073442	1.105001	H	-2.243357	1.690166	1.395961
H	6.487688	-0.305292	-0.220643	H	-6.50251	0.271064	1.463124
H	5.26706	-1.446474	-2.116522	H	-5.69135	-2.140969	1.038675
H	1.810279	-1.155961	-0.320284	H	-2.717358	-0.999598	-1.093705
H	-1.006783	4.452347	1.00432	H	1.060159	3.247556	-2.294915
H	-2.807834	5.312593	-0.355383	H	2.900033	4.666208	-1.65825
H	-2.203179	4.387731	-1.834715	H	2.352401	4.678713	0.105762
H	0.780952	2.203111	-2.272407	H	-0.645336	3.19962	1.721455
H	0.009293	3.76235	-2.618176	H	0.137807	4.661053	1.106087
H	-0.984178	2.34257	-2.222811	H	1.115485	3.247386	1.56254
H	1.537542	4.216433	0.800629	H	-1.41485	3.145825	-1.984019

H	1.352332	4.940418	-0.806297	H	-1.277759	4.625793	-1.012305
H	2.24391	3.422726	-0.621853	H	-2.151765	3.219461	-0.376131
H	6.925896	-2.752347	-0.79733	H	-7.945389	-1.412623	0.28433
H	5.67218	-3.163659	0.39507	H	-7.242266	-0.727091	-1.201053
H	5.537443	-3.754492	-1.279841	H	-7.174619	-2.479436	-0.912156
H	-4.188822	-1.056221	2.278167	H	3.647471	-2.365735	-1.912884
H	-3.832785	0.652359	2.145311	H	3.059209	-0.875352	-2.616791
H	-5.426085	-0.096141	0.524685	H	5.251806	-0.677374	-1.668219
H	-5.075515	-3.507223	-0.726387	H	5.971222	-2.669188	1.307205
H	-6.037171	-2.421432	0.296896	H	6.28814	-2.475749	-0.428738
H	-4.475462	-3.055393	0.876998	H	4.751757	-3.185098	0.131096
H	-2.344976	-2.372478	-0.330593	H	3.14289	-1.729875	1.471915
H	-2.352777	-0.941982	-1.39862	H	3.316483	0.046301	1.427091
H	-2.215137	-2.898921	-2.651833	H	3.996158	-0.742624	3.48792
H	-4.310025	0.886915	-1.268357	H	4.721694	1.264807	-0.484251
H	-4.808609	-1.597865	-2.525879	H	6.087725	-0.054548	1.622355
Free Energy = -1473.193646 Hartree				Free Energy = -1473.192894 Hartree			

conf. 2b-5	X axis(Å)	Y axis(Å)	Z axis(Å)	conf. 2b-6	X axis(Å)	Y axis(Å)	Z axis(Å)
C	0.209201	-3.089459	0.071796	C	0.152832	-3.261188	-0.559316
C	1.438959	-2.85295	-0.576117	C	-1.1666	-3.126557	-0.076943
C	1.881931	-1.567212	-0.897457	C	-1.724977	-1.879123	0.206639
C	1.008707	-0.5177	-0.549557	C	-0.892903	-0.762494	-0.005549
C	-0.25498	-0.729013	0.063728	C	0.449316	-0.873047	-0.447825
C	-0.642836	-2.040838	0.397323	C	0.964103	-2.148897	-0.749918
N	1.160842	0.843026	-0.702864	N	-1.15817	0.583004	0.149995
C	0.067623	1.51577	-0.196782	C	-0.044739	1.339513	-0.171875
C	-0.856682	0.576715	0.26969	C	0.987271	0.473188	-0.536569
C	-2.166369	0.820996	0.871054	C	2.345227	0.805102	-0.97019
C	-3.325759	0.188942	0.575587	C	3.491197	0.244013	-0.522422
C	-4.567228	0.553178	1.321504	C	4.796766	0.681343	-1.102898
N	-5.69526	-0.127989	0.953084	N	5.904439	0.066751	-0.585896
C	-5.766234	-1.352317	0.156195	C	5.941306	-1.150843	0.223773
C	-4.56209	-1.493211	-0.777916	C	4.636464	-1.359946	0.995817
N	-3.460235	-0.745466	-0.468839	N	3.542123	-0.679812	0.538186
C	0.031785	3.035273	-0.358562	C	-0.146562	2.855756	-0.005834
C	1.394387	3.585694	0.051241	C	-0.417902	3.126849	1.472994
C	2.26404	4.257271	-0.708857	C	-1.492847	3.711364	2.009625
C	-1.013466	3.711571	0.558633	C	-1.26805	3.40035	-0.921871
C	-0.319127	3.362196	-1.830096	C	1.165512	3.585066	-0.37215
C	-7.080945	-1.408045	-0.626433	C	7.145139	-1.13129	1.169442
C	3.179247	-1.368668	-1.660253	C	-3.144822	-1.711592	0.69634

C	4.339785	-0.570575	-1.038664	C	-4.073739	-1.171882	-0.408891
C	4.90445	-1.016076	0.334772	C	-5.417227	-0.580006	0.070076
O	5.954036	-0.049372	0.567146	C	-6.296331	-1.593161	0.811355
C	3.896258	-0.899068	1.491485	C	-5.202013	0.67266	0.932225
O	4.67407	-0.93793	2.703114	O	-4.314052	1.61464	0.325015
O	3.970682	0.813287	-0.996498	O	-4.41308	-2.202014	-1.353017
C	5.509814	-2.423068	0.26297	O	-6.103236	-0.086124	-1.097708
O	-4.575665	-2.27238	-1.724183	O	4.571576	-2.134044	1.943853
O	-4.594651	1.435649	2.179314	O	4.883313	1.563769	-1.956083
H	-0.077714	-4.110741	0.307953	H	0.539633	-4.253359	-0.775686
H	2.0628	-3.703483	-0.843452	H	-1.765843	-4.019309	0.089852
H	-1.585771	-2.23209	0.900658	H	1.975464	-2.263221	-1.12826
H	2.050467	1.270328	-0.953373	H	-2.046413	0.982236	0.437909
H	-2.265865	1.578101	1.640782	H	2.494831	1.567375	-1.726652
H	-6.51661	0.119997	1.494897	H	6.773725	0.36111	-1.01879
H	-5.721636	-2.231136	0.822434	H	6.032597	-2.031284	-0.435533
H	-2.656353	-0.901082	-1.070933	H	2.677224	-0.880828	1.032718
H	1.6456	3.406954	1.098732	H	0.385606	2.796712	2.133897
H	3.199783	4.623848	-0.294084	H	-1.56405	3.862726	3.083942
H	2.085827	4.472728	-1.758932	H	-2.336632	4.05422	1.417343
H	-2.033397	3.413066	0.300894	H	-1.045178	3.161134	-1.967334
H	-0.940112	4.798092	0.440513	H	-1.335397	4.490151	-0.828801
H	-0.840241	3.477012	1.61474	H	-2.248917	2.981801	-0.677981
H	0.406375	2.931958	-2.52814	H	2.016755	3.22649	0.214135
H	-0.351658	4.44671	-1.987295	H	1.042429	4.6546	-0.170359
H	-1.304595	2.953109	-2.075733	H	1.406285	3.47026	-1.433683
H	-7.93329	-1.385797	0.062996	H	8.076637	-1.060949	0.59534
H	-7.160117	-0.555384	-1.308902	H	7.087928	-0.275467	1.850195
H	-7.125887	-2.330221	-1.210048	H	7.166266	-2.049318	1.76088
H	3.568937	-2.356509	-1.929657	H	-3.536815	-2.670519	1.052407
H	2.96218	-0.863494	-2.61256	H	-3.152769	-1.024422	1.552683
H	5.185073	-0.671418	-1.73705	H	-3.556955	-0.369476	-0.947278
H	6.000039	-2.667651	1.210388	H	-7.25811	-1.133768	1.062777
H	6.258193	-2.473814	-0.535158	H	-5.822336	-1.93673	1.738571
H	4.740323	-3.180678	0.076318	H	-6.484683	-2.467372	0.181105
H	3.18152	-1.726954	1.470393	H	-4.763502	0.420701	1.900392
H	3.352651	0.049599	1.420742	H	-6.182277	1.134552	1.111348
H	4.099323	-0.744745	3.458148	H	-4.689807	1.820855	-0.548738
H	4.696481	1.270429	-0.533414	H	-3.600111	-2.488868	-1.800028
H	6.129639	-0.054218	1.525623	H	-6.01901	-0.77052	-1.785042
Free Energy = -1473.192394 Hartree				Free Energy = -1473.192391 Hartree			

conf. 2b-7	X axis(Å)	Y axis(Å)	Z axis(Å)
C	0.523788	-3.085125	0.015244
C	1.756855	-2.792996	-0.602877
C	2.131067	-1.489896	-0.940818
C	1.18732	-0.489307	-0.642096
C	-0.083636	-0.756352	-0.06519
C	-0.399341	-2.082758	0.290188
N	1.276985	0.875309	-0.821146
C	0.137972	1.497439	-0.364613
C	-0.755428	0.52101	0.088707
C	-2.074176	0.773699	0.652
C	-3.2111	0.061127	0.471915
C	-4.454993	0.483215	1.179191
N	-5.558119	-0.292494	0.9368
C	-5.565717	-1.641544	0.371209
C	-4.394647	-1.850421	-0.592466
N	-3.325638	-1.014691	-0.43087
C	-0.00271	3.018423	-0.40558
C	-1.313942	3.37728	-1.096758
C	-2.290941	4.15817	-0.630162
C	1.1321	3.655591	-1.249805
C	0.093109	3.581543	1.032129
C	-6.903967	-1.928187	-0.313041
C	3.43738	-1.22254	-1.666519
C	4.527982	-0.342885	-1.027949
C	5.073205	-0.723432	0.372652
O	6.051229	0.313362	0.616142
C	4.021852	-0.647563	1.494144
O	4.761022	-0.610336	2.72935
O	4.086032	1.023304	-1.02773
C	5.769065	-2.089573	0.351698
O	-4.400711	-2.747922	-1.428126
O	-4.511013	1.479478	1.900354
H	0.290439	-4.115299	0.271
H	2.437776	-3.610821	-0.830009
H	-1.340442	-2.322916	0.774753
H	2.152645	1.325493	-1.072372
H	-2.210636	1.650131	1.276116
H	-6.378001	-0.008186	1.462714
H	-5.4203	-2.384104	1.175467
H	-2.546063	-1.193499	-1.057379
H	-1.414603	2.952693	-2.097042
H	-3.173195	4.362713	-1.230846

H	-2.272446	4.601608	0.361564
H	2.1203	3.475171	-0.808861
H	0.983301	4.739032	-1.292131
H	1.136731	3.27725	-2.278248
H	-0.703608	3.204724	1.679751
H	0.034161	4.676055	1.021242
H	1.052412	3.299932	1.480192
H	-7.723995	-1.850067	0.41073
H	-7.082759	-1.214834	-1.124225
H	-6.899138	-2.937849	-0.72961
H	3.897733	-2.18841	-1.902085
H	3.220277	-0.752392	-2.636692
H	5.398915	-0.408471	-1.697869
H	6.241472	-2.282181	1.319876
H	6.545911	-2.107864	-0.420168
H	5.05676	-2.899244	0.157265
H	3.36213	-1.5199	1.469865
H	3.420613	0.262078	1.385527
H	4.151105	-0.441956	3.462456
H	4.777337	1.519968	-0.551375
H	6.193518	0.341694	1.579791
Free Energy = -1473.192346 Hartree			

Table S18. The Cartesian coordinates of the lowest energy conformers (Conf. **1-8**) for isomer **2c**.

conf. 2c-1	X axis(Å)	Y axis(Å)	Z axis(Å)	conf. 2c-2	X axis(Å)	Y axis(Å)	Z axis(Å)
C	-0.117872	-3.01773	-0.994049	C	-0.190752	-2.817423	-1.60481
C	-1.446486	-2.80441	-0.573995	C	1.191757	-2.600094	-1.431739
C	-1.888359	-1.566938	-0.10132	C	1.710903	-1.363477	-1.042405
C	-0.921011	-0.544991	-0.066464	C	0.762875	-0.350777	-0.799783
C	0.436299	-0.741398	-0.438109	C	-0.635655	-0.544291	-0.956534
C	0.827876	-2.001496	-0.931928	C	-1.109631	-1.798226	-1.387062
N	-1.05894	0.778947	0.298194	N	0.951143	0.968727	-0.451335
C	0.136014	1.444527	0.153421	C	-0.256799	1.628221	-0.369326
C	1.109109	0.533733	-0.273221	C	-1.278887	0.726074	-0.674848
C	2.505457	0.853021	-0.529318	C	-2.717846	0.978292	-0.733668
C	3.598237	0.104313	-0.245485	C	-3.690069	0.23053	-0.163091
C	4.951837	0.617065	-0.605874	C	-5.119191	0.615335	-0.35654
N	5.993735	-0.219621	-0.299965	N	-6.038527	-0.173432	0.276751
C	5.897262	-1.650307	-0.011071	C	-5.816184	-1.421962	1.002637
C	4.574535	-1.986828	0.682761	C	-4.339025	-1.678473	1.293856
N	3.550261	-1.106985	0.4749	N	-3.428763	-0.869649	0.676213

C	0.260813	2.933451	0.472437	C	-0.256189	3.072237	0.130547
C	0.66428	3.659852	-0.807938	C	0.9463	3.785256	-0.478692
C	1.739231	4.428782	-1.001372	C	1.963457	4.36184	0.167899
C	1.271498	3.137673	1.623369	C	-0.213286	3.057945	1.677888
C	-1.101655	3.522693	0.916936	C	-1.512467	3.855378	-0.320947
C	7.094838	-2.110494	0.821742	C	-6.429136	-2.629611	0.270107
C	-3.315611	-1.391504	0.384803	C	3.210614	-1.144558	-0.97143
C	-4.170395	-0.355658	-0.368377	C	3.778336	-0.771326	0.409428
C	-5.702199	-0.597214	-0.299138	C	5.285602	-1.090229	0.602264
C	-6.132813	-1.793509	-1.156331	C	5.541463	-2.596283	0.737942
C	-6.197745	-0.726353	1.151031	C	6.150367	-0.461954	-0.503414
O	-7.626845	-0.590461	1.107329	O	7.508656	-0.51095	-0.037902
H	-3.900689	-0.360112	-1.434374	H	3.239063	-1.326101	1.191151
O	-6.259398	0.610978	-0.856727	O	5.611701	-0.441086	1.848352
O	4.433439	-3.01065	1.343328	O	-4.002987	-2.591334	2.041238
O	5.139704	1.728844	-1.100182	O	-5.459436	1.593589	-1.022696
H	0.168952	-3.997311	-1.367125	H	-0.536797	-3.796156	-1.926477
H	-2.15134	-3.632334	-0.61628	H	1.880959	-3.418213	-1.63114
H	1.843995	-2.177584	-1.270656	H	-2.169547	-1.965502	-1.554724
H	-1.968897	1.202568	0.458502	H	1.843025	1.333348	-0.126406
H	2.732191	1.817869	-0.973792	H	-3.087896	1.834594	-1.286883
H	6.89806	0.132369	-0.596407	H	-6.999479	0.108024	0.113657
H	5.888681	-2.223205	-0.955268	H	-6.291893	-1.338286	1.988484
H	2.666185	-1.370951	0.900397	H	-2.45674	-1.0867	0.880048
H	-0.036884	3.521267	-1.632976	H	0.930845	3.825218	-1.569407
H	2.489224	4.598409	-0.233899	H	2.048506	4.36143	1.251336
H	1.915453	4.908017	-1.960879	H	2.756676	4.86607	-0.378448
H	0.933148	2.600572	2.516388	H	-1.098086	2.545111	2.06859
H	1.353289	4.201069	1.876781	H	-0.209985	4.08016	2.074338
H	2.267229	2.766765	1.365928	H	0.67226	2.536619	2.057407
H	-0.977428	4.587095	1.140048	H	-1.413683	4.900491	-0.007882
H	-1.476715	3.029379	1.821766	H	-2.423515	3.454181	0.131193
H	-1.862623	3.439139	0.1319	H	-1.630265	3.839181	-1.410212
H	7.011796	-3.179031	1.032209	H	-6.276082	-3.53802	0.859636
H	7.134853	-1.56875	1.772457	H	-5.970015	-2.761023	-0.715929
H	8.028275	-1.930897	0.274997	H	-7.505953	-2.478745	0.130728
H	-3.319116	-1.108314	1.447323	H	3.505548	-0.351227	-1.673606
H	-3.801928	-2.370083	0.320492	H	3.691081	-2.064868	-1.318236
O	-3.879252	0.941066	0.178129	O	3.567263	0.633794	0.604998
H	-7.221885	-1.898864	-1.131272	H	6.60215	-2.778647	0.937006
H	-5.823668	-1.638906	-2.195	H	4.958569	-3.000423	1.571825
H	-5.696876	-2.73277	-0.797789	H	5.273006	-3.142025	-0.173545
H	-5.755422	0.067859	1.763889	H	5.838888	0.576076	-0.669315

H	-5.920501	-1.703168	1.566999	H	6.049087	-1.025841	-1.439381
H	-7.96875	-0.465198	2.004672	H	8.063811	0.051449	-0.597593
H	-4.561962	1.538172	-0.181818	H	4.090258	0.87498	1.392018
H	-7.182057	0.664639	-0.549189	H	6.578301	-0.32134	1.858767
Free Energy = -1473.195526 Hartree				Free Energy = -1473.195426 Hartree			

conf. 2c-3	X axis(Å)	Y axis(Å)	Z axis(Å)	conf. 2c-4	X axis(Å)	Y axis(Å)	Z axis(Å)
C	0.071215	-2.833313	-1.505492	C	-0.181261	-2.866489	-1.43381
C	1.444489	-2.543522	-1.374767	C	1.203826	-2.637079	-1.308497
C	1.905542	-1.286505	-0.977223	C	1.724187	-1.390876	-0.952028
C	0.911179	-0.335017	-0.683595	C	0.776184	-0.382625	-0.694108
C	-0.480607	-0.604159	-0.783346	C	-0.626246	-0.589939	-0.79298
C	-0.895722	-1.874101	-1.229436	C	-1.101505	-1.852894	-1.1958
N	1.047861	0.990216	-0.321764	N	0.969074	0.94549	-0.374097
C	-0.187644	1.577191	-0.172243	C	-0.238227	1.593464	-0.248515
C	-1.174577	0.625039	-0.447461	C	-1.265846	0.680109	-0.503
C	-2.609597	0.869674	-0.441745	C	-2.693894	0.969313	-0.553812
C	-3.587367	0.061791	0.032638	C	-3.704173	0.226456	-0.044732
C	-5.011245	0.494978	-0.072412	C	-5.112731	0.685284	-0.226005
N	-5.933969	-0.385524	0.426794	N	-6.071531	-0.120241	0.325456
C	-5.712454	-1.787662	0.77441	C	-5.900001	-1.485904	0.817741
C	-4.256055	-2.059394	1.143385	C	-4.456624	-1.771408	1.226455
N	-3.335813	-1.1446	0.7183	N	-3.500785	-0.926395	0.738899
C	-0.328649	3.044038	0.231398	C	-0.276844	3.087169	0.076189
C	-0.961935	3.787331	-0.941933	C	1.048024	3.502287	0.704033
C	-2.096262	4.49293	-0.940939	C	1.939977	4.373128	0.223586
C	1.057848	3.687939	0.483289	C	-1.371143	3.399196	1.132401
C	-1.154081	3.159123	1.531792	C	-0.576398	3.871816	-1.222407
C	-6.164722	-2.751297	-0.340304	C	-6.380516	-2.541175	-0.197384
C	3.392057	-0.981762	-0.953557	C	3.223711	-1.159744	-0.934376
C	3.978704	-0.553919	0.403815	C	3.833395	-0.769157	0.423324
C	5.498558	-0.820888	0.572368	C	5.348704	-1.073743	0.570888
C	5.801978	-2.314247	0.743561	C	5.621166	-2.576228	0.713355
C	6.321353	-0.198103	-0.568141	C	6.173259	-0.450222	-0.567768
O	7.689279	-0.195663	-0.130092	O	7.545669	-0.482582	-0.143978
H	3.473826	-1.101143	1.212943	H	3.32458	-1.320674	1.227446
O	5.82925	-0.127062	1.792953	O	5.70832	-0.409173	1.799534
O	-3.941316	-3.071934	1.760814	O	-4.182908	-2.737638	1.930687
O	-5.346192	1.58465	-0.538077	O	-5.402269	1.736935	-0.797923
H	-0.231808	-3.822176	-1.839309	H	-0.530084	-3.850907	-1.734486
H	2.170729	-3.317125	-1.615749	H	1.893149	-3.451579	-1.521558
H	-1.948188	-2.101549	-1.366628	H	-2.163627	-2.033297	-1.32936

H	1.936058	1.373437	-0.010395	H	1.869332	1.316155	-0.080972
H	-2.965741	1.81887	-0.832044	H	-3.02734	1.881431	-1.040579
H	-6.893927	-0.078367	0.310801	H	-7.019414	0.202554	0.161967
H	-6.287454	-2.007574	1.68106	H	-6.48781	-1.592047	1.736712
H	-2.3719	-1.365555	0.952306	H	-2.546641	-1.159359	1.000644
H	-0.388493	3.716738	-1.86805	H	1.248314	3.036719	1.671469
H	-2.728736	4.59322	-0.063222	H	1.814496	4.880274	-0.729214
H	-2.443516	4.989246	-1.843455	H	2.84038	4.614142	0.783275
H	1.588095	3.19178	1.305653	H	-2.371784	3.158879	0.765733
H	0.921407	4.738306	0.759513	H	-1.345005	4.466103	1.381472
H	1.693537	3.661407	-0.40938	H	-1.202992	2.828068	2.052531
H	-1.247014	4.208991	1.833364	H	-0.613558	4.949016	-1.022087
H	-0.65319	2.616491	2.341245	H	-1.544707	3.573729	-1.635317
H	-2.158702	2.742578	1.42085	H	0.184351	3.684755	-1.987864
H	-6.011265	-3.787473	-0.023426	H	-6.263269	-3.544458	0.223361
H	-5.601379	-2.572518	-1.263031	H	-5.807773	-2.474689	-1.129147
H	-7.229584	-2.604946	-0.555205	H	-7.439002	-2.3819	-0.433538
H	3.622806	-0.185204	-1.675916	H	3.48941	-0.371134	-1.653306
H	3.913792	-1.879046	-1.300993	H	3.699061	-2.079961	-1.288367
O	3.731488	0.851585	0.569777	O	3.616639	0.635876	0.61228
H	6.871828	-2.460203	0.922394	H	6.688965	-2.748183	0.881238
H	5.250353	-2.711387	1.601649	H	5.067726	-2.976701	1.568787
H	5.528959	-2.892811	-0.146082	H	5.329177	-3.132958	-0.184143
H	5.976775	0.825851	-0.755045	H	5.847928	0.583291	-0.735377
H	6.21724	-0.789859	-1.486432	H	6.048324	-1.02543	-1.493961
H	8.21733	0.364621	-0.717487	H	8.0784	0.07784	-0.726965
H	4.267472	1.124761	1.337956	H	4.162498	0.888836	1.379838
H	6.792017	0.020514	1.781444	H	6.673843	-0.282278	1.77867
Free Energy = -1473.195073 Hartree				Free Energy = -1473.194688 Hartree			

conf. 2c-5	X axis(Å)	Y axis(Å)	Z axis(Å)	conf. 2c-6	X axis(Å)	Y axis(Å)	Z axis(Å)
C	0.116372	-3.034761	-1.017585	C	-5.328825	1.536332	-0.790468
C	-1.208181	-2.851758	-0.570447	C	-0.223037	-3.941672	-1.597297
C	-1.690548	-1.61178	-0.146028	C	2.179267	-3.429067	-1.389269
C	-0.767022	-0.548587	-0.189608	C	-1.93681	-2.185826	-1.269696
C	0.583573	-0.71327	-0.596485	C	1.948193	1.370805	-0.149928
C	1.019045	-1.978792	-1.03328	C	-2.959171	1.76203	-1.02042
N	-0.937159	0.78427	0.117355	N	6.856728	-2.372408	1.144861
C	0.23033	1.486456	-0.100982	C	-6.893271	-0.048042	0.169511
C	1.217751	0.590607	-0.519099	C	-6.312531	-1.86749	1.696752
C	2.614593	0.873025	-0.843732	C	-2.385124	-1.287908	0.982058
C	3.698563	0.179659	-0.425263	C	-1.09985	2.752943	1.947836

C	5.056757	0.597852	-0.882291	C	-2.675809	4.669687	0.120326
N	6.09639	-0.148463	-0.397517	N	5.22655	-2.55149	1.82616
C	6.019794	-1.453122	0.258739	C	-3.022025	4.213925	1.874823
C	4.658724	-1.679681	0.921097	C	0.065228	3.566688	-2.172817
N	3.634726	-0.87761	0.502823	N	5.523101	-2.895546	0.106069
C	0.247797	2.97147	0.25977	C	-0.743876	4.857738	-1.265728
C	-1.041209	3.600342	-0.259548	C	-1.657153	3.430433	-1.790099
C	-1.991657	4.213314	0.451982	C	0.909616	4.731574	0.603071
C	1.413589	3.735572	-0.411048	C	1.829404	3.557312	-0.347732
C	0.391719	3.102031	1.795237	C	1.378737	3.191394	1.33639
C	7.158059	-1.612922	1.270562	C	-6.014817	-3.768827	0.131057
C	-3.108006	-1.478868	0.380007	C	-5.586686	-2.647173	-1.188004
C	-4.039294	-0.540798	-0.407323	C	-7.224225	-2.627696	-0.501692
C	-5.555554	-0.828842	-0.240355	C	3.646852	-0.329404	-1.687328
C	-5.979217	-2.114019	-0.962058	C	3.924176	-1.983342	-1.155573
C	-5.977491	-0.838623	1.238055	C	3.740695	0.908424	0.454749
O	-7.411837	-0.75757	1.254967	O	6.001218	0.751474	-0.834319
H	-3.822352	-0.625122	-1.482284	H	0.080851	-2.931597	-1.335568
O	-6.184635	0.301299	-0.878739	O	6.231488	-0.9246	-1.418385
O	4.494015	-2.567669	1.750561	O	8.237301	0.273151	-0.738945
O	5.245082	1.57306	-1.609548	O	4.270995	1.248139	1.199979
H	0.433226	-4.02088	-1.346594	H	1.453877	-2.637046	-1.214592
H	-1.878045	-3.709069	-0.548458	H	1.916391	-1.354765	-0.90992
H	2.035974	-2.126681	-1.384422	H	0.922834	-0.380494	-0.699036
H	-1.853506	1.20178	0.267456	H	-0.469198	-0.653039	-0.786847
H	2.853726	1.713415	-1.485912	H	-0.884946	-1.951562	-1.140174
H	7.003619	0.141114	-0.747777	H	1.060636	0.968131	-0.438273
H	6.111839	-2.253287	-0.49573	H	-0.174367	1.567556	-0.341404
H	2.730424	-1.089501	0.915746	H	-1.161902	0.599645	-0.546326
H	-1.156324	3.541863	-1.343583	H	-2.597386	0.84339	-0.571198
H	-1.948266	4.309726	1.533591	H	-3.582494	0.068559	-0.059744
H	-2.857788	4.652157	-0.0373	H	-5.00336	0.491801	-0.225272
H	2.385467	3.390844	-0.047585	H	-5.936083	-0.343113	0.331118
H	1.32421	4.801332	-0.17437	H	-5.723733	-1.716106	0.784992
H	1.393946	3.630751	-1.501498	H	-4.2737	-1.962871	1.194376
H	0.405653	4.15682	2.094287	H	-3.344177	-1.086108	0.713568
H	1.332092	2.644639	2.119747	H	-0.305541	3.066979	-0.084291
H	-0.425941	2.601714	2.324773	H	-1.319439	3.300476	1.029682
H	7.094133	-2.593234	1.748062	H	-2.382219	4.107852	1.002821
H	7.098016	-0.839655	2.043593	H	-0.690886	3.771844	-1.407211
H	8.128027	-1.52945	0.766086	H	1.040027	3.662867	0.406404
H	-3.089966	-1.125776	1.42117	H	-6.162498	-2.758357	-0.262356
H	-3.543011	-2.48307	0.396763	H	3.404334	-1.055314	-0.897514

O	-3.766651	0.800671	0.020111	O	6.783097	0.174493	1.772057
H	-7.06295	-2.246181	-0.884892	H	3.980431	-0.508035	0.420721
H	-5.713022	-2.051908	-2.022233	H	5.496981	-0.765989	0.629177
H	-5.502283	-3.002404	-0.532935	H	5.789788	-2.238224	0.94129
H	-5.538167	0.024982	1.750835	H	6.334529	-0.254962	-0.554979
H	-5.643318	-1.761806	1.72849	H	7.699001	-0.226018	-0.107399
H	-7.714378	-0.55293	2.151801	H	3.463742	-0.975531	1.271333
H	-4.463541	1.357469	-0.373018	H	5.818934	0.036839	1.783814
H	-7.093925	0.349612	-0.533148	H	-3.971577	-2.926052	1.892038
Free Energy = -1473.19461 Hartree				Free Energy = -1473.19434 Hartree			

conf. 2c-7	X axis(Å)	Y axis(Å)	Z axis(Å)	conf. 2c-8	X axis(Å)	Y axis(Å)	Z axis(Å)
C	0.080924	-2.931522	-1.335602	C	-0.151163	-3.069712	-0.842298
C	1.453939	-2.636919	-1.214635	C	-1.47616	-2.823176	-0.428806
C	1.916398	-1.354618	-0.90997	C	-1.910018	-1.55512	-0.036693
C	0.922821	-0.380377	-0.699083	C	-0.938706	-0.537187	-0.077546
C	-0.469208	-0.65298	-0.786896	C	0.415233	-0.763811	-0.443535
C	-0.884907	-1.95152	-1.140211	C	0.799091	-2.055798	-0.853374
N	1.060557	0.968263	-0.438341	N	-1.068689	0.808522	0.197828
C	-0.174476	1.567621	-0.341434	C	0.127771	1.458376	-0.00023
C	-1.161956	0.599661	-0.546377	C	1.094083	0.516746	-0.368584
C	-2.597466	0.843355	-0.571207	C	2.489725	0.803558	-0.667739
C	-3.582496	0.068447	-0.059715	C	3.579806	0.068159	-0.345124
C	-5.003395	0.491618	-0.225134	C	4.928448	0.531722	-0.781392
N	-5.936001	-0.343203	0.331631	N	5.972503	-0.275777	-0.411174
C	-5.723544	-1.716363	0.784965	C	5.878089	-1.668868	0.024946
C	-4.273532	-1.963021	1.194457	C	4.559561	-1.935516	0.75595
N	-3.34408	-1.086172	0.713645	N	3.534809	-1.076731	0.476077
C	-0.305723	3.067044	-0.084327	C	0.246181	2.970253	0.184067
C	-1.319695	3.300477	1.02959	C	1.421662	3.26778	1.109042
C	-2.382498	4.10782	1.002709	C	2.451427	4.084569	0.875418
C	-0.691016	3.771928	-1.407254	C	-1.018628	3.542643	0.876668
C	1.03979	3.663006	0.406445	C	0.382622	3.647234	-1.199924
C	-6.162026	-2.758305	-0.262818	C	7.080317	-2.040964	0.894592
C	3.404326	-1.055101	-0.897549	C	-3.332537	-1.341794	0.447844
C	3.980456	-0.508196	0.420833	C	-4.193039	-0.362747	-0.371607
C	5.497039	-0.766043	0.629094	C	-5.724723	-0.591326	-0.263534
C	5.790044	-2.238365	0.940628	C	-6.171331	-1.847234	-1.021551
C	6.334476	-0.254458	-0.554897	C	-6.200367	-0.60722	1.198846
O	7.699004	-0.225666	-0.107419	O	-7.629345	-0.467516	1.164455
H	3.463873	-0.976029	1.271323	H	-3.937272	-0.45068	-1.43742
O	5.81895	0.036397	1.78402	O	-6.285698	0.573367	-0.903887

O	-3.971333	-2.926179	1.892111	O	4.422097	-2.893862	1.509149
O	-5.328983	1.536066	-0.790398	O	5.111244	1.583444	-1.394707
H	-0.222921	-3.941615	-1.59731	H	0.129278	-4.073136	-1.151401
H	2.179362	-3.428906	-1.389321	H	-2.184498	-3.64903	-0.41101
H	-1.936762	-2.185814	-1.26975	H	1.812816	-2.258525	-1.184261
H	1.948058	1.370945	-0.14986	H	-1.975659	1.244836	0.33798
H	-2.959332	1.761967	-1.020411	H	2.729818	1.724795	-1.187476
H	-6.893218	-0.048249	0.169988	H	6.871757	0.037849	-0.761318
H	-6.312441	-1.868146	1.696587	H	5.865518	-2.335799	-0.855324
H	-2.38501	-1.287911	0.982118	H	2.655649	-1.295739	0.935947
H	-1.100134	2.752907	1.94773	H	1.363751	2.75891	2.072746
H	-2.676061	4.669689	0.120225	H	2.588438	4.609657	-0.065888
H	-3.022358	4.213824	1.87468	H	3.221817	4.236955	1.626685
H	0.06513	3.566764	-2.172823	H	-1.917056	3.401443	0.263247
H	-0.743994	4.857816	-1.265736	H	-0.888975	4.618777	1.028711
H	-1.657272	3.430539	-1.79018	H	-1.190151	3.083236	1.856816
H	0.909301	4.731708	0.603091	H	0.428364	4.737065	-1.091213
H	1.829207	3.557475	-0.347646	H	-0.485933	3.404992	-1.82204
H	1.378454	3.191568	1.336465	H	1.278978	3.320648	-1.734458
H	-6.01424	-3.7689	0.130243	H	6.998135	-3.081759	1.215443
H	-5.586127	-2.646699	-1.18836	H	7.125914	-1.402826	1.78325
H	-7.223745	-2.627715	-0.502222	H	8.010726	-1.919805	0.327028
H	3.646777	-0.328934	-1.687144	H	-3.326032	-0.982079	1.48694
H	3.924219	-1.98302	-1.155911	H	-3.820687	-2.321381	0.459395
O	3.740537	0.908229	0.45534	O	-3.889987	0.971033	0.069172
H	6.857009	-2.372498	1.144094	H	-7.26053	-1.944059	-0.976216
H	5.226852	-2.552054	1.825372	H	-5.873843	-1.774268	-2.07256
H	5.523423	-2.895371	0.105135	H	-5.735908	-2.759005	-0.597438
H	6.001106	0.752087	-0.833752	H	-5.746202	0.229245	1.743049
H	6.231428	-0.923715	-1.418596	H	-5.92102	-1.550884	1.684355
H	8.2371	0.274336	-0.738479	H	-7.958183	-0.272429	2.054174
H	4.270822	1.247768	1.200659	H	-4.57661	1.541018	-0.325612
H	6.783112	0.174046	1.772333	H	-7.203315	0.654752	-0.587331
Free Energy = -1473.194339 Hartree				Free Energy = -1473.194257 Hartree			

Table S19. The Cartesian coordinates of the lowest energy conformers (Conf. **1-7**) for isomer **2d**.

conf. 2d-1	X axis(Å)	Y axis(Å)	Z axis(Å)	conf. 2d-2	X axis(Å)	Y axis(Å)	Z axis(Å)
C	-0.130149	-2.819912	-1.887208	C	0.01136	-3.19906	-0.600648
C	-1.517818	-2.615139	-1.740188	C	1.347304	-3.061197	-0.169358
C	-2.03825	-1.414255	-1.254359	C	1.877686	-1.825024	0.204626
C	-1.099677	-0.426848	-0.896654	C	1.001823	-0.724323	0.137429

C	0.302133	-0.615185	-1.013348	C	-0.359395	-0.843687	-0.245824
C	0.782209	-1.830836	-1.540949	C	-0.844491	-2.105399	-0.64527
N	-1.304637	0.86716	-0.445433	N	1.238214	0.615065	0.39994
C	-0.093376	1.509343	-0.273165	C	0.08749	1.352047	0.194088
C	0.935058	0.622813	-0.602202	C	-0.937571	0.484005	-0.189426
C	2.358734	0.930899	-0.575993	C	-2.305999	0.873496	-0.506344
C	3.368081	0.139965	-0.141899	C	-3.448155	0.23979	-0.151245
C	4.77287	0.640675	-0.205238	C	-4.761286	0.796739	-0.594152
N	5.726612	-0.236752	0.239221	N	-5.86586	0.095324	-0.192437
C	5.562113	-1.677696	0.430293	C	-5.899337	-1.253678	0.369017
C	4.123379	-2.027232	0.819224	C	-4.579434	-1.627138	1.039121
N	3.168629	-1.11274	0.472486	N	-3.48534	-0.881958	0.700784
C	-0.006845	2.960114	0.200743	C	0.044643	2.864037	0.414555
C	0.622432	3.778536	-0.924352	C	-0.270153	3.523999	-0.925621
C	1.724175	4.531144	-0.862879	C	-1.291289	4.335795	-1.212822
C	0.79568	3.034799	1.519002	C	1.418324	3.407576	0.876759
C	-1.411723	3.556958	0.458539	C	-0.994124	3.199536	1.508266
C	6.55939	-2.198183	1.467218	C	-6.282225	-2.318088	-0.677688
C	-3.523604	-1.171564	-1.120991	C	3.316604	-1.660627	0.635806
C	-3.986208	-1.204612	0.34864	C	4.180878	-1.027146	-0.471843
C	-5.366835	-0.57382	0.631926	C	5.54339	-0.459724	-0.018633
O	-5.56786	-0.612995	2.05851	O	6.150203	0.159704	-1.169944
C	-5.375246	0.92308	0.291512	C	5.361796	0.694308	0.977416
O	-4.244622	1.620296	0.822411	O	4.389039	1.651392	0.54943
H	-3.26358	-0.651747	0.959619	H	3.630475	-0.189916	-0.915749
C	-6.518949	-1.287229	-0.084503	C	6.480435	-1.522781	0.565131
O	3.841677	-3.088064	1.366057	O	-4.514036	-2.573935	1.815977
O	5.062721	1.775345	-0.583472	O	-4.858365	1.837467	-1.244393
H	0.227124	-3.763663	-2.290385	H	-0.352915	-4.179766	-0.894597
H	-2.206944	-3.401514	-2.040727	H	1.983404	-3.942228	-0.115721
H	1.845528	-1.991224	-1.688761	H	-1.867147	-2.223097	-0.989722
H	-2.20186	1.250287	-0.162172	H	2.144334	1.015654	0.622268
H	2.673299	1.916382	-0.906971	H	-2.459346	1.790313	-1.068151
H	6.675071	0.112217	0.148241	H	-6.739767	0.481219	-0.53421
H	5.744779	-2.204059	-0.523098	H	-6.645963	-1.269426	1.171279
H	2.215911	-1.385571	0.696017	H	-2.617621	-1.171103	1.143666
H	0.071245	3.730239	-1.865195	H	0.452961	3.293972	-1.710484
H	2.332789	4.615019	0.03323	H	-2.058616	4.598184	-0.489893
H	2.067978	5.08619	-1.73182	H	-1.402252	4.759791	-2.207441
H	0.302476	2.433823	2.291207	H	1.736636	2.954524	1.822956
H	0.846232	4.069704	1.876607	H	1.34105	4.488332	1.031591
H	1.816216	2.660335	1.402873	H	2.205341	3.238003	0.134527
H	-1.303513	4.59799	0.778832	H	-1.022578	4.280536	1.687598

H	-1.945108	3.01714	1.248713	H	-0.718717	2.707266	2.44757
H	-2.037902	3.553291	-0.440641	H	-1.999944	2.867967	1.237596
H	6.426824	-3.273918	1.602028	H	-6.326207	-3.305128	-0.207359
H	6.407289	-1.702611	2.431708	H	-5.552254	-2.343119	-1.494501
H	7.586547	-2.009398	1.133141	H	-7.265797	-2.089733	-1.10417
H	-4.080084	-1.928012	-1.684843	H	3.741635	-2.634228	0.902987
H	-3.771122	-0.196867	-1.561276	H	3.355917	-1.035049	1.53693
O	-4.060173	-2.550911	0.846039	O	4.47524	-1.974331	-1.511912
H	-7.472292	-0.83363	0.206697	H	7.445998	-1.068866	0.812482
H	-6.541113	-2.345912	0.190026	H	6.65037	-2.321804	-0.162536
H	-6.420886	-1.219668	-1.174629	H	6.064631	-1.972553	1.474681
H	-6.306342	1.356641	0.679814	H	6.336904	1.17909	1.118645
H	-5.349862	1.084099	-0.78848	H	5.020526	0.329885	1.948931
H	-4.268087	1.494752	1.786757	H	4.697284	1.998342	-0.305434
H	-3.167432	-2.933028	0.838108	H	3.640429	-2.239087	-1.931715
H	-5.316791	-1.505236	2.35608	H	6.045847	-0.460101	-1.913321
Free Energy = -1473.195783 Hartree				Free Energy = -1473.195485 Hartree			

**conf. 2d-3**	**X axis(Å)**	**Y axis(Å)**	**Z axis(Å)**	**conf. 2d-4**	**X axis(Å)**	**Y axis(Å)**	**Z axis(Å)**
C	-0.14809	-2.94745	-1.738859	C	-0.148201	-2.947274	-1.739582
C	-1.534774	-2.738156	-1.588215	C	-1.53486	-2.738003	-1.58872
C	-2.054388	-1.510733	-1.173165	C	-2.054459	-1.510654	-1.173412
C	-1.115529	-0.4993	-0.889936	C	-1.115603	-0.499211	-0.890224
C	0.285153	-0.690542	-1.012243	C	0.285073	-0.690457	-1.01279
C	0.764253	-1.935211	-1.467234	C	0.764161	-1.93506	-1.467933
N	-1.319156	0.818033	-0.512692	N	-1.319157	0.818035	-0.51267
C	-0.106687	1.471287	-0.39057	C	-0.106621	1.471223	-0.390656
C	0.918582	0.572285	-0.684838	C	0.918563	0.57226	-0.685246
C	2.343895	0.881017	-0.73252	C	2.343927	0.880917	-0.733074
C	3.362801	0.124549	-0.263812	C	3.362706	0.124479	-0.26409
C	4.767705	0.597486	-0.435349	C	4.767696	0.597103	-0.435685
N	5.731285	-0.226164	0.083978	N	5.73108	-0.226217	0.084591
C	5.571193	-1.625846	0.477413	C	5.570884	-1.625791	0.478327
C	4.1351	-1.929058	0.910987	C	4.134588	-1.929143	0.911005
N	3.174715	-1.059677	0.476148	N	3.174403	-1.059577	0.476124
C	-0.031947	2.956755	-0.045575	C	-0.031759	2.956618	-0.045463
C	1.111743	3.196681	0.933171	C	1.11222	3.196385	0.932977
C	2.077604	4.113615	0.840587	C	2.07809	4.113292	0.840237
C	-1.324223	3.432299	0.669227	C	-1.323824	3.432083	0.669777
C	0.124961	3.761341	-1.358587	C	0.124807	3.761393	-1.358396
C	6.569635	-1.991393	1.578478	C	6.568602	-1.990867	1.580268
C	-3.539343	-1.260002	-1.047544	C	-3.539429	-1.260077	-1.047571

C	-3.989374	-1.154966	0.422371	C	-3.989231	-1.154883	0.422402
C	-5.368322	-0.502163	0.657466	C	-5.36805	-0.501907	0.657707
O	-5.550824	-0.394837	2.083205	O	-5.550241	-0.394486	2.08349
C	-5.3887	0.950963	0.163091	C	-5.38834	0.951224	0.163338
O	-4.243932	1.700959	0.58131	O	-4.243434	1.701092	0.581501
H	-3.263611	-0.545877	0.972917	H	-3.263313	-0.545886	0.972832
C	-6.527086	-1.288741	0.034239	C	-6.527012	-1.288393	0.034741
O	3.860488	-2.924402	1.572734	O	3.859673	-2.924828	1.572129
O	5.051679	1.670301	-0.967008	O	5.051891	1.6695	-0.968048
H	0.207964	-3.913787	-2.085711	H	0.207816	-3.913568	-2.086587
H	-2.224182	-3.544387	-1.829795	H	-2.224302	-3.5442	-1.830309
H	1.826659	-2.100443	-1.6167	H	1.826566	-2.100245	-1.617477
H	-2.217101	1.216268	-0.252114	H	-2.217081	1.216249	-0.25195
H	2.659037	1.824399	-1.165499	H	2.659153	1.824094	-1.166457
H	6.677448	0.101673	-0.080186	H	6.677276	0.101253	-0.080149
H	5.758074	-2.280421	-0.391751	H	5.758564	-2.280646	-0.390453
H	2.225654	-1.306475	0.742146	H	2.225224	-1.30649	0.741595
H	1.077835	2.560179	1.818842	H	1.078566	2.559746	1.818561
H	2.187994	4.77332	-0.015677	H	2.18824	4.773147	-0.015947
H	2.821878	4.217586	1.625467	H	2.822604	4.21712	1.624911
H	-2.213191	3.333237	0.037074	H	-2.212958	3.333117	0.037857
H	-1.21899	4.490067	0.929844	H	-1.218479	4.489807	0.930528
H	-1.497057	2.875994	1.598282	H	-1.496388	2.875627	1.598789
H	0.127042	4.838383	-1.154849	H	0.127047	4.838397	-1.154465
H	-0.71213	3.547347	-2.031878	H	-0.712514	3.547574	-2.031456
H	1.052387	3.50906	-1.882242	H	1.052045	3.509119	-1.882387
H	6.444079	-3.039241	1.860099	H	6.443177	-3.038716	1.861945
H	6.412032	-1.367893	2.46474	H	6.410146	-1.367236	2.466279
H	7.596349	-1.843105	1.223341	H	7.595525	-1.842283	1.225859
H	-4.099248	-2.066668	-1.532999	H	-4.099346	-2.066887	-1.532782
H	-3.790724	-0.331169	-1.576081	H	-3.79103	-0.331371	-1.576235
O	-4.054706	-2.448109	1.045558	O	-4.054604	-2.447997	1.0457
H	-7.477762	-0.80351	0.279966	H	-7.477595	-0.80297	0.280422
H	-6.548639	-2.309802	0.426185	H	-6.548711	-2.309386	0.426859
H	-6.437143	-1.344505	-1.057207	H	-6.437135	-1.344368	-1.056702
H	-6.308187	1.424005	0.531892	H	-6.307738	1.424369	0.532219
H	-5.396637	0.997909	-0.928094	H	-5.396365	0.998192	-0.927844
H	-4.247715	1.704132	1.553783	H	-4.246982	1.703908	1.553984
H	-3.161636	-2.829278	1.059047	H	-3.161576	-2.829277	1.058965
H	-5.295011	-1.251959	2.46724	H	-5.294568	-1.251664	2.467491
Free Energy = -1473.194405 Hartree				Free Energy = -1473.194399 Hartree			

conf. 2d-5	X axis(Å)	Y axis(Å)	Z axis(Å)	conf. 2d-6	X axis(Å)	Y axis(Å)	Z axis(Å)
C	0.024352	-3.246447	-0.47073	C	0.024444	-3.246393	-0.470608
C	1.359993	-3.091704	-0.043547	C	1.360051	-3.091605	-0.043314
C	1.892836	-1.839802	0.268805	C	1.892826	-1.839678	0.269043
C	1.020202	-0.741714	0.144038	C	1.020167	-0.741621	0.144125
C	-0.339333	-0.874618	-0.23895	C	-0.339304	-0.874553	-0.239038
C	-0.827764	-2.153401	-0.57412	C	-0.827682	-2.153369	-0.574165
N	1.259588	0.606302	0.346294	N	1.25947	0.606397	0.346571
C	0.112103	1.33732	0.102223	C	0.11204	1.337414	0.102179
C	-0.912083	0.457849	-0.250113	C	-0.912105	0.457926	-0.250213
C	-2.272686	0.826043	-0.631083	C	-2.272746	0.826003	-0.631169
C	-3.421154	0.216391	-0.258121	C	-3.421166	0.216321	-0.25811
C	-4.722023	0.71822	-0.79192	C	-4.722116	0.718091	-0.791803
N	-5.83772	0.064806	-0.343321	N	-5.837729	0.064635	-0.343064
C	-5.896542	-1.200454	0.385871	C	-5.896241	-1.200925	0.385604
C	-4.579923	-1.525245	1.08706	C	-4.579747	-1.525308	1.087197
N	-3.476018	-0.82753	0.686375	N	-3.47591	-0.827483	0.686535
C	0.112511	2.860622	0.204644	C	0.112334	2.860733	0.204644
C	-1.207318	3.333488	0.802699	C	-1.207511	3.333438	0.802821
C	-2.006802	4.296153	0.337592	C	-2.007156	4.295997	0.33777
C	0.375894	3.455033	-1.19969	C	0.375584	3.455224	-1.199685
C	1.22609	3.350329	1.170841	C	1.225937	3.350495	1.170754
C	-6.319313	-2.375944	-0.516204	C	-6.317961	-2.376389	-0.517053
C	3.330613	-1.650765	0.693652	C	3.330562	-1.650486	0.69395
C	4.18909	-1.050735	-0.437629	C	4.189107	-1.050914	-0.437545
C	5.528089	-0.416041	-0.003863	C	5.528005	-0.415909	-0.003955
O	6.132283	0.149244	-1.183968	O	6.132242	0.148917	-1.184246
C	5.300557	0.792494	0.915341	C	5.300264	0.792975	0.914757
O	4.316088	1.698979	0.407925	O	4.315637	1.699097	0.407025
H	3.618611	-0.256762	-0.932447	H	3.618596	-0.257201	-0.932746
C	6.487184	-1.412485	0.65647	C	6.487146	-1.411982	0.656877
O	-4.527377	-2.396607	1.948768	O	-4.527207	-2.396552	1.949028
O	-4.800783	1.675725	-1.561302	O	-4.801	1.675676	-1.561082
H	-0.342749	-4.239591	-0.715271	H	-0.342621	-4.239565	-0.715089
H	1.993172	-3.970999	0.055408	H	1.993229	-3.970891	0.05575
H	-1.849898	-2.285474	-0.91506	H	-1.849798	-2.285482	-0.915148
H	2.166915	1.014671	0.549383	H	2.166962	1.014862	0.548754
H	-2.420266	1.689203	-1.271347	H	-2.420422	1.689145	-1.271437
H	-6.701634	0.410923	-0.747376	H	-6.701725	0.410781	-0.746922
H	-6.634048	-1.097085	1.190774	H	-6.634145	-1.09817	1.190194
H	-2.614924	-1.081812	1.162559	H	-2.614785	-1.081638	1.162732
H	-1.469936	2.842001	1.740565	H	-1.469999	2.841908	1.740699
H	-1.82014	4.82169	-0.595027	H	-1.820631	4.821556	-0.594862

H	-2.908367	4.577567	0.874901	H	-2.908744	4.577272	0.875113
H	1.32742	3.083292	-1.595817	H	1.327165	3.083645	-1.595837
H	0.436492	4.548385	-1.152893	H	0.436025	4.548587	-1.152884
H	-0.413398	3.183095	-1.907868	H	-0.413667	3.183186	-1.907867
H	1.169427	4.440169	1.256405	H	1.169188	4.440327	1.256386
H	2.231316	3.097707	0.816301	H	2.231157	3.097992	0.816099
H	1.098707	2.927756	2.174285	H	1.098718	2.927852	2.174189
H	-6.384235	-3.294549	0.07457	H	-6.382748	-3.295197	0.073418
H	-5.598799	-2.522878	-1.328527	H	-5.596926	-2.522732	-1.329027
H	-7.301053	-2.175378	-0.960635	H	-7.299552	-2.176177	-0.961974
H	3.764114	-2.610378	0.995634	H	3.764046	-2.609967	0.996359
H	3.36436	-0.993519	1.572387	H	3.364212	-0.992849	1.572389
O	4.52435	-2.042899	-1.42209	O	4.52456	-2.043512	-1.421489
H	7.435131	-0.915385	0.888322	H	7.435048	-0.914689	0.888525
H	6.691544	-2.248696	-0.018646	H	6.691634	-2.248476	-0.017855
H	6.071036	-1.818591	1.586172	H	6.071034	-1.817723	1.586752
H	6.26077	1.309046	1.044142	H	6.260405	1.309716	1.043337
H	4.946438	0.48212	1.900897	H	4.946214	0.482923	1.900437
H	4.623751	1.981528	-0.470823	H	4.623327	1.981606	-0.471718
H	3.703619	-2.349562	-1.841008	H	3.703948	-2.350158	-1.840644
H	6.051762	-0.516597	-1.889654	H	6.051791	-0.5172	-1.889682
Free Energy = -1473.193969 Hartree				Free Energy = -1473.193966 Hartree			

conf. 2d-6	X axis(Å)	Y axis(Å)	Z axis(Å)
C	0.209817	-2.787932	-1.526618
C	1.569325	-2.42708	-1.432242
C	1.975117	-1.134447	-1.092637
C	0.93981	-0.220578	-0.821072
C	-0.438458	-0.561607	-0.88002
C	-0.798341	-1.867041	-1.267826
N	1.016936	1.122933	-0.514228
C	-0.243142	1.654143	-0.361321
C	-1.185999	0.644008	-0.574013
C	-2.632442	0.811725	-0.546679
C	-3.556912	-0.029045	-0.026481
C	-5.003004	0.321029	-0.132016
N	-5.870119	-0.575754	0.434076
C	-5.570716	-1.943597	0.852598
C	-4.094534	-2.12474	1.19751
N	-3.230637	-1.190343	0.703643
C	-0.443536	3.132455	-0.035668
C	-1.42502	3.261059	1.123831

C	-2.530114	4.008459	1.17268
C	-0.91673	3.872612	-1.309391
C	0.88615	3.780119	0.432375
C	-6.000728	-2.988402	-0.195817
C	3.44548	-0.759374	-1.098736
C	4.015184	-0.228084	0.22803
C	5.542563	-0.424218	0.409742
O	5.826429	0.324069	1.609039
C	5.877123	-1.898569	0.688701
O	7.233327	-1.927974	1.153594
H	3.521713	-0.733337	1.0711
C	6.368795	0.134444	-0.755749
O	-3.716031	-3.087748	1.857447
O	-5.403228	1.359774	-0.658421
H	-0.049863	-3.802335	-1.817715
H	2.328528	-3.173631	-1.657033
H	-1.840646	-2.150613	-1.374641
H	1.892759	1.561754	-0.242527
H	-3.057133	1.721209	-0.957826
H	-6.84658	-0.327933	0.313733
H	-6.112815	-2.140595	1.784521
H	-2.252662	-1.349058	0.929399
H	-1.140959	2.687621	2.007882
H	-2.888495	4.591288	0.32863
H	-3.139411	4.041403	2.072001
H	-0.180606	3.74544	-2.110527
H	-1.02277	4.94599	-1.113525
H	-1.877488	3.49662	-1.673143
H	0.706383	4.831019	0.679509
H	1.651488	3.749469	-0.352632
H	1.284481	3.290609	1.328885
H	-5.789438	-3.996802	0.173038
H	-5.468206	-2.834378	-1.141029
H	-7.076189	-2.905284	-0.391
H	3.641559	0.002651	-1.866036
H	4.008725	-1.649848	-1.398899
O	3.722501	1.176905	0.302786
H	7.436444	0.061356	-0.526576
H	6.115668	1.184844	-0.924175
H	6.185242	-0.422163	-1.681858
H	5.773699	-2.504417	-0.22077
H	5.200437	-2.288794	1.460762
H	7.43133	-2.801196	1.522699

H	4.210801	1.522311	1.071939
H	6.698637	0.03671	1.93074
Free Energy = -1473.19335 Hartree			

Table S20. The Cartesian coordinates of the lowest energy conformers (Conf. **1-6**) for isomer **3a**.

conf. 3a-1	X axis(Å)	Y axis(Å)	Z axis(Å)	conf. 3a-2	X axis(Å)	Y axis(Å)	Z axis(Å)
C	-0.024623	-2.769149	-1.758993	C	-0.024227	-2.768883	-1.759728
C	1.359429	-2.524891	-1.6641	C	1.359797	-2.524526	-1.664677
C	1.864589	-1.305178	-1.214675	C	1.864812	-1.304806	-1.21511
C	0.91627	-0.333514	-0.839795	C	0.916385	-0.333243	-0.840232
C	-0.483384	-0.563243	-0.905325	C	-0.483249	-0.563076	-0.905951
C	-0.949893	-1.79855	-1.395518	C	-0.94961	-1.798381	-1.396288
N	1.096929	0.975325	-0.419002	N	1.096895	0.975548	-0.419205
C	-0.127984	1.585137	-0.218003	C	-0.128112	1.585189	-0.218191
C	-1.138607	0.663889	-0.497904	C	-1.138605	0.663892	-0.498339
C	-2.569878	0.932217	-0.442513	C	-2.569939	0.932037	-0.442993
C	-3.544201	0.128672	0.044364	C	-3.544093	0.128346	0.043961
C	-4.963839	0.589034	-0.005239	C	-4.963821	0.588519	-0.005546
N	-5.886092	-0.284638	0.504133	N	-5.885859	-0.28488	0.504722
C	-5.680582	-1.693889	0.832251	C	-5.680171	-1.694054	0.833028
C	-4.215973	-2.003643	1.132088	C	-4.215482	-2.003627	1.13266
N	-3.292876	-1.098123	0.690917	N	-3.292535	-1.098445	0.69041
C	-0.247799	3.042238	0.229092	C	-0.24821	3.04221	0.229064
C	-0.898038	3.821276	-0.91203	C	-0.89842	3.8213	-0.912037
C	-2.025377	4.536478	-0.868615	C	-2.0259	4.536281	-0.868633
C	1.14101	3.678423	0.476555	C	1.140435	3.678606	0.476861
C	-1.050269	3.125634	1.546824	C	-1.050937	3.125279	1.546666
C	-6.207343	-2.637498	-0.266493	C	-6.206966	-2.637928	-0.265523
C	3.349725	-1.026391	-1.178982	C	3.349925	-1.025912	-1.179166
C	3.943123	-0.940732	0.236241	C	3.943139	-0.940737	0.236174
C	5.301082	-0.204525	0.361293	C	5.300878	-0.204217	0.361728
C	6.442359	-0.853655	-0.427395	C	6.442408	-0.852695	-0.427122
C	5.139615	1.26481	-0.076089	C	5.139073	1.265282	-0.074951
O	4.00834	1.899704	0.520051	O	4.00757	1.899574	0.521415
Cl	4.089488	-2.632719	0.952237	Cl	4.089896	-2.632995	0.951412
O	5.642392	-0.091572	1.752891	O	5.641989	-0.091831	1.753435
O	-3.897858	-3.036321	1.712288	O	-3.897116	-3.035917	1.713379
O	-5.291696	1.694184	-0.437642	O	-5.291862	1.693369	-0.438549
H	-0.369639	-3.729994	-2.131095	H	-0.369134	-3.729732	-2.131918
H	2.058199	-3.303332	-1.960718	H	2.058663	-3.302888	-1.961273
H	-2.013411	-1.987674	-1.502639	H	-2.013106	-1.987579	-1.503528

H	1.992407	1.398614	-0.191815	H	1.992289	1.398732	-0.191443
H	-2.922562	1.894561	-0.802138	H	-2.922781	1.894294	-0.802705
H	-6.843167	0.043511	0.428708	H	-6.843017	0.042986	0.429014
H	-6.218187	-1.907943	1.763126	H	-6.217617	-1.907998	1.764013
H	-2.325799	-1.346797	0.879942	H	-2.325389	-1.346976	0.879277
H	-0.342393	3.773306	-1.850328	H	-0.342656	3.773568	-1.850274
H	-2.384869	5.062493	-1.749215	H	-2.385392	5.062376	-1.749185
H	-2.639859	4.616438	0.023839	H	-2.640498	4.615959	0.023766
H	1.681168	3.16573	1.280578	H	1.68046	3.166033	1.281046
H	1.006563	4.722482	0.776657	H	1.005757	4.722662	0.776874
H	1.771931	3.672114	-0.418838	H	1.771608	3.672351	-0.418356
H	-2.060982	2.722872	1.442542	H	-2.061586	2.722423	1.44211
H	-1.126516	4.166882	1.880897	H	-1.127363	4.166453	1.880922
H	-0.540637	2.5546	2.330966	H	-0.541388	2.554155	2.330795
H	-7.277214	-2.464167	-0.430117	H	-5.683388	-2.465473	-1.212494
H	-5.683595	-2.46502	-1.213368	H	-6.063702	-3.679651	0.037234
H	-6.064291	-3.679276	0.036174	H	-7.276896	-2.464813	-0.429004
H	3.885201	-1.78753	-1.755302	H	3.885541	-1.786783	-1.755712
H	3.539815	-0.067712	-1.679908	H	3.539978	-0.067032	-1.679712
H	3.241501	-0.44905	0.908985	H	3.241286	-0.44955	0.909043
H	6.215269	-0.903009	-1.498652	H	6.215405	-0.901649	-1.498415
H	6.630273	-1.871721	-0.073571	H	6.630541	-1.870867	-0.073721
H	7.359264	-0.270319	-0.293299	H	7.359147	-0.269178	-0.292697
H	5.00197	1.348323	-1.155901	H	5.001504	1.349291	-1.154734
H	6.062066	1.794746	0.193579	H	6.061347	1.79535	0.195061
H	4.142693	1.876754	1.483063	H	4.141751	1.875899	1.484441
H	5.611874	-0.977256	2.154564	H	5.611593	-0.977702	2.154704
Free Energy = -1857.585457 Hartree				Free Energy = -1857.585447 Hartree			

**conf. 3a-3**	**X axis(Å)**	**Y axis(Å)**	**Z axis(Å)**	**conf. 3a-4**	**X axis(Å)**	**Y axis(Å)**	**Z axis(Å)**
C	-0.251953	-2.719663	-1.789251	C	0.020167	-3.151902	0.638643
C	1.140472	-2.527725	-1.695863	C	1.334997	-2.963612	0.169371
C	1.691455	-1.324427	-1.255965	C	1.809962	-1.709148	-0.211737
C	0.779808	-0.313519	-0.896874	C	0.899358	-0.638811	-0.116916
C	-0.627571	-0.490139	-0.957576	C	-0.444569	-0.811155	0.306367
C	-1.14063	-1.712398	-1.433117	C	-0.872273	-2.089708	0.714211
N	1.006669	0.991954	-0.495622	N	1.075629	0.710946	-0.379592
C	-0.191114	1.653074	-0.301841	C	-0.096779	1.402272	-0.135064
C	-1.236782	0.766	-0.56299	C	-1.075318	0.493686	0.273097
C	-2.663211	1.066624	-0.51517	C	-2.448874	0.827874	0.625858
C	-3.650621	0.298786	0.001191	C	-3.57494	0.145482	0.30993
C	-5.061608	0.783496	-0.069408	C	-4.896291	0.654297	0.783284

N	-5.999932	-0.045081	0.480894	N	-5.976742	-0.1126	0.434415
C	-5.822211	-1.434628	0.898059	C	-5.942929	-1.502181	-0.021442
C	-4.360077	-1.767912	1.18509	C	-4.657875	-1.800839	-0.798052
N	-3.42078	-0.907514	0.689665	N	-3.59575	-0.980935	-0.536915
C	-0.211928	3.132115	0.089722	C	-0.208372	2.912359	-0.343312
C	1.176106	3.559362	0.546564	C	-0.550065	3.550135	1.00107
C	1.975671	4.46788	-0.01831	C	-1.596469	4.3284	1.28963
C	-1.1645	3.361623	1.294883	C	-1.262534	3.208181	-1.433945
C	-0.699206	3.966371	-1.118477	C	1.138453	3.523928	-0.798534
C	-6.401085	-2.431848	-0.124172	C	-7.184181	-1.823228	-0.85563
C	3.186502	-1.109432	-1.191949	C	3.220806	-1.514556	-0.717897
C	3.763586	-1.143375	0.232973	C	4.153759	-0.7951	0.268919
C	5.178236	-0.535745	0.413193	C	5.432005	-0.164764	-0.339328
C	6.261831	-1.213103	-0.430852	C	6.3805	-1.174459	-0.992871
C	5.14264	0.97488	0.105058	C	5.042091	0.917696	-1.365838
O	4.094434	1.651633	0.793452	O	4.045423	1.815384	-0.876598
Cl	3.759497	-2.879597	0.857172	Cl	4.62336	-1.943885	1.63325
O	5.524643	-0.569748	1.807709	O	6.111114	0.584169	0.681962
O	-4.058311	-2.781891	1.804777	O	-4.57518	-2.750299	-1.569422
O	-5.366075	1.872699	-0.556554	O	-5.024782	1.709852	1.403051
H	-0.632975	-3.670256	-2.152481	H	-0.297838	-4.145457	0.942536
H	1.808966	-3.336171	-1.981289	H	2.006096	-3.81707	0.110262
H	-2.210756	-1.863285	-1.536012	H	-1.87915	-2.244665	1.088884
H	1.910373	1.402751	-0.284885	H	1.95124	1.143298	-0.659959
H	-3.015446	2.011992	-0.91664	H	-2.625095	1.744407	1.181226
H	-6.950221	0.299831	0.394918	H	-6.856535	0.231314	0.805005
H	-6.342937	-1.57277	1.852866	H	-5.923613	-2.18067	0.849709
H	-2.457269	-1.17428	0.872976	H	-2.736706	-1.227976	-1.019997
H	1.523656	3.062415	1.454246	H	0.176472	3.336073	1.787209
H	2.946156	4.695352	0.414033	H	-1.724534	4.741299	2.28684
H	1.70581	5.011631	-0.919377	H	-2.369708	4.570622	0.565883
H	-2.192674	3.077058	1.057546	H	-0.967801	2.73304	-2.376254
H	-1.155519	4.421676	1.572841	H	-1.339761	4.287646	-1.608224
H	-0.844099	2.776527	2.164567	H	-2.251897	2.830827	-1.161892
H	-0.050403	3.827337	-1.989966	H	1.926039	3.38706	-0.04948
H	-0.716771	5.032232	-0.863491	H	1.010097	4.600938	-0.945609
H	-1.712786	3.677615	-1.40892	H	1.485539	3.093864	-1.744946
H	-5.897007	-2.335003	-1.092195	H	-8.091173	-1.681354	-0.255987
H	-6.277283	-3.455289	0.242352	H	-7.238435	-1.171515	-1.733885
H	-7.469917	-2.240087	-0.273306	H	-7.145879	-2.861366	-1.19287
H	3.696135	-1.854512	-1.81123	H	3.65414	-2.481489	-0.992913
H	3.425989	-0.128739	-1.624956	H	3.186308	-0.916866	-1.638364
H	3.0935	-0.629018	0.921267	H	3.61546	-0.004078	0.789418

H	6.044528	-1.13802	-1.502647	H	5.888409	-1.725442	-1.802677
H	6.348735	-2.273544	-0.175547	H	6.740353	-1.901165	-0.258501
H	7.227888	-0.73508	-0.238595	H	7.247213	-0.648676	-1.406761
H	4.98363	1.162397	-0.958837	H	4.633432	0.475436	-2.27635
H	6.119941	1.391278	0.383347	H	5.95425	1.46689	-1.631786
H	4.233425	1.492271	1.74311	H	4.42133	2.26468	-0.10038
H	5.403787	-1.477988	2.13525	H	6.256286	0.002618	1.448428
Free Energy = -1857.584994 Hartree				Free Energy = -1857.584966 Hartree			

conf. 3a-5	X axis(Å)	Y axis(Å)	Z axis(Å)	conf. 3a-6	X axis(Å)	Y axis(Å)	Z axis(Å)
C	0.199262	-3.207306	-0.411391	C	-0.029953	-2.869637	-1.643225
C	-1.111933	-3.008493	0.066003	C	1.355646	-2.634265	-1.546601
C	-1.630914	-1.733809	0.289404	C	1.868986	-1.396202	-1.161325
C	-0.767898	-0.652231	0.024806	C	0.926782	-0.395128	-0.85419
C	0.569683	-0.828077	-0.412444	C	-0.474104	-0.614936	-0.920055
C	1.043811	-2.130965	-0.655589	C	-0.948737	-1.871108	-1.344349
N	-0.984418	0.708324	0.12386	N	1.116389	0.931558	-0.50078
C	0.156141	1.410187	-0.230199	C	-0.104877	1.561959	-0.340305
C	1.154973	0.493169	-0.558142	C	-1.120729	0.638736	-0.58343
C	2.523467	0.756689	-1.005261	C	-2.553433	0.916539	-0.581544
C	3.649685	0.176753	-0.532147	C	-3.538869	0.147342	-0.06461
C	4.969158	0.543153	-1.129697	C	-4.958004	0.592188	-0.196321
N	6.055117	-0.086858	-0.585618	N	-5.891004	-0.231522	0.373893
C	6.049009	-1.270559	0.273551	C	-5.692724	-1.602778	0.839269
C	4.739241	-1.398696	1.05509	C	-4.233196	-1.885973	1.186293
N	3.669409	-0.700181	0.568759	N	-3.301188	-1.024092	0.681722
C	0.115366	2.934512	-0.121418	C	-0.198563	3.049659	-0.009938
C	-0.129826	3.273305	1.347922	C	-1.329304	3.282457	0.985373
C	-1.161711	3.943525	1.868492	C	-2.303829	4.191337	0.904604
C	-0.98994	3.490588	-1.049858	C	-0.389445	3.838361	-1.327968
C	1.452185	3.597092	-0.524777	C	1.096783	3.554211	0.67985
C	7.255239	-1.256558	1.216172	C	-6.207669	-2.645961	-0.171379
C	-3.032732	-1.517634	0.812284	C	3.35636	-1.128884	-1.127155
C	-4.017095	-0.97089	-0.235142	C	3.930387	-0.921255	0.283093
C	-5.274115	-0.254011	0.320938	C	5.29192	-0.18487	0.363708
C	-6.153329	-1.136073	1.212072	C	6.44362	-0.915108	-0.334379
C	-4.856245	1.014146	1.093239	C	5.154121	1.23495	-0.219812
O	-3.958431	1.838502	0.350965	O	4.012627	1.933949	0.278312
Cl	-4.534026	-2.332628	-1.366548	Cl	4.05487	-2.542932	1.150599
O	-6.031708	0.279197	-0.778211	O	5.606171	0.058922	1.744667
O	4.648869	-2.128034	2.036103	O	-3.926279	-2.864347	1.859332
O	5.08633	1.385362	-2.019171	O	-5.27774	1.645103	-0.74804

H	0.552537	-4.220085	-0.584894	H	-0.381077	-3.846519	-1.964376
H	-1.742644	-3.872425	0.261355	H	2.049084	-3.435437	-1.790763
H	2.050062	-2.293888	-1.029714	H	-2.013192	-2.055592	-1.450214
H	-1.860623	1.153127	0.382726	H	2.016628	1.360671	-0.30477
H	2.699023	1.477827	-1.795692	H	-2.905039	1.844882	-1.01877
H	6.93381	0.15767	-1.030354	H	-6.846594	0.08151	0.238548
H	6.105747	-2.180394	-0.348703	H	-6.242936	-1.726465	1.779258
H	2.797502	-0.851372	1.068711	H	-2.337984	-1.255258	0.9091
H	0.657402	2.919528	2.016126	H	-1.276173	2.649321	1.872373
H	-1.214578	4.139762	2.936636	H	-3.036797	4.291965	1.700463
H	-1.986684	4.316416	1.267894	H	-2.432966	4.846588	0.047538
H	-0.789567	3.196362	-2.085746	H	0.438626	3.6289	-2.01382
H	-1.007061	4.585393	-1.004238	H	-0.403587	4.917586	-1.136349
H	-1.985638	3.128227	-0.778533	H	-1.322298	3.566166	-1.831506
H	2.293551	3.230025	0.070327	H	1.296081	3.005427	1.608394
H	1.371702	4.677845	-0.365733	H	0.975158	4.61097	0.937693
H	1.680323	3.430412	-1.582254	H	1.974643	3.469498	0.030544
H	8.187336	-1.246458	0.638829	H	-7.275449	-2.489106	-0.363176
H	7.232193	-0.370873	1.859725	H	-5.672643	-2.563047	-1.124021
H	7.242892	-2.148591	1.846376	H	-6.069013	-3.654531	0.229709
H	-3.424412	-2.449738	1.231812	H	3.892701	-1.942681	-1.625346
H	-2.992514	-0.798318	1.641317	H	3.562656	-0.219643	-1.707592
H	-3.508998	-0.275176	-0.902071	H	3.223557	-0.367766	0.899995
H	-5.603351	-1.494556	2.089837	H	6.23431	-1.074576	-1.398503
H	-6.517122	-2.007984	0.660232	H	6.621814	-1.8913	0.126116
H	-7.02011	-0.562148	1.555561	H	7.360727	-0.323371	-0.245706
H	-4.346269	0.768662	2.026918	H	5.046112	1.210946	-1.305708
H	-5.771646	1.568405	1.338067	H	6.072987	1.784312	0.021057
H	-4.403829	2.056168	-0.486456	H	4.123866	2.020594	1.240561
H	-6.200717	-0.43527	-1.416694	H	5.559389	-0.78464	2.227432
Free Energy = -1857.584487 Hartree				Free Energy = -1857.584243 Hartree			

Table S21. The Cartesian coordinates of the lowest energy conformers (Conf. **1-8**) for isomer **3b**.

conf. 3b-1	X axis(Å)	Y axis(Å)	Z axis(Å)	conf. 3b-2	X axis(Å)	Y axis(Å)	Z axis(Å)
C	0.401659	-3.278717	-0.479988	C	0.408932	-3.262814	-0.603157
C	-0.923921	-3.183461	-0.007237	C	-0.90212	-3.164565	-0.091021
C	-1.498402	-1.956525	0.320657	C	-1.470111	-1.93661	0.24423
C	-0.677943	-0.823531	0.16688	C	-0.657446	-0.802324	0.056492
C	0.668981	-0.89258	-0.270415	C	0.672998	-0.875438	-0.425354
C	1.201043	-2.149986	-0.620235	C	1.200159	-2.133439	-0.77778
N	-0.975575	0.510091	0.377186	N	-0.943893	0.53204	0.267708

C	0.11923	1.296247	0.08638	C	0.142857	1.317519	-0.070523
C	1.173879	0.467772	-0.308422	C	1.181317	0.483165	-0.49052
C	2.495973	0.897435	-0.748922	C	2.523805	0.862072	-0.930122
C	3.692085	0.347854	-0.4344	C	3.691174	0.314711	-0.520612
C	4.937855	0.929687	-1.01523	C	4.975426	0.813621	-1.09727
N	6.101063	0.30957	-0.647615	N	6.107539	0.213806	-0.617089
C	6.233832	-1.010164	-0.030746	C	6.191771	-1.03203	0.144419
C	4.998229	-1.372558	0.797418	C	4.901767	-1.314222	0.918282
N	3.849438	-0.694158	0.499197	N	3.782197	-0.650974	0.499556
C	0.032873	2.818369	0.205005	C	0.061198	2.819037	0.20067
C	-1.155469	3.199106	1.078766	C	-1.303418	3.321891	-0.257416
C	-2.193708	3.972762	0.74871	C	-2.225056	3.95362	0.475495
C	-0.065022	3.432539	-1.210746	C	1.114287	3.625015	-0.59773
C	1.287055	3.385369	0.927306	C	0.302835	3.057973	1.711021
C	7.506847	-1.080387	0.816103	C	7.403228	-1.008939	1.080255
C	-2.921074	-1.818676	0.809651	C	-2.872567	-1.800338	0.788519
C	-3.878429	-1.233615	-0.256292	C	-3.873853	-1.218846	-0.237525
C	-4.868285	-0.184307	0.299801	C	-4.844665	-0.1742	0.359508
O	-3.98773	0.838512	0.81642	O	-3.947402	0.850936	0.840654
C	-5.695717	0.444261	-0.837705	C	-5.720907	0.450409	-0.74295
O	-6.238848	1.66363	-0.309724	O	-6.248026	1.667344	-0.193723
Cl	-4.771782	-2.576491	-1.130025	Cl	-4.795929	-2.564847	-1.076397
C	-5.775002	-0.707882	1.41896	C	-5.702853	-0.703165	1.513896
O	5.031764	-2.256013	1.646543	O	4.871867	-2.129242	1.833404
O	4.934734	1.925491	-1.739462	O	5.026243	1.732542	-1.914675
H	0.802629	-4.255227	-0.738006	H	0.804116	-4.241493	-0.861827
H	-1.518963	-4.087628	0.097472	H	-1.490538	-4.069256	0.043102
H	2.213824	-2.240531	-1.000646	H	2.204029	-2.221309	-1.182613
H	-1.89674	0.864208	0.627169	H	-1.855653	0.891206	0.543542
H	2.575038	1.762798	-1.400641	H	2.642768	1.654922	-1.660203
H	6.929144	0.703214	-1.082247	H	6.962088	0.552408	-1.046903
H	6.296132	-1.783329	-0.816231	H	6.305497	-1.882901	-0.549293
H	3.030964	-0.986666	1.025905	H	2.927975	-0.904433	0.988623
H	-1.099731	2.816222	2.099752	H	-1.501661	3.159267	-1.31868
H	-2.958258	4.219824	1.481628	H	-3.149032	4.307193	0.023867
H	-2.320114	4.3984	-0.243082	H	-2.098722	4.154052	1.536112
H	0.808119	3.159181	-1.810242	H	2.132891	3.377833	-0.286654
H	-0.957079	3.079578	-1.73993	H	1.029239	3.449345	-1.675827
H	-0.102771	4.52666	-1.155051	H	0.956231	4.69366	-0.417054
H	1.395904	2.948135	1.926217	H	-0.440322	2.544417	2.330276
H	2.203755	3.181919	0.369127	H	1.291967	2.681392	1.990904
H	1.18548	4.470937	1.036958	H	0.269211	4.12862	1.94501
H	8.388835	-0.899307	0.190288	H	8.326471	-0.8856	0.501679

H	7.48221	-0.32974	1.612921	H	7.324403	-0.182195	1.794057
H	7.595841	-2.070045	1.269531	H	7.460041	-1.947796	1.63554
H	-3.29841	-2.785772	1.153356	H	-3.233913	-2.76728	1.149371
H	-2.93818	-1.147013	1.67617	H	-2.854859	-1.12667	1.653466
H	-3.300137	-0.749155	-1.045307	H	-3.329819	-0.731516	-1.04889
H	-6.434278	0.094346	1.764504	H	-6.353756	0.094599	1.88484
H	-6.394505	-1.538505	1.065031	H	-6.329932	-1.538458	1.184966
H	-5.180084	-1.052508	2.269557	H	-5.072482	-1.042729	2.340646
H	-6.49594	-0.233229	-1.153629	H	-6.529705	-0.231664	-1.025222
H	-5.044146	0.658554	-1.695787	H	-5.106609	0.667626	-1.627431
H	-6.686658	2.152652	-1.015512	H	-6.746348	2.142375	-0.874762
H	-4.484868	1.676805	0.797752	H	-4.447698	1.68741	0.84675
Free Energy = -1857.58397 Hartree				Free Energy = -1857.583967 Hartree			

conf. 3b-3	X axis(Å)	Y axis(Å)	Z axis(Å)	conf. 3b-4	X axis(Å)	Y axis(Å)	Z axis(Å)
C	0.192491	-3.261724	-0.670773	C	-0.444383	-2.848172	-1.959067
C	-1.130292	-3.155581	-0.192425	C	0.957215	-2.736664	-1.838174
C	-1.661447	-1.935793	0.222916	C	1.565794	-1.555803	-1.41652
C	-0.80144	-0.825286	0.150861	C	0.703494	-0.490022	-1.098065
C	0.543907	-0.907166	-0.288574	C	-0.707746	-0.579776	-1.192575
C	1.031575	-2.154595	-0.72834	C	-1.28199	-1.782059	-1.651595
N	-1.061195	0.500155	0.447633	N	1.006162	0.797534	-0.704561
C	0.057174	1.27047	0.206897	C	-0.150531	1.538698	-0.54923
C	1.089996	0.436318	-0.232697	C	-1.245139	0.719892	-0.831937
C	2.430668	0.874339	-0.593471	C	-2.667039	1.065221	-0.807428
C	3.607012	0.260565	-0.322363	C	-3.651839	0.367477	-0.197432
C	4.880649	0.87788	-0.795225	C	-5.065676	0.828401	-0.330427
N	6.018565	0.179229	-0.488223	N	-5.997044	0.08746	0.342661
C	6.092981	-1.222666	-0.077194	C	-5.771688	-0.952231	1.344822
C	4.842067	-1.641983	0.69941	C	-4.328557	-1.45748	1.353459
N	3.720202	-0.892905	0.479868	N	-3.421858	-0.77564	0.591995
C	0.056923	2.780212	0.444371	C	-0.027751	2.966285	-0.01988
C	0.303218	3.466844	-0.896741	C	1.162723	3.6294	-0.704721
C	1.280769	4.323561	-1.204779	C	2.23253	4.179746	-0.123219
C	1.116176	3.142985	1.508907	C	0.129932	2.912338	1.518793
C	-1.322656	3.266261	0.955048	C	-1.26764	3.828998	-0.358776
C	7.362982	-1.476019	0.737059	C	-6.754187	-2.114789	1.153928
C	-3.07709	-1.777693	0.725664	C	3.063361	-1.386898	-1.316846
C	-4.007354	-1.059178	-0.28005	C	3.577868	-1.331508	0.141399
C	-4.978534	-0.04936	0.373143	C	4.670177	-0.264096	0.381519
O	-4.081772	0.92067	0.96488	O	3.987838	0.965335	0.047437
C	-5.811993	0.684518	-0.694459	C	5.053023	-0.194209	1.871902

O	-6.341712	1.852666	-0.05015	O	5.724009	1.06175	2.052216
Cl	-4.923701	-2.281789	-1.293364	Cl	4.150623	-2.986254	0.685058
C	-5.879494	-0.6566	1.45422	C	5.91325	-0.430919	-0.499804
O	4.83848	-2.622584	1.435599	O	-4.005735	-2.416628	2.045963
O	4.924522	1.960713	-1.379338	O	-5.38684	1.796183	-1.020148
H	0.559932	-4.230076	-0.999661	H	-0.872309	-3.784359	-2.307436
H	-1.756711	-4.043565	-0.153537	H	1.582889	-3.589345	-2.091327
H	2.040996	-2.253227	-1.115266	H	-2.357094	-1.872434	-1.778247
H	-1.967798	0.851252	0.741636	H	1.943689	1.138356	-0.50337
H	2.532668	1.820698	-1.116587	H	-3.013615	1.945592	-1.33802
H	6.865667	0.598813	-0.856937	H	-6.943852	0.439095	0.245198
H	6.115971	-1.872864	-0.969518	H	-5.929645	-0.529192	2.35023
H	2.888546	-1.217892	0.964865	H	-2.468008	-1.120938	0.658219
H	-0.431365	3.21236	-1.663177	H	1.084931	3.663648	-1.792921
H	1.345897	4.759515	-2.198308	H	3.006187	4.657381	-0.71935
H	2.0575	4.610883	-0.501831	H	2.377883	4.187994	0.953983
H	0.891102	2.628235	2.449571	H	-0.745935	2.429943	1.964627
H	2.124089	2.853054	1.20069	H	1.017184	2.343438	1.817562
H	1.110383	4.222107	1.702052	H	0.210629	3.922826	1.936604
H	-2.122441	3.069497	0.231645	H	-1.461256	3.853988	-1.43685
H	-1.587932	2.792848	1.908245	H	-2.165607	3.460904	0.144945
H	-1.283867	4.348089	1.116724	H	-1.090069	4.856773	-0.024443
H	8.250938	-1.246035	0.136207	H	-7.786644	-1.755021	1.236192
H	7.376204	-0.851343	1.636326	H	-6.62353	-2.572828	0.167731
H	7.406397	-2.524607	1.03959	H	-6.584579	-2.872505	1.922661
H	-3.499372	-2.752378	0.985427	H	3.573426	-2.189017	-1.857414
H	-3.064254	-1.18473	1.648369	H	3.34768	-0.446704	-1.804646
H	-3.41016	-0.509844	-1.010629	H	2.750349	-1.106976	0.817259
H	-6.53926	0.115314	1.862439	H	6.633635	0.362209	-0.276694
H	-6.499963	-1.458584	1.040816	H	6.395596	-1.397058	-0.317863
H	-5.280806	-1.063864	2.273645	H	5.64828	-0.365472	-1.558796
H	-6.619326	0.042774	-1.061935	H	5.709603	-1.028179	2.141112
H	-5.167617	0.972722	-1.53607	H	4.146115	-0.233699	2.490589
H	-6.800116	2.40481	-0.700328	H	5.924685	1.19203	2.990543
H	-4.60125	1.737524	1.081403	H	4.472588	1.686276	0.488784
Free Energy = -1857.583667 Hartree				Free Energy = -1857.583667 Hartree			

conf. 3b-5	X axis(Å)	Y axis(Å)	Z axis(Å)	conf. 3b-6	X axis(Å)	Y axis(Å)	Z axis(Å)
C	0.14917	-3.303795	-0.504358	C	0.149803	-3.303951	-0.503707
C	-1.170975	-3.157055	-0.029176	C	-1.170511	-3.157439	-0.028919
C	-1.692716	-1.908681	0.304987	C	-1.692556	-1.909152	0.305058
C	-0.825827	-0.811374	0.155065	C	-0.825819	-0.811685	0.155339

C	0.51667	-0.931173	-0.283861	C	0.516825	-0.931251	-0.283173
C	0.994746	-2.208473	-0.640394	C	0.995231	-2.208486	-0.639507
N	-1.075193	0.532407	0.364509	N	-1.075544	0.532077	0.364533
C	0.04743	1.277678	0.068796	C	0.047009	1.277539	0.069113
C	1.071775	0.409715	-0.318665	C	1.071657	0.409759	-0.317985
C	2.412798	0.806281	-0.727064	C	2.412594	0.806621	-0.726319
C	3.585657	0.201456	-0.426406	C	3.585571	0.201878	-0.42592
C	4.85512	0.759479	-0.976659	C	4.854895	0.760133	-0.976292
N	5.994774	0.090947	-0.614754	N	5.994567	0.091173	-0.615278
C	6.075557	-1.257744	-0.054348	C	6.075318	-1.257699	-0.055292
C	4.822302	-1.606783	0.752362	C	4.822632	-1.606283	0.752542
N	3.69986	-0.881225	0.468054	N	3.70002	-0.880901	0.468371
C	0.04155	2.801815	0.172977	C	0.040761	2.801677	0.173063
C	1.249834	3.250299	0.987845	C	1.249065	3.250637	0.987634
C	2.175257	4.147631	0.640902	C	2.174106	4.148265	0.640434
C	-1.214521	3.302058	0.933173	C	-1.2153	3.301691	0.933431
C	0.016418	3.40924	-1.249912	C	0.015176	3.408825	-1.249944
C	7.340102	-1.411286	0.793796	C	7.340628	-1.412061	0.791451
C	-3.104388	-1.706602	0.802426	C	-3.104366	-1.707263	0.802163
C	-4.030553	-1.03438	-0.238165	C	-4.030276	-1.034682	-0.238408
C	-4.999582	0.006943	0.366569	C	-4.999209	0.006678	0.366404
O	-4.100446	1.000315	0.914472	O	-4.09996	0.999753	0.914759
C	-5.829818	0.69488	-0.733723	C	-5.829081	0.695003	-0.733906
O	-6.358454	1.890886	-0.141775	O	-6.357343	1.891119	-0.141923
Cl	-4.948278	-2.301013	-1.194167	Cl	-4.948158	-2.300925	-1.194758
C	-5.903431	-0.54909	1.472504	C	-5.903341	-0.549493	1.472027
O	4.819343	-2.51864	1.572368	O	4.820117	-2.517656	1.573065
O	4.894482	1.772904	-1.67459	O	4.894139	1.774046	-1.673508
H	0.509043	-4.294464	-0.768817	H	0.509931	-4.294561	-0.768041
H	-1.802792	-4.036338	0.071835	H	-1.802197	-4.036835	0.07194
H	2.001636	-2.339567	-1.024322	H	2.002253	-2.339431	-1.023129
H	-1.972267	0.906818	0.657982	H	-1.972654	0.906269	0.658227
H	2.530525	1.705272	-1.322339	H	2.53015	1.705812	-1.321321
H	6.837557	0.46645	-1.037019	H	6.837393	0.467011	-1.037144
H	6.113717	-1.99938	-0.871474	H	6.112225	-1.999141	-0.872671
H	2.87047	-1.158507	0.985488	H	2.87092	-1.157863	0.986443
H	1.313174	2.785553	1.973199	H	1.312776	2.786005	1.973018
H	2.980923	4.406581	1.322669	H	2.979809	4.407549	1.322032
H	2.191032	4.637407	-0.328822	H	2.189576	4.637919	-0.329354
H	-2.138953	3.048641	0.400232	H	-2.139771	3.047828	0.400769
H	-1.270174	2.887261	1.946337	H	-1.270573	2.887114	1.946709
H	-1.170201	4.392065	1.021133	H	-1.171319	4.391732	1.02112
H	0.900787	3.135152	-1.832116	H	0.899576	3.134954	-1.832206

H	-0.866128	3.053542	-1.792964	H	-0.867317	3.052679	-1.792789
H	-0.034107	4.503278	-1.201149	H	-0.035766	4.502854	-1.201399
H	8.231744	-1.237037	0.179772	H	8.231746	-1.238194	0.176558
H	7.339145	-0.694659	1.621742	H	7.340931	-0.69564	1.619582
H	7.389879	-2.421785	1.205431	H	7.390279	-2.422668	1.202833
H	-3.536603	-2.659995	1.118882	H	-3.536634	-2.660781	1.118185
H	-3.080767	-1.061473	1.68927	H	-3.080968	-1.062458	1.689246
H	-3.430993	-0.52114	-0.992634	H	-3.430536	-0.521395	-0.992704
H	-6.561008	0.241887	1.846228	H	-6.560741	0.241541	1.845932
H	-6.526191	-1.366007	1.093055	H	-6.526261	-1.366114	1.09221
H	-5.307006	-0.923044	2.309286	H	-5.307114	-0.923901	2.308749
H	-6.638037	0.039717	-1.074853	H	-6.637465	0.040129	-1.075214
H	-5.182904	0.944695	-1.585617	H	-5.181982	0.94469	-1.585706
H	-6.820171	2.412047	-0.814765	H	-6.820455	2.411609	-0.814471
H	-4.621556	1.819395	1.004689	H	-4.62091	1.818898	1.005238
Free Energy = -1857.583526 Hartree				Free Energy = -1857.58352 Hartree			

conf. 3b-7	X axis(Å)	Y axis(Å)	Z axis(Å)	conf. 3b-8	X axis(Å)	Y axis(Å)	Z axis(Å)
C	-0.23019	-3.040656	-1.528502	C	-0.229871	-3.040526	-1.528819
C	1.167551	-2.8531	-1.497585	C	1.167835	-2.852819	-1.497906
C	1.73133	-1.609555	-1.217531	C	1.731488	-1.609198	-1.217864
C	0.830292	-0.563632	-0.949338	C	0.830342	-0.56338	-0.949676
C	-0.577715	-0.72764	-0.942449	C	-0.577673	-0.72756	-0.942738
C	-1.106756	-1.994083	-1.264353	C	-1.106557	-1.994051	-1.264657
N	1.09415	0.77092	-0.701141	N	1.094048	0.771201	-0.701502
C	-0.085667	1.466925	-0.536529	C	-0.085855	1.467048	-0.536733
C	-1.154251	0.575616	-0.664397	C	-1.154331	0.575623	-0.664636
C	-2.566121	0.926844	-0.592588	C	-2.566231	0.926721	-0.592731
C	-3.567867	0.228456	-0.009147	C	-3.567853	0.228308	-0.009121
C	-4.961031	0.758594	-0.07872	C	-4.961072	0.75834	-0.078521
N	-5.91345	0.001329	0.54921	N	-5.9133	0.001183	0.549859
C	-5.77561	-1.375518	1.020024	C	-5.775358	-1.375758	1.020378
C	-4.321616	-1.736854	1.313312	C	-4.321309	-1.73708	1.313423
N	-3.364852	-0.941152	0.750555	N	-3.36467	-0.941223	0.75069
C	-0.091293	2.974926	-0.293219	C	-0.091635	2.975045	-0.29335
C	-0.988833	3.287266	0.899194	C	-0.9891	3.287224	0.899162
C	-1.99894	4.158601	0.948145	C	-1.999286	4.158454	0.948254
C	-0.537045	3.694515	-1.588731	C	-0.537619	3.694655	-1.588773
C	1.327157	3.483956	0.073812	C	1.326791	3.484235	0.073566
C	-6.387689	-2.396563	0.041077	C	-6.3875	-2.396678	0.041326
C	3.219783	-1.35386	-1.2202	C	3.21992	-1.353381	-1.220432
C	3.80498	-1.130555	0.194023	C	3.805011	-1.130466	0.193936

C	4.863924	-0.006829	0.266207	C	4.86397	-0.006739	0.266505
O	4.113406	1.166996	-0.125986	O	4.113475	1.167157	-0.125387
C	5.33824	0.212211	1.715492	C	5.338198	0.211931	1.715881
O	5.960996	1.505381	1.737582	O	5.961194	1.505	1.738323
Cl	4.473035	-2.699809	0.866486	Cl	4.472882	-2.699895	0.866036
C	6.05382	-0.207068	-0.678576	C	6.053952	-0.206731	-0.678231
O	-4.042811	-2.717949	1.995057	O	-4.042361	-2.718299	1.994946
O	-5.243999	1.822203	-0.630986	O	-5.244232	1.821775	-0.631028
H	-0.626691	-4.023098	-1.770412	H	-0.626268	-4.023009	-1.770737
H	1.823708	-3.69361	-1.710996	H	1.824089	-3.693249	-1.711321
H	-2.179167	-2.152552	-1.320816	H	-2.178951	-2.152641	-1.321153
H	2.026311	1.148769	-0.559112	H	2.026149	1.149071	-0.559154
H	-2.88981	1.868042	-1.023406	H	-2.890061	1.867839	-1.023625
H	-6.853891	0.3698	0.453435	H	-6.853803	0.369472	0.453942
H	-6.294794	-1.459873	1.981629	H	-6.294432	-1.460311	1.982025
H	-2.407524	-1.222281	0.943079	H	-2.407297	-1.22243	0.942866
H	-0.725306	2.73862	1.805143	H	-0.725426	2.73855	1.805052
H	-2.553869	4.315191	1.869318	H	-2.554165	4.314937	1.869475
H	-2.333349	4.725584	0.083707	H	-2.33381	4.725478	0.083883
H	0.138836	3.434942	-2.410734	H	0.138247	3.43527	-2.410845
H	-1.551362	3.416203	-1.888371	H	-1.551909	3.416181	-1.888347
H	-0.506627	4.782325	-1.455896	H	-0.507384	4.782457	-1.455835
H	1.714202	2.99745	0.976635	H	1.713982	2.997736	0.976327
H	2.04023	3.322649	-0.743855	H	2.039792	3.323066	-0.744184
H	1.282306	4.560219	0.267241	H	1.281815	4.560482	0.267056
H	-7.451719	-2.181271	-0.110768	H	-7.451554	-2.1814	-0.110363
H	-5.886991	-2.35257	-0.932585	H	-5.886911	-2.352511	-0.932382
H	-6.290479	-3.407984	0.447302	H	-6.290203	-3.408155	0.447389
H	3.746911	-2.17647	-1.711218	H	3.747174	-2.175791	-1.71164
H	3.42419	-0.452015	-1.810461	H	3.424282	-0.451321	-1.810372
H	3.004721	-0.868058	0.888961	H	3.004684	-0.868087	0.88884
H	6.760026	0.62138	-0.565381	H	6.760202	0.621635	-0.564716
H	6.578492	-1.141679	-0.453868	H	6.578564	-1.141432	-0.45374
H	5.721388	-0.233589	-1.720039	H	5.721635	-0.232943	-1.719736
H	6.048352	-0.567051	2.010587	H	6.048152	-0.567506	2.010893
H	4.476529	0.192738	2.396508	H	4.476411	0.192464	2.396795
H	6.196383	1.739077	2.647395	H	6.196113	1.738639	2.648274
H	4.597012	1.933172	0.234463	H	4.597186	1.933242	0.235108
Free Energy = -1857.583436 Hartree				Free Energy = -1857.583436 Hartree			

Table S22. The Cartesian coordinates of the lowest energy conformers (Conf. **1-8**) for isomer **3c**.

conf. 3c-1	X axis(Å)	Y axis(Å)	Z axis(Å)	conf. 3c-2	X axis(Å)	Y axis(Å)	Z axis(Å)
C	0.418002	-2.863617	-1.943344	C	0.14415	-2.873839	-1.89545
C	-0.982767	-2.744858	-1.820351	C	-1.249871	-2.679356	-1.80182
C	-1.58428	-1.559704	-1.400925	C	-1.790773	-1.472181	-1.362767
C	-0.715572	-0.497466	-1.087868	C	-0.870246	-0.472751	-1.000166
C	0.694819	-0.594121	-1.184644	C	0.535629	-0.647394	-1.056646
C	1.2614	-1.800958	-1.640346	C	1.040182	-1.872068	-1.539277
N	-1.011996	0.792861	-0.697759	N	-1.111632	0.825123	-0.588422
C	0.147243	1.530138	-0.548001	C	0.079445	1.488079	-0.379775
C	1.238768	0.70553	-0.831136	C	1.134624	0.610414	-0.648454
C	2.658976	1.051778	-0.816652	C	2.549097	0.946283	-0.571935
C	3.657004	0.341837	-0.242117	C	3.559963	0.181185	-0.095923
C	5.062444	0.834648	-0.350767	C	4.953162	0.715076	-0.112133
N	6.005987	0.060857	0.268446	N	5.911022	-0.13521	0.374542
C	5.845792	-1.311271	0.749021	C	5.773594	-1.578399	0.569577
C	4.387864	-1.626832	1.092042	C	4.330637	-1.958621	0.912237
N	3.442017	-0.806885	0.543154	N	3.367283	-1.071551	0.521105
C	0.027481	2.958968	-0.020224	C	0.114175	2.94801	0.070892
C	-1.158075	3.624754	-0.71128	C	0.743478	3.766387	-1.053898
C	-2.228573	4.179536	-0.135308	C	1.824444	4.547863	-0.983065
C	1.270289	3.8205	-0.351157	C	0.881735	3.071134	1.405884
C	-0.13858	2.905469	1.517729	C	-1.316878	3.500634	0.28736
C	6.764006	-1.57206	1.945849	C	6.746788	-2.069082	1.643109
C	-3.080733	-1.382987	-1.298032	C	-3.27571	-1.204141	-1.296871
C	-3.59125	-1.319921	0.161296	C	-3.826368	-1.143325	0.147273
C	-4.678471	-0.247	0.399922	C	-4.86363	-0.020537	0.377642
C	-5.924627	-0.412516	-0.477271	C	-6.078866	-0.089814	-0.553947
C	-5.056854	-0.16893	1.891017	C	-5.300437	0.032544	1.854206
O	-5.723099	1.090092	2.067408	O	-5.897966	1.323631	2.044603
H	-2.76103	-1.096215	0.834116	H	-3.007449	-0.978111	0.850347
O	-3.991848	0.978055	0.058604	O	-4.102015	1.180522	0.108164
O	4.0893	-2.594529	1.782888	O	4.056265	-3.020165	1.461353
O	5.357867	1.886965	-0.916881	O	5.230354	1.853937	-0.488603
H	0.840753	-3.803483	-2.288121	H	0.522002	-3.824981	-2.260793
H	-1.613211	-3.595255	-2.069339	H	-1.921584	-3.48351	-2.09273
H	2.335812	-1.898735	-1.764338	H	2.108496	-2.031797	-1.646781
H	-1.947816	1.138324	-0.496345	H	-2.035268	1.193967	-0.381605
H	2.992512	1.958349	-1.309311	H	2.855752	1.935553	-0.89955
H	6.954119	0.403392	0.152947	H	6.853559	0.235518	0.313269
H	6.113826	-2.018879	-0.054504	H	6.000555	-2.106252	-0.373459
H	2.48061	-1.077698	0.731423	H	2.413583	-1.365521	0.712126

H	-1.075308	3.657261	-1.799162	H	0.215402	3.685293	-2.005703
H	-2.378596	4.190073	0.94124	H	2.411109	4.663521	-0.07599
H	-2.997955	4.658906	-0.735561	H	2.173824	5.094353	-1.855215
H	2.16499	3.453715	0.159248	H	0.388519	2.469038	2.177196
H	1.09009	4.848335	-0.018516	H	0.893809	4.113558	1.745055
H	1.471124	3.845841	-1.427886	H	1.914904	2.724637	1.320817
H	-0.217946	3.916316	1.934845	H	-1.253022	4.546393	0.604301
H	0.733484	2.420596	1.968303	H	-1.848241	2.943684	1.068737
H	-1.029397	2.339819	1.81196	H	-1.913959	3.471262	-0.631597
H	6.63869	-2.600646	2.291344	H	6.634049	-3.146634	1.781337
H	6.525769	-0.892289	2.770641	H	6.550899	-1.570984	2.598389
H	7.81181	-1.422276	1.659571	H	7.780196	-1.858997	1.342553
H	-3.596095	-2.184542	-1.834401	H	-3.824437	-1.957642	-1.868644
H	-3.361906	-0.44317	-1.788378	H	-3.482402	-0.236848	-1.77129
Cl	-4.169413	-2.970153	0.712877	Cl	-4.508514	-2.771189	0.642938
H	-6.640979	0.384756	-0.255987	H	-6.762878	0.73504	-0.331573
H	-5.662197	-0.353278	-1.537262	H	-5.770825	-0.007426	-1.599967
H	-6.41065	-1.375681	-0.289448	H	-6.619408	-1.03273	-0.420406
H	-4.148258	-0.208561	2.507222	H	-4.423739	-0.081814	2.506107
H	-5.715417	-0.999445	2.166016	H	-6.018503	-0.764486	2.072826
H	-5.919491	1.225975	3.005852	H	-6.110714	1.450485	2.980752
H	-4.472957	1.703102	0.49718	H	-4.569251	1.907101	0.560513
Free Energy = -1857.584631 Hartree				Free Energy = -1857.584271 Hartree			

**conf. 3c-3**	**X axis(Å)**	**Y axis(Å)**	**Z axis(Å)**	**conf. 3c-4**	**X axis(Å)**	**Y axis(Å)**	**Z axis(Å)**
C	0.379528	-2.889734	-1.846546	C	-0.273647	-3.255899	-0.530613
C	-1.021356	-2.752585	-1.755368	C	1.062569	-3.151307	-0.090471
C	-1.615266	-1.559943	-1.345725	C	1.618173	-1.926604	0.275606
C	-0.739917	-0.510487	-1.010516	C	0.768751	-0.808454	0.194192
C	0.671962	-0.62773	-1.067713	C	-0.588549	-0.887576	-0.207768
C	1.230603	-1.840759	-1.518035	C	-1.101757	-2.141167	-0.598273
N	-1.030809	0.785796	-0.627605	N	1.049628	0.521691	0.446782
C	0.132681	1.501805	-0.438527	C	-0.067114	1.297176	0.213612
C	1.221231	0.663138	-0.69478	C	-1.119103	0.462482	-0.175603
C	2.629619	1.039606	-0.677006	C	-2.466473	0.901599	-0.512567
C	3.667219	0.332473	-0.172205	C	-3.637572	0.31332	-0.172588
C	5.048053	0.889696	-0.272129	C	-4.921124	0.921219	-0.632081
N	6.035831	0.109463	0.264903	N	-6.058315	0.267899	-0.239999
C	5.938471	-1.30406	0.62856	C	-6.15489	-1.073333	0.332565
C	4.505622	-1.693841	1.001094	C	-4.856795	-1.500178	1.01357
N	3.514825	-0.871801	0.541374	N	-3.72929	-0.805167	0.680119
C	0.078603	2.98271	-0.060799	C	-0.04655	2.812722	0.408127

C	-1.289331	3.315706	0.521133	C	-0.3216	3.463444	-0.945217
C	-2.186223	4.184171	0.045378	C	-1.301421	4.317495	-1.253427
C	0.396233	3.830005	-1.315065	C	1.350245	3.29963	0.868695
C	1.107009	3.307742	1.057257	C	-1.074614	3.215622	1.48857
C	6.913552	-1.63503	1.760906	C	-6.579534	-2.128448	-0.707154
C	-3.111407	-1.364319	-1.281551	C	3.047355	-1.772635	0.740117
C	-3.662204	-1.315734	0.163054	C	3.960702	-1.082779	-0.300786
C	-4.74936	-0.238986	0.38181	C	4.95112	-0.065315	0.310217
C	-5.968771	-0.384451	-0.535808	C	5.872721	-0.655445	1.383446
C	-5.173984	-0.177167	1.861229	C	5.764531	0.64332	-0.789746
O	-5.832238	1.086647	2.034095	O	6.312077	1.822031	-0.180435
H	-2.850404	-1.104335	0.861927	H	3.351818	-0.546139	-1.031129
O	-4.043339	0.984441	0.077763	O	4.072904	0.921618	0.901876
O	4.263329	-2.714335	1.6357	O	-4.839065	-2.445357	1.7953
O	5.288137	1.994235	-0.76051	O	-4.969378	1.962304	-1.287955
H	0.797738	-3.832942	-2.187748	H	-0.659948	-4.22904	-0.821589
H	-1.657934	-3.592246	-2.024125	H	1.679815	-4.045251	-0.042085
H	2.305231	-1.954722	-1.62207	H	-2.122199	-2.238447	-0.955844
H	-1.966606	1.139264	-0.441933	H	1.966587	0.870093	0.710831
H	2.921459	1.996701	-1.09952	H	-2.577561	1.822854	-1.077013
H	6.967301	0.496349	0.15413	H	-6.911525	0.688245	-0.593096
H	6.193712	-1.930554	-0.243791	H	-6.905827	-1.048909	1.130737
H	2.570336	-1.17999	0.755625	H	-2.877733	-1.130422	1.12946
H	-1.51683	2.795558	1.454025	H	0.392227	3.182625	-1.721992
H	-2.036442	4.743848	-0.873716	H	-2.058745	4.629723	-0.540038
H	-3.115697	4.373464	0.577224	H	-1.388468	4.725699	-2.257015
H	1.39391	3.593853	-1.696209	H	1.636452	2.849971	1.827276
H	0.374018	4.899152	-1.0739	H	1.325185	4.385812	1.001353
H	-0.322117	3.638433	-2.11941	H	2.128662	3.075341	0.130165
H	1.018598	4.363027	1.339069	H	-1.056577	4.300024	1.648536
H	2.134041	3.125832	0.732569	H	-0.828444	2.727802	2.438246
H	0.922738	2.697938	1.94912	H	-2.092067	2.923708	1.215574
H	6.833746	-2.692807	2.021166	H	-6.671146	-3.108061	-0.22827
H	6.689947	-1.036581	2.650253	H	-5.846365	-2.194201	-1.518808
H	7.943395	-1.426649	1.447388	H	-7.549143	-1.859404	-1.141908
H	-3.621824	-2.151209	-1.843773	H	3.466177	-2.746381	1.008807
H	-3.366921	-0.413957	-1.76545	H	3.062428	-1.161848	1.651068
Cl	-4.265645	-2.96858	0.678399	Cl	4.84937	-2.332796	-1.305229
H	-6.687141	0.413987	-0.325295	H	6.542593	0.121909	1.76389
H	-5.673486	-0.311556	-1.586261	H	5.290136	-1.04621	2.222276
H	-6.465951	-1.347151	-0.376925	H	6.482526	-1.46652	0.971972
H	-4.286315	-0.23549	2.505789	H	5.103197	0.917095	-1.622968
H	-5.849796	-1.004084	2.103239	H	6.561815	-0.008526	-1.161391

H	-6.060425	1.210224	2.967041	H	6.758729	2.359155	-0.85106
H	-4.533746	1.707036	0.509806	H	4.599102	1.737786	0.98978
Free Energy = -1857.584111 Hartree				Free Energy = -1857.583989 Hartree			

conf. 3c-5	X axis(Å)	Y axis(Å)	Z axis(Å)	conf. 3c-6	X axis(Å)	Y axis(Å)	Z axis(Å)
C	-0.417975	-3.261391	-0.625774	C	0.125147	-2.997244	-1.730132
C	0.8911	-3.166555	-0.107883	C	-1.268408	-2.797488	-1.640964
C	1.46109	-1.940236	0.230506	C	-1.807613	-1.560877	-1.290614
C	0.652333	-0.803609	0.040712	C	-0.885551	-0.535923	-1.0129
C	-0.676639	-0.873548	-0.446552	C	0.519936	-0.71384	-1.063409
C	-1.205862	-2.129878	-0.803059	C	1.022517	-1.971123	-1.45575
N	0.940225	0.529804	0.253876	N	-1.125643	0.790044	-0.702383
C	-0.14509	1.317047	-0.087088	C	0.066618	1.467521	-0.552854
C	-1.182734	0.485167	-0.510317	C	1.120319	0.571677	-0.754204
C	-2.529084	0.861422	-0.945793	C	2.536847	0.907005	-0.716283
C	-3.692342	0.333699	-0.501908	C	3.552613	0.174717	-0.202252
C	-4.980125	0.792677	-1.103131	C	4.94653	0.698136	-0.292196
N	-6.112342	0.24255	-0.568741	N	5.91075	-0.105414	0.257505
C	-6.238039	-0.528728	0.666621	C	5.776131	-1.522653	0.593766
C	-4.898419	-1.09356	1.14129	C	4.335117	-1.87323	0.973452
N	-3.771501	-0.625092	0.526143	N	3.367443	-1.025223	0.513247
C	-0.064226	2.818853	0.180605	C	0.097839	2.964589	-0.24978
C	1.303527	3.320305	-0.269279	C	1.02923	3.216903	0.93084
C	2.220706	3.952702	0.468646	C	2.053519	4.070966	0.988878
C	-0.317429	3.06387	1.687931	C	-1.304094	3.47567	0.173054
C	-1.112007	3.619094	-0.630442	C	0.520373	3.730897	-1.526286
C	-7.275853	-1.645647	0.505438	C	6.751421	-1.904198	1.709279
C	2.861705	-1.807802	0.78048	C	-3.292299	-1.290136	-1.229456
C	3.86811	-1.226731	-0.240892	C	-3.824921	-1.122847	0.21307
C	4.838719	-0.184778	0.361285	C	-4.860565	0.013123	0.374412
C	5.690634	-0.71675	1.518927	C	-6.089903	-0.126426	-0.530363
C	5.721299	0.438683	-0.736771	C	-5.273944	0.17589	1.849628
O	6.2489	1.653886	-0.184109	O	-5.873583	1.475945	1.952287
H	3.328172	-0.73718	-1.053659	H	-2.996716	-0.905819	0.890779
O	3.941842	0.842135	0.839299	O	-4.106161	1.191471	0.003728
O	-4.848553	-1.902144	2.061573	O	4.065904	-2.884356	1.61309
O	-5.029462	1.591446	-2.038437	O	5.221367	1.792222	-0.785301
H	-0.813708	-4.238729	-0.888608	H	0.501659	-3.972668	-2.026278
H	1.476839	-4.072756	0.027684	H	-1.941148	-3.622046	-1.86469
H	-2.206793	-2.214461	-1.216852	H	2.090185	-2.137935	-1.558314
H	1.851656	0.887452	0.532576	H	-2.047871	1.1697	-0.508946
H	-2.652137	1.616722	-1.714636	H	2.853308	1.866107	-1.111455

H	-6.969271	0.592798	-0.984332	H	6.852625	0.25418	0.142678
H	-6.570229	0.136322	1.480969	H	6.004017	-2.139851	-0.293108
H	-2.900232	-0.98896	0.902924	H	2.416163	-1.301625	0.73926
H	1.508881	3.155401	-1.328838	H	0.780547	2.635543	1.820453
H	2.087462	4.155136	1.528034	H	2.375102	4.667816	0.13982
H	3.14809	4.304525	0.022684	H	2.633716	4.181883	1.90108
H	-1.308123	2.687248	1.962105	H	-2.039611	3.352923	-0.631278
H	-0.286102	4.13547	1.917977	H	-1.241283	4.543374	0.405154
H	0.421414	2.553177	2.314642	H	-1.673436	2.960265	1.067182
H	-0.959334	4.689085	-0.452933	H	0.508181	4.812507	-1.348142
H	-2.132293	3.36864	-0.327584	H	-0.179869	3.514031	-2.340261
H	-1.016869	3.438339	-1.706866	H	1.52273	3.452718	-1.863809
H	-7.370398	-2.200269	1.441935	H	6.642455	-2.96381	1.950831
H	-6.977422	-2.338006	-0.288848	H	6.554318	-1.316563	2.611985
H	-8.252952	-1.219953	0.24797	H	7.784018	-1.720634	1.389254
H	3.219837	-2.775935	1.141297	H	-3.846124	-2.084943	-1.736625
H	2.841803	-1.135293	1.646287	H	-3.50626	-0.361161	-1.772366
Cl	4.790741	-2.573336	-1.078014	Cl	-4.497562	-2.71023	0.837018
H	6.342285	0.078939	1.893025	H	-6.774713	0.709217	-0.355649
H	5.055892	-1.055005	2.342855	H	-5.798446	-0.11581	-1.58435
H	6.316736	-1.55361	1.192091	H	-6.623903	-1.059804	-0.323387
H	5.111468	0.658171	-1.623765	H	-4.38634	0.113543	2.493839
H	6.5297	-0.245091	-1.016003	H	-5.984672	-0.604946	2.139147
H	6.751437	2.128264	-0.862498	H	-6.066115	1.67457	2.880324
H	4.444693	1.677028	0.849724	H	-4.56318	1.948394	0.414938
Free Energy = -1857.583674 Hartree				Free Energy = -1857.58364 Hartree			

**conf. 3c-7**	**X axis(Å)**	**Y axis(Å)**	**Z axis(Å)**	**conf. 3c-8**	**X axis(Å)**	**Y axis(Å)**	**Z axis(Å)**
C	-0.496913	-3.242984	-0.292046	C	-0.177639	-3.293502	-0.521424
C	0.843269	-3.157356	0.13943	C	1.142758	-3.154257	-0.044234
C	1.447374	-1.93135	0.413055	C	1.671954	-1.90911	0.290605
C	0.641687	-0.789384	0.249527	C	0.812144	-0.806267	0.140033
C	-0.718445	-0.847068	-0.146712	C	-0.530712	-0.919123	-0.299968
C	-1.280892	-2.104926	-0.444041	C	-1.016657	-2.193184	-0.65819
N	0.968192	0.544134	0.412043	N	1.067582	0.536059	0.349103
C	-0.121219	1.341	0.129782	C	-0.052912	1.286318	0.05363
C	-1.200995	0.520514	-0.210195	C	-1.079919	0.423278	-0.33327
C	-2.526387	0.962758	-0.626957	C	-2.425066	0.8167	-0.737951
C	-3.724255	0.441933	-0.272922	C	-3.59301	0.231088	-0.388063
C	-4.971494	1.031828	-0.843423	C	-4.868525	0.749888	-0.961167
N	-6.140597	0.452744	-0.433358	N	-6.009075	0.124778	-0.539166
C	-6.300245	-0.811659	0.28247	C	-6.162923	-0.884138	0.506155

C	-5.037196	-1.205511	1.044869	C	-4.82437	-1.403588	1.029194
N	-3.877523	-0.579627	0.683872	N	-3.688464	-0.829815	0.533939
C	-0.006476	2.864202	0.202956	C	-0.041476	2.81031	0.154455
C	1.21468	3.248796	1.028288	C	-1.250099	3.264844	0.965188
C	2.255063	3.994116	0.644281	C	-2.168177	4.168977	0.616019
C	-1.227582	3.472244	0.94861	C	-0.012636	3.413321	-1.270074
C	0.056537	3.43768	-1.231748	C	1.214685	3.307906	0.916139
C	-6.737248	-1.960408	-0.647149	C	-7.043071	-2.046649	0.024308
C	2.88745	-1.801608	0.850146	C	3.084068	-1.715943	0.790294
C	3.81575	-1.275921	-0.270779	C	4.015434	-1.046522	-0.247438
C	4.849869	-0.231349	0.208945	C	4.988113	-0.010639	0.360739
C	5.786354	-0.735396	1.312171	C	5.886668	-0.572406	1.468125
C	5.646447	0.334742	-0.98183	C	5.82433	0.674532	-0.736746
O	6.238323	1.558652	-0.521313	O	6.35758	1.867099	-0.141963
H	3.218418	-0.804405	-1.05337	H	3.419726	-0.529542	-1.002398
O	4.011886	0.82981	0.719338	O	4.092701	0.986581	0.907834
O	-5.073877	-2.06817	1.915629	O	-4.789852	-2.288089	1.87877
O	-4.961494	1.999404	-1.605453	O	-4.905239	1.667692	-1.781615
H	-0.921501	-4.219436	-0.509312	H	-0.54207	-4.281883	-0.788203
H	1.425861	-4.068421	0.253673	H	1.769276	-4.037271	0.057197
H	-2.305079	-2.189296	-0.794063	H	-2.022809	-2.316954	-1.047744
H	1.903623	0.888845	0.618204	H	1.965814	0.906437	0.644034
H	-2.606235	1.813836	-1.296988	H	-2.546784	1.679897	-1.383749
H	-6.96816	0.857863	-0.858089	H	-6.856793	0.507366	-0.944459
H	-7.069986	-0.672826	1.050632	H	-6.64835	-0.425171	1.381602
H	-3.050379	-0.884152	1.189953	H	-2.827312	-1.176316	0.947882
H	1.184363	2.896127	2.061192	H	-1.319964	2.799599	1.949852
H	2.357937	4.389086	-0.36281	H	-2.176599	4.66053	-0.352906
H	3.046081	4.247995	1.346095	H	-2.97362	4.433761	1.295952
H	-2.165678	3.268564	0.42719	H	0.868749	3.052356	-1.811488
H	-1.105111	4.558609	1.023461	H	0.04206	4.507363	-1.22528
H	-1.310007	3.065556	1.962826	H	-0.898057	3.140132	-1.851267
H	0.112621	4.532204	-1.208137	H	1.174619	4.398303	1.001401
H	-0.838667	3.162261	-1.796736	H	2.139129	3.049414	0.385656
H	0.926296	3.056082	-1.777926	H	1.266699	2.895137	1.930332
H	-6.87758	-2.877018	-0.066394	H	-7.16787	-2.775213	0.829353
H	-5.986194	-2.140847	-1.424308	H	-6.588566	-2.543189	-0.839754
H	-7.684449	-1.708009	-1.137644	H	-8.031665	-1.674942	-0.269981
H	3.260514	-2.761198	1.218729	H	3.510558	-2.672368	1.105354
H	2.946174	-1.096949	1.688174	H	3.062514	-1.072574	1.678479
Cl	4.642403	-2.66951	-1.130136	Cl	4.92939	-2.316009	-1.203311
H	6.474965	0.063347	1.604349	H	6.546987	0.21502	1.84451
H	5.216157	-1.03655	2.195535	H	5.286493	-0.944698	2.302967

H	6.374511	-1.591669	0.965942	H	6.506567	-1.391712	1.089139
H	4.966804	0.533571	-1.821699	H	5.180694	0.92875	-1.589816
H	6.417177	-0.374044	-1.302374	H	6.630089	0.015834	-1.076868
H	6.669736	2.010034	-1.26155	H	6.823527	2.386767	-0.813183
H	4.526933	1.654815	0.653882	H	4.617741	1.802906	1.000183
Free Energy = -1857.583553 Hartree				Free Energy = -1857.583352 Hartree			

Table S23. The Cartesian coordinates of the lowest energy conformers (Conf. **1-8**) for isomer **3d**.

conf. 3d-1	X axis(Å)	Y axis(Å)	Z axis(Å)	conf. 3d-2	X axis(Å)	Y axis(Å)	Z axis(Å)
C	0.063467	-2.695183	1.951554	C	0.063246	-2.695327	1.951411
C	1.442754	-2.452991	1.799537	C	1.442552	-2.453253	1.799419
C	1.928408	-1.252771	1.281576	C	1.928293	-1.253122	1.281322
C	0.964861	-0.299689	0.898432	C	0.964812	-0.300014	0.898072
C	-0.431063	-0.529042	1.01916	C	-0.431107	-0.52923	1.018838
C	-0.876699	-1.743028	1.577537	C	-0.876847	-1.743143	1.577269
N	1.127847	0.989562	0.414381	N	1.127949	0.989156	0.413833
C	-0.104554	1.587581	0.225743	C	-0.104387	1.587316	0.225228
C	-1.103029	0.677437	0.577832	C	-1.102949	0.67731	0.577432
C	-2.535414	0.94091	0.550387	C	-2.535309	0.940967	0.550384
C	-3.524887	0.111241	0.143483	C	-3.524897	0.111353	0.14364
C	-4.941681	0.577662	0.203272	C	-4.941664	0.577839	0.20374
N	-5.875769	-0.334684	-0.210749	N	-5.875819	-0.334342	-0.210496
C	-5.675644	-1.775016	-0.368572	C	-5.675702	-1.774694	-0.368174
C	-4.23052	-2.097875	-0.757233	C	-4.230783	-2.097353	-0.757841
N	-3.296712	-1.151296	-0.43971	N	-3.296818	-1.151095	-0.439752
C	-0.242909	3.0213	-0.286588	C	-0.242666	3.021053	-0.287145
C	-0.871483	3.852641	0.829553	C	-0.87015	3.852578	0.829457
C	-2.001455	4.562882	0.774927	C	-2.000183	4.562821	0.775937
C	-1.074791	3.037437	-1.588619	C	-1.075449	3.037466	-1.588592
C	1.136806	3.651278	-0.594697	C	1.136987	3.650641	-0.596307
C	-6.664606	-2.344898	-1.387401	C	-6.66541	-2.344862	-1.386056
C	3.410424	-0.974859	1.178605	C	3.410323	-0.975271	1.178497
C	3.948509	-0.948254	-0.261267	C	3.948561	-0.94845	-0.261288
C	5.303669	-0.226488	-0.4695	C	5.303605	-0.226388	-0.469231
O	5.589697	-0.172885	-1.877071	O	5.589717	-0.172285	-1.876739
C	5.166466	1.260247	-0.086136	C	5.166129	1.260179	-0.085363
O	4.014047	1.877182	-0.660394	O	4.013374	1.877125	-0.658992
H	3.223172	-0.479122	-0.925058	H	3.223257	-0.479384	-0.925158
C	6.472021	-0.850686	0.299388	C	6.472077	-0.850581	0.299523
O	-3.926371	-3.164974	-1.278693	O	-3.926869	-3.163958	-1.280419
O	-5.258234	1.714496	0.553331	O	-5.258122	1.714613	0.554052

H	-0.266026	-3.639911	2.375335	H	-0.266316	-3.639987	2.375293
H	2.153668	-3.217253	2.104084	H	2.153384	-3.217546	2.10408
H	-1.935347	-1.930341	1.727283	H	-1.935507	-1.930374	1.727001
H	2.014611	1.40479	0.142953	H	2.014833	1.404365	0.142672
H	-2.877448	1.924525	0.859085	H	-2.877154	1.924583	0.85925
H	-6.832124	-0.007169	-0.123009	H	-6.832163	-0.006823	-0.122683
H	-5.840378	-2.283007	0.597939	H	-5.839572	-2.282492	0.598602
H	-2.338525	-1.406716	-0.661286	H	-2.338728	-1.406257	-0.662049
H	-0.295894	3.851855	1.75696	H	-0.293693	3.851841	1.756331
H	-2.634832	4.59765	-0.107163	H	-2.634426	4.597618	-0.105534
H	-2.343994	5.130675	1.636119	H	-2.341852	5.1306	1.637482
H	-0.581374	2.428521	-2.354429	H	-0.582806	2.428338	-2.354732
H	-1.161273	4.060456	-1.972495	H	-1.161796	4.06053	-1.972376
H	-2.081873	2.639019	-1.441068	H	-2.082577	2.639434	-1.440364
H	0.990292	4.680009	-0.938807	H	0.990455	4.679313	-0.940601
H	1.661519	3.104826	-1.386584	H	1.660949	3.103809	-1.388434
H	1.787812	3.688715	0.28554	H	1.788613	3.688146	0.28345
H	-6.506396	-3.420075	-1.496505	H	-6.5071	-3.42002	-1.495188
H	-6.528863	-1.869737	-2.36453	H	-6.530589	-1.869809	-2.363366
H	-7.694605	-2.172818	-1.052865	H	-7.695153	-2.172934	-1.050657
H	3.96875	-1.7116	1.764866	H	3.968563	-1.712121	1.764704
H	3.618119	0.003867	1.631765	H	3.617978	0.003367	1.631871
Cl	4.058705	-2.668176	-0.914536	Cl	4.059103	-2.668291	-0.91473
H	7.38638	-0.280673	0.104353	H	7.386335	-0.280379	0.104549
H	6.639146	-1.884205	-0.018212	H	6.639361	-1.884012	-0.018284
H	6.28762	-0.853206	1.379907	H	6.287722	-0.853333	1.380043
H	6.079174	1.773471	-0.41478	H	6.078608	1.773707	-0.414146
H	5.073928	1.387876	0.994124	H	5.073919	1.387453	0.994965
H	4.110201	1.817629	-1.626396	H	4.109736	1.819363	-1.62506
H	5.534013	-1.073709	-2.240678	H	5.534482	-1.073033	-2.240586
Free Energy = -1857.585463 Hartree				Free Energy = -1857.585461 Hartree			

conf. 3d-3	X axis(Å)	Y axis(Å)	Z axis(Å)	conf. 3d-4	X axis(Å)	Y axis(Å)	Z axis(Å)
C	-0.053198	-3.151325	-0.496765	C	-0.150383	-2.672384	1.949359
C	1.274515	-2.960731	-0.066092	C	1.23631	-2.471896	1.803909
C	1.770511	-1.699397	0.262067	C	1.760966	-1.279849	1.305002
C	0.867809	-0.623442	0.154611	C	0.828196	-0.290293	0.94104
C	-0.487664	-0.796562	-0.230218	C	-0.574679	-0.476067	1.05508
C	-0.937303	-2.08326	-0.585477	C	-1.05999	-1.685481	1.588893
N	1.06193	0.732335	0.3688	N	1.029657	1.001845	0.486206
C	-0.110436	1.426269	0.12961	C	-0.179638	1.646116	0.30958
C	-1.106232	0.514065	-0.224056	C	-1.208076	0.761009	0.637986

C	-2.487775	0.846004	-0.549883	C	-2.636816	1.051824	0.631324
C	-3.60667	0.190411	-0.161534	C	-3.639947	0.258539	0.188413
C	-4.939687	0.68593	-0.616854	C	-5.049235	0.741799	0.287415
N	-6.018947	-0.030678	-0.174918	N	-6.000273	-0.126301	-0.175873
C	-6.007723	-1.352246	0.448966	C	-5.82659	-1.553723	-0.444433
C	-4.669613	-1.658729	1.116889	C	-4.383731	-1.881119	-0.837554
N	-3.602133	-0.896465	0.735538	N	-3.432732	-0.97405	-0.459383
C	-0.2042	2.943324	0.289118	C	-0.225779	3.110019	-0.133468
C	-0.593193	3.536974	-1.062388	C	1.139048	3.525386	-0.665524
C	-1.64228	4.316522	-1.337157	C	1.957216	4.457318	-0.169588
C	1.163382	3.560475	0.668927	C	-0.663941	3.984766	1.064609
C	-1.21342	3.284505	1.408613	C	-1.230279	3.293998	-1.303767
C	-6.374737	-2.474046	-0.541865	C	-6.812035	-2.021265	-1.517896
C	3.194666	-1.504004	0.729043	C	3.251014	-1.054001	1.186098
C	4.108555	-0.812206	-0.294747	C	3.78612	-1.139866	-0.252866
C	5.402974	-0.175605	0.271722	C	5.187963	-0.525035	-0.498371
O	6.063373	0.548523	-0.779352	O	5.496248	-0.613468	-1.899406
C	5.040666	0.930281	1.283413	C	5.143112	0.996941	-0.253462
O	4.039921	1.824351	0.795763	O	4.070656	1.632843	-0.943132
H	3.562706	-0.029754	-0.820298	H	3.090271	-0.661213	-0.941372
C	6.360307	-1.176497	0.925789	C	6.302173	-1.155027	0.342544
O	-4.568037	-2.570368	1.931274	O	-4.096412	-2.922737	-1.416016
O	-5.074912	1.692986	-1.311923	O	-5.342668	1.859452	0.712163
H	-0.387428	-4.151051	-0.760351	H	-0.510327	-3.613252	2.356686
H	1.938904	-3.818558	0.004703	H	1.921492	-3.264175	2.095058
H	-1.954428	-2.239525	-0.931081	H	-2.124609	-1.842514	1.731782
H	1.94713	1.165426	0.616586	H	1.921594	1.411273	0.228314
H	-2.675012	1.734288	-1.145964	H	-2.977948	2.014083	1.001107
H	-6.906653	0.312587	-0.526411	H	-6.950558	0.212241	-0.066407
H	-6.743192	-1.350773	1.261769	H	-6.016528	-2.131081	0.477159
H	-2.720764	-1.144425	1.17652	H	-2.477759	-1.237608	-0.686029
H	0.099738	3.287477	-1.868116	H	1.449965	2.996298	-1.568359
H	-2.384021	4.592394	-0.592823	H	1.723489	5.033527	0.721507
H	-1.80541	4.695888	-2.342513	H	2.906872	4.67235	-0.651682
H	1.545855	3.164049	1.616344	H	-1.660003	3.698704	1.4128
H	1.047551	4.643206	0.780051	H	-0.703318	5.040432	0.772274
H	1.919186	3.389304	-0.105581	H	0.026299	3.88272	1.908961
H	-1.275445	4.369784	1.550296	H	-1.232905	4.342439	-1.62273
H	-0.887299	2.837412	2.354271	H	-2.247431	3.021585	-1.01146
H	-2.215126	2.907217	1.185819	H	-0.948425	2.675104	-2.163318
H	-6.385078	-3.43895	-0.025919	H	-6.674659	-3.088075	-1.707545
H	-5.655053	-2.517397	-1.367014	H	-6.651784	-1.474908	-2.453227
H	-7.369762	-2.292453	-0.964314	H	-7.843107	-1.852938	-1.185009

H	3.627972	-2.467981	1.014168	H	3.786817	-1.768498	1.819101
H	3.186946	-0.886552	1.636925	H	3.494094	-0.054357	1.571114
Cl	4.541103	-1.993107	-1.643221	Cl	3.785261	-2.899997	-0.806812
H	7.240281	-0.647606	1.306341	H	7.256849	-0.674756	0.104508
H	6.696927	-1.922942	0.200287	H	6.394834	-2.224141	0.128551
H	5.884006	-1.704618	1.759859	H	6.112707	-1.038405	1.415889
H	5.961351	1.478854	1.519517	H	6.108506	1.410732	-0.574126
H	4.647576	0.509764	2.210952	H	5.008817	1.228737	0.80512
H	4.403472	2.256811	0.004257	H	4.185975	1.431655	-1.888018
H	6.192084	-0.049597	-1.53593	H	5.376195	-1.535442	-2.186303
Free Energy = -1857.585144 Hartree				Free Energy = -1857.584979 Hartree			

conf. 3d-5	X axis(Å)	Y axis(Å)	Z axis(Å)	conf. 3d-6	X axis(Å)	Y axis(Å)	Z axis(Å)
C	-0.068077	-2.820684	-1.806908	C	-0.38243	-3.175412	-0.534783
C	-1.447883	-2.578256	-1.659222	C	0.947808	-3.069768	-0.079722
C	-1.937924	-1.351984	-1.211043	C	1.526138	-1.840281	0.231799
C	-0.977602	-0.371534	-0.893767	C	0.703136	-0.706718	0.080855
C	0.418668	-0.599169	-1.01168	C	-0.647084	-0.789802	-0.34394
C	0.868477	-1.841702	-1.498547	C	-1.183985	-2.048813	-0.674278
N	-1.145032	0.942032	-0.483831	N	0.986754	0.631514	0.270143
C	0.085562	1.557028	-0.338473	C	-0.120426	1.407999	-0.024829
C	1.085937	0.636355	-0.648318	C	-1.168444	0.564797	-0.395593
C	2.518448	0.90844	-0.68257	C	-2.530938	0.919652	-0.797398
C	3.521594	0.111582	-0.247217	C	-3.673154	0.367755	-0.32972
C	4.935202	0.563199	-0.405714	C	-4.98475	0.821049	-0.883239
N	5.883903	-0.30444	0.067786	N	-6.093002	0.228813	-0.345948
C	5.693096	-1.720739	0.37969	C	-6.156233	-0.896189	0.584594
C	4.257892	-2.007913	0.827909	C	-4.808606	-1.193482	1.238472
N	3.312871	-1.096826	0.44694	N	-3.708975	-0.574316	0.71553
C	0.203303	3.030268	0.045651	C	0.009475	2.921171	0.144462
C	1.349779	3.211079	1.03403	C	1.118465	3.402541	-0.789271
C	2.340494	4.103539	0.969751	C	2.194431	4.129936	-0.471605
C	-1.075856	3.52612	0.770241	C	0.301432	3.242441	1.629991
C	0.387424	3.865393	-1.244736	C	-1.27557	3.676036	-0.269379
C	6.704022	-2.17887	1.433103	C	-6.711411	-2.167169	-0.084418
C	-3.421068	-1.073653	-1.123694	C	2.950576	-1.723052	0.725161
C	-3.955076	-0.923025	0.309704	C	3.898439	-1.015455	-0.257394
C	-5.306282	-0.17852	0.457933	C	5.184982	-0.40207	0.350271
O	-5.582955	0.008221	1.855819	O	5.86364	0.351864	-0.666936
C	-5.168623	1.264552	-0.065829	C	4.813818	0.666808	1.399223
O	-4.006952	1.929676	0.431999	O	3.782608	1.556927	0.971464
H	-3.226208	-0.403695	0.93055	H	3.37462	-0.215767	-0.778626

C	-6.482325	-0.867264	-0.241469	C	6.129284	-1.428138	0.98536
O	3.970015	-3.023423	1.451761	O	-4.732895	-1.975575	2.179998
O	5.239317	1.654176	-0.887335	O	-5.073467	1.689642	-1.751104
H	0.26444	-3.787214	-2.175624	H	-0.782336	-4.155466	-0.780237
H	-2.15567	-3.363656	-1.913264	H	1.546606	-3.971661	0.0233
H	1.927344	-2.030917	-1.64407	H	-2.202662	-2.138602	-1.039641
H	-2.034802	1.368964	-0.240873	H	1.885674	1.009917	0.566071
H	2.856314	1.860903	-1.076532	H	-2.688482	1.687394	-1.546679
H	6.835959	0.009227	-0.089911	H	-6.965143	0.546558	-0.755547
H	5.841933	-2.326273	-0.531542	H	-6.822215	-0.621344	1.412508
H	2.361908	-1.331284	0.717644	H	-2.828872	-0.818754	1.161677
H	1.295023	2.551259	1.901307	H	0.958929	3.134468	-1.835303
H	2.471985	4.782531	0.131804	H	2.41616	4.438937	0.546405
H	3.084775	4.165271	1.759032	H	2.890326	4.45735	-1.240944
H	-1.963661	3.473764	0.131204	H	-0.508944	2.858146	2.258587
H	-0.937545	4.571905	1.06208	H	0.361518	4.326063	1.78261
H	-1.26921	2.948512	1.68237	H	1.23994	2.798549	1.976756
H	0.420257	4.936182	-1.012327	H	-1.130537	4.74612	-0.086911
H	-0.453539	3.693981	-1.925474	H	-2.142277	3.342306	0.309391
H	1.308513	3.60023	-1.7731	H	-1.502821	3.550622	-1.332364
H	6.554446	-3.237838	1.654911	H	-6.775947	-2.975321	0.64976
H	6.583181	-1.606006	2.358432	H	-6.066946	-2.485122	-0.911434
H	7.726707	-2.037665	1.063636	H	-7.713867	-1.975076	-0.484094
H	-3.978628	-1.860432	-1.641656	H	3.345268	-2.714537	0.968566
H	-3.634178	-0.138681	-1.659	H	2.952583	-1.149334	1.661614
Cl	-4.07241	-2.58016	1.107807	Cl	4.349247	-2.168354	-1.625246
H	-7.392278	-0.275041	-0.09901	H	6.994734	-0.914127	1.416664
H	-6.654083	-1.863579	0.176447	H	6.492104	-2.137239	0.23556
H	-6.303352	-0.976534	-1.317342	H	5.631678	-1.998036	1.778598
H	-6.074909	1.811267	0.223732	H	5.727267	1.226544	1.636189
H	-5.089204	1.288472	-1.15427	H	4.449052	0.205596	2.319175
H	-4.091729	1.974618	1.399774	H	4.104143	2.059576	0.203668
H	-5.535496	-0.855558	2.301186	H	6.001008	-0.223681	-1.439299
Free Energy = -1857.584463 Hartree				Free Energy = -1857.584425 Hartree			

**conf. 3d-7**	**X axis(Å)**	**Y axis(Å)**	**Z axis(Å)**	**conf. 3d-8**	**X axis(Å)**	**Y axis(Å)**	**Z axis(Å)**
C	-0.084117	-3.185296	-0.408638	C	-0.294143	-3.1732	-0.301751
C	1.245463	-2.99762	0.018735	C	1.031046	-2.991633	0.142888
C	1.753973	-1.732449	0.310435	C	1.580317	-1.723659	0.328829
C	0.862667	-0.651437	0.168527	C	0.73374	-0.629983	0.060697
C	-0.491385	-0.818229	-0.219882	C	-0.616199	-0.7873	-0.344551
C	-0.955512	-2.109841	-0.534535	C	-1.121955	-2.084868	-0.550104

N	1.069651	0.704088	0.353273	N	0.979479	0.727527	0.126694
C	-0.092539	1.408649	0.088851	C	-0.15456	1.445009	-0.217914
C	-1.093445	0.502192	-0.25807	C	-1.178123	0.542086	-0.505028
C	-2.464233	0.813456	-0.66006	C	-2.552221	0.822188	-0.926326
C	-3.591241	0.185842	-0.252967	C	-3.676613	0.282971	-0.404176
C	-4.910333	0.615465	-0.805666	C	-5.003097	0.6526	-0.984268
N	-6.001117	-0.056269	-0.323724	N	-6.091952	0.082688	-0.386395
C	-6.01181	-1.289889	0.459911	C	-6.117501	-0.969479	0.62752
C	-4.685882	-1.528992	1.177838	C	-4.760898	-1.168429	1.299703
N	-3.608603	-0.810662	0.743037	N	-3.681569	-0.563763	0.720273
C	-0.089436	2.933312	0.166911	C	-0.083234	2.970148	-0.141662
C	-1.497278	3.455854	0.413058	C	0.183159	3.334941	1.317754
C	-2.132003	4.415797	-0.264154	C	1.231163	3.99876	1.81376
C	0.528456	3.496096	-1.135594	C	-1.411735	3.648398	-0.546299
C	0.764105	3.409817	1.378027	C	1.02211	3.486345	-1.092761
C	-6.380718	-2.520515	-0.390877	C	-6.627605	-2.30694	0.059227
C	3.179833	-1.527116	0.768279	C	2.997334	-1.526097	0.816797
C	4.082618	-0.853171	-0.278994	C	3.968416	-1.017883	-0.262186
C	5.351083	-0.147354	0.264303	C	5.252529	-0.317529	0.251443
O	6.007738	0.53044	-0.819539	O	5.993836	0.180769	-0.874879
C	4.949408	1.010046	1.200404	C	4.879099	0.971544	1.011401
O	3.958255	1.866316	0.629422	O	3.977609	1.800338	0.278479
H	3.515642	-0.116623	-0.847156	H	3.459056	-0.323892	-0.929936
C	6.32318	-1.082189	0.990481	C	6.135109	-1.202043	1.136795
O	-4.60228	-2.354854	2.080953	O	-4.65979	-1.863985	2.304555
O	-5.02498	1.534309	-1.616401	O	-5.119873	1.44036	-1.922897
H	-0.429807	-4.188802	-0.641292	H	-0.671208	-4.181868	-0.446895
H	1.899748	-3.860697	0.115981	H	1.648678	-3.864117	0.342024
H	-1.973819	-2.265187	-0.877222	H	-2.139225	-2.234251	-0.89956
H	1.962682	1.136498	0.572695	H	1.869642	1.15922	0.358803
H	-2.636391	1.634284	-1.347495	H	-2.733244	1.516795	-1.739063
H	-6.87746	0.238852	-0.741286	H	-6.974679	0.340042	-0.815275
H	-6.756938	-1.181566	1.256963	H	-6.793396	-0.65465	1.43261
H	-2.736469	-1.016026	1.222857	H	-2.793212	-0.742897	1.180781
H	-1.992585	3.013309	1.277982	H	-0.602427	3.007968	2.00133
H	-1.710719	4.900033	-1.141073	H	2.055234	4.346275	1.197088
H	-3.12717	4.737239	0.030585	H	1.298704	4.215602	2.877073
H	1.536314	3.093386	-1.284362	H	-1.65654	3.457474	-1.595992
H	0.608436	4.588168	-1.088088	H	-1.308074	4.731208	-0.417563
H	-0.073253	3.229244	-2.010726	H	-2.252259	3.314372	0.068985
H	0.710951	4.501373	1.447506	H	1.057643	4.581462	-1.072103
H	1.819324	3.134125	1.279266	H	0.806727	3.172371	-2.119832
H	0.384041	2.991783	2.317242	H	2.014473	3.11406	-0.822717

H	-6.411572	-3.414067	0.239759	H	-6.665382	-3.057972	0.85351
H	-5.649765	-2.673366	-1.19271	H	-5.971401	-2.665173	-0.741678
H	-7.366996	-2.380085	-0.848116	H	-7.635459	-2.180833	-0.352896
H	3.618309	-2.48363	1.06988	H	3.3797	-2.458457	1.244248
H	3.17549	-0.892767	1.664874	H	2.989248	-0.792106	1.63373
Cl	4.565908	-2.082863	-1.565182	Cl	4.430515	-2.409957	-1.380199
H	7.187431	-0.510933	1.344844	H	7.01902	-0.638792	1.453336
H	6.684182	-1.866346	0.318231	H	6.471896	-2.088257	0.590609
H	5.848693	-1.566059	1.852007	H	5.59787	-1.537814	2.031274
H	5.857601	1.580945	1.431341	H	5.810918	1.51215	1.222426
H	4.530053	0.641623	2.138434	H	4.389689	0.751866	1.962273
H	4.330736	2.225676	-0.194394	H	4.4055	1.996734	-0.573193
H	6.158365	-0.106062	-1.539952	H	6.130971	-0.547976	-1.504833
Free Energy = -1857.58436 Hartree				Free Energy = -1857.584302 Hartree			

Table S24. The Cartesian coordinates of the lowest energy conformers (Conf. **1-7**) for isomer **4a**.

conf. 4a-1	X axis(Å)	Y axis(Å)	Z axis(Å)	conf. 4a-2	X axis(Å)	Y axis(Å)	Z axis(Å)
C	-0.136226	-2.677557	-1.73852	C	0.059602	-3.030162	0.208732
C	-1.488149	-2.296958	-1.625978	C	1.316175	-2.769908	-0.375652
C	-1.866549	-1.011262	-1.23262	C	1.72183	-1.480541	-0.730632
C	-0.814828	-0.130511	-0.922017	C	0.786073	-0.457478	-0.483666
C	0.556321	-0.49097	-1.006266	C	-0.506117	-0.695199	0.057631
C	0.889933	-1.784845	-1.450558	C	-0.855178	-2.007935	0.430942
N	-0.868007	1.201373	-0.560757	N	0.896778	0.901627	-0.68792
C	0.403629	1.703892	-0.389814	C	-0.251098	1.54707	-0.288832
C	1.326893	0.689702	-0.655949	C	-1.167398	0.592181	0.163521
C	2.775206	0.837757	-0.644144	C	-2.494813	0.854732	0.705108
C	3.701588	-0.041252	-0.194945	C	-3.636067	0.16348	0.477375
C	5.148654	0.302306	-0.308713	C	-4.895589	0.591094	1.15522
N	6.013967	-0.649836	0.164324	N	-5.998963	-0.167934	0.873054
C	5.700119	-2.057509	0.410012	C	-6.011688	-1.501077	0.2708
C	4.24346	-2.233365	0.847374	C	-4.813349	-1.708815	-0.659126
N	3.38288	-1.238692	0.476848	N	-3.737398	-0.891577	-0.449272
C	0.629316	3.163099	0.000326	C	-0.344431	3.071279	-0.371905
C	1.621644	3.22636	1.156185	C	0.762058	3.59691	-1.277985
C	2.739876	3.952114	1.226745	C	1.757737	4.425912	-0.95131
C	-0.686598	3.808831	0.508015	C	-0.257538	3.664142	1.053614
C	1.102871	3.952717	-1.243367	C	-1.68407	3.503732	-1.030046
C	6.664369	-2.649723	1.439206	C	-7.331539	-1.744339	-0.46425
C	-3.32685	-0.600167	-1.233836	C	3.049224	-1.256298	-1.430349
C	-3.939872	-0.333787	0.156943	C	4.156633	-0.384478	-0.794545

C	-5.490582	-0.407345	0.161584	C	4.616841	-0.785972	0.629302
C	-6.163122	0.612217	-0.750616	C	3.640621	-0.466153	1.754398
C	-6.036947	-1.822475	-0.096535	C	5.083258	-2.246636	0.708847
O	-5.44222	-2.838136	0.688494	O	5.990278	-2.633769	-0.309365
O	-3.494869	0.970641	0.555826	O	3.725962	0.981194	-0.844758
Cl	-6.004864	0.003106	1.926898	Cl	6.16087	0.263783	0.943172
O	3.866438	-3.231562	1.451901	O	-4.805162	-2.591231	-1.509861
O	5.550567	1.37707	-0.754592	O	-4.958578	1.582487	1.882541
H	0.104197	-3.684035	-2.070335	H	-0.198085	-4.050915	0.478231
H	-2.262115	-3.019439	-1.875687	H	1.987704	-3.604017	-0.569256
H	1.925971	-2.08229	-1.577431	H	-1.815853	-2.221596	0.88902
H	-1.72194	1.622262	-0.213096	H	1.775205	1.355619	-0.924172
H	3.202191	1.766972	-1.005691	H	-2.630249	1.706905	1.36467
H	6.991393	-0.411779	0.031562	H	-6.833394	0.122995	1.371766
H	5.796854	-2.631328	-0.528657	H	-5.90625	-2.266465	1.059425
H	2.413366	-1.393418	0.738226	H	-2.93662	-1.076914	-1.046815
H	1.334757	2.621361	2.018033	H	0.686561	3.262417	-2.314602
H	3.356698	3.936691	2.121346	H	2.470326	4.76123	-1.700865
H	3.102151	4.563188	0.404635	H	1.900762	4.807895	0.055908
H	-1.458877	3.823008	-0.270817	H	-1.075804	3.293176	1.677296
H	-0.489797	4.845387	0.798558	H	-0.33189	4.757248	1.020319
H	-1.083551	3.287683	1.387433	H	0.684197	3.394327	1.543898
H	2.054074	3.57844	-1.632887	H	-1.783244	3.077792	-2.034898
H	1.228111	5.014384	-1.000473	H	-1.713319	4.59581	-1.116599
H	0.357859	3.87323	-2.04245	H	-2.547731	3.183927	-0.441851
H	7.69642	-2.580983	1.075044	H	-8.173629	-1.669009	0.233968
H	6.591175	-2.11231	2.390381	H	-7.470314	-1.007464	-1.26221
H	6.422949	-3.700753	1.612336	H	-7.331204	-2.743047	-0.906384
H	-3.46455	0.307728	-1.837381	H	3.498488	-2.231809	-1.642959
H	-3.889059	-1.396977	-1.728812	H	2.864869	-0.790859	-2.409009
H	-3.566717	-1.084017	0.861605	H	5.02465	-0.502311	-1.452208
H	-7.249915	0.589938	-0.626642	H	4.101483	-0.663165	2.727155
H	-5.932922	0.377792	-1.797392	H	2.748471	-1.092664	1.656427
H	-5.804917	1.622315	-0.53894	H	3.328112	0.579623	1.720665
H	-5.830695	-2.092625	-1.137169	H	4.203844	-2.887311	0.588482
H	-7.12829	-1.803241	0.029139	H	5.49956	-2.433193	1.708015
H	-5.694242	-2.698461	1.61654	H	6.82322	-2.151532	-0.175183
H	-3.78404	1.126837	1.470749	H	4.465368	1.551497	-0.5748
Free Energy = -1857.58424 Hartree				Free Energy = -1857.582993 Hartree			

conf. 4a-3	X axis(Å)	Y axis(Å)	Z axis(Å)	conf. 4a-4	X axis(Å)	Y axis(Å)	Z axis(Å)
C	0.040827	-3.012307	0.295371	C	0.325139	-3.014615	0.231712

C	1.282426	-2.754135	-0.321482	C	1.565144	-2.703365	-0.363264
C	1.685318	-1.465617	-0.68258	C	1.907174	-1.400288	-0.734431
C	0.760695	-0.438559	-0.408537	C	0.925122	-0.421029	-0.493913
C	-0.513419	-0.674335	0.172859	C	-0.352305	-0.71039	0.056636
C	-0.861357	-1.986094	0.548133	C	-0.635951	-2.03404	0.447366
N	0.866002	0.919257	-0.612996	N	0.976904	0.939355	-0.712446
C	-0.26797	1.568904	-0.169283	C	-0.194434	1.538549	-0.308127
C	-1.170314	0.614053	0.306463	C	-1.068795	0.548088	0.15012
C	-2.505929	0.832318	0.859223	C	-2.412591	0.774045	0.66387
C	-3.631742	0.146132	0.555598	C	-3.519482	0.019496	0.469253
C	-4.908611	0.492329	1.249308	C	-4.79961	0.423428	1.120967
N	-5.99667	-0.251134	0.880545	N	-5.868447	-0.395992	0.869642
C	-5.988755	-1.51727	0.148253	C	-5.814839	-1.761531	0.347926
C	-4.761239	-1.639149	-0.75789	C	-4.606039	-1.962427	-0.569677
N	-3.700178	-0.831962	-0.454506	N	-3.569518	-1.087819	-0.400573
C	-0.353836	3.078425	-0.392474	C	-0.381811	3.05207	-0.401332
C	0.980682	3.692527	0.019651	C	-1.676374	3.345029	-1.152641
C	1.837118	4.370487	-0.750107	C	-2.69194	4.112785	-0.751095
C	-1.439019	3.752677	0.479184	C	0.763872	3.700988	-1.221639
C	-0.687661	3.336195	-1.881648	C	-0.359158	3.661992	1.020099
C	-7.284142	-1.683768	-0.649963	C	-7.119662	-2.1122	-0.370308
C	2.993751	-1.246056	-1.418368	C	3.2175	-1.116818	-1.444956
C	4.121595	-0.381049	-0.81012	C	4.286698	-0.186516	-0.826572
C	4.617268	-0.788229	0.600083	C	4.771491	-0.547441	0.599558
C	3.674325	-0.46433	1.751983	C	3.785677	-0.262395	1.72564
C	5.077705	-2.251408	0.664696	C	5.309053	-1.982695	0.694073
O	5.954627	-2.641671	-0.378499	O	6.224416	-2.339293	-0.327787
O	3.695061	0.986092	-0.845218	O	3.801428	1.162947	-0.891156
Cl	6.175794	0.252489	0.874025	Cl	6.264172	0.580415	0.892678
O	-4.719536	-2.452513	-1.673908	O	-4.555495	-2.885415	-1.375409
O	-4.996684	1.410831	2.063777	O	-4.911544	1.441043	1.804591
H	-0.214502	-4.033591	0.565029	H	0.117294	-4.042822	0.515441
H	1.944303	-3.590528	-0.537457	H	2.273866	-3.507307	-0.551632
H	-1.812396	-2.194379	1.028835	H	-1.582196	-2.28757	0.914726
H	1.740406	1.37485	-0.861289	H	1.83611	1.413546	-0.969613
H	-2.655518	1.614877	1.594612	H	-2.597319	1.663912	1.255598
H	-6.843022	-0.015528	1.38835	H	-6.714514	-0.121292	1.357866
H	-5.909216	-2.357115	0.860194	H	-5.673742	-2.473644	1.179943
H	-2.876564	-0.973797	-1.032756	H	-2.76401	-1.261656	-0.994853
H	1.22022	3.560169	1.076597	H	-1.72751	2.880458	-2.138794
H	2.749691	4.789238	-0.332874	H	-3.557163	4.268317	-1.390033
H	1.667615	4.543809	-1.809429	H	-2.722135	4.593095	0.223018
H	-2.44335	3.413128	0.211261	H	1.739537	3.561387	-0.73942

H	-1.397291	4.836188	0.323779	H	0.585652	4.778078	-1.297696
H	-1.281087	3.560519	1.546129	H	0.816712	3.297528	-2.239262
H	0.064124	2.903638	-2.550013	H	-1.169273	3.281468	1.648309
H	-0.752333	4.412199	-2.081401	H	-0.450194	4.753211	0.970862
H	-1.654641	2.885772	-2.128141	H	0.588968	3.42436	1.514895
H	-8.148511	-1.674619	0.024643	H	-7.9657	-2.037428	0.323219
H	-7.397417	-0.87166	-1.375712	H	-7.292767	-1.430915	-1.209792
H	-7.268572	-2.634598	-1.187214	H	-7.069626	-3.13386	-0.75328
H	3.432318	-2.222994	-1.646292	H	3.713949	-2.070873	-1.650049
H	2.784995	-0.77636	-2.390078	H	3.004864	-0.672859	-2.427972
H	4.97165	-0.500918	-1.490521	H	5.155477	-0.273719	-1.487608
H	4.160826	-0.664849	2.711408	H	4.259258	-0.427367	2.698212
H	2.776188	-1.085621	1.678425	H	2.923775	-0.931272	1.6377
H	3.366862	0.583187	1.727625	H	3.424137	0.767126	1.683298
H	4.191904	-2.887156	0.567216	H	4.461028	-2.667193	0.591428
H	5.52019	-2.44196	1.65178	H	5.743141	-2.134597	1.691448
H	6.792856	-2.162705	-0.267291	H	7.037556	-1.821964	-0.203721
H	4.443653	1.553024	-0.594482	H	4.52763	1.759416	-0.641637
Free Energy = -1857.582728 Hartree				Free Energy = -1857.582462 Hartree			

conf. 4a-5	X axis(Å)	Y axis(Å)	Z axis(Å)	conf. 4a-6	X axis(Å)	Y axis(Å)	Z axis(Å)
C	0.040827	-3.012307	0.295371	C	0.341468	-2.491248	-1.943275
C	1.282426	-2.754135	-0.321482	C	1.67745	-2.040802	-1.924771
C	1.685318	-1.465617	-0.68258	C	2.022012	-0.74699	-1.523927
C	0.760695	-0.438559	-0.408537	C	0.950547	0.073996	-1.126671
C	-0.513419	-0.674335	0.172859	C	-0.402911	-0.357204	-1.113619
C	-0.861357	-1.986094	0.548133	C	-0.703278	-1.660924	-1.554337
N	0.866002	0.919257	-0.612996	N	0.961497	1.399831	-0.745281
C	-0.26797	1.568904	-0.169283	C	-0.318209	1.83393	-0.477078
C	-1.170314	0.614053	0.306463	C	-1.204654	0.775073	-0.687288
C	-2.505929	0.832318	0.859223	C	-2.655628	0.837072	-0.560329
C	-3.631742	0.146132	0.555598	C	-3.474742	-0.090904	-0.013769
C	-4.908611	0.492329	1.249308	C	-4.947945	0.145998	-0.004894
N	-5.99667	-0.251134	0.880545	N	-5.700403	-0.833728	0.58571
C	-5.988755	-1.51727	0.148253	C	-5.268642	-2.180197	0.955897
C	-4.761239	-1.639149	-0.75789	C	-3.75896	-2.260775	1.167202
N	-3.700178	-0.831962	-0.454506	N	-3.008655	-1.2489	0.640512
C	-0.353836	3.078425	-0.392474	C	-0.590968	3.279187	-0.067329
C	0.980682	3.692527	0.019651	C	-1.533848	3.29583	1.130413
C	1.837118	4.370487	-0.750107	C	-2.671351	3.983123	1.256177
C	-1.439019	3.752677	0.479184	C	-1.154443	4.047404	-1.286626
C	-0.687661	3.336195	-1.881648	C	0.716762	3.980946	0.385148

C	-7.284142	-1.683768	-0.649963	C	-5.716652	-3.242749	-0.066511
C	2.993751	-1.246056	-1.418368	C	3.451892	-0.254793	-1.641877
C	4.121595	-0.381049	-0.81012	C	4.246072	0.203948	-0.397377
C	4.617268	-0.788229	0.600083	C	4.364642	-0.819993	0.759184
C	3.674325	-0.46433	1.751983	C	3.096865	-1.04782	1.572909
C	5.077705	-2.251408	0.664696	C	4.968838	-2.156078	0.303468
O	5.954627	-2.641671	-0.378499	O	6.145884	-2.036639	-0.477401
O	3.695061	0.986092	-0.845218	O	3.688636	1.446426	0.05539
Cl	6.175794	0.252489	0.874025	Cl	5.632983	-0.05733	1.938931
O	-4.719536	-2.452513	-1.673908	O	-3.256054	-3.212808	1.755722
O	-4.996684	1.410831	2.063777	O	-5.462342	1.165725	-0.464272
H	-0.214502	-4.033591	0.565029	H	0.128119	-3.500968	-2.283676
H	1.944303	-3.590528	-0.537457	H	2.462667	-2.712183	-2.266979
H	-1.812396	-2.194379	1.028835	H	-1.729886	-2.009803	-1.605964
H	1.740406	1.37485	-0.861289	H	1.818778	1.876838	-0.487102
H	-2.655518	1.614877	1.594612	H	-3.172037	1.725673	-0.907721
H	-6.843022	-0.015528	1.38835	H	-6.698959	-0.658373	0.546613
H	-5.909216	-2.357115	0.860194	H	-5.710804	-2.424537	1.928782
H	-2.876564	-0.973797	-1.032756	H	-2.006596	-1.343316	0.779481
H	1.22022	3.560169	1.076597	H	-1.189272	2.694216	1.973214
H	2.749691	4.789238	-0.332874	H	-3.248039	3.940052	2.176223
H	1.667615	4.543809	-1.809429	H	-3.087881	4.588966	0.456158
H	-2.44335	3.413128	0.211261	H	-0.444865	3.998656	-2.119604
H	-1.397291	4.836188	0.323779	H	-1.31371	5.103216	-1.038282
H	-1.281087	3.560519	1.546129	H	-2.105781	3.630389	-1.630003
H	0.064124	2.903638	-2.550013	H	1.179477	3.470338	1.238106
H	-0.752333	4.412199	-2.081401	H	0.486239	5.004583	0.695909
H	-1.654641	2.885772	-2.128141	H	1.449645	4.03894	-0.428927
H	-8.148511	-1.674619	0.024643	H	-6.808565	-3.236181	-0.162422
H	-7.397417	-0.87166	-1.375712	H	-5.284766	-3.041008	-1.05312
H	-7.268572	-2.634598	-1.187214	H	-5.401043	-4.23649	0.26545
H	3.432318	-2.222994	-1.646292	H	4.049323	-1.032415	-2.128696
H	2.784995	-0.77636	-2.390078	H	3.478735	0.611095	-2.319078
H	4.97165	-0.500918	-1.490521	H	5.262538	0.390965	-0.759765
H	4.160826	-0.664849	2.711408	H	3.309747	-1.671238	2.4466
H	2.776188	-1.085621	1.678425	H	2.349913	-1.556209	0.955026
H	3.366862	0.583187	1.727625	H	2.671488	-0.101357	1.913278
H	4.191904	-2.887156	0.567216	H	4.233709	-2.657318	-0.334158
H	5.52019	-2.44196	1.65178	H	5.13504	-2.790204	1.184567
H	6.792856	-2.162705	-0.267291	H	6.855839	-1.692983	0.090109
H	4.443653	1.553024	-0.594482	H	4.268283	1.807038	0.747739
Free Energy = -1857.582461 Hartree				Free Energy = -1857.58205 Hartree			

conf. 4a-7	X axis(Å)	Y axis(Å)	Z axis(Å)
C	0.040827	-3.012307	0.295371
C	1.282426	-2.754135	-0.321482
C	1.685318	-1.465617	-0.68258
C	0.760695	-0.438559	-0.408537
C	-0.513419	-0.674335	0.172859
C	-0.861357	-1.986094	0.548133
N	0.866002	0.919257	-0.612996
C	-0.26797	1.568904	-0.169283
C	-1.170314	0.614053	0.306463
C	-2.505929	0.832318	0.859223
C	-3.631742	0.146132	0.555598
C	-4.908611	0.492329	1.249308
N	-5.99667	-0.251134	0.880545
C	-5.988755	-1.51727	0.148253
C	-4.761239	-1.639149	-0.75789
N	-3.700178	-0.831962	-0.454506
C	-0.353836	3.078425	-0.392474
C	0.980682	3.692527	0.019651
C	1.837118	4.370487	-0.750107
C	-1.439019	3.752677	0.479184
C	-0.687661	3.336195	-1.881648
C	-7.284142	-1.683768	-0.649963
C	2.993751	-1.246056	-1.418368
C	4.121595	-0.381049	-0.81012
C	4.617268	-0.788229	0.600083
C	3.674325	-0.46433	1.751983
C	5.077705	-2.251408	0.664696
O	5.954627	-2.641671	-0.378499
O	3.695061	0.986092	-0.845218
Cl	6.175794	0.252489	0.874025
O	-4.719536	-2.452513	-1.673908
O	-4.996684	1.410831	2.063777
H	-0.214502	-4.033591	0.565029
H	1.944303	-3.590528	-0.537457
H	-1.812396	-2.194379	1.028835
H	1.740406	1.37485	-0.861289
H	-2.655518	1.614877	1.594612
H	-6.843022	-0.015528	1.38835
H	-5.909216	-2.357115	0.860194
H	-2.876564	-0.973797	-1.032756

H	1.22022	3.560169	1.076597
H	2.749691	4.789238	-0.332874
H	1.667615	4.543809	-1.809429
H	-2.44335	3.413128	0.211261
H	-1.397291	4.836188	0.323779
H	-1.281087	3.560519	1.546129
H	0.064124	2.903638	-2.550013
H	-0.752333	4.412199	-2.081401
H	-1.654641	2.885772	-2.128141
H	-8.148511	-1.674619	0.024643
H	-7.397417	-0.87166	-1.375712
H	-7.268572	-2.634598	-1.187214
H	3.432318	-2.222994	-1.646292
H	2.784995	-0.77636	-2.390078
H	4.97165	-0.500918	-1.490521
H	4.160826	-0.664849	2.711408
H	2.776188	-1.085621	1.678425
H	3.366862	0.583187	1.727625
H	4.191904	-2.887156	0.567216
H	5.52019	-2.44196	1.65178
H	6.792856	-2.162705	-0.267291
H	4.443653	1.553024	-0.594482
Free Energy = -1857.582046 Hartree			

Table S25. The Cartesian coordinates of the lowest energy conformers (Conf. **1-4**) for isomer **4b**.

conf. 4b-1	X axis(Å)	Y axis(Å)	Z axis(Å)	conf. 4b-2	X axis(Å)	Y axis(Å)	Z axis(Å)
C	0.040701	-2.683241	-1.848388	C	0.266358	-2.736492	-1.762701
C	-1.325079	-2.367269	-1.703793	C	-1.110943	-2.469096	-1.630448
C	-1.75394	-1.127649	-1.223716	C	-1.590512	-1.231191	-1.195936
C	-0.737457	-0.223908	-0.864415	C	-0.611916	-0.273826	-0.869454
C	0.647085	-0.521037	-0.979294	C	0.783323	-0.520999	-0.976509
C	1.030666	-1.769498	-1.506521	C	1.218185	-1.771517	-1.455707
N	-0.841801	1.077807	-0.416972	N	-0.76191	1.037137	-0.467724
C	0.408592	1.62246	-0.225645	C	0.466046	1.636336	-0.298057
C	1.371047	0.665569	-0.561632	C	1.463312	0.707012	-0.606521
C	2.811814	0.873476	-0.538419	C	2.899814	0.953824	-0.642758
C	3.769202	0.010388	-0.123604	C	3.888274	0.148475	-0.189388
C	5.203119	0.417946	-0.190156	C	5.309813	0.576721	-0.343195
N	6.099617	-0.527541	0.233985	N	6.241454	-0.304888	0.134856
C	5.840305	-1.957486	0.401484	C	6.023991	-1.714342	0.461028
C	4.385108	-2.215634	0.800901	C	4.582957	-1.971094	0.909859

N	3.490743	-1.234785	0.475471	N	3.655292	-1.045994	0.519223
C	0.585175	3.054601	0.276335	C	0.548025	3.105695	0.117382
C	1.238392	3.860586	-0.843891	C	-0.760295	3.513902	0.783761
C	2.393472	4.529761	-0.794735	C	-1.645157	4.420019	0.358162
C	1.411914	3.060362	1.581067	C	0.855256	3.960322	-1.134587
C	-0.785135	3.714542	0.571931	C	1.659526	3.327071	1.1782
C	6.810539	-2.562716	1.417731	C	7.025102	-2.179906	1.520547
C	-3.230815	-0.785208	-1.17916	C	-3.080516	-0.95034	-1.165975
C	-3.816752	-0.552569	0.232731	C	-3.683367	-0.733006	0.240374
C	-5.340955	-0.835224	0.319183	C	-5.20319	-1.043253	0.312213
Cl	-5.834707	-0.378943	2.079999	Cl	-5.71885	-0.604397	2.071731
C	-6.168242	0.071741	-0.607154	C	-6.03844	-0.145871	-0.615855
O	-7.566741	-0.138819	-0.515718	O	-7.434395	-0.378464	-0.534592
O	-3.516174	0.807026	0.577169	O	-3.40826	0.628795	0.587621
C	-5.676533	-2.315099	0.158421	C	-5.512731	-2.52762	0.141364
O	4.038661	-3.262594	1.33724	O	4.275307	-2.975661	1.541544
O	5.565725	1.536729	-0.553494	O	5.631098	1.664212	-0.822503
H	0.32002	-3.65471	-2.247228	H	0.584353	-3.710311	-2.125293
H	-2.069799	-3.102355	-2.002166	H	-1.825427	-3.24327	-1.90268
H	2.077341	-2.015071	-1.655873	H	2.274529	-1.979232	-1.594883
H	-1.717025	1.459074	-0.074778	H	-1.647373	1.423247	-0.1567
H	3.190941	1.840231	-0.857463	H	3.259323	1.886448	-1.06808
H	7.068494	-0.241034	0.140134	H	7.199959	-0.00618	-0.012507
H	5.977215	-2.477655	-0.562984	H	6.162977	-2.331341	-0.443889
H	2.523876	-1.445351	0.705891	H	2.69823	-1.261648	0.784266
H	0.662516	3.873609	-1.771008	H	-0.953346	3.007753	1.732394
H	2.755489	5.079023	-1.65995	H	-2.532922	4.64948	0.942335
H	3.028255	4.547729	0.086781	H	-1.525151	4.969752	-0.57143
H	0.898827	2.474139	2.351619	H	1.813939	3.66623	-1.572201
H	1.528118	4.084347	1.954318	H	0.917786	5.022378	-0.870151
H	2.407053	2.630401	1.440224	H	0.085529	3.836816	-1.903887
H	-1.42134	3.764766	-0.319441	H	1.486219	2.707375	2.065382
H	-0.623292	4.740395	0.917515	H	1.661718	4.37776	1.489553
H	-1.327717	3.179228	1.361449	H	2.650825	3.085705	0.787832
H	7.84485	-2.435606	1.076511	H	8.050604	-2.060588	1.151281
H	6.700446	-2.077344	2.393054	H	6.913136	-1.596236	2.440204
H	6.608346	-3.62978	1.533678	H	6.85645	-3.233874	1.752164
H	-3.428714	0.121556	-1.767681	H	-3.310606	-0.056543	-1.762311
H	-3.770529	-1.600276	-1.668732	H	-3.582623	-1.790949	-1.652614
H	-3.321913	-1.226726	0.944554	H	-3.183337	-1.402111	0.953694
H	-5.905155	-0.174006	-1.641641	H	-5.76489	-0.383625	-1.649534
H	-5.905424	1.120939	-0.42902	H	-5.792927	0.906445	-0.431933
H	-7.865409	0.157464	0.359923	H	-7.742003	-0.094089	0.341916

H	-3.836542	0.968537	1.481584	H	-3.72333	0.782873	1.494743
H	-6.736682	-2.487609	0.353757	H	-6.570802	-2.719677	0.329751
H	-5.082964	-2.930389	0.841519	H	-4.912426	-3.13668	0.82424
H	-5.474291	-2.637966	-0.869039	H	-5.299321	-2.840947	-0.88694
Free Energy = -1857.584417 Hartree				Free Energy = -1857.584376 Hartree			

conf. 4b-3	X axis(Å)	Y axis(Å)	Z axis(Å)	conf. 4b-4	X axis(Å)	Y axis(Å)	Z axis(Å)
C	-0.341302	-3.010721	-0.935044	C	0.278557	-2.704304	-1.879874
C	0.974559	-2.8354	-0.460452	C	-1.096881	-2.442477	-1.715023
C	1.453496	-1.597464	-0.025651	C	-1.572575	-1.207696	-1.268196
C	0.537148	-0.529596	-0.085888	C	-0.590778	-0.246384	-0.961414
C	-0.805139	-0.68648	-0.522436	C	0.801274	-0.485116	-1.111741
C	-1.237704	-1.949406	-0.969598	C	1.232333	-1.73387	-1.598056
N	0.705774	0.800721	0.229284	N	-0.733419	1.060874	-0.551084
C	-0.454805	1.509446	-0.012699	C	0.497413	1.670801	-0.425707
C	-1.435057	0.61989	-0.454878	C	1.488133	0.745452	-0.762924
C	-2.825509	0.903779	-0.81269	C	2.934891	0.9495	-0.796564
C	-3.918116	0.228607	-0.389849	C	3.878257	0.135879	-0.268859
C	-5.263302	0.614384	-0.911152	C	5.320798	0.489802	-0.42207
N	-6.31894	-0.102858	-0.42276	N	6.204057	-0.385661	0.148905
C	-6.32887	-1.095515	0.6497	C	5.925863	-1.74578	0.609411
C	-4.924158	-1.482733	1.111582	C	4.456995	-1.923839	1.001091
N	-3.870949	-0.814028	0.554725	N	3.578746	-1.000608	0.506449
C	-0.469863	2.994931	0.344	C	0.546439	3.088185	0.144171
C	0.81888	3.619807	-0.181472	C	-0.6604	3.855139	-0.38575
C	1.782618	4.215979	0.526978	C	-1.647942	4.408625	0.324129
C	-0.609486	3.131461	1.879224	C	1.803333	3.867522	-0.314158
C	-1.637339	3.75491	-0.327523	C	0.557146	2.998024	1.689348
C	-7.124485	-2.342703	0.238659	C	6.855198	-2.121922	1.766355
C	2.86195	-1.46732	0.52249	C	-3.062566	-0.933692	-1.199897
C	3.821222	-0.591585	-0.316129	C	-3.63042	-0.71779	0.221336
C	5.317617	-0.956613	-0.120836	C	-5.146163	-1.036745	0.332414
Cl	6.275345	0.304628	-1.143783	Cl	-5.618896	-0.600269	2.104063
C	5.787777	-0.777873	1.332204	C	-6.010037	-0.144627	-0.574346
O	7.164568	-1.052444	1.527567	O	-7.402182	-0.384578	-0.456724
O	3.559624	0.763946	0.064371	O	-3.354469	0.646117	0.559802
C	5.674773	-2.323661	-0.697373	C	-5.451448	-2.523038	0.169847
O	-4.766397	-2.348153	1.965986	O	4.087817	-2.873717	1.682591
O	-5.420759	1.5081	-1.743078	O	5.69857	1.519018	-0.981408
H	-0.656766	-3.994953	-1.270641	H	0.591816	-3.677713	-2.247611
H	1.638664	-3.69663	-0.423267	H	-1.813729	-3.220586	-1.969401
H	-2.24749	-2.091486	-1.343809	H	2.287265	-1.934499	-1.759381

H	1.618005	1.215504	0.397889	H	-1.612015	1.447104	-0.220934
H	-3.046933	1.714251	-1.498698	H	3.337186	1.828089	-1.288828
H	-7.217175	0.19261	-0.790662	H	7.176832	-0.135634	0.004451
H	-6.808757	-0.659006	1.539541	H	6.094441	-2.458436	-0.216361
H	-2.959584	-1.085151	0.914327	H	2.603147	-1.17584	0.730919
H	0.921066	3.573623	-1.267535	H	-0.676891	3.958984	-1.472213
H	2.646594	4.655063	0.033943	H	-2.447817	4.956965	-0.167086
H	1.750974	4.301573	1.609883	H	-1.699451	4.348375	1.408078
H	-1.55041	2.678055	2.207651	H	2.719545	3.438424	0.099829
H	-0.619036	4.187351	2.174226	H	1.729955	4.902491	0.03714
H	0.207096	2.630592	2.409824	H	1.891407	3.886511	-1.406178
H	-1.613267	3.655254	-1.418297	H	-0.325386	2.475157	2.074182
H	-1.556357	4.820163	-0.085597	H	0.587524	3.999127	2.135393
H	-2.607894	3.400368	0.030223	H	1.444077	2.450135	2.023259
H	-8.15649	-2.068101	-0.009389	H	7.901938	-2.068716	1.444298
H	-6.672174	-2.818344	-0.63819	H	6.713947	-1.440939	2.612156
H	-7.140406	-3.058722	1.064022	H	6.641437	-3.140949	2.096423
H	2.838212	-1.044931	1.536536	H	-3.311561	-0.040978	-1.790251
H	3.272131	-2.476888	0.612209	H	-3.573364	-1.776452	-1.673561
H	3.592165	-0.725291	-1.382074	H	-3.10847	-1.383214	0.922297
H	5.255592	-1.50952	1.949643	H	-5.7622	-0.381793	-1.614609
H	5.521794	0.224804	1.686059	H	-5.765509	0.909145	-0.397529
H	7.680635	-0.371092	1.065806	H	-7.68824	-0.101782	0.427566
H	4.095946	1.351151	-0.495387	H	-3.646428	0.799805	1.474691
H	6.753899	-2.483962	-0.654515	H	-6.503338	-2.721069	0.384864
H	5.341669	-2.413676	-1.736005	H	-4.830741	-3.128414	0.837587
H	5.200522	-3.116447	-0.107916	H	-5.262241	-2.835579	-0.863364
Free Energy = -1857.584306 Hartree				Free Energy = -1857.584082 Hartree			

Table S26. The Cartesian coordinates of the lowest energy conformers (Conf. **1-6**) for isomer **4c**.

conf. 4c-1	X axis(Å)	Y axis(Å)	Z axis(Å)	conf. 4c-1	X axis(Å)	Y axis(Å)	Z axis(Å)
C	-0.116548	-2.767604	-1.621877	C	-0.359334	-2.745101	-1.728915
C	1.253047	-2.447034	-1.535044	C	1.0217	-2.491166	-1.602852
C	1.696743	-1.184315	-1.135227	C	1.518432	-1.246006	-1.210894
C	0.69219	-0.260324	-0.794777	C	0.552584	-0.264612	-0.917535
C	-0.695299	-0.560102	-0.854279	C	-0.844612	-0.494772	-1.029997
C	-1.095314	-1.834225	-1.301499	C	-1.297794	-1.7556	-1.462283
N	0.80975	1.064046	-0.423922	N	0.715469	1.055009	-0.557987
C	-0.434935	1.621215	-0.228645	C	-0.507295	1.680612	-0.42828
C	-1.406556	0.649452	-0.484334	C	-1.512815	0.753641	-0.710764
C	-2.847112	0.859485	-0.438299	C	-2.959389	0.967501	-0.717523

C	-3.78914	0.026233	0.062699	C	-3.890714	0.188294	-0.121848
C	-5.22693	0.422538	-0.001382	C	-5.335832	0.5381	-0.260106
N	-6.11175	-0.482436	0.520935	N	-6.211315	-0.283026	0.393409
C	-5.844258	-1.878167	0.862566	C	-5.931356	-1.539027	1.086062
C	-4.371261	-2.11287	1.187783	C	-4.439465	-1.75666	1.328627
N	-3.487182	-1.17386	0.737823	N	-3.571965	-0.913942	0.693798
C	-0.597051	3.080831	0.192563	C	-0.53341	3.118184	0.089668
C	-1.273165	3.820173	-0.959572	C	0.671793	3.853781	-0.486209
C	-2.426551	4.49325	-0.926762	C	1.675662	4.422552	0.187778
C	0.780526	3.752219	0.419287	C	-0.521593	3.085381	1.637158
C	-1.395624	3.166517	1.511876	C	-1.790573	3.891039	-0.378945
C	-6.305595	-2.854227	-0.237495	C	-6.529979	-2.749816	0.346597
C	3.174175	-0.842393	-1.156059	C	3.011891	-0.983974	-1.186957
C	3.807891	-0.552268	0.223799	C	3.616112	-0.7296	0.212608
C	5.337499	-0.816161	0.26458	C	5.13326	-1.050794	0.294443
C	5.685065	-2.29683	0.14007	C	5.430777	-2.5418	0.163446
C	6.120838	0.068265	-0.719901	C	5.976947	-0.185649	-0.656374
O	7.524165	-0.123559	-0.670504	O	7.370839	-0.427807	-0.567221
H	3.346614	-1.205197	0.976804	H	3.110274	-1.372964	0.945287
Cl	5.887747	-0.296357	1.990625	Cl	5.650781	-0.569374	2.041789
O	-4.011708	-3.117587	1.792873	O	-4.053385	-2.668938	2.051756
O	-5.601551	1.504008	-0.455013	O	-5.722981	1.51666	-0.899169
H	-0.407845	-3.75841	-1.960006	H	-0.689251	-3.727608	-2.055642
H	1.988242	-3.198334	-1.816103	H	1.725726	-3.284776	-1.844836
H	-2.146306	-2.083985	-1.407502	H	-2.357829	-1.950501	-1.595592
H	1.693217	1.4604	-0.123102	H	1.60378	1.444489	-0.259518
H	-3.237821	1.799002	-0.818861	H	-3.370461	1.819183	-1.248523
H	-7.082362	-0.200194	0.433459	H	-7.184512	-0.025388	0.267557
H	-6.386098	-2.111312	1.786169	H	-6.379946	-1.488768	2.086622
H	-2.511652	-1.370918	0.942978	H	-2.588469	-1.106457	0.863934
H	-0.716852	3.776239	-1.897652	H	0.67186	3.918751	-1.575826
H	-2.806131	4.990233	-1.815737	H	2.472568	4.945118	-0.335328
H	-3.042741	4.565748	-0.034846	H	1.743968	4.400386	1.27222
H	1.339573	3.260932	1.225768	H	-1.407888	2.558021	2.004082
H	0.628276	4.796592	0.709233	H	-0.537084	4.102569	2.045924
H	1.396404	3.749238	-0.487599	H	0.362189	2.569132	2.028044
H	-2.393315	2.729532	1.419147	H	-1.89357	3.87192	-1.469668
H	-1.504414	4.211406	1.824819	H	-1.705081	4.937214	-0.065566
H	-0.865916	2.627764	2.305489	H	-2.704017	3.483448	0.06211
H	-7.379551	-2.734082	-0.420811	H	-7.614593	-2.629249	0.243175
H	-5.774403	-2.663297	-1.176691	H	-6.097922	-2.846745	-0.65552
H	-6.117792	-3.885837	0.075645	H	-6.331808	-3.665113	0.911571
H	3.696789	-1.678266	-1.628866	H	3.503699	-1.845903	-1.64602

H	3.351367	0.038207	-1.789155	H	3.253667	-0.112604	-1.811194
O	3.504383	0.815625	0.529768	O	3.352782	0.644284	0.518866
H	6.752845	-2.451659	0.306555	H	6.486629	-2.737702	0.360246
H	5.454353	-2.654106	-0.869804	H	5.218073	-2.879905	-0.857042
H	5.120731	-2.896326	0.860978	H	4.823281	-3.127813	0.860033
H	5.851693	1.119891	-0.567555	H	5.740244	0.873229	-0.500837
H	5.824986	-0.215157	-1.735707	H	5.702457	-0.448552	-1.683669
H	7.848514	0.203042	0.185011	H	7.679635	-0.1237	0.302228
H	3.854046	1.012823	1.416059	H	3.66775	0.822708	1.421546
Free Energy = -1857.584355 Hartree				Free Energy = -1857.584224 Hartree			

conf. 4c-3	X axis(Å)	Y axis(Å)	Z axis(Å)	conf. 4c-4	X axis(Å)	Y axis(Å)	Z axis(Å)
C	-0.35308	-2.792253	-1.564526	C	-0.101974	-2.869926	-1.469457
C	1.028903	-2.528673	-1.482841	C	1.267439	-2.545974	-1.39398
C	1.527662	-1.2755	-1.119365	C	1.710125	-1.257245	-1.086735
C	0.564249	-0.297191	-0.81033	C	0.704535	-0.308408	-0.827322
C	-0.834785	-0.539278	-0.867903	C	-0.682899	-0.610448	-0.87376
C	-1.290297	-1.80728	-1.27672	C	-1.081565	-1.913819	-1.227977
N	0.732382	1.029956	-0.474866	N	0.821449	1.040175	-0.557053
C	-0.487585	1.644681	-0.299597	C	-0.424425	1.611349	-0.412445
C	-1.497643	0.708971	-0.537185	C	-1.394895	0.624076	-0.597645
C	-2.934656	0.95978	-0.545564	C	-2.836988	0.83286	-0.579519
C	-3.907461	0.186166	-0.011165	C	-3.783042	0.034196	-0.033399
C	-5.333922	0.605581	-0.148857	C	-5.22028	0.418227	-0.15377
N	-6.25272	-0.229852	0.424711	N	-6.111208	-0.438311	0.435925
C	-6.028676	-1.590949	0.908643	C	-5.848043	-1.79917	0.899732
C	-4.565932	-1.838503	1.270018	C	-4.377756	-2.007323	1.254013
N	-3.648771	-0.964967	0.757688	N	-3.488376	-1.110973	0.733565
C	-0.548742	3.132812	0.046063	C	-0.580385	3.104847	-0.13723
C	0.772955	3.557097	0.675383	C	-1.579169	3.30711	0.996389
C	1.663308	4.428719	0.193036	C	-2.655421	4.096796	0.997204
C	-1.643563	3.417206	1.108746	C	-1.002979	3.81473	-1.446056
C	-0.863373	3.929002	-1.242061	C	0.761789	3.722557	0.335786
C	-6.513562	-2.657275	-0.09271	C	-6.303688	-2.866009	-0.115049
C	3.019318	-1.003388	-1.144984	C	3.188577	-0.920509	-1.115597
C	3.665053	-0.73701	0.233444	C	3.804307	-0.521178	0.245305
C	5.187151	-1.042871	0.268514	C	5.330265	-0.795039	0.333047
C	5.495418	-2.531442	0.135588	C	5.662979	-2.284203	0.349633
C	5.991399	-0.174551	-0.71304	C	6.136868	-0.012077	-0.716434
O	7.389826	-0.401676	-0.666369	O	7.537562	-0.208343	-0.626583
H	3.188817	-1.38197	0.984338	H	3.324956	-1.103969	1.043273
Cl	5.755178	-0.546952	1.99626	Cl	5.861983	-0.12555	2.012754

O	-4.242935	-2.800053	1.959182	O	-4.024637	-2.958133	1.944177
O	-5.668306	1.651548	-0.705894	O	-5.590671	1.45074	-0.712687
H	-0.686504	-3.779342	-1.873451	H	-0.392497	-3.882658	-1.735638
H	1.731007	-3.319435	-1.739124	H	2.003682	-3.317596	-1.609503
H	-2.351385	-2.012934	-1.37813	H	-2.132002	-2.169552	-1.324469
H	1.627075	1.422702	-0.201024	H	1.702684	1.454636	-0.274242
H	-3.306873	1.863242	-1.020112	H	-3.236072	1.738808	-1.022853
H	-7.213816	0.066937	0.291696	H	-7.080812	-0.16891	0.306582
H	-6.583519	-1.713964	1.845861	H	-6.396458	-1.951193	1.836339
H	-2.681213	-1.173458	0.988744	H	-2.515569	-1.288523	0.96727
H	0.971854	3.096543	1.64573	H	-1.335283	2.752426	1.904084
H	2.561656	4.676045	0.753241	H	-3.281781	4.181756	1.881159
H	1.537258	4.931924	-0.761768	H	-2.973315	4.664579	0.127069
H	-2.642996	3.174686	0.740585	H	-0.257829	3.631342	-2.227728
H	-1.626394	4.480224	1.374445	H	-1.073562	4.89791	-1.292817
H	-1.468901	2.833412	2.019675	H	-1.969853	3.458606	-1.813817
H	-0.104761	3.760303	-2.013828	H	1.125192	3.249953	1.256158
H	-0.912395	5.0032	-1.02885	H	0.614397	4.78644	0.545592
H	-1.830193	3.623045	-1.653158	H	1.540079	3.641591	-0.432974
H	-7.58295	-2.525827	-0.294053	H	-7.376726	-2.762672	-0.313552
H	-5.973372	-2.574323	-1.042408	H	-5.767631	-2.755359	-1.064305
H	-6.356282	-3.657603	0.321868	H	-6.117435	-3.86698	0.286041
H	3.502194	-1.865255	-1.613602	H	3.715616	-1.791584	-1.514049
H	3.235816	-0.134735	-1.78214	H	3.376608	-0.093015	-1.813817
O	3.397849	0.635409	0.542237	O	3.51007	0.870404	0.429102
H	6.559001	-2.715562	0.29933	H	6.727468	-2.433284	0.541062
H	5.253511	-2.877168	-0.875849	H	5.437874	-2.731084	-0.625082
H	4.916574	-3.1197	0.854325	H	5.085405	-2.809081	1.116757
H	5.748526	0.88272	-0.55607	H	5.874457	1.051115	-0.667153
H	5.687414	-0.446446	-1.729619	H	5.85467	-0.387188	-1.706082
H	7.722032	-0.090802	0.191998	H	7.850859	0.19217	0.201179
H	3.740276	0.820846	1.433427	H	3.847279	1.139569	1.301163
Free Energy = -1857.584099 Hartree				Free Energy = -1857.584041 Hartree			

**conf. 4c-5**	**X axis(Å)**	**Y axis(Å)**	**Z axis(Å)**	**conf. 4c-6**	**X axis(Å)**	**Y axis(Å)**	**Z axis(Å)**
C	0.043433	-3.051008	-0.814423	C	0.14999	-3.014732	-0.657249
C	-1.268269	-2.807532	-0.359685	C	-1.164413	-2.755635	-0.218919
C	-1.686727	-1.542609	0.059008	C	-1.590126	-1.47669	0.146667
C	-0.715743	-0.525803	0.001932	C	-0.622121	-0.457813	0.052567
C	0.626924	-0.750923	-0.403508	C	0.720499	-0.69709	-0.346284
C	0.995344	-2.038567	-0.839679	C	1.096533	-2.000041	-0.72615
N	-0.832689	0.815559	0.30179	N	-0.738149	0.896377	0.293613

C	0.361531	1.465337	0.080815	C	0.45776	1.535551	0.052906
C	1.312703	0.526023	-0.327333	C	1.405361	0.582267	-0.329947
C	2.701787	0.811335	-0.65975	C	2.785204	0.838012	-0.725808
C	3.79674	0.069538	-0.371764	C	3.885979	0.122046	-0.398816
C	5.135631	0.534681	-0.838376	C	5.215475	0.539218	-0.934754
N	6.186399	-0.279257	-0.505643	N	6.269793	-0.250493	-0.562591
C	6.102264	-1.674779	-0.075106	C	6.192443	-1.600654	-0.004371
C	4.799885	-1.948216	0.681499	C	4.907301	-1.799715	0.803715
N	3.769608	-1.084392	0.436351	N	3.874162	-0.953711	0.509657
C	0.490582	2.973681	0.285329	C	0.550383	3.055305	0.192481
C	1.687542	3.251434	1.188975	C	-0.588506	3.548982	1.076741
C	2.717162	4.064752	0.943335	C	-1.58676	4.369313	0.737276
C	-0.756067	3.541173	1.013693	C	0.512428	3.690701	-1.217402
C	0.600621	3.671371	-1.090777	C	1.86223	3.472599	0.910893
C	7.322707	-2.05035	0.767594	C	7.431662	-1.903765	0.840027
C	-3.092622	-1.329367	0.587076	C	-3.008245	-1.250333	0.636364
C	-4.003959	-0.437462	-0.287377	C	-3.83154	-0.228085	-0.188083
C	-5.516627	-0.729933	-0.094862	C	-5.349996	-0.510345	-0.318246
C	-5.927968	-2.095682	-0.63723	C	-6.067212	0.607627	-1.072931
C	-5.990161	-0.491158	1.348515	C	-5.63908	-1.883646	-0.958202
O	-7.378035	-0.704938	1.539215	O	-7.017171	-2.138085	-1.160786
H	-3.772463	-0.614026	-1.346331	H	-3.450803	-0.205994	-1.216611
Cl	-6.410504	0.543146	-1.160298	Cl	-6.063828	-0.560495	1.415371
O	4.677192	-2.915554	1.425106	O	4.798627	-2.698902	1.629607
O	5.303593	1.593033	-1.444123	O	5.370567	1.54643	-1.625381
H	0.31352	-4.05153	-1.141287	H	0.423862	-4.027237	-0.94136
H	-1.976187	-3.633244	-0.326504	H	-1.871244	-3.580664	-0.159053
H	1.999545	-2.239843	-1.199205	H	2.102014	-2.21021	-1.07715
H	-1.731952	1.255259	0.462301	H	-1.627311	1.360615	0.464648
H	2.931495	1.736449	-1.177139	H	2.997664	1.700298	-1.35122
H	7.077721	0.037324	-0.873044	H	7.155404	0.032353	-0.969264
H	6.072364	-2.336522	-0.95869	H	6.140214	-2.339439	-0.823275
H	2.902126	-1.307839	0.915746	H	3.013169	-1.136304	1.017765
H	1.646846	2.73049	2.147049	H	-0.535156	3.195115	2.108463
H	3.504474	4.202817	1.679618	H	-2.325821	4.681364	1.471106
H	2.836876	4.601643	0.00635	H	-1.709669	4.763987	-0.267533
H	-1.66825	3.416953	0.416431	H	1.354862	3.337644	-1.819943
H	-0.617717	4.613856	1.180869	H	0.581575	4.782656	-1.149824
H	-0.910364	3.067135	1.989697	H	-0.409509	3.432837	-1.75003
H	1.483987	3.350254	-1.64968	H	1.934015	3.006698	1.900192
H	0.652993	4.759129	-0.966132	H	1.874073	4.560358	1.043251
H	-0.281722	3.44235	-1.698519	H	2.74951	3.189481	0.340211
H	8.241035	-1.924647	0.181732	H	8.336325	-1.832061	0.224614

H	7.385736	-1.417261	1.658758	H	7.515652	-1.196081	1.671367
H	7.248512	-3.093145	1.083824	H	7.364158	-2.914919	1.247322
H	-3.550595	-2.315485	0.701832	H	-3.511369	-2.220739	0.655812
H	-3.059005	-0.879196	1.588868	H	-3.001954	-0.901103	1.678266
O	-3.687164	0.920098	0.049623	O	-3.606527	1.102764	0.298084
H	-7.013312	-2.208154	-0.599979	H	-7.132859	0.387266	-1.157955
H	-5.493415	-2.8915	-0.02194	H	-5.650333	0.674033	-2.086435
H	-5.590673	-2.228623	-1.669857	H	-5.928113	1.573285	-0.584948
H	-5.687355	0.509723	1.677529	H	-5.180437	-2.689207	-0.374246
H	-5.492565	-1.22577	1.990734	H	-5.186857	-1.887701	-1.95683
H	-7.863615	-0.014984	1.057618	H	-7.44236	-2.231551	-0.29206
H	-4.217672	1.504814	-0.519044	H	-4.049733	1.1823	1.161601
Free Energy = -1857.582862 Hartree				Free Energy = -1857.581969 Hartree			

Table S27. The Cartesian coordinates of the lowest energy conformers (Conf. **1-6**) for isomer **4d**.

**conf. 4d-1**	**X axis(Å)**	**Y axis(Å)**	**Z axis(Å)**	**conf. 4d-2**	**X axis(Å)**	**Y axis(Å)**	**Z axis(Å)**
C	-0.218399	-2.701511	-1.624166	C	-0.359334	-2.745101	-1.728915
C	1.153772	-2.400246	-1.507694	C	1.0217	-2.491166	-1.602852
C	1.609332	-1.143896	-1.101307	C	1.518432	-1.246006	-1.210894
C	0.610378	-0.202865	-0.785373	C	0.552584	-0.264612	-0.917535
C	-0.779603	-0.486119	-0.870022	C	-0.844612	-0.494772	-1.029997
C	-1.189901	-1.755058	-1.322434	C	-1.297794	-1.7556	-1.462283
N	0.731975	1.117705	-0.403893	N	0.715469	1.055009	-0.557987
C	-0.508575	1.689082	-0.230747	C	-0.507295	1.680612	-0.42828
C	-1.485472	0.729456	-0.50941	C	-1.512815	0.753641	-0.710764
C	-2.929655	0.934479	-0.518409	C	-2.959389	0.967501	-0.717523
C	-3.880199	0.113887	-0.014682	C	-3.890714	0.188294	-0.121848
C	-5.318247	0.493913	-0.145164	C	-5.335832	0.5381	-0.260106
N	-6.213314	-0.386989	0.396965	N	-6.211315	-0.283026	0.393409
C	-5.950303	-1.753498	0.844387	C	-5.931356	-1.539027	1.086062
C	-4.481067	-1.969483	1.199389	C	-4.439465	-1.75666	1.328627
N	-3.589351	-1.053859	0.716572	N	-3.571965	-0.913942	0.693798
C	-0.624091	3.162624	0.160949	C	-0.53341	3.118184	0.089668
C	0.693677	3.626381	0.769873	C	0.671793	3.853781	-0.486209
C	1.515362	4.57399	0.309158	C	1.675662	4.422552	0.187778
C	-1.702905	3.362684	1.259639	C	-0.521593	3.085381	1.637158
C	-1.007871	3.984636	-1.091393	C	-1.790573	3.891039	-0.378945
C	-6.405116	-2.80576	-0.185505	C	-6.529979	-2.749816	0.346597
C	3.095275	-0.836777	-1.074895	C	3.011891	-0.983974	-1.186957
C	3.644398	-0.352518	0.290588	C	3.616112	-0.7296	0.212608
C	5.100372	-0.760065	0.632766	C	5.13326	-1.050794	0.294443

C	5.552181	-0.163031	1.964605	C	5.430777	-2.5418	0.163446
C	5.304689	-2.288931	0.614519	C	5.976947	-0.185649	-0.656374
O	6.604549	-2.693319	1.003504	O	7.370839	-0.427807	-0.567221
H	3.027757	-0.771605	1.095112	H	3.110274	-1.372964	0.945287
Cl	6.21047	-0.051107	-0.702081	Cl	5.650781	-0.569374	2.041789
O	-4.130474	-2.942596	1.85824	O	-4.053385	-2.668938	2.051756
O	-5.682603	1.546452	-0.670116	O	-5.722981	1.51666	-0.899169
H	-0.516212	-3.689516	-1.964919	H	-0.689251	-3.727608	-2.055642
H	1.883461	-3.164667	-1.766542	H	1.725726	-3.284776	-1.844836
H	-2.242714	-1.990635	-1.443376	H	-2.357829	-1.950501	-1.595592
H	1.616766	1.539287	-0.133278	H	1.60378	1.444489	-0.259518
H	-3.328264	1.841971	-0.962785	H	-3.370461	1.819183	-1.248523
H	-7.182429	-0.1148	0.269819	H	-7.184512	-0.025388	0.267557
H	-6.501089	-1.917724	1.777767	H	-6.379946	-1.488768	2.086622
H	-2.616455	-1.241425	0.943009	H	-2.588469	-1.106457	0.863934
H	0.955429	3.125757	1.704377	H	0.67186	3.918751	-1.575826
H	2.416237	4.842122	0.855693	H	2.472568	4.945118	-0.335328
H	1.326883	5.122155	-0.610009	H	1.743968	4.400386	1.27222
H	-2.697266	3.074451	0.910439	H	-1.407888	2.558021	2.004082
H	-1.735137	4.418468	1.55142	H	-0.537084	4.102569	2.045924
H	-1.472197	2.7678	2.150663	H	0.362189	2.569132	2.028044
H	-0.261565	3.877746	-1.886009	H	-1.89357	3.87192	-1.469668
H	-1.100718	5.047755	-0.840616	H	-1.705081	4.937214	-0.065566
H	-1.969805	3.649742	-1.490624	H	-2.704017	3.483448	0.06211
H	-7.477463	-2.698003	-0.385173	H	-7.614593	-2.629249	0.243175
H	-5.866276	-2.683322	-1.13169	H	-6.097922	-2.846745	-0.65552
H	-6.221168	-3.811971	0.202987	H	-6.331808	-3.665113	0.911571
H	3.624836	-1.73536	-1.402584	H	3.503699	-1.845903	-1.64602
H	3.338849	-0.064552	-1.81808	H	3.253667	-0.112604	-1.811194
O	3.477157	1.067	0.422419	O	3.352782	0.644284	0.518866
H	6.576479	-0.465478	2.189856	H	6.486629	-2.737702	0.360246
H	4.900524	-0.544245	2.761742	H	5.218073	-2.879905	-0.857042
H	5.482098	0.925604	1.960225	H	4.823281	-3.127813	0.860033
H	5.050449	-2.703162	-0.367248	H	5.740244	0.873229	-0.500837
H	4.623986	-2.725879	1.354567	H	5.702457	-0.448552	-1.683669
H	7.23196	-2.406645	0.319222	H	7.679635	-0.1237	0.302228
H	4.112668	1.498405	-0.176501	H	3.66775	0.822708	1.421546
Free Energy = -1857.58508 Hartree				Free Energy = -1857.584007Hartree			

conf. 4d-3	X axis(Å)	Y axis(Å)	Z axis(Å)	conf. 4d-4	X axis(Å)	Y axis(Å)	Z axis(Å)
C	0.14999	-3.014732	-0.657249	C	-0.085568	-3.028064	-0.67722
C	-1.164413	-2.755635	-0.218919	C	-1.388367	-2.726488	-0.231023

C	-1.590126	-1.47669	0.146667	C	-1.767148	-1.434917	0.14145
C	-0.622121	-0.457813	0.052567	C	-0.76391	-0.451388	0.046717
C	0.720499	-0.69709	-0.346284	C	0.567736	-0.732186	-0.361291
C	1.096533	-2.000041	-0.72615	C	0.895255	-2.046392	-0.748444
N	-0.738149	0.896377	0.293613	N	-0.838629	0.905879	0.287426
C	0.45776	1.535551	0.052906	C	0.373176	1.508866	0.034251
C	1.405361	0.582267	-0.329947	C	1.291117	0.525729	-0.345496
C	2.785204	0.838012	-0.725808	C	2.682741	0.755737	-0.709369
C	3.885979	0.122046	-0.398816	C	3.759655	-0.004794	-0.402846
C	5.215475	0.539218	-0.934754	C	5.104989	0.398471	-0.906698
N	6.269793	-0.250493	-0.562591	N	6.136333	-0.42936	-0.547973
C	6.192443	-1.600654	-0.004371	C	6.017992	-1.800742	-0.052706
C	4.907301	-1.799715	0.803715	C	4.717865	-2.001381	0.730098
N	3.874162	-0.953711	0.509657	N	3.709897	-1.119617	0.457598
C	0.550383	3.055305	0.192481	C	0.548716	3.019592	0.175907
C	-0.588506	3.548982	1.076741	C	1.777753	3.299648	1.034363
C	-1.58676	4.369313	0.737276	C	2.814921	4.084409	0.733615
C	0.512428	3.690701	-1.217402	C	-0.662055	3.649111	0.914268
C	1.86223	3.472599	0.910893	C	0.638806	3.658459	-1.230047
C	7.431662	-1.903765	0.840027	C	7.237543	-2.172706	0.792901
C	-3.008245	-1.250333	0.636364	C	-3.172982	-1.159034	0.642978
C	-3.83154	-0.228085	-0.188083	C	-3.946738	-0.056816	-0.124791
C	-5.349996	-0.510345	-0.318246	C	-5.475149	-0.264237	-0.273613
C	-6.067212	0.607627	-1.072931	C	-6.13915	0.922041	-0.97065
C	-5.63908	-1.883646	-0.958202	C	-5.821617	-1.588104	-0.986069
O	-7.017171	-2.138085	-1.160786	O	-7.208161	-1.766205	-1.209743
H	-3.450803	-0.205994	-1.216611	H	-3.559992	0.009634	-1.148982
Cl	-6.063828	-0.560495	1.415371	Cl	-6.197469	-0.373761	1.453133
O	4.798627	-2.698902	1.629607	O	4.576709	-2.930067	1.51838
O	5.370567	1.54643	-1.625381	O	5.295511	1.421814	-1.563655
H	0.423862	-4.027237	-0.94136	H	0.151643	-4.047866	-0.968026
H	-1.871244	-3.580664	-0.159053	H	-2.123136	-3.526779	-0.172196
H	2.102014	-2.21021	-1.07715	H	1.889756	-2.290308	-1.108464
H	-1.627311	1.360615	0.464648	H	-1.713019	1.376722	0.501206
H	2.997664	1.700298	-1.35122	H	2.930527	1.649983	-1.271038
H	7.155404	0.032353	-0.969264	H	7.031447	-0.156264	-0.940235
H	6.140214	-2.339439	-0.823275	H	5.958468	-2.501144	-0.904404
H	3.013169	-1.136304	1.017765	H	2.84231	-1.296377	0.955988
H	-0.535156	3.195115	2.108463	H	1.75513	2.810431	2.009639
H	-2.325821	4.681364	1.471106	H	3.626089	4.229653	1.442068
H	-1.709669	4.763987	-0.267533	H	2.917044	4.588984	-0.223215
H	1.354862	3.337644	-1.819943	H	-1.59506	3.52011	0.351966
H	0.581575	4.782656	-1.149824	H	-0.492815	4.724194	1.029692

H	-0.409509	3.432837	-1.75003	H	-0.793592	3.222625	1.915627
H	1.934015	3.006698	1.900192	H	1.496723	3.288092	-1.798245
H	1.874073	4.560358	1.043251	H	0.726133	4.748241	-1.151376
H	2.74951	3.189481	0.340211	H	-0.266957	3.432567	-1.803382
H	8.336325	-1.832061	0.224614	H	8.152149	-2.096711	0.192824
H	7.515652	-1.196081	1.671367	H	7.327398	-1.504357	1.655559
H	7.364158	-2.914919	1.247322	H	7.139445	-3.198732	1.154236
H	-3.511369	-2.220739	0.655812	H	-3.725297	-2.101891	0.61557
H	-3.001954	-0.901103	1.678266	H	-3.1492	-0.864238	1.701495
O	-3.606527	1.102764	0.298084	O	-3.669818	1.23645	0.43864
H	-7.132859	0.387266	-1.157955	H	-7.212604	0.751982	-1.071288
H	-5.650333	0.674033	-2.086435	H	-5.714133	1.023946	-1.977839
H	-5.928113	1.573285	-0.584948	H	-5.962757	1.85372	-0.431392
H	-5.180437	-2.689207	-0.374246	H	-5.40627	-2.443278	-0.441742
H	-5.186857	-1.887701	-1.95683	H	-5.361456	-1.562404	-1.980718
H	-7.44236	-2.231551	-0.29206	H	-7.644188	-1.890259	-0.350301
H	-4.049733	1.1823	1.161601	H	-4.107441	1.28053	1.307678
Free Energy = -1857.583475 Hartree				Free Energy = -1857.583446 Hartree			

**conf. 4d-5**	**X axis(Å)**	**Y axis(Å)**	**Z axis(Å)**	**conf. 4d-6**	**X axis(Å)**	**Y axis(Å)**	**Z axis(Å)**
C	-0.35308	-2.792253	-1.564526	C	-0.116548	-2.767604	-1.621877
C	1.028903	-2.528673	-1.482841	C	1.253047	-2.447034	-1.535044
C	1.527662	-1.2755	-1.119365	C	1.696743	-1.184315	-1.135227
C	0.564249	-0.297191	-0.81033	C	0.69219	-0.260324	-0.794777
C	-0.834785	-0.539278	-0.867903	C	-0.695299	-0.560102	-0.854279
C	-1.290297	-1.80728	-1.27672	C	-1.095314	-1.834225	-1.301499
N	0.732382	1.029956	-0.474866	N	0.80975	1.064046	-0.423922
C	-0.487585	1.644681	-0.299597	C	-0.434935	1.621215	-0.228645
C	-1.497643	0.708971	-0.537185	C	-1.406556	0.649452	-0.484334
C	-2.934656	0.95978	-0.545564	C	-2.847112	0.859485	-0.438299
C	-3.907461	0.186166	-0.011165	C	-3.78914	0.026233	0.062699
C	-5.333922	0.605581	-0.148857	C	-5.22693	0.422538	-0.001382
N	-6.25272	-0.229852	0.424711	N	-6.11175	-0.482436	0.520935
C	-6.028676	-1.590949	0.908643	C	-5.844258	-1.878167	0.862566
C	-4.565932	-1.838503	1.270018	C	-4.371261	-2.11287	1.187783
N	-3.648771	-0.964967	0.757688	N	-3.487182	-1.17386	0.737823
C	-0.548742	3.132812	0.046063	C	-0.597051	3.080831	0.192563
C	0.772955	3.557097	0.675383	C	-1.273165	3.820173	-0.959572
C	1.663308	4.428719	0.193036	C	-2.426551	4.49325	-0.926762
C	-1.643563	3.417206	1.108746	C	0.780526	3.752219	0.419287
C	-0.863373	3.929002	-1.242061	C	-1.395624	3.166517	1.511876
C	-6.513562	-2.657275	-0.09271	C	-6.305595	-2.854227	-0.237495

C	3.019318	-1.003388	-1.144984	C	3.174175	-0.842393	-1.156059
C	3.665053	-0.73701	0.233444	C	3.807891	-0.552268	0.223799
C	5.187151	-1.042871	0.268514	C	5.337499	-0.816161	0.26458
C	5.495418	-2.531442	0.135588	C	5.685065	-2.29683	0.14007
C	5.991399	-0.174551	-0.71304	C	6.120838	0.068265	-0.719901
O	7.389826	-0.401676	-0.666369	O	7.524165	-0.123559	-0.670504
H	3.188817	-1.38197	0.984338	H	3.346614	-1.205197	0.976804
Cl	5.755178	-0.546952	1.99626	Cl	5.887747	-0.296357	1.990625
O	-4.242935	-2.800053	1.959182	O	-4.011708	-3.117587	1.792873
O	-5.668306	1.651548	-0.705894	O	-5.601551	1.504008	-0.455013
H	-0.686504	-3.779342	-1.873451	H	-0.407845	-3.75841	-1.960006
H	1.731007	-3.319435	-1.739124	H	1.988242	-3.198334	-1.816103
H	-2.351385	-2.012934	-1.37813	H	-2.146306	-2.083985	-1.407502
H	1.627075	1.422702	-0.201024	H	1.693217	1.4604	-0.123102
H	-3.306873	1.863242	-1.020112	H	-3.237821	1.799002	-0.818861
H	-7.213816	0.066937	0.291696	H	-7.082362	-0.200194	0.433459
H	-6.583519	-1.713964	1.845861	H	-6.386098	-2.111312	1.786169
H	-2.681213	-1.173458	0.988744	H	-2.511652	-1.370918	0.942978
H	0.971854	3.096543	1.64573	H	-0.716852	3.776239	-1.897652
H	2.561656	4.676045	0.753241	H	-2.806131	4.990233	-1.815737
H	1.537258	4.931924	-0.761768	H	-3.042741	4.565748	-0.034846
H	-2.642996	3.174686	0.740585	H	1.339573	3.260932	1.225768
H	-1.626394	4.480224	1.374445	H	0.628276	4.796592	0.709233
H	-1.468901	2.833412	2.019675	H	1.396404	3.749238	-0.487599
H	-0.104761	3.760303	-2.013828	H	-2.393315	2.729532	1.419147
H	-0.912395	5.0032	-1.02885	H	-1.504414	4.211406	1.824819
H	-1.830193	3.623045	-1.653158	H	-0.865916	2.627764	2.305489
H	-7.58295	-2.525827	-0.294053	H	-7.379551	-2.734082	-0.420811
H	-5.973372	-2.574323	-1.042408	H	-5.774403	-2.663297	-1.176691
H	-6.356282	-3.657603	0.321868	H	-6.117792	-3.885837	0.075645
H	3.502194	-1.865255	-1.613602	H	3.696789	-1.678266	-1.628866
H	3.235816	-0.134735	-1.78214	H	3.351367	0.038207	-1.789155
O	3.397849	0.635409	0.542237	O	3.504383	0.815625	0.529768
H	6.559001	-2.715562	0.29933	H	6.752845	-2.451659	0.306555
H	5.253511	-2.877168	-0.875849	H	5.454353	-2.654106	-0.869804
H	4.916574	-3.1197	0.854325	H	5.120731	-2.896326	0.860978
H	5.748526	0.88272	-0.55607	H	5.851693	1.119891	-0.567555
H	5.687414	-0.446446	-1.729619	H	5.824986	-0.215157	-1.735707
H	7.722032	-0.090802	0.191998	H	7.848514	0.203042	0.185011
H	3.740276	0.820846	1.433427	H	3.854046	1.012823	1.416059
Free Energy = -1857.583418 Hartree				Free Energy = -1857.582565 Hartree			
